# A Simple Access to γ‐ and ε‐Keto Arenes via Enzymatic Divergent C─H Bond Oxyfunctionalization

**DOI:** 10.1002/advs.202304605

**Published:** 2023-10-23

**Authors:** Huanhuan Li, Yalan Zhang, Yawen Huang, Peigao Duan, Ran Ge, Xiaofeng Han, Wuyuan Zhang

**Affiliations:** ^1^ School of Chemical Engineering and Technology Xi'an Jiaotong University Xi'an 710049 China; ^2^ Key Laboratory of Engineering Biology for Low‐carbon Manufacturing Tianjin Institute of Industrial Biotechnology Chinese Academy of Sciences 32 West 7th Avenue Tianjin 300308 China; ^3^ National Innovation Center for Synthetic Biotechnology 32 West 7th Avenue Tianjin 300308 China

**Keywords:** biocatalysis, cascades, C─H bond oxyfunctionalization, keto arenes, peroxygenases

## Abstract

Performing divergent C─H bond functionalization on molecules with multiple reaction sites is a significant challenge in organic chemistry. Biocatalytic oxyfunctionalization reactions of these compounds to the corresponding ketones/aldehydes are typically hindered by selectivity issues. To address these challenges, the catalytic performance of oxidoreductases is explored. The results show that combining the peroxygenase‐catalyzed propargylic C─H bond oxidation with the Old Yellow Enzyme‐catalyzed reduction of conjugated C─C triple bonds in one‐pot enables the regio‐ and chemoselective oxyfunctionalization of sp^3^ C─H bonds that are distant from benzylic sites. This enzymatic approach yielded a variety of γ‐keto arenes with diverse structural and electronic properties in yields of up to 99% and regioselectivity of 100%, which are difficult to achieve using other chemocatalysis and enzymes. By adjusting the C─C triple bond, the carbonyl group's position can be further tuned to yield ε‐keto arenes. This enzymatic approach can be combined with other biocatalysts to establish new synthetic pathways for accessing various challenging divergent C─H bond functionalization reactions.

## Graphical abstract

Peroxygenases show surprisingly high activity and selectivity in propargylic C─H bond activation. Old Yellow Enzyme is readily cascaded with peroxygenase in one‐pot synthesis, whereby a variety of carbonyl compounds with structural and electronic diversity are obtained by this simple enzymatic approach to realize the remote C─H bond oxyfunctionalization.

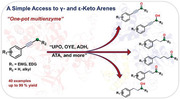

## 1 Introduction

Carbon‐hydrogen (C─H) bonds are omnipresent structural units in organic molecules, yet their selective activation is still a long‐standing challenge in organic chemistry.^[^ ^1^ ^]^ Over the past decades, substantial advances have been made in the field of C─H bond functionalization reactions by using precious transition metal complexes^[^ ^2^ ^]^ and organocatalysts.^[^ ^3^ ^]^ However, due to the wide availability of isolated and/or electronically unbiased C─H bonds in complex organic compounds, the divergent C─H bond functionalization of substrates bearing multiple reaction sites is still highly challenging with chemical catalysts.^[^ ^4^ ^]^ The activation and functionalization of more inert C─H bonds (e.g., separated from benzylic C─H, alpha C─H, and allylic C─H bonds) are of great importance to synthesize and endow the distinct function of natural products and pharmaceuticals.^[^ ^5^ ^]^

**Scheme 1:**
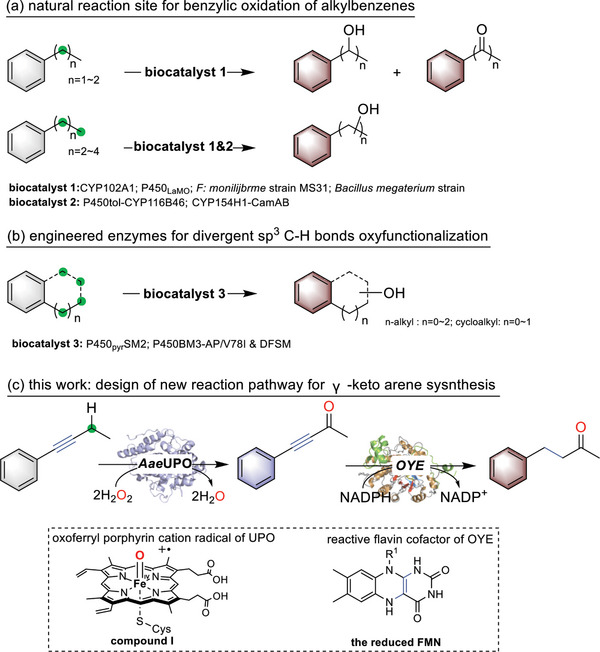
Biocatalyzed C─H bond oxyfunctionalization reaction. a) The natural reaction site of alkylbenzene by enzymes; b) engineered enzymes for non‐activated diver‐gent C─H bond oxidation of alkylbenzenes; c) the proposed enzymatic methods using *Aae*UPO catalyzed activation of the propargylic C─H bond to yield ynones and OYE‐catalyzed reduction of the C─C triple bonds.

Alternatively, a number of enzymes for C─H bond functionalization reactions are available.^[^ ^6^ ^]^ For the relatively simple alkylbenzenes such as ethylbenzene or propylbenzene, the benzylic C─H bonds are almost exclusively oxidized to give alcohols and ketones as side‐products by wild‐type enzymes such as CYP102A1^[^ ^7^ ^]^ and P450_LaMO_^[^ ^8^ ^]^ or resting cells^[^ ^9^ ^]^ (**Scheme** **1**a). However, the increase in chain length of the aliphatic substitutes not only impairs the enzyme activity but also leads to poor regioselectivity (Scheme 1a). The above issues were observed with most oxidoreductases such as monooxygenase, peroxidase, and peroxygenase.^[^ ^6^, ^10^ ^]^ To address the C─H bonds oxidation distant from the native benzylic sites in poor regioselectivity, protein engineering has been largely adopted. For example, evolved P450 enzymes have been applied to provide direct access to the oxidation of less reachable C─H bonds in artemisinin,^[^ ^11^ ^]^ sclareol,^[^ ^12^ ^]^ terpenes^[^ ^13^ ^]^ or steroids.^[^ ^14^ ^]^ The recent synergistic use of protein engineering and exogenous decoy molecules enabled P450 peroxygenases to perform divergent hydroxylation of alkylarenes at multiple sp^3^ C─H bonds^[^ ^15^ ^]^ (Scheme 1b). Also, both the wild‐type and engineered fatty acid hydroxylases P450 BM3^[^ ^16^ ^]^ and CYP152^[^ ^17^ ^]^ have demonstrated to hydroxylate the fatty acids at divergent sites from the carboxylate group for valorizing the renewable fatty acids. Despite these exciting efforts, enzymatic approaches are still scarce in divergent C─H bond functionalization of substrates with multiple reaction sites available. Therefore, there is a pressing need to expand the toolbox of enzymes for this challenging reaction, which will bring great benefits for the synthetic manipulation of complex molecules and pharmaceuticals.^[^ ^5^, ^18^ ^]^

Another class of oxidoreductase, unspecific peroxygenases such as that from *Agrocybe aegerita* (*Aae*UPO), has attracted considerable interest in the past decade.^[^ ^19^ ^]^ The peroxygenases use the oxoferryl porphyrin cation radical as the oxygenating species (so‐called compound I) to oxidize a broad range of C─H bonds.^[^ ^20^ ^]^ However, peroxygenases directly use peroxides as oxygen and electron sources to generate compound I, and the electron transport chain is significantly shortened compared to that of P450s. As a result, the simplicity of peroxygenases makes them appealing in synthetic chemistry.^[^ ^19b^ ^]^ To date, peroxygenases have enabled facile syntheses of (chiral) fine chemicals and functionalized drugs.^[^ ^21^ ^]^ However, when using alkylarene hydrocarbons as substrates, the hydroxylation reaction almost exclusively occurs at the benzylic sites (except for toluene) as it is the natural site of peroxygenase.^[^ ^22^ ^]^ Reactions of divergent C─H bond oxyfunctionalization by peroxygenases have never been truly considered due to selectivity and activity issues. Specifically, even using the simple butylbenzene peroxygenase can produce a mixture of oxidized products with significantly reduced regioselectivity compared to ethylbenzene as substrate.^[^ ^23^ ^]^

Herein, we envisioned a straightforward enzymatic approach in synthesizing γ‐keto arenes, which are valuable building blocks in organic synthesis. However, as stated starting from the divergent C─H oxyfunctionalization of alkylbenzenes, the biocatalytic methods are typically impaired by the regioselectivity issues as the benzylic sites are the native sites of an enzyme to act.^[^ ^24^ ^]^ To address this issue, we chose the direct use of alkyne as substrate for peroxygenase to yield ynone and subsequently cascaded an unusual activity of Old Yellow Enzyme (OYE) for the reduction of the C─C triple bond (Scheme 1c). The presence of a C─C triple bond directs the site selectivity of peroxygenases toward the propargylic C─H site, compared to alkylbenzenes. This is inspired by substrate engineering, which aims to control the molecular recognition of substrates by an enzyme.^[^ ^25^ ^]^ The envisioned combination of both enzymes allows an overall divergent sp^3^ C─H bond oxyfunctionalization of aromatic hydrocarbons. The well‐known function of the oxoferryl‐heme species (compound I) of peroxygenase and the reduced flavin mononucleotide (FMN) of OYE offers a great advantage in synthesizing γ‐keto arenes in excellent regioselectivity. This present strategy represents a simple strategy in γ‐keto arenes synthesis, which is also a great complement in divergent C─H bond oxyfunctionalization chemistry as the literature methods centered on alcohol synthesis. Access to products with γ‐keto arenes in one pot will lead to pathways for various challenging remote C─H bond functionalization reactions.

## 2 Results and Discussion

**Figure 1:**
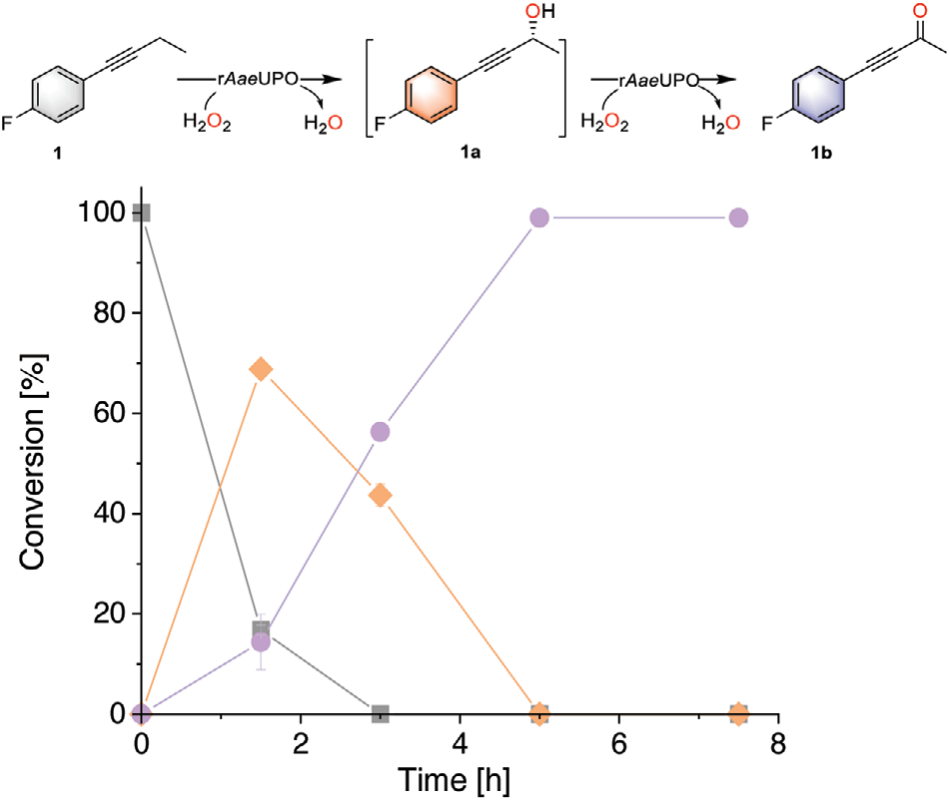
Time course of r*Aae*UPO‐catalyzed C─H bond oxyfunctionalization of 1 (■) to 1b (●) via 1a (◆). Reaction conditions: [1] = 5 mM, [r*Aae*UPO] = 500 nM, [H_2_O_2_] = 3 mM h^−1^, 30% (v/v) MeCN, NaPi buffer (100 mM, pH = 7), 30 °C, 800 rpm, 5 h, 1 mL. The conversion was determined by GC chromatography. Error bars indicate the standard deviation of duplicate experiments (*n* = 2).

To demonstrate the synthesis of γ‐keto arenes starting from the C─H bond activation, we used the recombinant unspecific peroxygenase from *Agrocybe aegerita* (r*Aae*UPO, PaDa‐I variant) and expressed it in *Pichia pastoris* following reported protocols.^[^ ^26^ ^]^ The regioselective oxidation of 1‐(but‐1‐yn‐1‐yl)−4‐fluorobenzene (1) to 4‐(4‐fluorophenyl) but‐3‐yn‐2‐one (1b) was chosen as the model reaction (**Figure** **1**). The optimal reaction conditions, i.e., a substrate concentration of 5 mM, r*Aae*UPO of 250–500 nM with an H_2_O_2_ feeding rate of 3 mM h^−1^ at pH 7, were obtained by investigating the enzyme and H_2_O_2_ concentrations (Table S1, Supporting Information). Complete conversion of the substrate 1 to 1b (4.9 mM), corresponding to the highest TON of 19 510 of the r*Aae*UPO, was achieved. The control reactions that only used H_2_O_2_ or thermally inactivated r*Aae*UPO did not show any formation of the desired product (Table S1, Supporting Information). More importantly, a control reaction using butylbenzene yielded a mixture of five oxidized products (Table S2 and Figures S1–S6, Supporting Information), suggesting the poor regioselectivity of r*Aae*UPO for divergent C─H bond oxyfunctionalization of alkylbenzenes and the necessity of presenting a C─C triple bond to butylbenzene unless protein engineering is considered. A molecular docking investigation also confirmed that due to the flexibility of *n*‐butane chain of bultylbenzene, a few configurations were possible with similar binding energies and distances toward the heme center, which could result in poor regioselectivity of butylbenzene oxidation by r*Aae*UPO (Figure S7, Supporting Information).

The reaction course clearly showed that the formation of ynone 1b underwent a stepwise oxidation of intermediate alkynol (1a) upon the supply of excessive H_2_O_2_ (Figure 1 and Figure S8, Supporting Information). The direct formation of the ynone product was expected, as alkynols are well‐accepted substrates by peroxygenase.^[^ ^21d^ ^]^ A detailed investigation using chiral chromatography showed that the oxidation of the propargylic C─H bond first yielded (*R*)−1a in 99% *ee* as an intermediate (Figure S9, Supporting Information). However, r*Aae*UPO so far has shown no stereoselectivity toward racemic alcohols,^[^ ^21d^ ^]^ which in turn is a great advantage to obtain ynone products directly for the envisioned cascade reaction.

**Figure 2:**
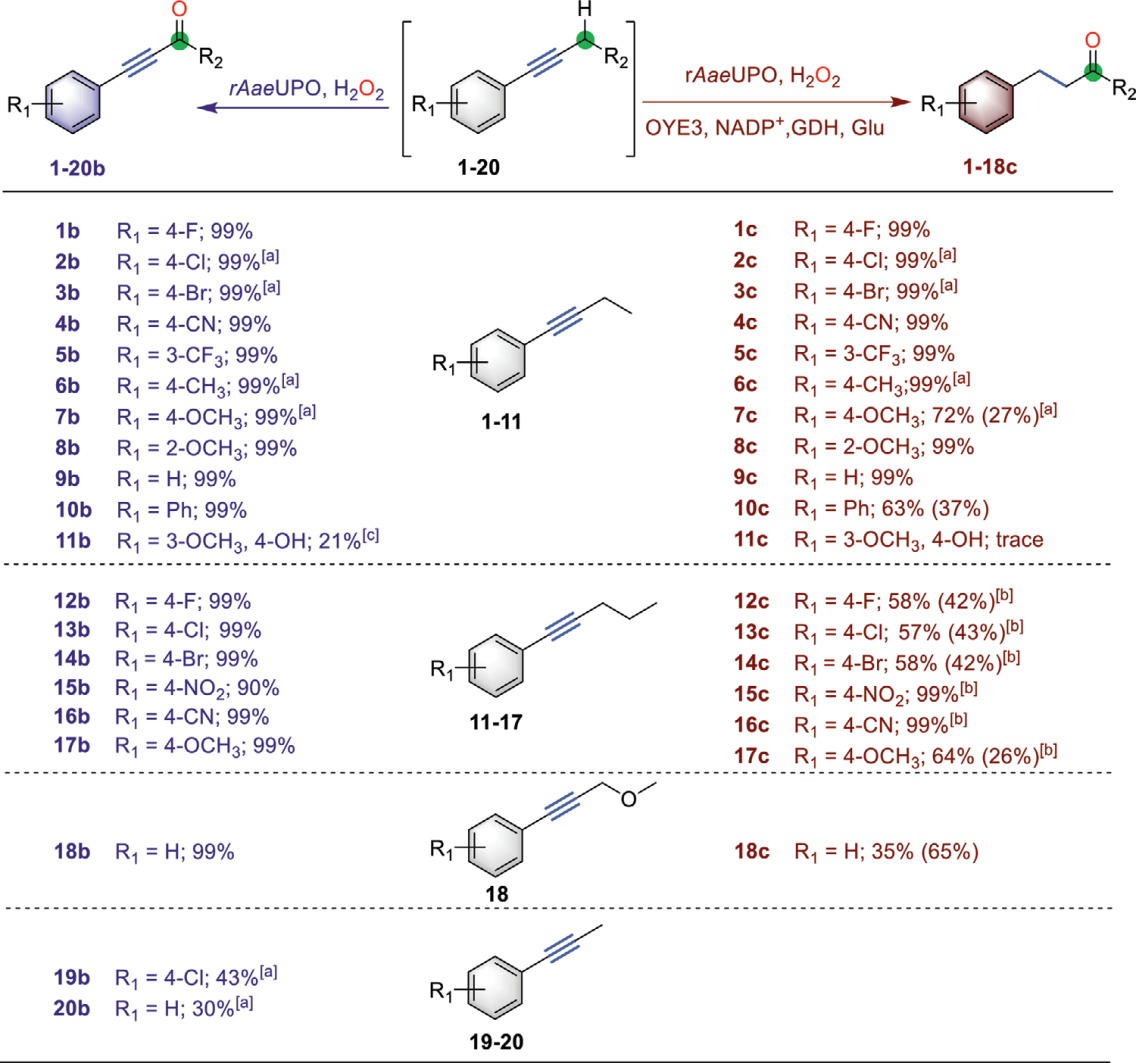
Study of the substrate scope. The scope of r*Aae*UPO‐catalyzed oxidation of alkynes to ynones and ynals (left) and the scope of the cascade between r*Aae*UPO and OYE3 for divergent C─H bond oxyfunctionalization (right), respectively. Reaction conditions unless otherwise noted: [substrates] = 5 mM, [r*Aae*UPO] = 500 nM, [H_2_O_2_] = 3 mM h^−1^, 30% (v/v) MeCN, NaPi buffer (100 mM, pH = 7), 30 °C, 800 rpm, 5 h, 1 mL or^[a]^ [r*Aae*UPO] = 2 µM. After oxidation, [OYE3] = 25 µM or^[b]^ [OYE3] = 50 µM, [d‐glucose] = 50 mM, [NADP^+^] = 2 mM, and [GDH] = 1 mg mL^−1^ were added to the reaction mixture for 24 h. The number in the bracket indicates the yield of the enone or enal intermediate. The conversion was determined by GC chromatography.

To investigate the applicability of peroxygenase‐catalyzed propargylic C─H bond oxidation to ynone synthesis, the procedure was then extended to the oxidation of a range of alkyne substrates with structural and electronic diversity (**Figure** **2**, left column), which were easily prepared by coupling terminal alkynes with aryl or vinyl halides (Figure S10, Supporting Information). Specifically, substituted internal alkynes with different side chain lengths were converted into the corresponding ketones (1b‐17b) with excellent yields and regioselectivity (Figures S11–S26, Supporting Information). There was no indication of ring‐hydroxylation, and only ketone products were obtained. Also, possible demethylation of 7, 8, 11, and 17 were not observed, and the corresponding methoxy ynones were obtained as the sole products. The naphthalene substrate was oxidized in high selectivity and yield without the observation of any aromatic C─H bond oxidation (10b). Although r*Aae*UPO showed lower activity against a few substrates, it was found that elevating the enzyme concentration and prolonging the reaction time enabled complete oxidation to ynone products (2b, 3b, 6b, 7b, 19b, and 20b). Two ynal products (19b and 20b) were obtained in relatively low yield, probably due to the lower enzyme activity on propynes (Figures S27 and S28, Supporting Information). However, using a methyl ether substrate, the yield to access to ynal (18b) was significantly enhanced via a hydrogen abstraction and oxygen rebound mechanism (Figure S29, Supporting Information).^[^ ^27^ ^]^

The excellent performance of r*Aae*UPO on the propargylic C─H bond oxidation allows the significant simplification of the downstream process. As such, the reaction could be readily scaled up to 1 mmol scale as attempted with 13 and 16. After the reaction, the desired products 13b (115 mg) and 16b (135 mg) were recovered in 60 and 74% isolated by a simple extraction and evaporation of the solvent (Figure S30, Supporting Information). Notably, to date, only a handful of methods have demonstrated the C─H bond oxyfunctionalization of alkynes using enzymes as catalysts.^[^ ^28^ ^]^ This work is the first study on the use of peroxygenase to convert a broad range of alkyne compounds into ynones and ynals in remarkable regioselectivity (up to 100%) and activity (up to 99% yield).

**Scheme 2:**
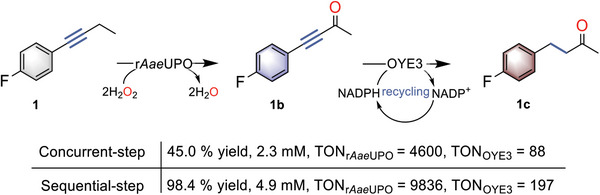
r*Aae*UPO‐catalyzed oxidation of 1 combined with OYE3‐catalyzed reduction for remote C─H bond oxyfunctionalization. Reaction conditions: concurrent steps: [1] = 5 mM, [r*Aae*UPO] = 500 nM, [H_2_O_2_] = 3 mM h^−1^, 30% (v/v) MeCN, [OYE3] = 25 µM, [d‐glucose] = 50 mM, [NADP^+^] = 2 mM, [GDH] = 1 mg mL^−1^, NaPi buffer (100 mM, pH = 7), 30 °C, 800 rpm, 1 mL, 24 h. Sequential steps: step 1: [1] = 5 mM, [r*Aae*UPO] = 500 nM, [H_2_O_2_]_final_ = 3 mM h^−1^, 30% (v/v) MeCN, NaPi buffer (100 mM, pH = 7), 30 °C, 5 h, 800 rpm; step 2: [OYE3] = 25 µM, [d‐glucose] = 50 mM, [NADP^+^] = 2 mM, [GDH] = 1 mg mL^−1^, 1 mL, 24 h.

Motivated by the wide substrate scope of peroxygenase, we next moved to the reduction of the C─C triple bond to achieve the overall divergent C─H bond oxyfunctionalization of aromatic hydrocarbons. We chose OYE3 from *S. cerevisiae*, which exhibited unique activity toward the C─C triple bond conjugated with a carbonyl group.^[^ ^29^ ^]^ A concurrent reaction combining r*Aae*UPO‐catalyzed oxidation and OYE3‐catalyzed reduction was attempted first (**Scheme** **2**). To sustain the reduction reaction of OYE3, a regeneration system of NADP^+^ to NADPH using glucose dehydrogenase (GDH) was used. Such a reaction afforded the corresponding γ‐keto arene in 45% yield in 24 h (Figure S31, Supporting Information). Further optimization, however, did not improve the yield. A detailed study showed that the performance of the two enzymes was not influenced by other reagents in its single transformation (Tables S3,S4). Although it was also surprising to see that H_2_O_2_ did not impair the performance of OYE3, the possible reason for the inhibition of the enzymes in a concurrent cascade reaction will be investigated further. As a compromise, we then chose a sequential manner in one pot, i.e., after the oxidation step of r*Aae*UPO, the OYE3 was added to initiate the reduction. Pleasantly, the conversion of alkyne substrate 1 into 1c was completed even without the removal of the remaining H_2_O_2_ by adding catalase after the oxidation step (Scheme 2). It should be noted that a stepwise reduction of 1b to 1c via an enone intermediate was observed (Figure S32, Supporting Information), in agreement with the report by Müller et al.^[^ ^29^ ^]^ The highest TON of 197 and 9836 was achieved for OYE3 and r*Aae*UPO, respectively.

Nevertheless, the sequential reaction approach was then applied to the substrate scope for the overall divergent C─H bond oxyfuntionalization (Figure 2, right column). Surprisingly, OYE3 could accept various ynones with substituted patterns and convert them into the desired products bearing a carbonyl group distant from the benzylic site (Figures S33–S51, Supporting Information). A good to satisfactory yield was thus achieved for γ‐keto arenes synthesis (1–17c). In some cases, the remaining enone intermediate was observed as a side product during the reduction process (7c, 10c, 12c, 13c,14c, and 17c). After the demethylation reaction, the ynal compound was converted to the corresponding aldehyde (18c), albeit in a higher formation of enal product. It is also possible to achieve the synthesis of two natural products, 7c and 11c, throughout the entire reaction sequence. Both enzymes, however, showed low activity to yield product 11c. In addition, two scaled‐up reactions in 1 mmol gave 7c and 15c in 61% (109 mg) and 20% (42 mg) isolated yield (Figure S52, Supporting Information).

**Scheme 3:**
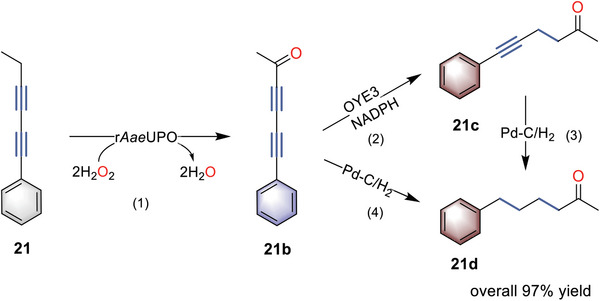
r*Aae*UPO‐catalyzed oxidation of 21 combined with OYE3 or Pd─C/H_2_ for remote C─H bond oxyfunctionalization. Reaction conditions: step 1, [21] = 5 mM, [r*Aae*UPO] = 2 µM, [H_2_O_2_] = 3 mM h^−1^, 30% (v/v) MeCN, NaPi buffer (100 mM, pH = 7), 30 °C, 800 rpm, 48 h. Step 2, [OYE3] = 50 µM, [d‐glucose] = 50 mM, [NADP^+^] = 2 mM, [GDH] = 1 mg mL^−1^, 30 °C, 800 rpm, 24 h. Step 3: 5 mol % Pd─C catalyst, H_2_ balloon, 20 h. Step 4: 5 mol % Pd─C catalyst, H_2_ balloon, 20 h.

The alkynes with two C─C triple bonds also drew our interest, and we asked if both enzymes could accept them and thus give ε‐keto arenes product (**Scheme** **3**). Strikingly, after r*Aae*UPO‐catalyzed complete oxidation, OYE3 could further reduce the C─C triple bond adjacent to the carbonyl group to give 21c in 99% yield and 100% selectivity without any enone formation (Figures S53–S56, Supporting Information). To fully desaturate the remaining triple bond of 21c and 21b, it was also possible to combine the well‐documented highly active Pd─C/H_2_ reduction, which eventually led to the desired product (21d) with a carbonyl group far away from the benzyl site in 97% conversion. By comparison, a reaction using hexyne as a substrate was only oxidized to trace amounts of alkynol product by the peroxygenase (Figures S57 and S58, Supporting Information). This result highlights the significance of an additional C─C triple bond in enabling the regioselectivity and enzyme activity in the synthesis of ε‐keto arenes, compared to alkylbenzenes with long‐chain substituents.

**Figure 3:**
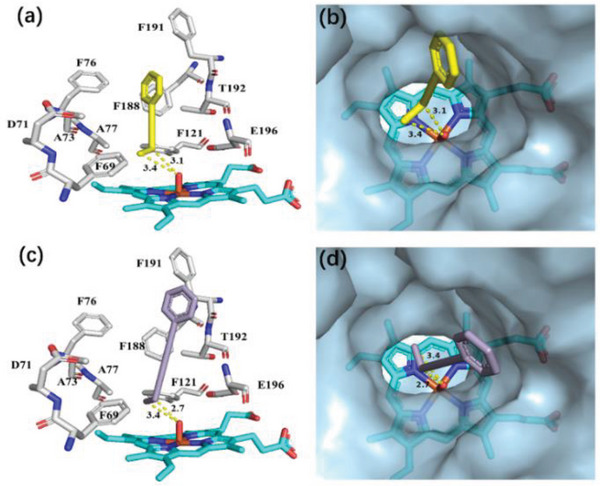
Active site model of PaDa‐I in complex with compounds a,b) 9 and c, d) 21. The substrates and heme center are shown in stick representation. Important active sites and catalytic residues are shown in lines. The dashed lines indicate distances (in Å) from the surrounding residues.

To shed light on the interplay of the C─C triple bond on r*Aae*UPO activity and selectivity, molecular modeling was again performed (**Figure** **3**). The presence of a C─C triple bond to the benzylic position of butylbenzene effectively reduced the flexibility of the substrate (9), thus increasing the regioselectivity for positioning the propargylic carbon compared to butylbenzene (Figure 3a,b). A similar configuration of the dialkyne (21) was found, in which the propargylic C─H showed a shorter distance to the oxygen atom of the heme center (Figure 3c,d). The alkyne substrates are well suited in the conical funnel‐shaped channel of r*Aae*UPO toward the active site, and even the presence of two C─C triple bonds did not noticeably influence its orientation and binding. The docking study is in good agreement with the results observed in Scheme 3. Therefore, it is rational to hypothesize that the position of the remote carbonyl group can be tuned by engineering the number and distance of the C─C triple bond in an alkane substrate.

**Figure 4:**
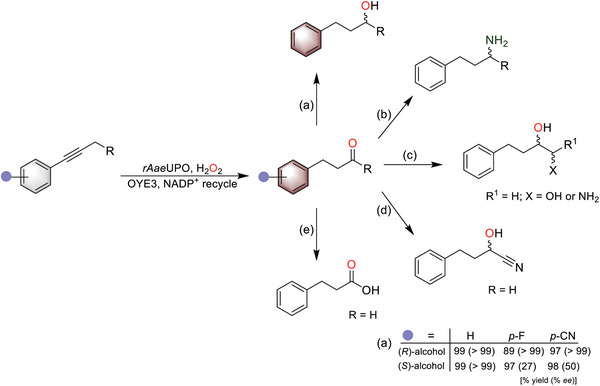
The cascade reaction can be combined with known bio‐catalysts to establish new synthetic pathways to yield various functionalized products. a) Combination with *Tb*ADH and *Lk*ADH for chiral alcohol synthesis. Reaction conditions: [substrate] = 5 mM, [r*Aae*UPO] = 500 nM, [H_2_O_2_] = 3 mM h^−1^, 30% (v/v) MeCN, NaPi buffer (100 mM, pH = 7), 800 rpm, 30 °C, 1 mL, 5 h. After the reaction, [OYE3] = 25 µM, [Glu] = 50 mM, [GDH] = 1 mg mL^−1^, and [NADP^+^] = 2 mM were added to the above reaction mixture and reacted for 24 h. Then, [*Lk*ADH] = 50 µM or [*Tb*ADH] = 25 µM, 50 µL isopropanol, [lysozyme] = 1 mg mL^−1^, and [Dnase I] = 6 U mL^−1^ were added to the reaction for 5 h. Other proposed reactions include cascades with b) amine transaminases or imine reductases for biocatalytic variants of the Mitsunobu reaction to yield enantiopure amines, c) aldolase for C─C bond formation, d) hydroxynitrile lyases for asymmetric synthesis of cyanohydrins and e) aldehyde dehydrogenases to convert aldehydes to acids.

The envisioned enzymatic approach has the potential to expand biochemical synthesis by enabling new biosynthetic routes. Therefore, in a final test of the generality of our C─H functionalization approach, we explored the obtained γ‐keto arenes as substrates for further biosynthetic transformations (**Figure** **4**). Combining two alcohol dehydrogenases (ADHs), (*S*)‐selective ADH from *Thermoanaerobacter brokii* (*Tb*ADH)^[^ ^30^ ^]^ and (*R*)‐selective ADH from *Lactobacillus kefir* (*Lk*ADH),^[^ ^31^ ^]^ alcohol products with complementary chirality were readily obtained in one‐pot (Figure 4a and Figures S59–S61, Supporting Information). In this sequential reaction, the yield of the three substrates was between 89 and 99%, with excellent stereoselectivity for *Lk*ADH (> 99% *ee*) and varied stereoselectivity for *Tb*ADH (27–99% *ee*). Although not investigated experimentally herein, we propose that the design of *de novo* multienzyme cascades or synthetic metabolic pathways could yield a variety of remotely functionalized asymmetric products, as shown in Figure 4b–e.

## 3 Conclusion

In summary, we have developed a synthetic approach for the divergent sp^3^ C─H bond oxyfunctionalization of hydrocarbons to synthesize the valuable γ‐ and ε‐keto arenes. The peroxygenase demonstrated excellent regioselectivity and activity in activating the propargylic C─H bond, leading to ynone and ynal products. These products were then precisely reduced by the Old Yellow Enzyme, resulting in a broad range of structurally diverse and oxyfunctionalized products with carbonyl groups distant from the benzylic site. While there are currently no chemical methods to directly introduce a C─C triple bond to a saturated C─C bond of alkanes, the substrates utilized in this study can be prepared through straightforward coupling reactions. This thus enables the broadening of the substrate scope for both the peroxygenase and Old Yellow Enzyme.

Biocatalytic divergent C─H bond oxyfunctionalization remains a significant challenge in the synthetic community. Though protein engineering has been attempted, the present enzymatic cascade offers a practical and efficient method with a broad substrate scope. These results expand the knowledge of the non‐natural reactions and substrate specificity of both peroxygenases and Old Yellow Enzymes. Last but not least, this approach can be combined with well‐established biocatalysts to catalyze various asymmetric divergent C─H functionalization reactions in shorter enzymatic pathways.

## 4 Experimental Section

### Materials

All chemicals, unless otherwise stated, were purchased from commercial sources (Sigma‒Aldrich, Bide Pharmatech Ltd., Macklin, and Energy Chemical) and used without prior purification through the experiments. All substrates and intermediate compounds used for enzymatic reactions were obtained by either commercial purchase or synthesis by ourselves. Spectroscopic characterization of the in‐house synthesized compounds is included in Figures S59–S140 and Table S5 (Supporting Information).

### Enzyme Preparation

The unspecific peroxygenase (PaDa‐I variant) from *A. aegerita* in *P. pastoris* was prepared and purified according to a previous method.^[^ ^26^ ^]^ The Old Yellow Enzyme 3 from *S. cerevisiae*,^[^ ^29^ ^]^ (*S*)‐selective ADH from *Thermoanaerobacter brokii* ^[^ ^30^ ^]^ and (*R*)‐selective ADH from *Lactobacillus kefir* (*Lk*ADH)^[^ ^31^ ^]^ were prepared via adapted procedures. A detailed description of the procedures was given in the Supporting Information.

### Enzymatic Reactions

Oxyfunctionalization of propargylic C─H bonds using r*Aae*UPO. To a 2 mL glass bottle, 5 mM alkyne substrate and 500 nM r*Aae*UPO were added to sodium phosphate buffer (100 mM, pH 7.0) with 30% acetonitrile. The reaction volume was adjusted to 1 mL using the same buffer. Then, the oxygenase source H_2_O_2_ from a stock solution was dosed to the reaction mixture by a syringe pump at a rate of 5 µL h^−1^, corresponding to a concentration of 3 mM h^−1^. The reaction vial was sealed and stirred in a thermal shaker at 800 rpm and 30 °C. At intervals, 100 µL of the reaction mixture was withdrawn and extracted with 200 µL of ethyl acetate. After vortex mixing, the organic phase was collected by centrifugation, dried over anhydrous Na_2_SO_4,_ and subjected to GC analysis. The temperature file of the GC methods was included in Table S6 (Supporting Information).

### Reduction of the C─C triple Bond of Ynones

When the oxidation step using r*Aae*UPO was completed, 25 – 50 µM OYE3, 50 mM GDH, 1 mg mL^−1^ glucose and 2 mM NADP^+^ were added. The reaction mixture was allowed to react for 24 h. To analyze the product concentration and yield, the sampling procedures described above were used.

### Cascade Reaction with Alcohol Dehydrogenases

After the reduction of ynones was completed, 50 µM *Lk*ADH or 25 µM *Tb*ADH, 50 µL isopropanol, 1 mg mL^−1^ lysozyme, and 6 U mL^−1^ DNase I were added for the asymmetric reduction of ketones for another 5 h. Similar sampling procedures were used as described. The product concentration and yield and the enantiomeric excess were determined by chiral GC.

### Preparative‐Scale Synthesis

The reactions were carried out on a 1 mmol scale with similar procedures described in the oxidation reaction by r*Aae*UPO or cascade reactions between r*Aae*UPO and OYE3. Upon completion of the reaction, the reaction mixture was extracted with ethyl acetate (three times). The organic phase was combined and dried over anhydrous Na_2_SO_4_ and evaporated under reduced pressure. The obtained yellowish oil was purified by flash chromatography (5% ethyl acetate in petroleum ether).

### Molecular Docking

The structure of r*Aae*UPO (PDB ID: 5OXU) was used in the molecular modeling.^[^ ^32^ ^]^ The parameter setting of AutoGrid 4.2.5 was as follows: Grid points in *xyz* were 36 × 40 × 34 with a grid center at 11.955 × 3.904 × 10.377, and the grid‐point spacing was 0.303 Å. AutoDock 4.2 software was used for the docking simulations by running ≈100 docking trials. The modeling structures were visualized by using the PyMOL Molecular Graphics System.^[^ ^33^ ^]^

### Statistical Analysis

The experimental statistical data were analyzed from at least two samples and expressed as mean ± SD.

## Conflict of Interest

The authors declare no conflict of interest.

## Author Contributions

H.L., Y.Z., Y.H., R.G., and X.H. performed the experimental work, docking study, and analyzed the results; P.D. participated in the planning and analysis of the experiments; W.Z. conceived and designed the experiments and wrote the manuscript. All authors participated in the writing of the manuscript.

## Supporting information

Supporting Information

## Data Availability

The data that support the findings of this study are available in the supplementary material of this article.
