# Supplementary material for: A Simple Access to γ‐ and ε‐Keto Arenes via Enzymatic Divergent C─H Bond Oxyfunctionalization
Source: Adv Sci (Weinh). 2023 Oct 23;10(34):2304605. doi: 10.1002/advs.202304605 (PMC10700168; doi:10.1002/advs.202304605)
Supplement: Supplementary file 1 — Supporting Information [file ADVS-10-2304605-s001.pdf]

ADVANCED SCIENCE
Open Access

Supporting Information

for *Adv. Sci.*, DOI 10.1002/advs.202304605

A Simple Access to γ- and ϵ-Keto Arenes via Enzymatic Divergent C—H Bond Oxyfunctionalization

Huanhuan Li, Yalan Zhang, Yawen Huang, Peigao Duan, Ran Ge, Xiaofeng Han and Wuyuan Zhang**

Supplementary information

A Simple Access to γ- and ε-Keto Arenes via Enzymatic Divergent C-H bond Oxyfunctionalization

Huanhuan Li, Yalan Zhang, Yawen Huang, Peigao Duan, Ran Ge, Xiaofeng Han, Wuyuan Zhang

Table of contents

Table of contents 2
Substrate synthesis 3
Synthesis of ynone standards 5
Synthesis of alkyl ketones 7
Enzymatic reactions 8
Semipreparative scale synthesis 8
Enzyme preparation 10
Analytics 10
NMR spectrum 49
Details of the gas chromatograph and temperature profiles 96
References 101

Substrate synthesis

Terminal alkyne substrates were purchased from Leyan.com. Other chemicals were purchased from commercial sources (Sigma-Aldrich, Alfa Aesar, TCI chemicals, etc.) and used directly in all experiments. All substrates and intermediate compounds used for enzymatic reactions were obtained by either commercial purchase or synthesis in-house. For the latter, spectroscopic characterization of the synthesized compounds was included in the Supplementary Information. The procedures of the synthesis of noncommercially available substrates were shown as follows.

Synthesis of substrates **2-4, 6-8,10** and **11**

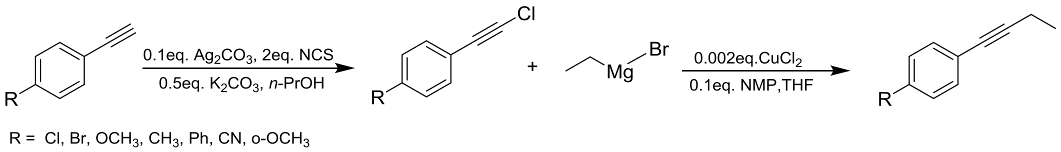

The procedures were adapted from reported protocols[1,2]:

Step 1: K_2CO_3 (0.5 mmol), NCS (2 mmol) and Ag_2CO_3 (0.1 mmol) were added to a two-necked round-bottom flask. The flask was purged with N_2, and terminal alkyne (1 mmol) and *n*-propanol (20 mL) were subsequently added via a syringe. The reaction mixture was stirred at 50 °C for 12 h. The reaction progress was monitored by thin-layer chromatography (TLC). After the reaction, the mixture was allowed to cool to room temperature, and brine was added at 0 °C in an ice bath. The crude mixture was filtered, and the solid residue was washed with ethyl acetate. The combined organic phase was concentrated under reduced pressure and purified by column chromatography with 100% PE on silica gel to obtain the corresponding 1-chloroalkynes.

Step 2: To a dried two-necked round-bottom flask was added 1-chloroalkynes (1 mmol), NMP (0.1 mmol) and $CuCl_2$ (0.002 mmol) in dry THF (10 mL) at 0 °C. The flask was purged with N_2, and ethylmagnesium bromide 3.0 M solution in Et_2O (1.5 mmol) was added via a syringe. The reaction progress was monitored by TLC. After approximately 3 hours, 1 M HCl solution was added to quench the reaction. The crude mixture was filtered, and the solid residue was washed with ethyl acetate and dried by Na_2SO_4. The combined organic phase was concentrated under reduced pressure and purified by flash column chromatography by 100% PE.

Synthesis of substrates **12-17**

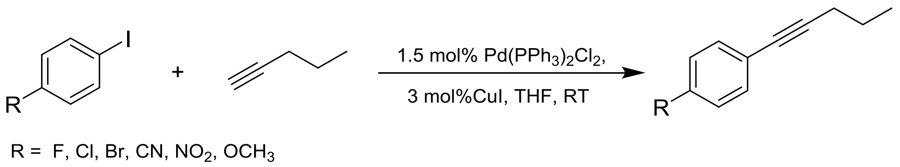

The procedures were adapted from the literature[3]: To a dried two-necked round-bottom flask, the corresponding substituents iodobenzene (5 mmol), $Pd(PPh_3)_2Cl_2$ (0.08 mmol), CuI (0.15 mmol), triethylamine (12 mmol) and THF (20 mL) were added. The flask was purged with N_2, and alkynes (5.5 mmol) were added via syringe. The reaction mixture was stirred overnight at room temperature. After the reaction, the mixture was filtered, dried, concentrated under reduced pressure and purified by flash column chromatography with 100% PE.

Synthesis of substrate **18**

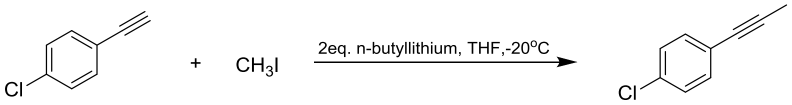

The procedures were adapted from the literature[4]: To a dried two-neck round-bottom flask, phenylacetylene (5 mmol) and THF (30 mL) were added under N_2 protection. The flask was placed in an ice water bath for 30 min. n-Butyllithium (10 mmol, 2 eq.) was added dropwise, and the reaction was allowed to stir for 1 h. Then, CH_3I (2.1 eq.) was added at -20 °C to react for 1 h. The reaction was then stirred at room temperature for 12 h. The reaction progress was monitored by TLC. Upon completion, the reaction was quenched with a saturated solution of ammonium chloride and extracted with ethyl acetate. Then, the crude mixture was filtered, dried and concentrated to yield a crude mixture. Finally, the residue was purified by column chromatography (100% PE).

Synthesis of substrate **20**

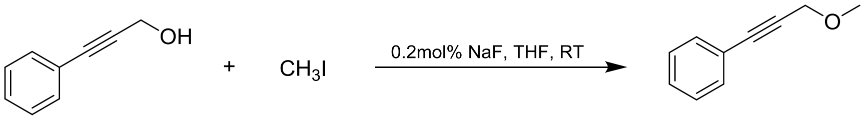

The procedures were adapted from the literature[5]: 3-phenyl-2-propyn-1-ol (5 mmol), NaH (10 nmol), CH_3I (10 mmol) and 20 mL THF were added to a dried two-necked round-bottom flask. The reaction mixture was stirred at room temperature for 10 h. The reaction progress was monitored by TLC. Upon completion, the crude mixture was filtered, and the solid residue was washed with ethyl acetate and purified by flash column chromatography by PE/EA (v/v, 95%/5%).

Synthesis of substrate **22**

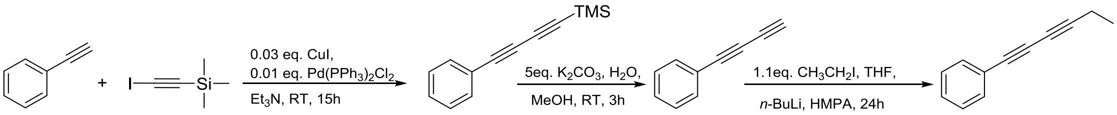

The procedures were adapted from the literature[6,7]. **Step 1**: to a dried two-neck round-bottom flask, phenylacetylene (3.5 mmol), 1-iodo-2-(trimethylsilyl)acetylene (2.5 mmol), CuI (0.12 mmol), $Pd(PPh_3)_2Cl_2$ (0.35 mmol) and Et_3N (10 mL) were added under N_2 protection. The reaction was stirred for about 15 hours at room temperature. The mixture was diluted with ethyl acetate and filtered. Then mixture was dried, evaporated and subjected to silica gel column chromatography with 100% EA to yield the corresponding trimethyl(phenylbuta-1,3-diyn-1-yl)silane. **Step 2**: To a round -bottom flask, the trimethyl(phenylbuta-1,3-diyn-1-yl)silane (2 mmol), K_2CO_3 (10 mmol) and 10 mL of H_2O_2/MeOH (1:1). Then the reaction mixture was stirred at room temperature for 3 h. the mixture was filtrated and extracted with ethyl acetate, and the organic phase was dried, evaporated to yield the corresponding the buta-1,3-diyn-1-ylbenzene. **Step 3**: to a dried three-neck round-bottom flask, buta-1,3-diyn-1-ylbenzene (1 mmol), 20 mL dry THF was added with N_2 protect. The reaction was stirred at - 78°C, subsequently, the *n*-butyllithium (1.2 eq.) and HMPA (1.2 eq.) was added to reactant for another 2 hours. Then iodoethane (2 eq.) was added to the solution after the reaction reached room temperature. And the reactant was refluxed for about 24h. The reaction was quenched with saturated aqueous NH_4Cl solution and extracted with ethyl acetate. Then, the crude mixture was filtered, dried and concentrated to yield a crude mixture. Finally, the residue was purified by column chromatography (100% PE).

Synthesis of ynone standards

Synthesis of **1-8b** and **10-11b**

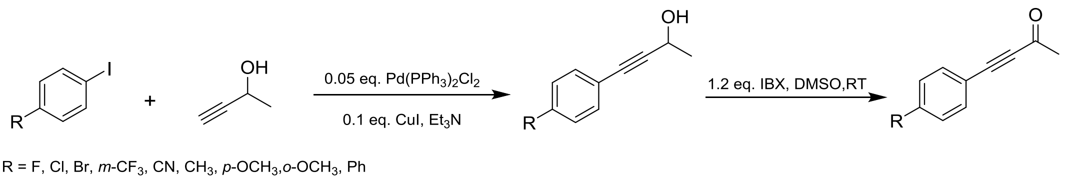

The procedures were adapted from reported protocols [8,9,10,11]:

Step 1: $Pd(PPh_3)_2Cl_2$ (0.05 eq.), CuI (0.1 eq.) and substituted aryl iodide (1 eq.). The flask was purged with N_2 and subsequently Et_3N and 3-butyne-2-ol (1.2 eq.) were added via a syringe under N_2 protection. The reaction mixture was stirred at room temperature. The reaction progress was monitored by TLC. Upon completion, the crude mixture was washed with water (3 times). Then, the reaction residue was extracted with ethyl acetate. The combined organic phase was concentrated under reduced pressure. The desired aryl propargyl alcohols were purified via flash column chromatography. **Step 2**: When aryl propargyl alcohols were finished, the corresponding ynones were obtained by further oxidation. The IBX (1.2 eq.) and DMSO (5 mL) were added to a dry round-bottom flask. Then, the reaction was stirred for a few minutes until the solution became transparent. Subsequently, aryl propargyl alcohol (1 eq.) was added dropwise into the above solution and reacted at room temperature. The reaction progress was monitored by TLC. Upon completion, the crude mixture was quenched with water. Then, the reaction residue was extracted with ethyl acetate. The combined organic phase was

concentrated under reduced pressure and purified via flash column chromatography to yield the desired ynone product.

Synthesis of **12-14b** and **16-17b**

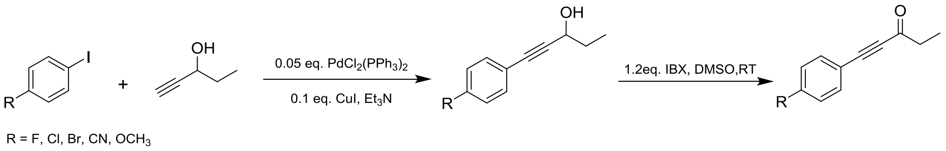

The synthesis of compounds **12-14b** and **16-17b** was the same as that described above.

Synthesis of **15b**

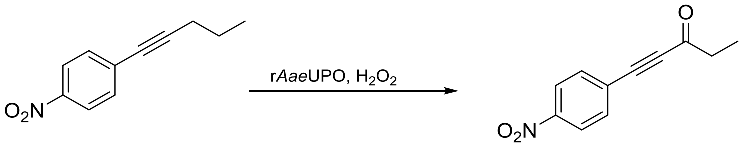

15b is a semipreparative synthesis by r*Aae*UPO-catalyzed oxidation of **15**. The specific reaction steps were as follows: to a 50 mL glass bottle, 1-nitro-4-(pent-1-yn-1-yl) benzene (**15**, 20 mM) and r*Aae*UPO (20 µM) were added to 20 mL sodium phosphate buffer (100 mM NaPi, pH 7.0) with 30%MeCN. Then, the H_2O_2 from a stock solution (600 mM) was dosed by a syringe pump at a rate of 100 µL h^{-1}. The reaction vial was sealed and stirred in a water bath at 200 rpm at 30 °C. Upon completion of the reaction, the reaction mixture was extracted with ethyl acetate (three times). The organic phase was combined and dried over anhydrous Na_2SO_4 and evaporated under reduced pressure. The products were not further purified and were directly used for NMR analysis.

Synthesis of **22b**

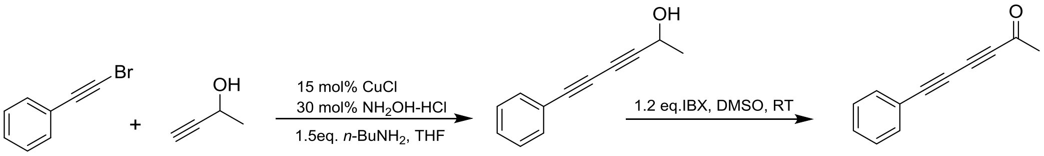

The procedures were adapted from reported protocols [12]:

Step 1: To a 100 mL round-bottom flask was added CuCl powder (0.75 mmol) dissolved in a 2:3 mixture of *n*-$BuNH_2/H_2O$ (2.5 mL) at 0 °C. A few crystals of NH_2OH•HCl was added to discharge the blue color. 3-Butyn-2-ol (5 mmol) was added to the solution, and the mixture was stirred for 5 min. (Bromoethynyl)benzene (5.5 mmol) was then added to the mixture. More crystals of NH_2OH•HCl was added to the reaction mixture to prevent the solution from turning blue. The reaction progress was monitored by TLC. Upon completion, the crude mixture was washed with saturated NH_4Cl and extracted with ethyl acetate. The combined organic phase was concentrated under reduced pressure and

purified via flash column chromatography (PE: EA = 9: 1). **Step 2**: The resulting alcohol was subjected to further oxidation according to the general procedures. IBX (1.2 eq.) and DMSO (5 mL) were added to a flame-dried round-bottom flask. Then, the reaction was stirred for a few minutes until the solution became transparently clear. Subsequently, aryl alcohol (1 eq.) was added dropwise into the above solution and reacted at room temperature. The reaction progress was monitored by TLC. Upon completion, the crude mixture was quenched with water. Then, the reaction residue was extracted with ethyl acetate. The combined organic phase was concentrated under reduced pressure and purified via flash column chromatography to yield 6-phenylhexa-3,5-diyn-2-one PE/EA (v/v, 90%/10%).

Synthesis of alkyl ketones

Synthesis of benzylacetone analogues **5c-10c**, and **17c**

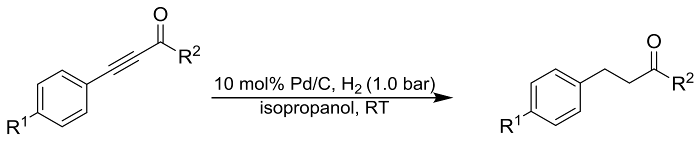

R^1 = CF_3, CH_3, *p*-OCH_3,*o*-OCH_3, Ph; R^2 = CH_3
R^1 = OCH_3; R^2 = CH_2CH_3

The procedures were adapted from reported protocols**:** the alkyne ketone, Pd/C catalyst (10 mol%), and 10 mL of isopropyl alcohol (IPA) were added to a 50 mL round-bottom flask. The round-bottom flask was exchanged with H_2 three times. Then, the reaction mixture was reacted overnight with 1 bar H_2. After the reaction, the reaction mixture was filtered. The Pd/C catalyst was washed with dichloromethane three times. The reaction resolution was concentrated under reduced pressure and purified via flash column chromatography to give the final ketone product.

Synthesis of **22c**

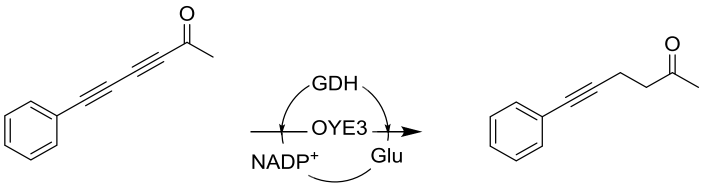

The procedure was adopted by using OYE3-catalyzed reduction[13]**:** 6-phenylhexa-3,5-diyn-2-one (15 mM), OYE3 (500 µM), $NADP^+$ (2 mM), Glu (50 mM), and 1 mg mL^{-1} dry glucose dehydrogenase were added to a 10 mL NaPi solution (100 mM, pH 7) with 30% MeCN. The reaction mixture was reacted in a thermal shaker at 30 °C and 800 rpm for 30 h. After the reaction, the mixture was extracted with ethyl acetate. The combined organic phase was dried and concentrated under reduced pressure, and the target product (6-phenylhex-5-yn-2-one) was obtained. The product was not further purified and directly used for NMR analysis.

Enzymatic reactions

Oxyfunctionalization of propargylic C-H bonds using r*Aae*UPO.

To a 2 mL glass bottle, 5 mM alkyne substrate and 500 nM *rAae*UPO were added to sodium phosphate buffer (100 mM NaPi, pH 7.0) with 30% MeCN. The reaction volume was adjusted to 1 mL using the same buffer. Then, the H_2O_2 from a stock solution was dosed by a syringe pump at a rate of 5 µL h^{-1}, corresponding to a concentration of 3 mM h^{-1}. The reaction vial was sealed and reacted in a thermal shaker at 800 rpm and 30 °C. After the reaction, 100 µL of the reaction mixture was withdrawn and extracted with 200 µL of ethyl acetate containing 5 mM dodecane as an internal standard. The organic phase was dried over anhydrous Na_2SO_4 and subjected to GC.

Reduction of the C-C triple bond of ynones.

After the oxyfunctionalization step, 25-50 µM OYE3, 50 mM GDH, 1 mg mL^{-1} glucose and 2 mM $NADP^+$ were added to the reaction mixture. The reaction mixture was allowed to continue for 24 h. The yield analysis of the ynone products and the sampling procedures were the same as described above.

Cascade reaction with alcohol dehydrogenases.

After the reduction of ynones was completed, 50 µM *Lk*ADH or 25 µM *Tb*ADH, 50 µL isopropanol, 1 mg mL^{-1} lysozyme, and 6 U mL^{-1} DNase I were added for the asymmetric reduction of ketones, and the reaction mixture was reacted for another 5 h. Similar sampling procedures were used as described. The product concentration and yield were determined by GC. The enantiomeric excess was determined by GC equipment with a chiral column (column B).

Statistical Analysis: the experimental statistical data were analyzed from at least two samples and expressed as mean ± SD.

Semipreparative scale synthesis

Oxyfunctionalization of propargylic C-H bonds using r*Aae*UPO.

The reactions were carried out on a 1 mmol scale. To a 100 mL glass bottle, alkyne substrates (**13, 16**) and r*Aae*UPO (125 µM) (added to the reaction twice) were added to 50 mL of sodium phosphate buffer (100 mM, pH 7.0) with 30% MeCN. Then, the H_2O_2 from a stock solution (600 mM) was dosed by a syringe pump at a rate of 250 µL h^{-1}. Then, the reaction was sealed and stirred in a water bath at 200 rpm and 30 °C. Upon completion of the reaction, the reaction mixture was extracted with ethyl acetate (three times). The organic phase was combined and dried over anhydrous Na_2SO_4 and evaporated under reduced pressure. The reaction achieved complete conversion by GC. The isolation yield of 60% 12b (115mg) and 74% 15b (135mg) was obtained, respectively.

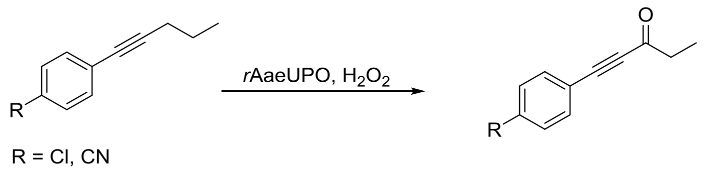

Synthesis of ynones by cascade between r*Aae*UPO and OYE3

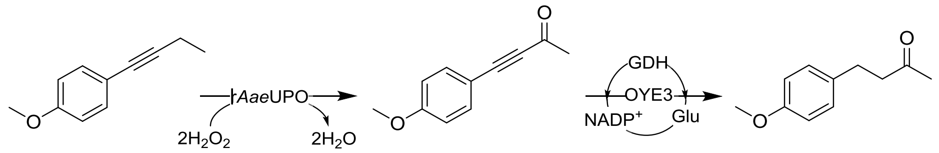

The reactions were carried out on a 1 mmol scale. To a 200 mL glass bottle, alkyne substrates (**7**) and r*Aae*UPO (600 µM, added to the system in batches) were added to 100 mL of sodium phosphate buffer (100 mM NaPi, pH 7.0) with 30% MeCN. Then, the H_2O_2 from a stock solution (600 mM) was dosed by a syringe pump at a rate of 250 µL h^{-1}. Then, the reaction was stirred in a water bath at 200 rpm and 30 °C. The reaction process was monitored by GC, and the reduction steps were performed after 100 h of reaction. The reaction mixture was not further processed, and 5 mM OYE3 (added to the system in batches), 50 mM GDH, 1 mg mL^{-1} glucose and 2 mM $NADP^+$ were added. The recycling system was added every 12 hours. The reaction process was monitored by GC (the reaction was performed for approximately 60 h). Upon completion of the reaction, the reaction mixture was extracted with ethyl acetate (three times). The organic phase was combined and dried over anhydrous Na_2SO_4 and evaporated under reduced pressure. The mixture was purified via flash column chromatography (PE/EA, v/v, 95%/5%). The corresponding yield was 61% (109mg).

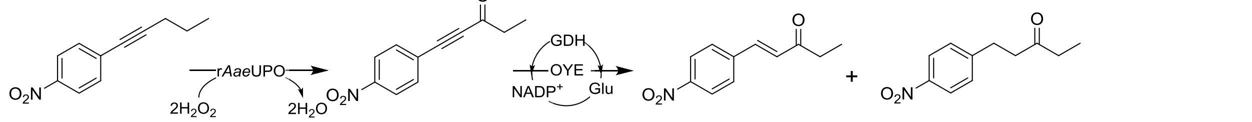

The reactions were carried out on a 1 mmol scale. To a 200 mL glass bottle, alkyne substrates (**16**) and r*Aae*UPO (100 µM) were added to 100 mL sodium phosphate buffer (100 mM NaPi, pH 7.0) with 30% MeCN. Then, the H_2O_2 from a stock solution (600 mM) was dosed by a syringe pump at a rate of 250 µL h^{-1}. Then, the reaction was stirred in a water bath at 200 rpm and 30 °C. The reaction process was monitored by GC. After oxidation, 5 mM OYE3, 50 mM GDH, 1 mg mL^{-1} glucose and 2 mM $NADP^+$ were added. The recycling system was added every 12 hours. The reaction process was monitored by GC (the reaction was performed for approximately 60 h). After reaction, the reaction mixture was extracted with ethyl acetate (three times). The organic phase was combined and dried over anhydrous Na_2SO_4 and evaporated under reduced pressure. The mixture was purified via flash column chromatography (PE/EA, v/v, 95%/5%). The corresponding yield was 20% 1-(4-nitrophenyl)pentan-3-one (42 mg).

Enzyme preparation

Preparation of peroxygenase.

The expression and preparation of the unspecific peroxygenase from *A. aegerita* (PaDa-I variant) in P. pastoris were adopted according to the previous methods [14,15].

Preparation of Old Yellow Enzyme

The Old Yellow Enzyme 3 from *S. cerevisiae* (P41816, *E. coli* Lu13669) was produced according to reported procedures [13]. The genes were incorporated into pET-28a (+) and expressed in *E. coli* BL21(DE3) cells at 37 °C overnight. Subsequently, 1% (v/v) of the seed culture was cultivated in 200 mL of LB medium at 37 °C and 220 rpm for 3 h. Induction of protein expression was initiated by the addition of 0.3 mM isopropyl *β*-D-1-thiogalactopyranoside (IPTG) when the OD_{600} reached 0.6-0.8. The cultivation continued overnight at 16 °C and 220 rpm. The cells were harvested by centrifugation at 4 °C and 10000 rpm for 30 min, washed and resuspended in PBK buffer (100 mM, pH 7.4), and stored at -20 °C until further use. Twelve percent SDS–PAGE was run with the crude enzymes after suspending and disrupting the cells in KPB buffer.

Preparation of alcohol dehydrogenases

Genes for alcohol dehydrogenases (ADHs) from *Lactobacillus kefiri* DSM 20587 (GenBank: AY267012.1)[16] and *Thermoanaerobacter brockii* (GenBank: WP_014063649.1, *Tb*ADH)[17] were incorporated into pET24a between the restriction sites of *Nde* I and *Xho* I and expressed in *E. coli* BL21(DE3) cells. The cells were cultivated in 5 mL of LB medium containing 50 μg mL^{-1} kanamycin at 37 °C and 220 rpm for 6-8 h. Subsequently, 1% (v/v) of the seed culture was transferred into TB medium containing 50 μg mL^{-1} kanamycin at 37 °C and 220 rpm. Induction of protein expression started with the addition of 0.1 mM isopropyl *β*-D-1-thiogalactopyranoside (IPTG) when the OD_{600} reached 0.6-0.8. Then, 0.1 mM zinc ions were added to the culture. The cultivation continued for 16 h at 20 °C and 220 rpm. The cells were harvested by centrifugation at 4 °C and 4000 rpm for 10 min, washed and resuspended in KPB buffer (100 mM, pH 7.4), and stored at -20 °C until further use. Twelve percent SDS–PAGE was run with the crude enzymes after suspending and disrupting the cells in KPB buffer.

Analytics

Gas chromatography methods

Gas chromatography equipped with a flame ionization detector (FID) was used to quantify the concentration of each compound and determine the yield of the reaction. Nitrogen was used as the

carrier gas, and a column flow at 3 mL min^{-1} and split ratio of 30:1 was used in all analyses. Suitable columns (A and B) were used depending on the compounds.

Column A: SH-Rtx-1 column (30 m length, 0.25 mm inner diameter, 1 μm film thickness).

Column B: Chiral CP7503 column (25 m length, 0.32 mm inner diameter, 0.25 μm film thickness).

^{1}H NMR spectroscopy analysis

^{1}H NMR spectra were recorded at 298.2 K on a Bruker AVANCE III 400 MHz NMR spectrometer (Bruker Bio spin, Germany) operating at 400 MHz for proton frequency and 101 MHz for carbon frequency. TMS was used as an internal standard, and $CDCl_3$ was used as the solvent.

GC–MS analysis

High-resolution GC-QTOF-MS analysis was carried out on a Thermo Scientific Orbitrap Exploris GC 240 Mass Spectrometer (Thermo Fisher, Germany) with a TraceGOLD TG-5SILMS 30 m column (30 m length, 0.25 mm inner diameter, 0.25 μm film thickness). A sample volume of 1 μL was injected into an S/SL injector. The GC was programmed from 70 °C held for 2 min; 10 °C min^{-1} to 325 °C held for 8.5 min. Eluting peaks were transferred through an auxiliary transfer temperature of 290 °C into a mass spectrometer. The injection, transfer line, and ion source temperatures were 250, 290, and 250 °C, respectively. The accurate mass spectrometry data were acquired in full-scan mode at 60,000 mass resolution (FWHM m/z 200) after a solvent delay of 6.2 min. Thermo Scientific Xcalibur Version 4.5.445.18 software with the NIST2020 library was used for raw peak exacting, peak identification and integration of the peak area. To generate the extracted ion chromatograms (EICs), a mass window of ± 5 ppm was used, meaning that only ions with a mass accuracy ≤5 ppm were extracted.

Computational methods

The structure of r*Aae*UPO (PDB ID: 5OXU) was used in the molecular docking[18]. Parameter Setting of AutoGrid 4.2.5: Grid points in xyz were 36×40×34 with a grid center at 11.955×3.904×10.377 and a grid-point spacing of 0.303 Å. AutoDock 4.2 was used for the docking simulations by running 100 docking trials with the Genetic Algorithm. Models were visualized with the PyMOL Molecular Graphics System. Specifically, the distance from the carbon atom of the ligands to the oxygen atom of compound I was plotted and measured by PyMOL. The binding energy of every conformation was calculated by AutoDock 4.2.

Table S1. Optimization of r*Aae*UPO-catalyzed C-H bond oxyfunctionalization of **1** to **1b**

**Entry**	**Cosolvent**	**[r*Aae*UPO] nM**	**[H_2O_2] mM h^{-1}**	**Concentration of 1b, mM**	**Initial reaction rate of 1b, mM h^{-1}**	**TON**	**GC Yield of 1b, %**
1	Acetone	500	3	4.77	0.95	9545	95.5
2	IPA	500	3	2.42	0.48	4843	48.4
3	DMSO	500	3	2.24	0.45	4477	44.8
4	MeCN	500	3	4.92	0.98	9836	98.4
5	MeCN	500	1	1.18	0.24	2360	23.6
6	MeCN	500	2	3.74	0.75	7480	74.8
7	MeCN	500	5	4.90	0.98	9793	97.9
8	MeCN	100	3	4.22	0.84	42170	84.3
8	MeCN	250	3	4.85	0.97	19514	97.0
9	MeCN	375	3	4.88	0.98	13010	97.6
10	MeCN	500	-	-	-	-	N.D.
11	MeCN	-	3	-	-	-	N.D.
12	MeCN	500[a]	3	-	-	-	N.D.

Reaction conditions: [**1**] = 5 mM, [*rAae*UPO] = 100-500 nM, [H_2O_2] = 1-5 mM h^{-1}, 30% (v/v) MeCN/acetone/IPA/DMSO, 70% NaPi buffer (100 mM, pH = 7), 30 °C, 800 rpm, 1 mL, 5 h. N.D. means not detected. The initial rate is based on concentration of 1b at 5 h. TON = Turnover number ([1b]/[r*Aae*UPO]). [a] r*AaeUPO* was boiled in 100 °C water for 30 min to inactivate. Values are based on duplicate experiments.

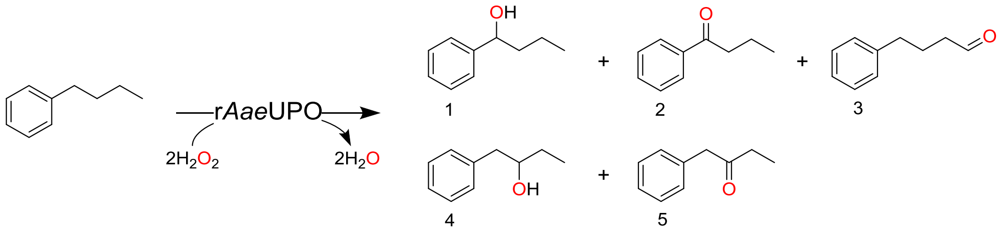

Table S2. The control reaction of the r*Aae*UPO-catalyzed oxidation of butylbenzene yields a mixture of five oxidized products.

Compound	1	2	3	4	5
**Retention time(min)**	14.27	14.40	14.11	13.85	13.62
**Yield[a] (%)**	1.24	1.48	72.40	22.0	0.65

Reaction conditions: [substrate] = 5 mM, NaPi buffer (100 mM), pH = 7, [r*Aae*UPO] = 500 nM, 30% MeCN, 30 °C, 800 rpm, 5 h, 1 mL. [a] The yield was determined based on GC–MS. [a] The yield was determined based on GC–MS.

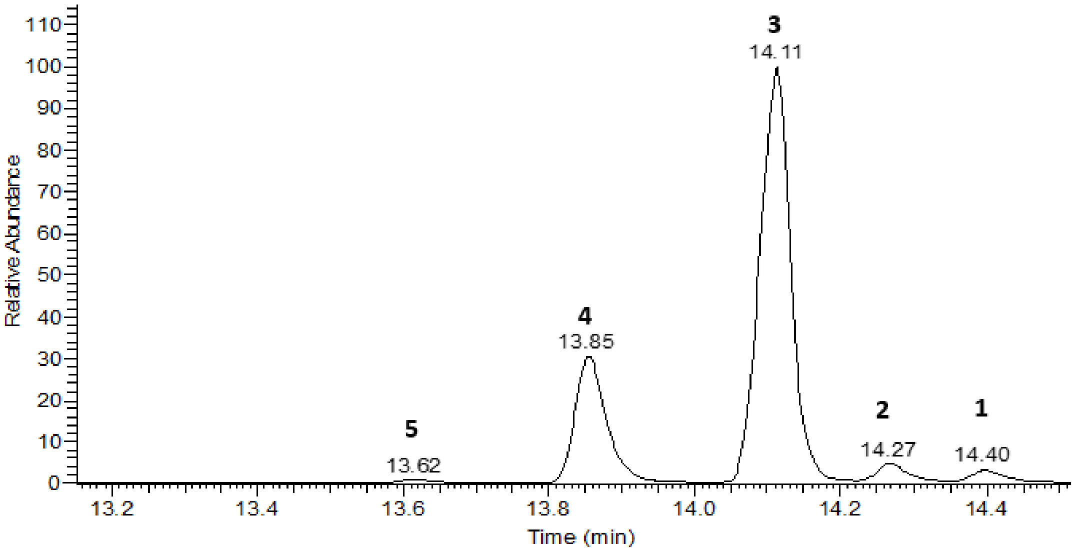

Figure S1. Representative GC–MS chromatogram of r*Aae*UPO-catalyzed oxidation of butylbenzene. Each peak was determined by the MS shown in Supplementary Figures S2-6.

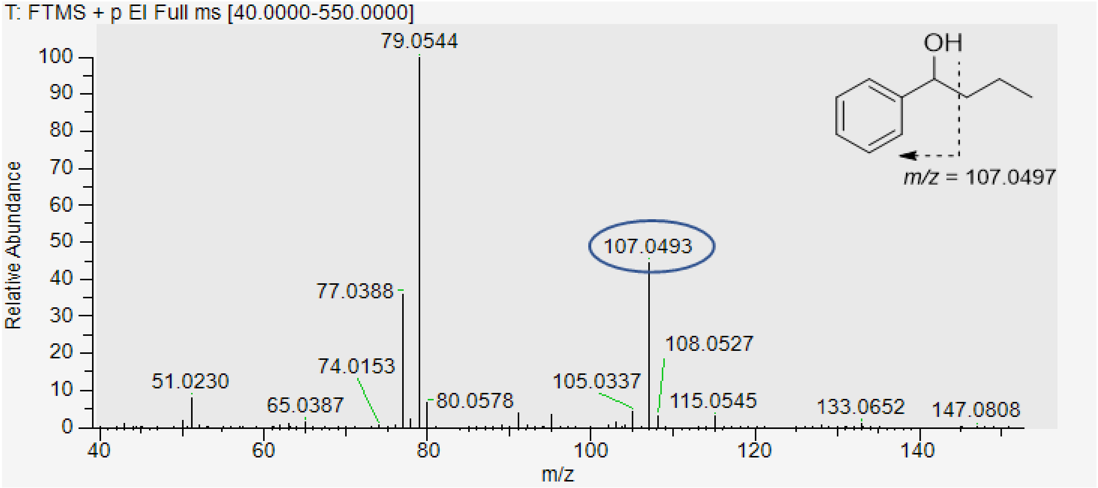

Figure S2. GC–MS spectrum of oxidation product 1.

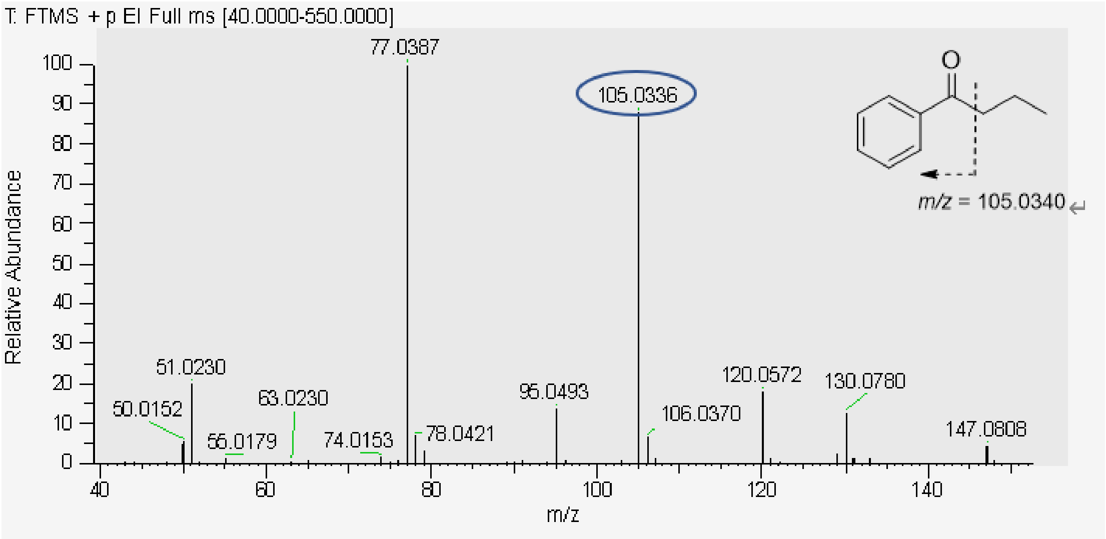

Figure S3. GC–MS spectrum of oxidation product 2.

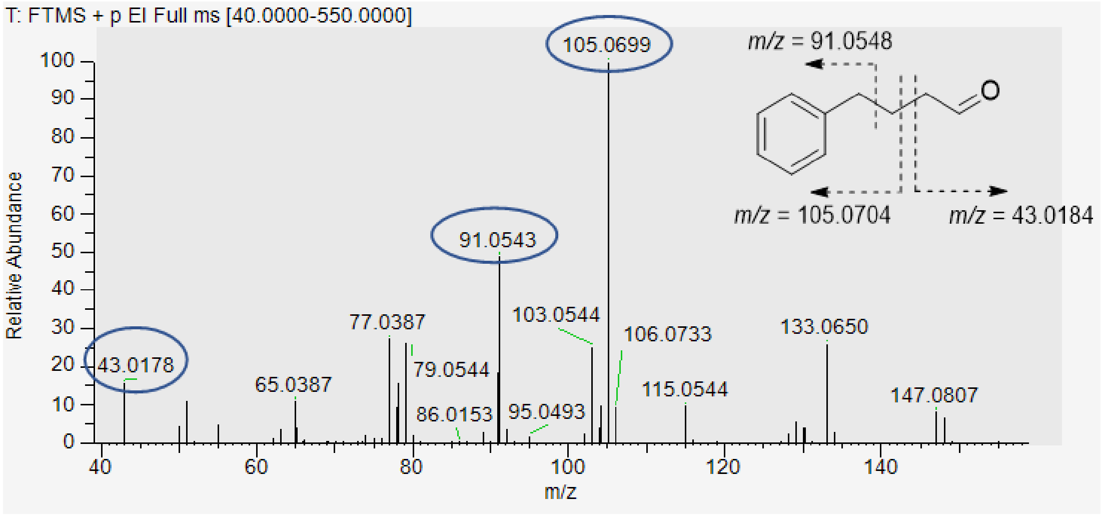

Figure S4. GC–MS spectrum of oxidation product 3.

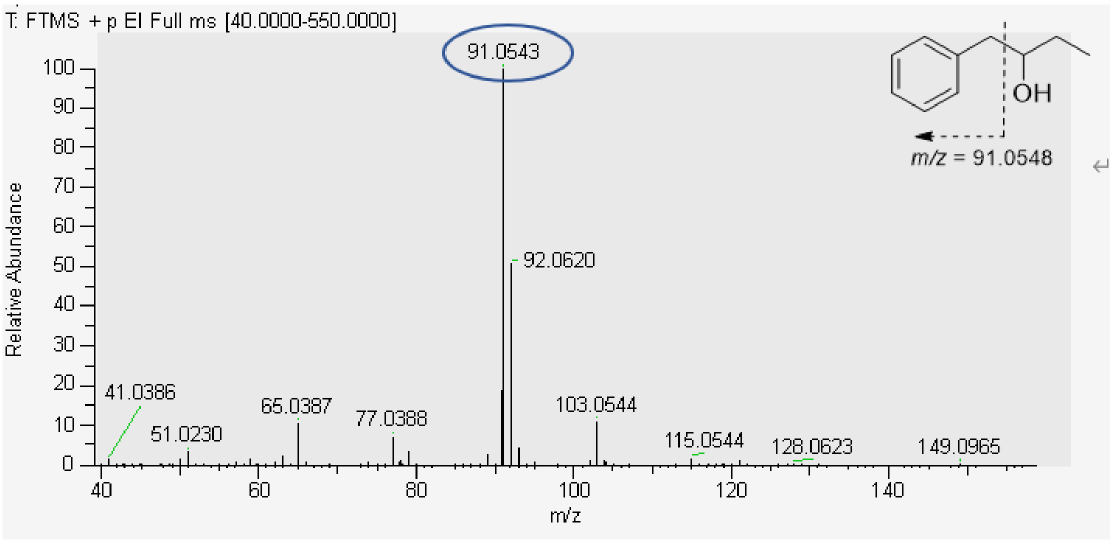

Figure S5. GC–MS spectrum of oxidation product 4.

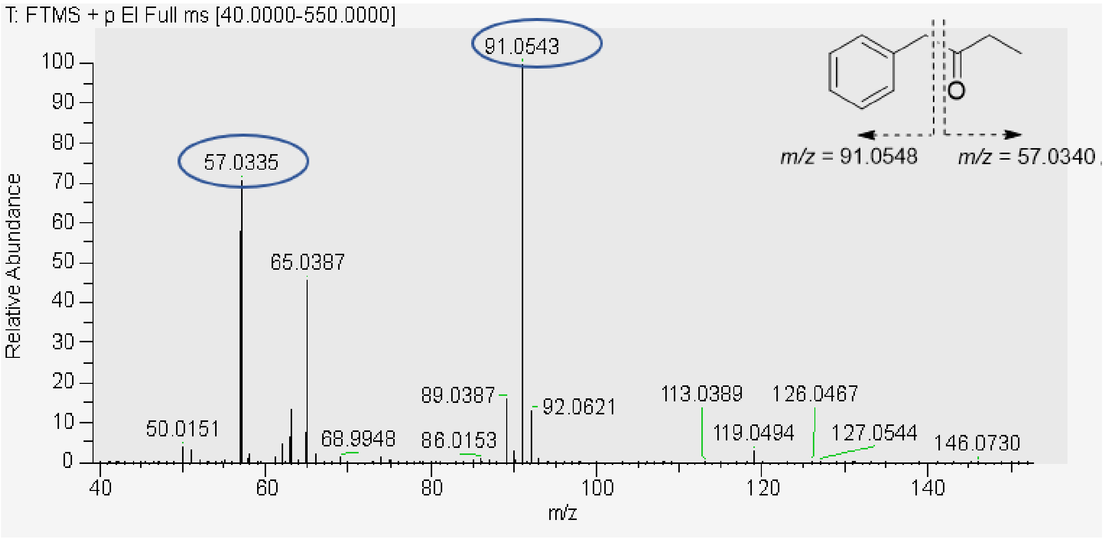

Figure S6. GC–MS spectrum of oxidation product 5.

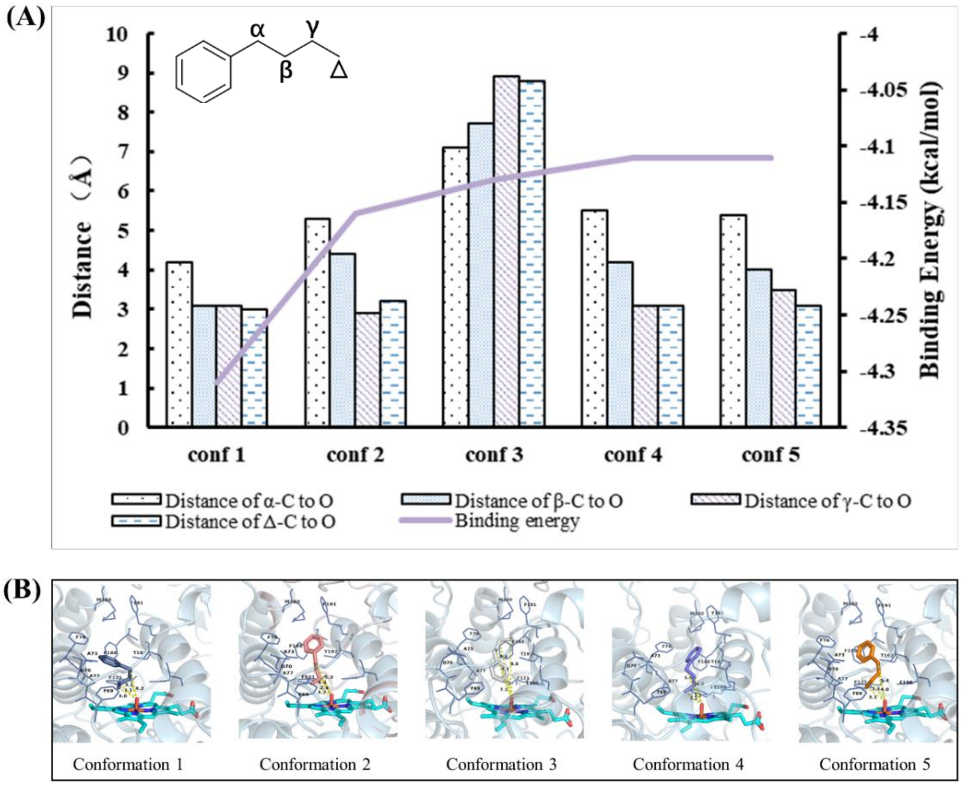

Figure S7. Active site model of r*Aae*UPO in complex with butyl benzene as substrate. (A): The top 5 conformations were ranked by binding energy and their distances of four carbon positions in butyl benzene to oxygen of compound I. (B): The top 5 conformations of butylbenzene inside the heme access channel of PaDa-1 predicted by AutoDock. Butylbenzene and heme cofactor are shown as sticks. Important active sites and catalytic residues are shown as lines. The dashed lines indicate distances (in Å) from the surrounding residues. As shown above, Δ and γ-C showed a closer distance to the oxygen atom of compound I in all conformations except conf 3, which had less reliability since no hydroxylation on the benzene ring was detected. The orientation of carbon atoms to the oxygen atom of compound I largely determines the probability of the reaction; thus, the regioselectivity of butylbenzene is very low.

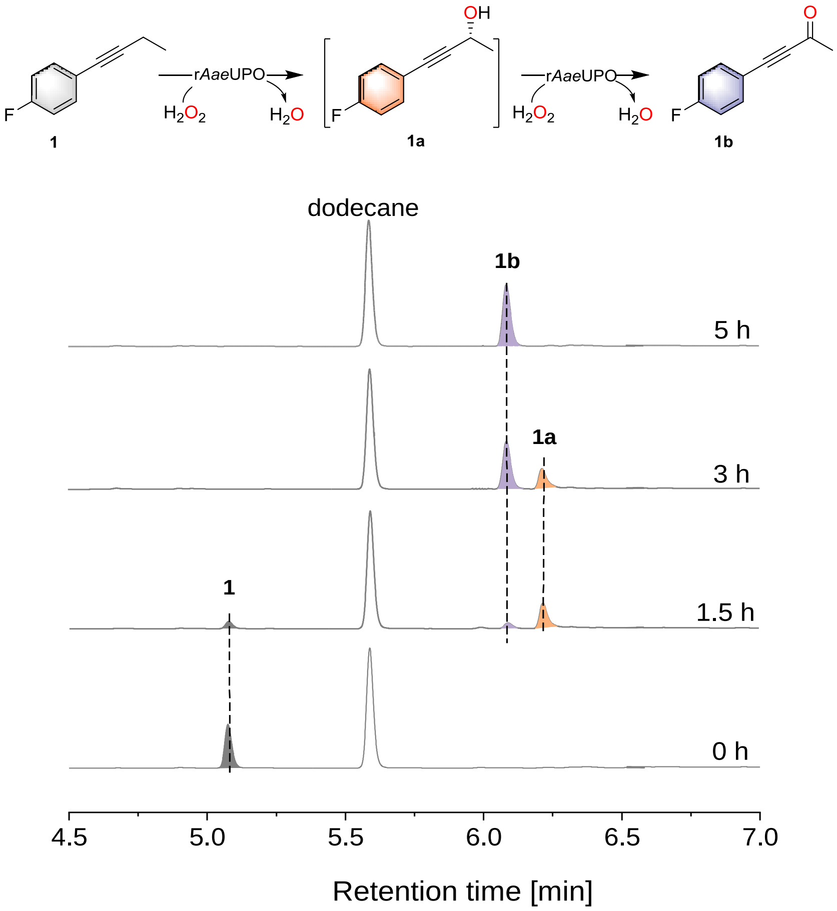

Figure S8. The GC chromatogram corresponds to the time course of C-H bond oxyfunctionalization catalyzed by r*Aae*UPO in Figure 1. The retention time of 1, 1a, 1b and the inner standard dodecane was 5.074, 6.213, 6.090 and 5.585 min, respectively.

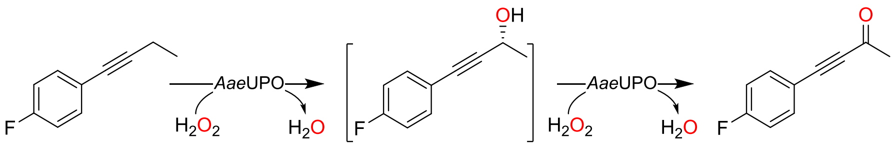

1 1a 1b

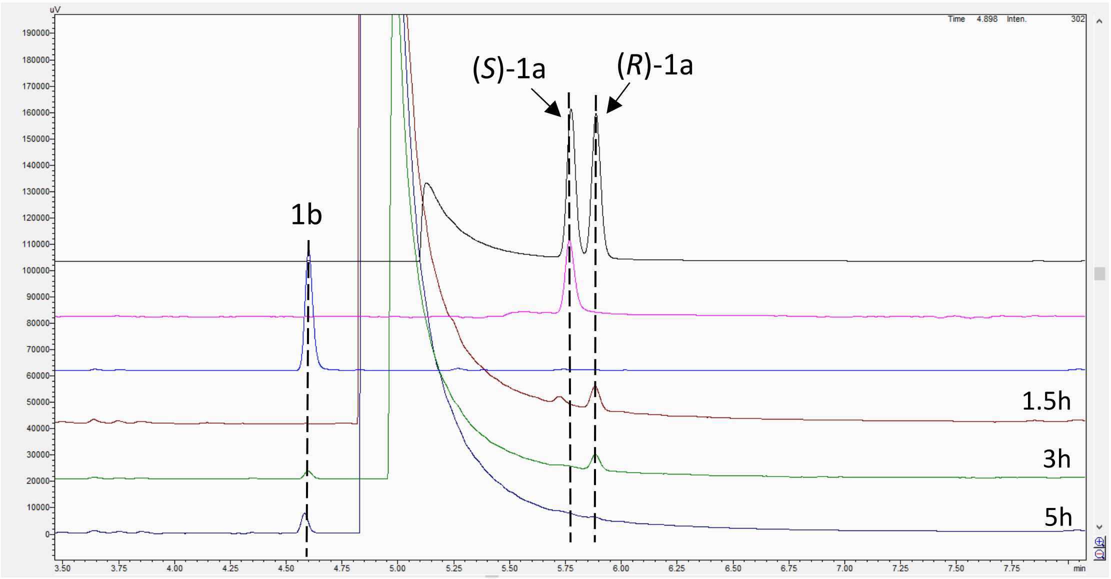

Figure S9. GC chromatogram of r*Aae*UPO-catalyzed C-H bond oxyfunctionalization of **1** to **1b**. From the black to blue line: commercial standard compounds of *rac*-**1a**, (*S*)-**1a** and **1b**, respectively. From the dark red to dark blue line: reactions for 1.5, 3 and 5 h, respectively. Sample preparation: After the reaction, the reaction mixture (100 μL) was extracted using ethyl acetate (200 μL) and dried over Na_2SO_4. Then, the samples were subjected to derivatization with acetic anhydride and DMAP for 1 h. The reaction was quenched by water and dried before analysis by GC equipped with a chiral CP7503 column (Column B). The broad peaks between 4.75 and 5.5 min in the GC chromatogram are from the impurities introduced during the derivatization process.

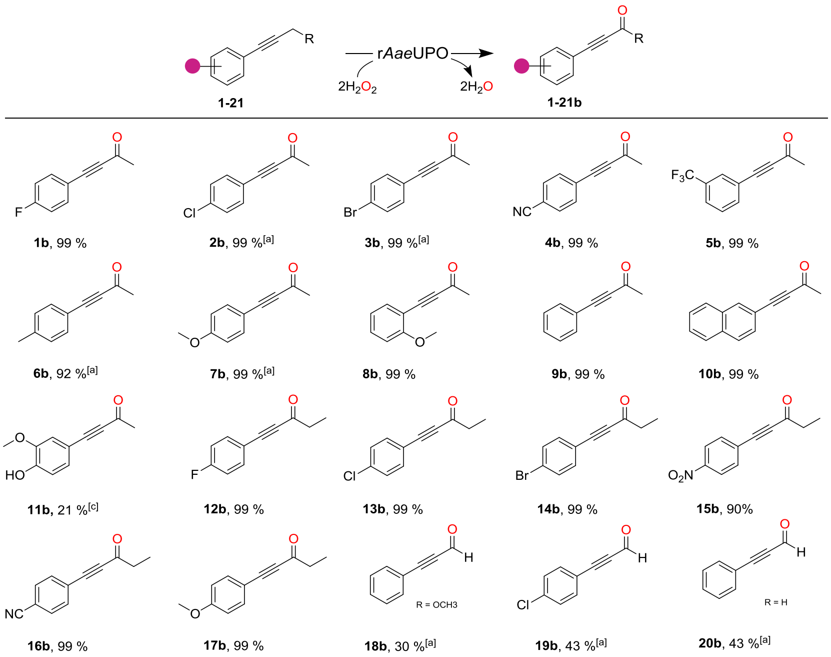

Figure S10. Substrate scope of peroxygenase-catalyzed oxidation of alkynes to ynones.

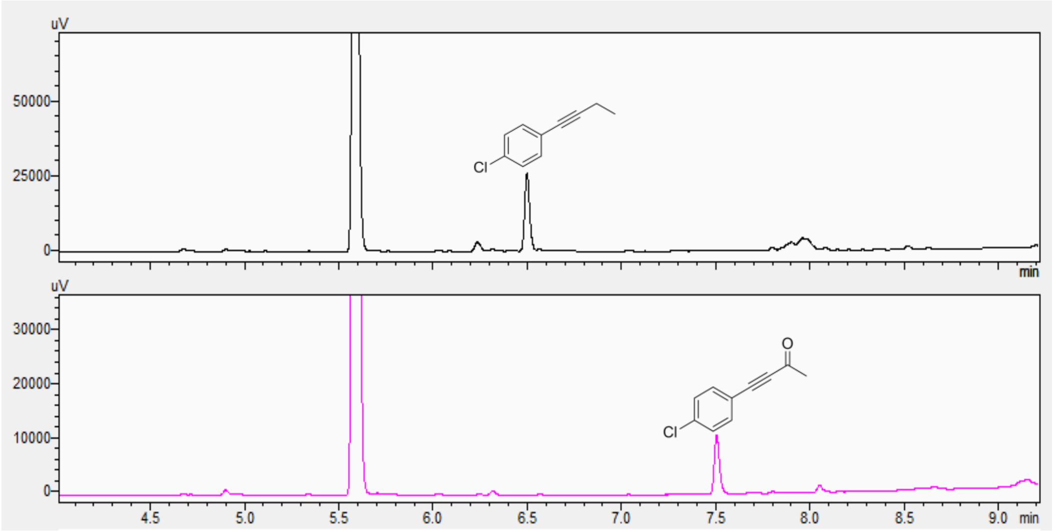

Figure S11. Representative GC chromatogram of r*Aae*UPO-catalyzed C-H bond oxyfunctionalization of **2** to **2b**.

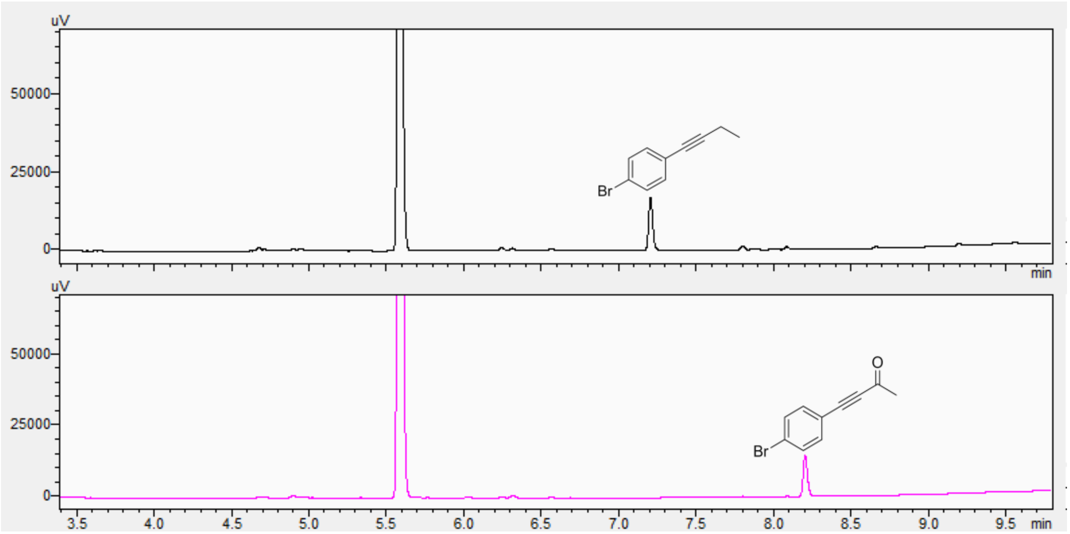

Figure S12. Representative GC chromatogram of r*Aae*UPO-catalyzed C-H bond oxyfunctionalization of **3** to **3b**.

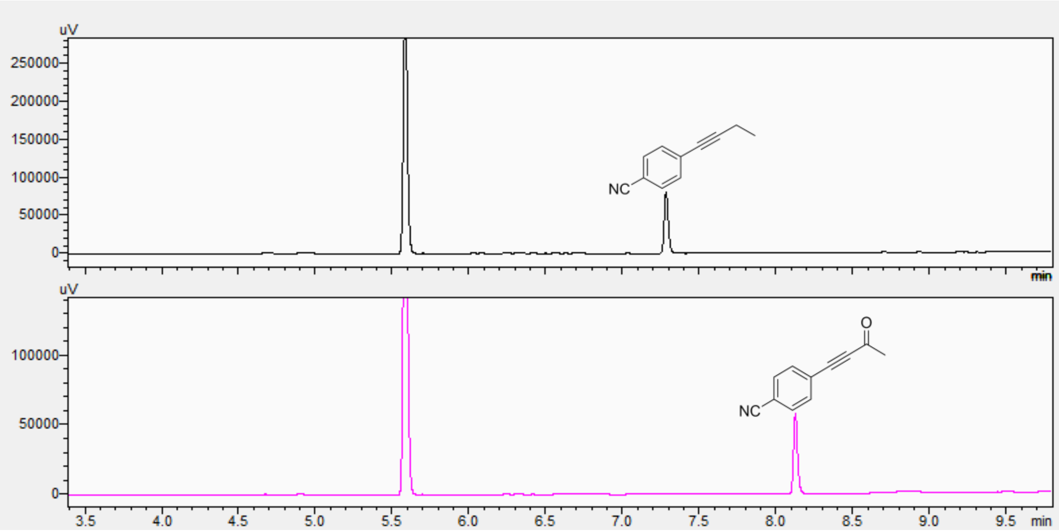

Figure S13. Representative GC chromatogram of r*Aae*UPO-catalyzed C-H bond oxyfunctionalization of **4** to **4b**.

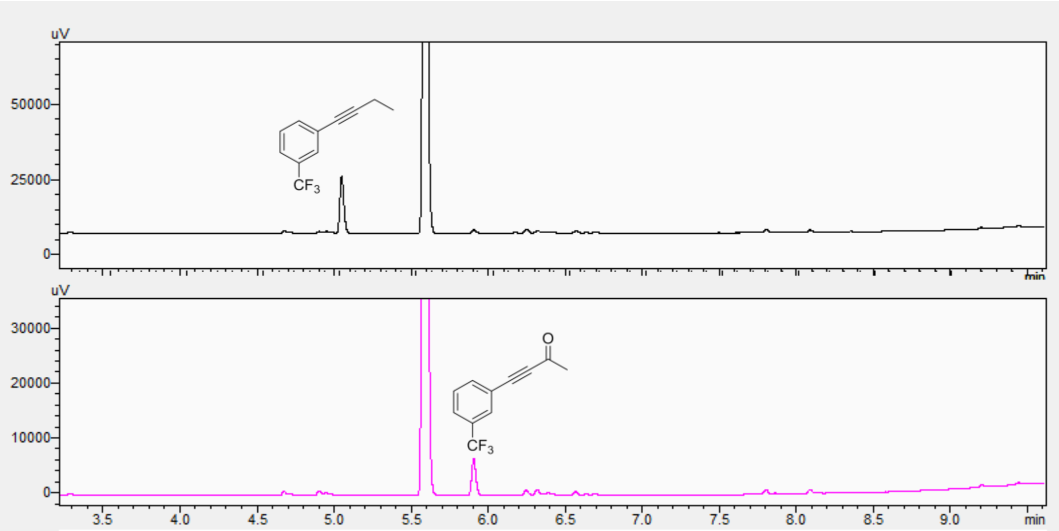

Figure S14. Representative GC chromatogram of r*Aae*UPO-catalyzed C-H bond oxyfunctionalization of **5** to **5b**.

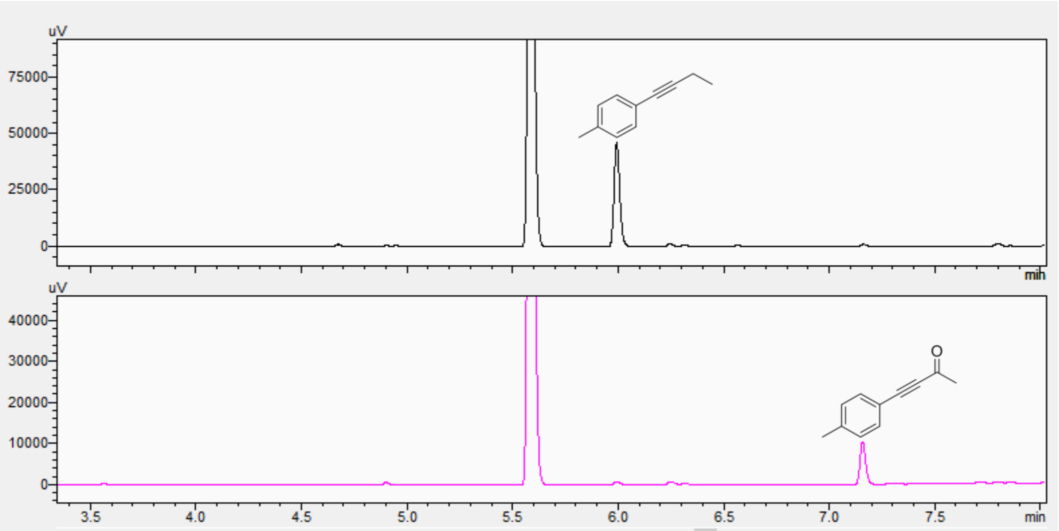

Figure S15. Representative GC chromatogram of r*Aae*UPO-catalyzed C-H bond oxyfunctionalization of **6** to **6b**.

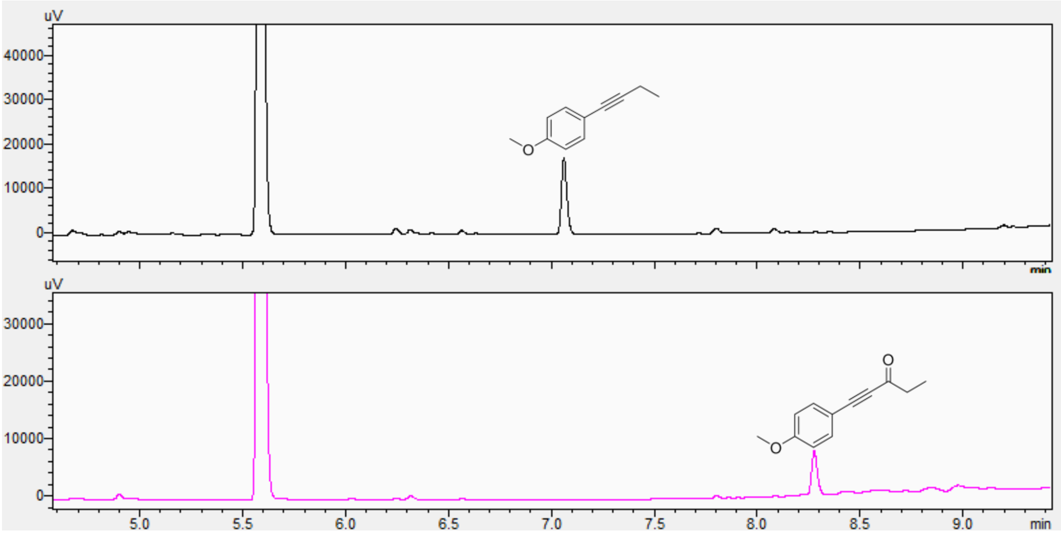

Figure S16. Representative GC chromatogram of r*Aae*UPO-catalyzed C-H bond oxyfunctionalization of **7** to **7b**.

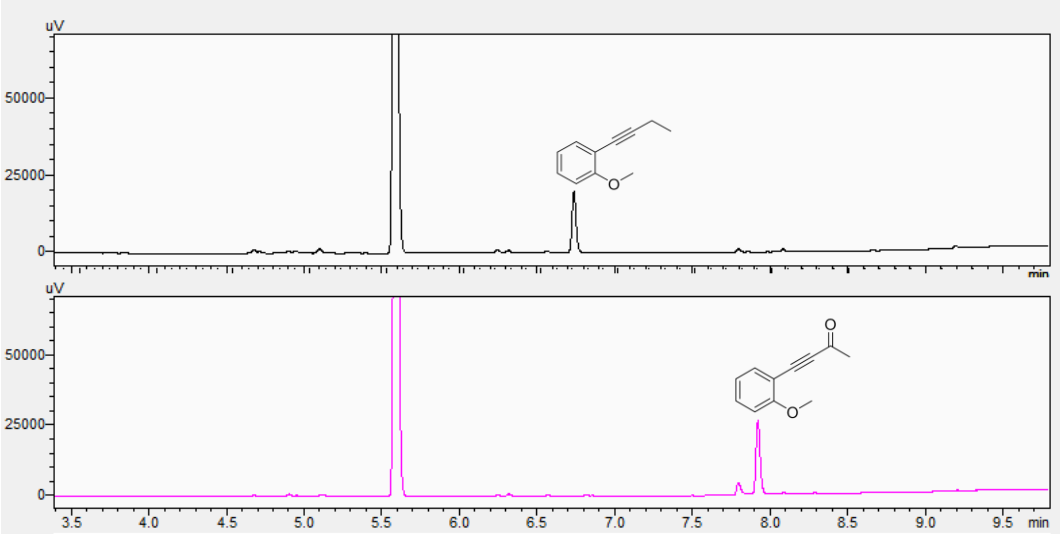

Figure S17. Representative GC chromatogram of r*Aae*UPO-catalyzed C-H bond oxyfunctionalization of **8** to **8b**.

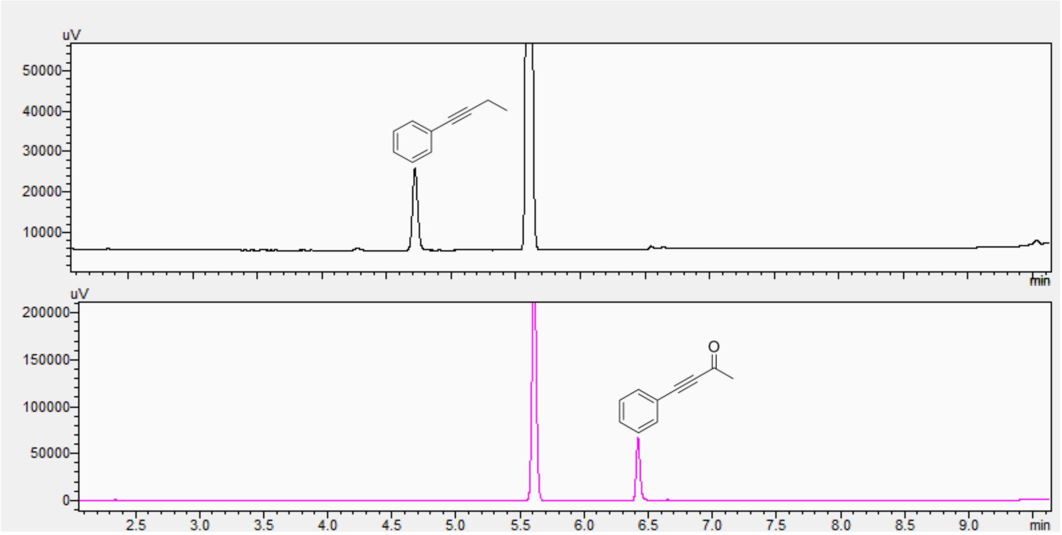

Figure S18. Representative GC chromatogram of r*Aae*UPO-catalyzed C-H bond oxyfunctionalization of **9** to **9b**.

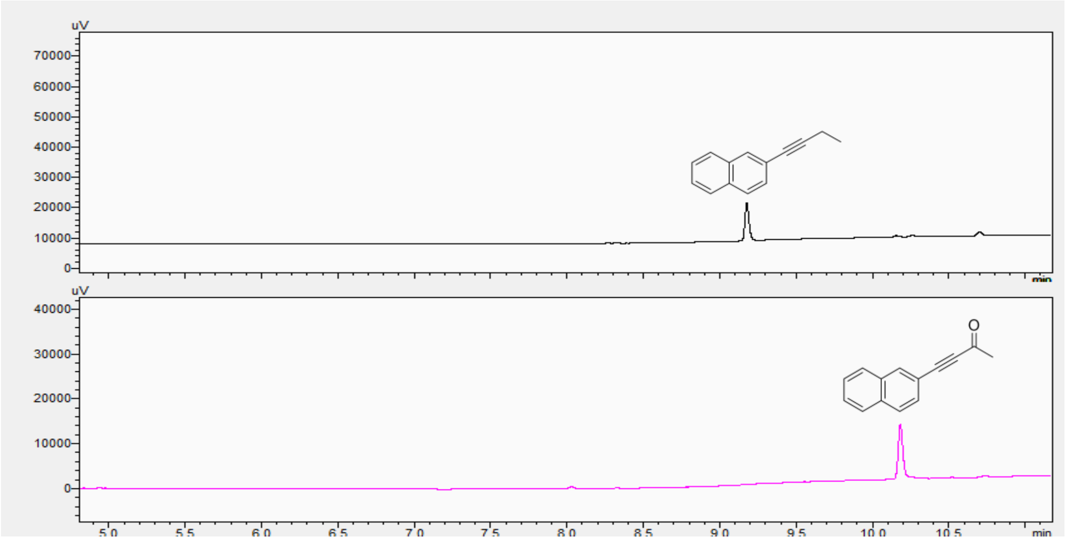

Figure S19. Representative GC chromatogram of r*Aae*UPO-catalyzed C-H bond oxyfunctionalization of **10** to **10b**.

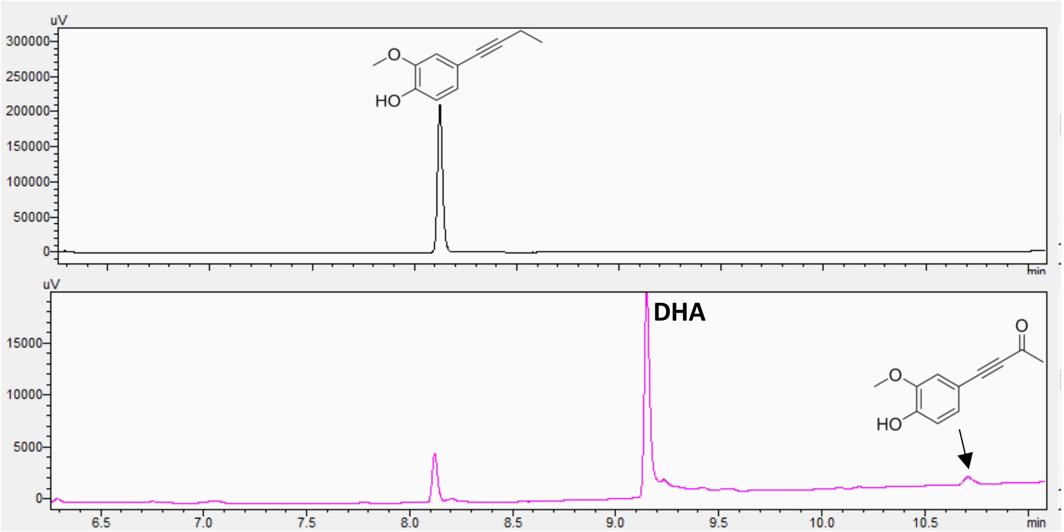

Figure S20. Representative GC chromatogram of r*Aae*UPO-catalyzed C-H bond oxyfunctionalization of **11** to **11b**. The peak of DHA represents the oxidation product of VC. DHA: Dehydroascorbic acid.

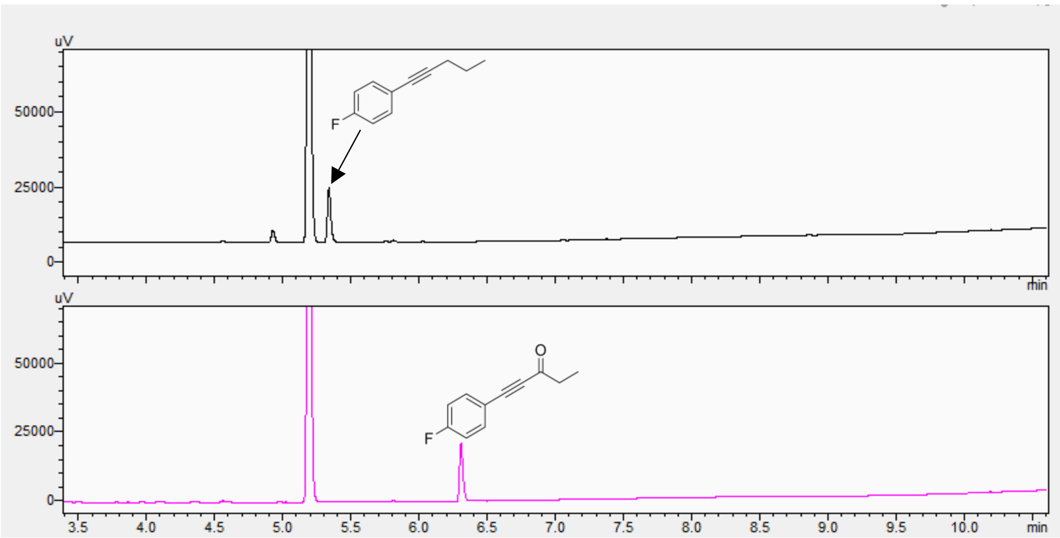

Figure S21. Representative GC chromatogram of r*Aae*UPO-catalyzed C-H bond oxyfunctionalization of **12** to **12b**.

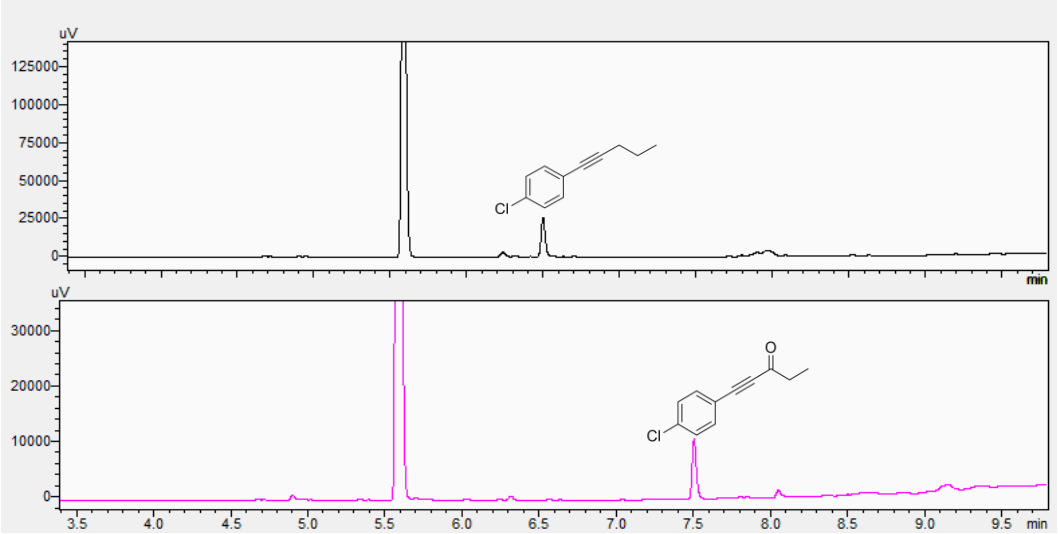

Figure S22. Representative GC chromatogram of r*Aae*UPO-catalyzed C-H bond oxyfunctionalization of **13** to **13b**.

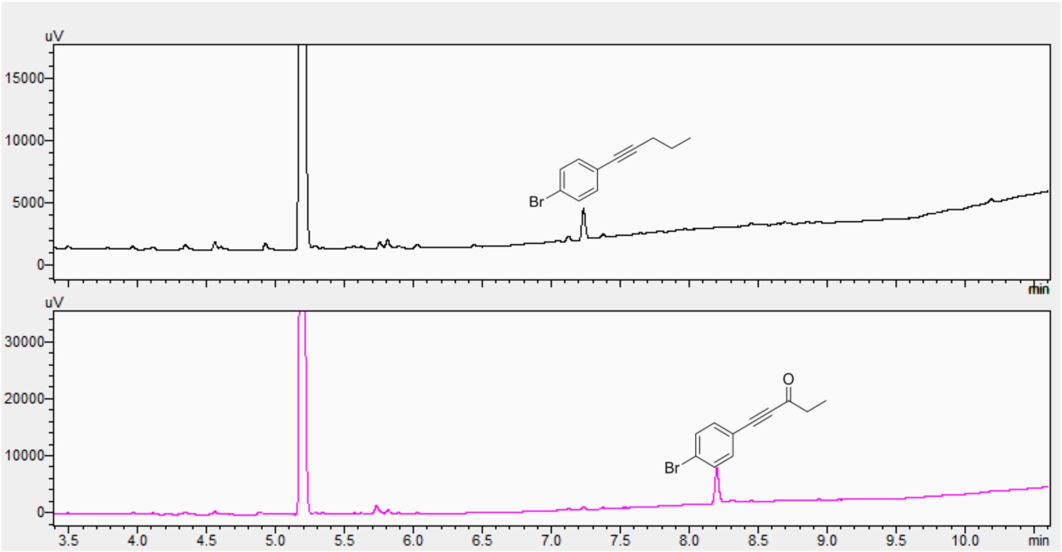

Figure S23. Representative GC chromatogram of r*Aae*UPO-catalyzed C-H bond oxyfunctionalization of **14** to **14b**.

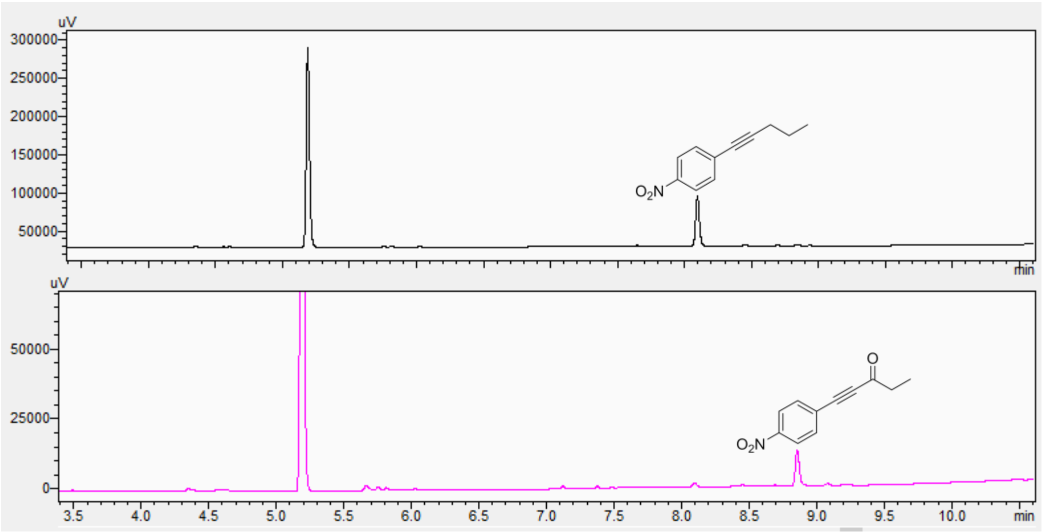

Figure S24. Representative GC chromatogram of r*Aae*UPO-catalyzed C-H bond oxyfunctionalization of **15** to **15b**.

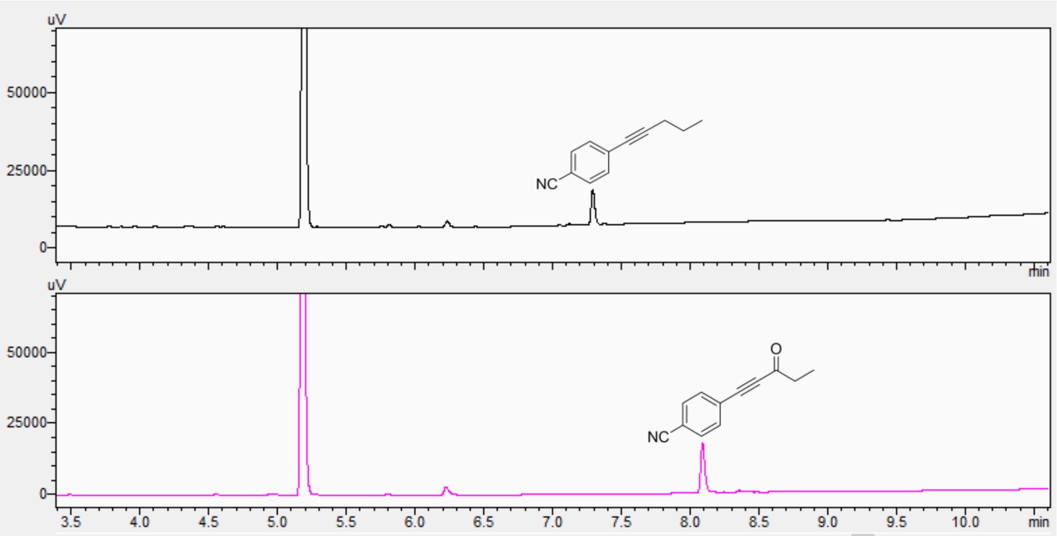

Figure S25. Representative GC chromatogram of r*Aae*UPO-catalyzed C-H bond oxyfunctionalization of **16** to **16b**.

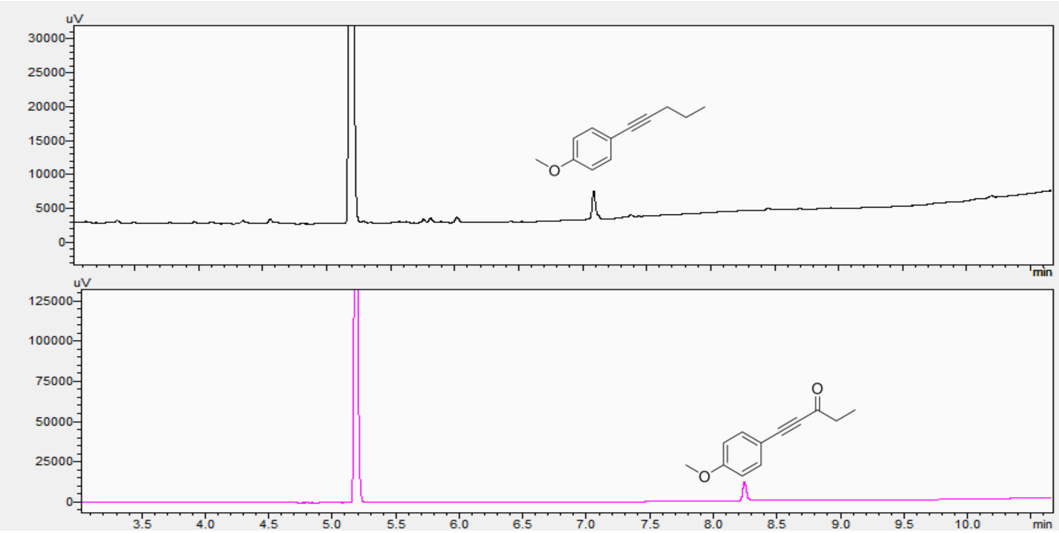

Figure S26. Representative GC chromatogram of r*Aae*UPO-catalyzed C-H bond oxyfunctionalization of **17** to **17b**.

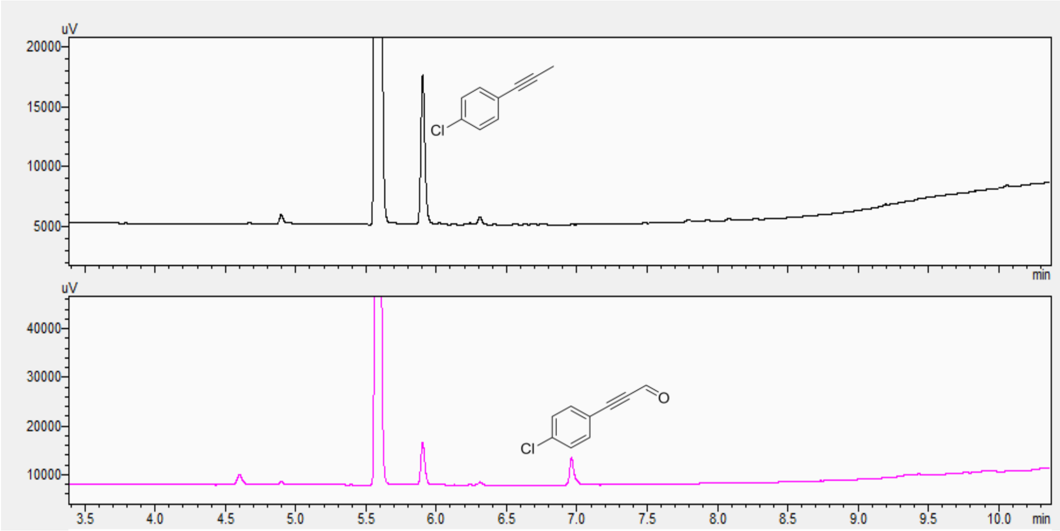

Figure S27. Representative GC chromatogram of r*Aae*UPO-catalyzed C-H bond oxyfunctionalization of **19** to **19b**.

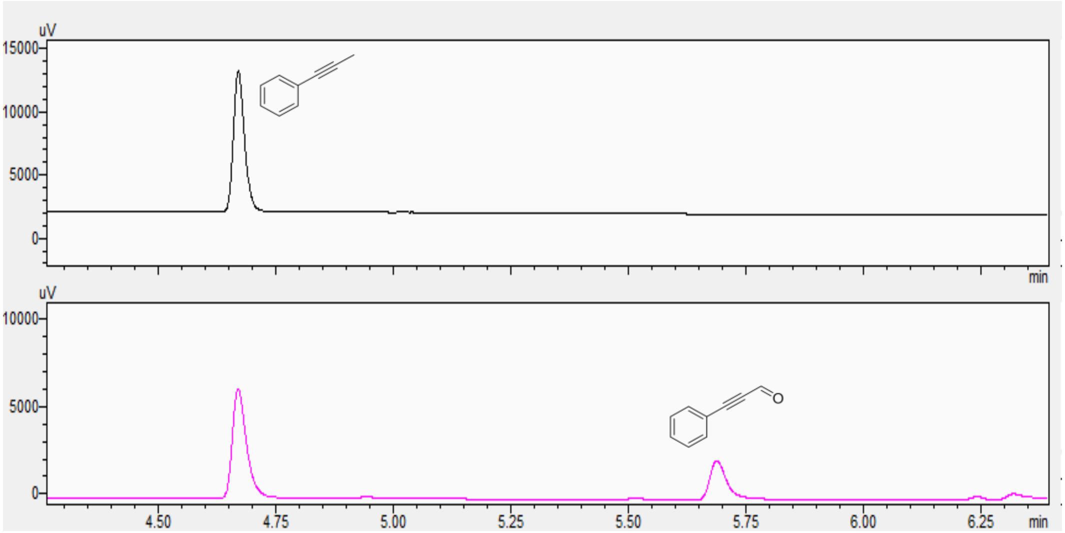

Figure S28. Representative GC chromatogram of r*Aae*UPO-catalyzed C-H bond oxyfunctionalization of **20** to **20b**.

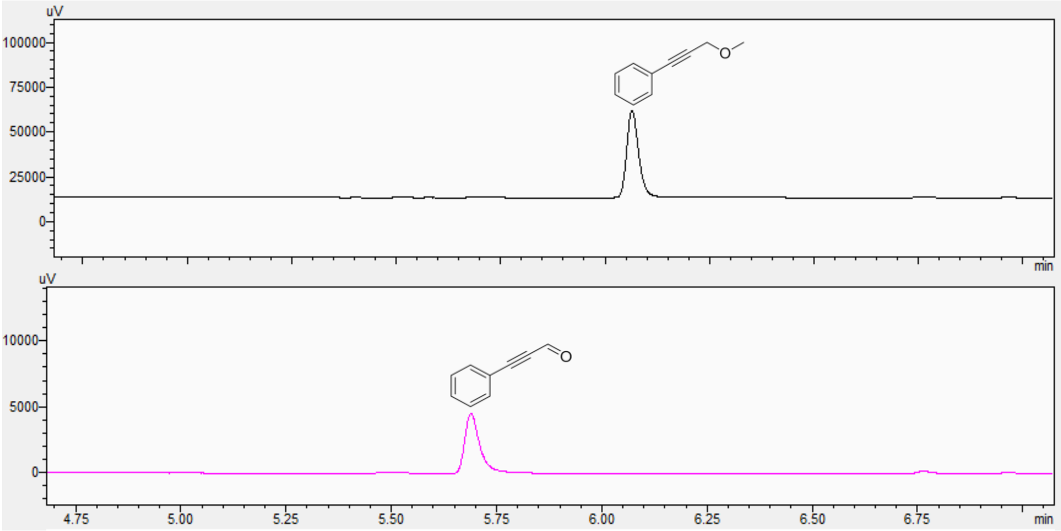

Figure S29. Representative GC chromatogram of r*Aae*UPO-catalyzed demethylation of **18** to **18b.**

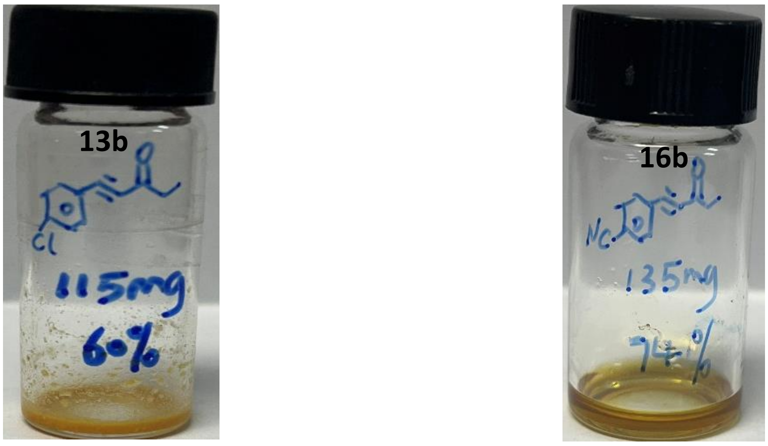

Figure S30. Image of isolated ynone products **13b** (left) and **16b** (right) from semipreparative reactions.

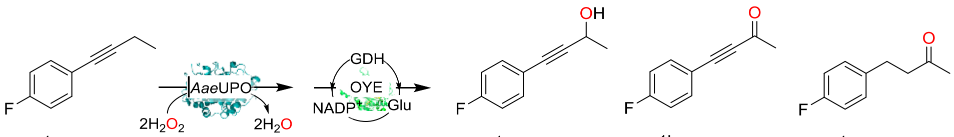

1 1a 1b 1c

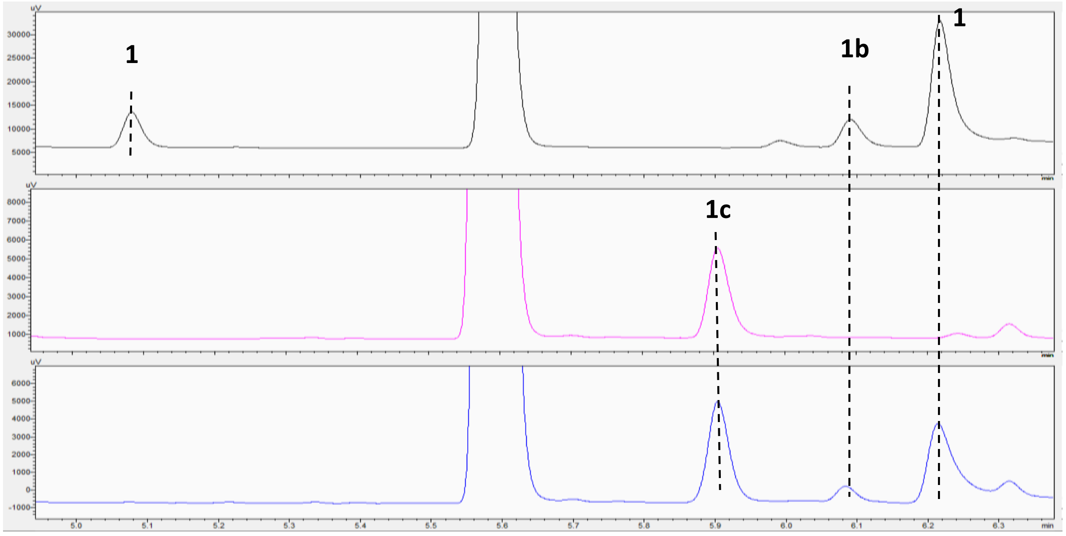

Figure S31. Representative GC chromatogram of r*Aae*UPO-catalyzed oxidation of alkynes combined with OYE3-catalyzed reduction for remote C-H bond oxyfunctionalization in a concurrent manner. The black and pink lines: standard compound **1**, **1a**, **1b** and **1c**. The blue pink line: reaction for 24h. The retention times of **1**, **1a**, **1b**, **1c** and the inner standard dodecane were 5.074, 6.090, 6.213, 5.906 and 5.585 min, respectively. Reaction conditions: [1a] = 5 mM, [r*Aae*UPO] = 500 nM, [H_2O_2] = 3 mM h^{-1}, 30% (v/v) MeCN, NaPi buffer (100 mM, pH = 7), [OYE3] = 25 µM, [Glu] = 50 mM, [GDH] = 1 mg mL^{-1}, [$NADP^+$] = 2 mM, 800 rpm, 30 °C, 1 mL, 24h.

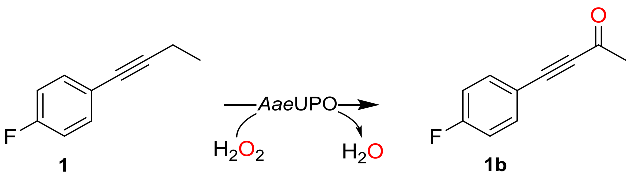

Table S3. Control reactions for oxidation **1** using *r*AaeUPO[a]

Entry	OYE3	Glu	GDH	NADP$^+$	Yield (%) [b]
1	-	-	-	-	>99
2	+	-	-	-	>99
3	-	+	-	-	>99
4	-	-	+	-	>99
5	-	-	-	+	>99

[a] Reaction conditions: [substrate] = 5 mM, NaPi buffer (100 mM), pH = 7, [*r*AaeUPO] = 500 nM, [OYE3] = 25 µM, [Glu] = 50 mM, [GDH] = 1 mg mL^{-1}, [NADP$^+$] = 2 mM, 30% MeCN, 30 °C, 800 rpm, 1 mL.

[b]The yield was calculated based on the substrate and product peaks determined by GC.

"+" means containing this substance. "-" means not containing this substance.

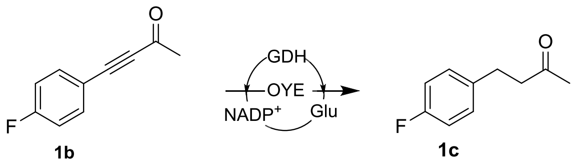

Table S4. Control reactions for the reduction of **1b** using OYE3[a]

Entry	H_2O_2	*r*AaeUPO	Yield (%) [b]
1	-	-	>99
2	-	+	>99
3	+	-	>99
4	+	+	>99

[a] Reaction conditions: [substrate] = 5 mM, NaPi buffer (100 mM), pH = 7, [*r*AaeUPO] = 500 nM, [OYE3] = 25 µM, [Glu] = 50 mM, [GDH] = 1 mg mL^{-1}, [NADP$^+$] = 2 mM, 30% MeCN, 30 °C, 800 rpm, 1 mL.

[b] The yield was calculated based on the substrate and product peaks determined by GC.

"+" means containing this substance. "-" means not containing this substance.

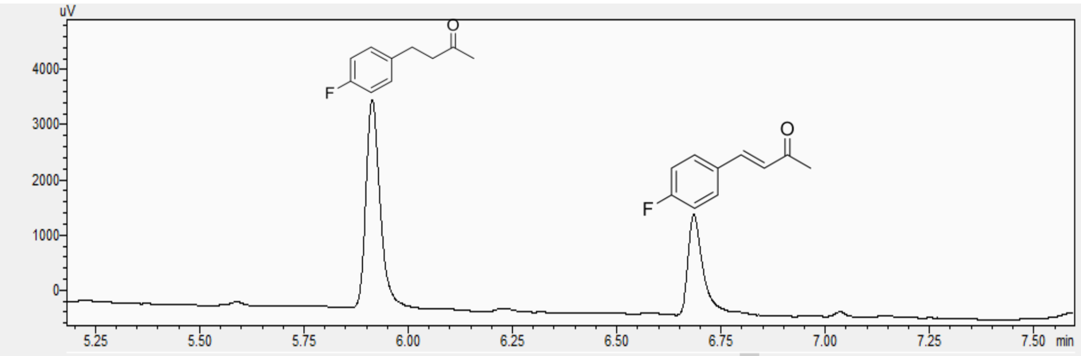

Figure S32. Representative GC chromatogram showing the enone intermediate.

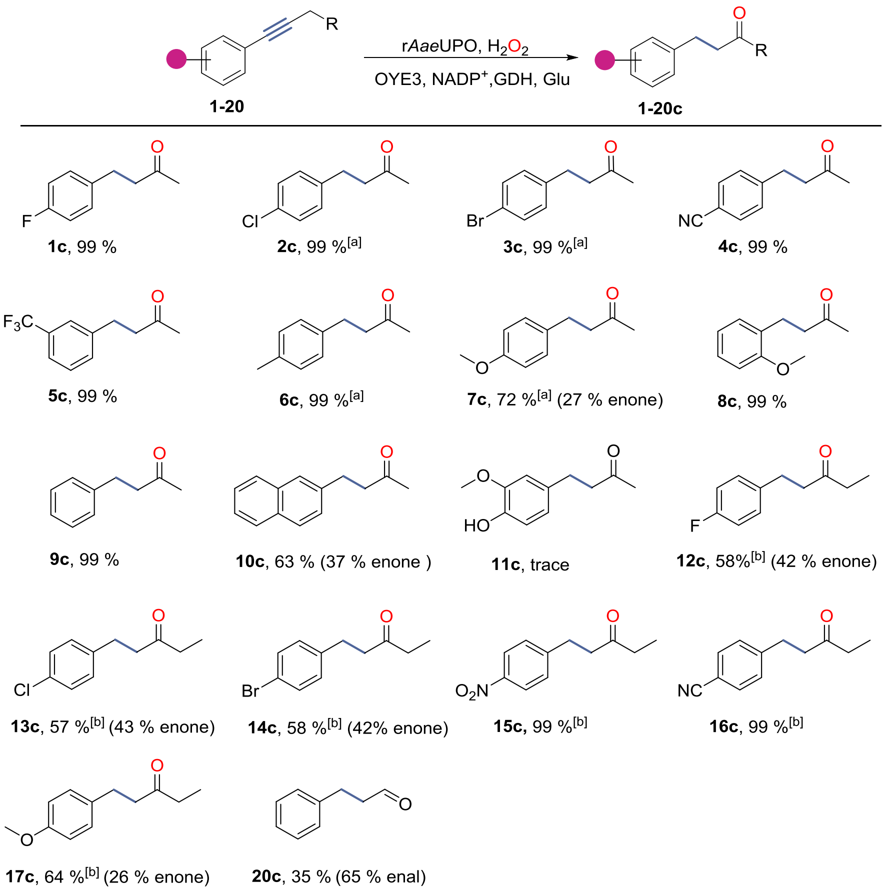

Figure S33. Substrate scope of the cascade between r*Aae*UPO and OYE3 for C-H bond oxyfunctionalization.

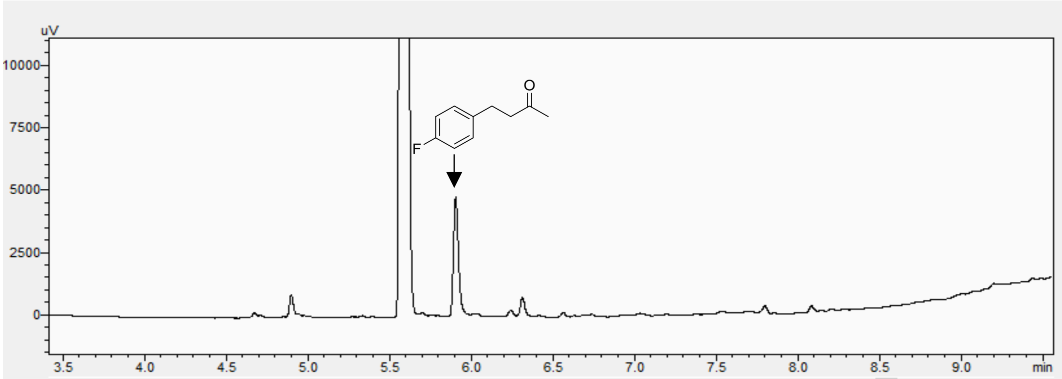

Figure S34. Representative GC chromatogram of the cascade between r*Aae*UPO and OYE3 for remote C-H bond oxyfunctionalization of **1** to **1c**.

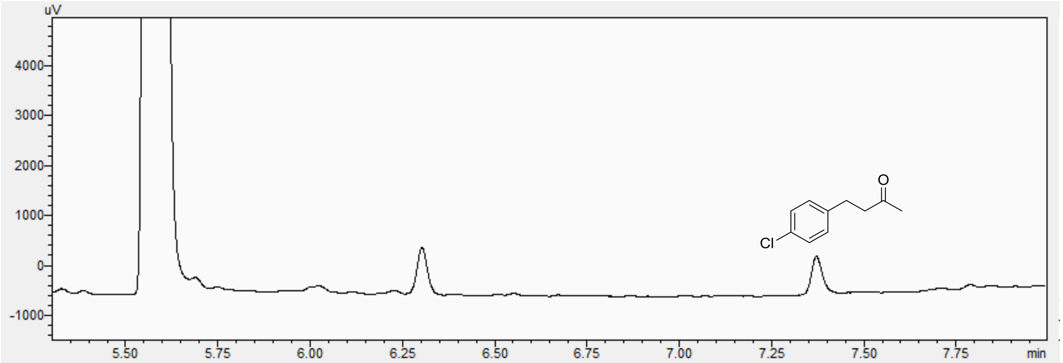

Figure S35. Representative GC chromatogram of the cascade between r*Aae*UPO and OYE3 for remote C-H bond oxyfunctionalization of **2** to **2c**. Please note, the peak at 6.303 min was impurity that was observed in most of the reduction reactions.

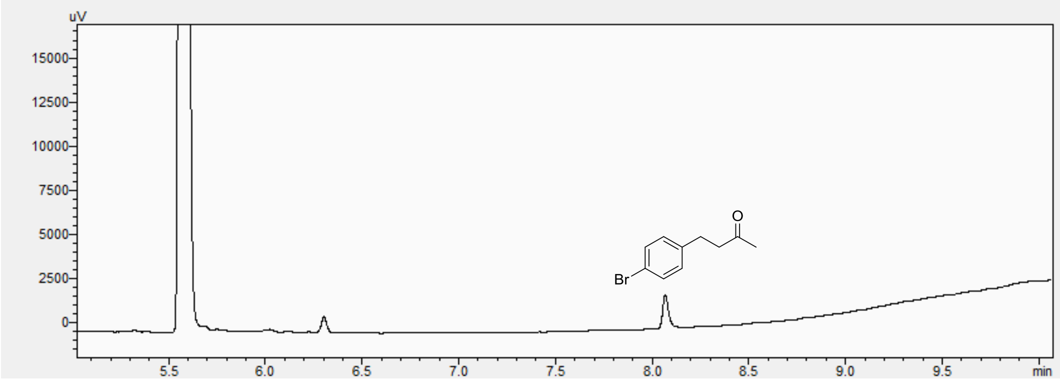

Figure S36. Representative GC chromatogram of the cascade between r*Aae*UPO and OYE3 for remote C-H bond oxyfunctionalization of **3** to **3c**. The peak of 6.303 min was impurity.

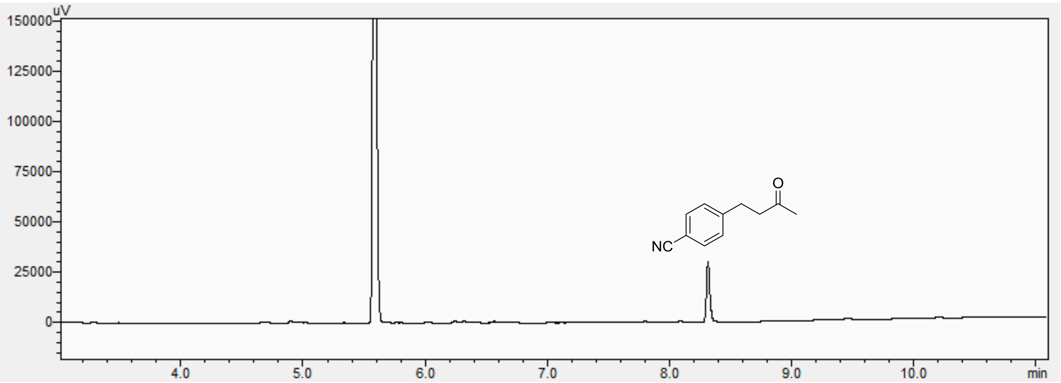

Figure S37. Representative GC chromatogram of the cascade between r*Aae*UPO and OYE3 for remote C-H bond oxyfunctionalization of **4** to **4c**.

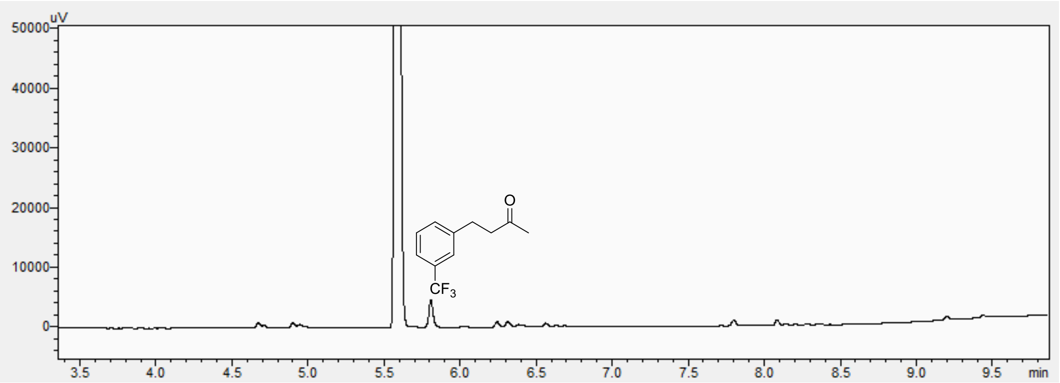

Figure S38. Representative GC chromatogram of the cascade between r*Aae*UPO and OYE3 for remote C-H bond oxyfunctionalization of **5** to **5c**.

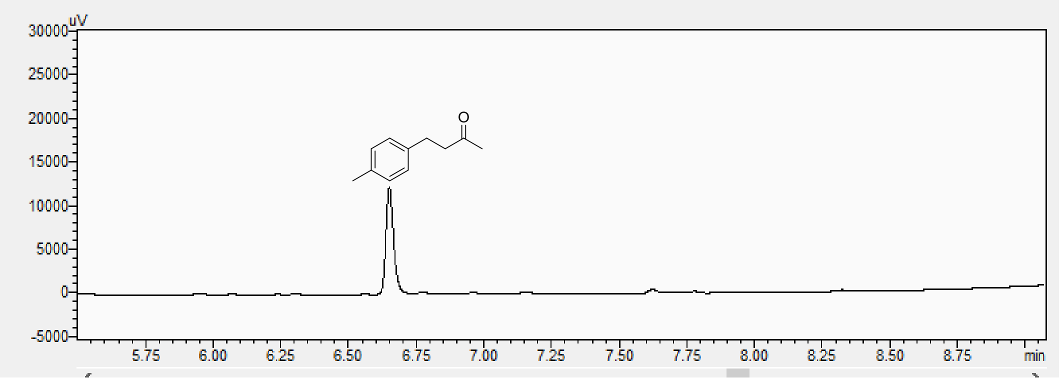

Figure S39. Representative GC chromatogram of the cascade between r*Aae*UPO and OYE3 for remote C-H bond oxyfunctionalization of **6** to **6c**.

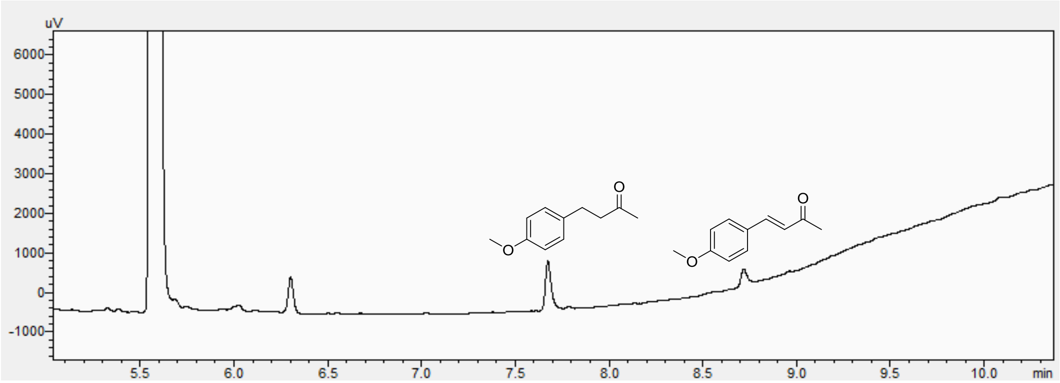

Figure S40. Representative GC chromatogram of the cascade between r*Aae*UPO and OYE3 for remote C-H bond oxyfunctionalization of **7** to **7c.** The peak of 6.303 min was impurity.

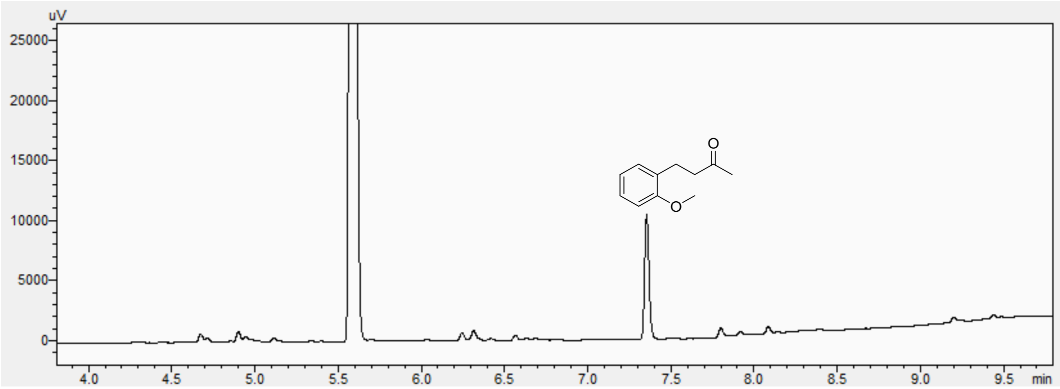

Figure S41. Representative GC chromatogram of the cascade between r*Aae*UPO and OYE3 for remote C-H bond oxyfunctionalization of **8** to **8c.**

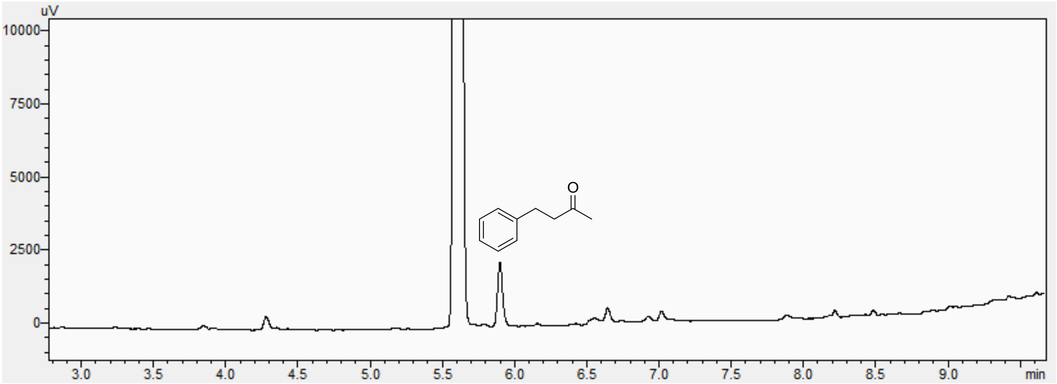

Figure S42. Representative GC chromatogram of the cascade between r*Aae*UPO and OYE3 for remote C-H bond oxyfunctionalization of **9** to **9c.**

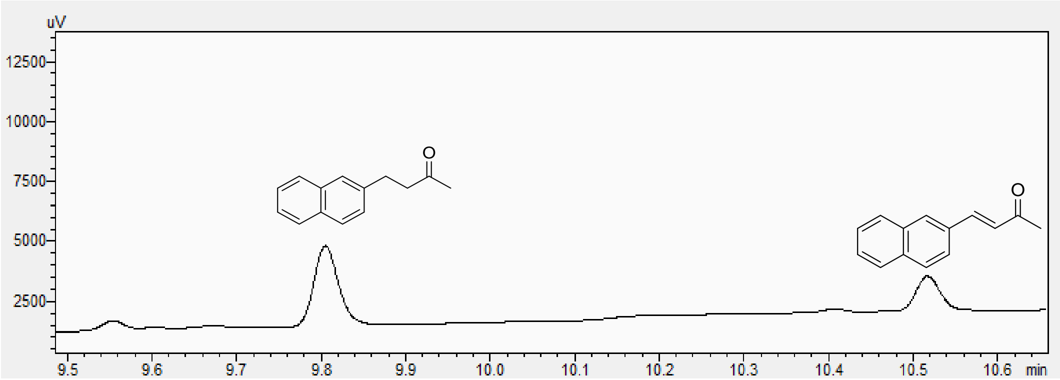

Figure S43. Representative GC chromatogram of the cascade between r*Aae*UPO and OYE3 for remote C-H bond oxyfunctionalization of **10** to **10c.**

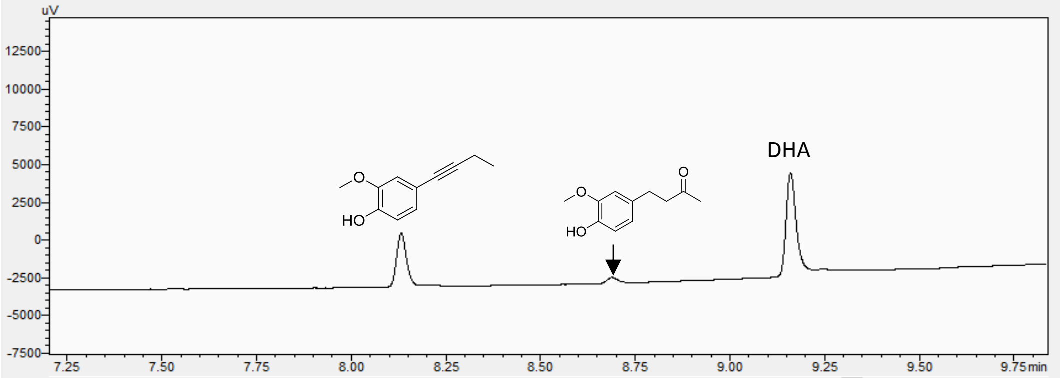

Figure S44. Representative GC chromatogram of the cascade between *rAae*UPO and OYE3 for remote C-H bond oxyfunctionalization of **11** to **11c.** The peak of DHA represents the oxidation product of VC.

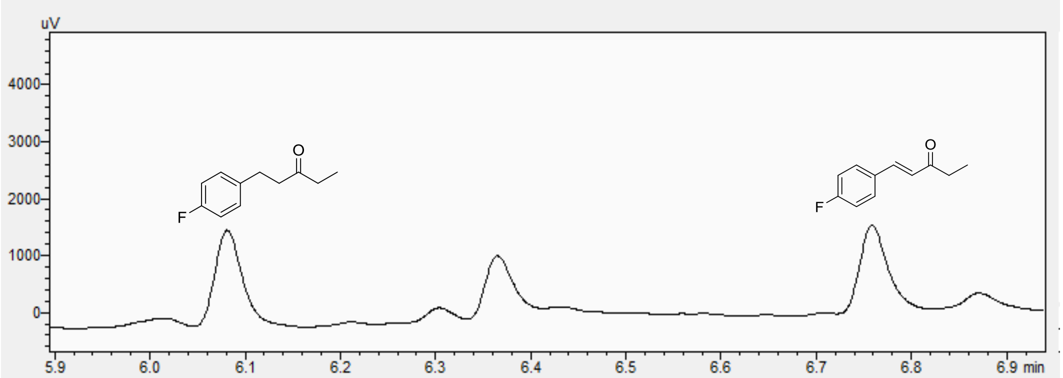

Figure S45. Representative GC chromatogram of the cascade between r*Aae*UPO and OYE3 for remote C-H bond oxyfunctionalization of **12** to **12c.**

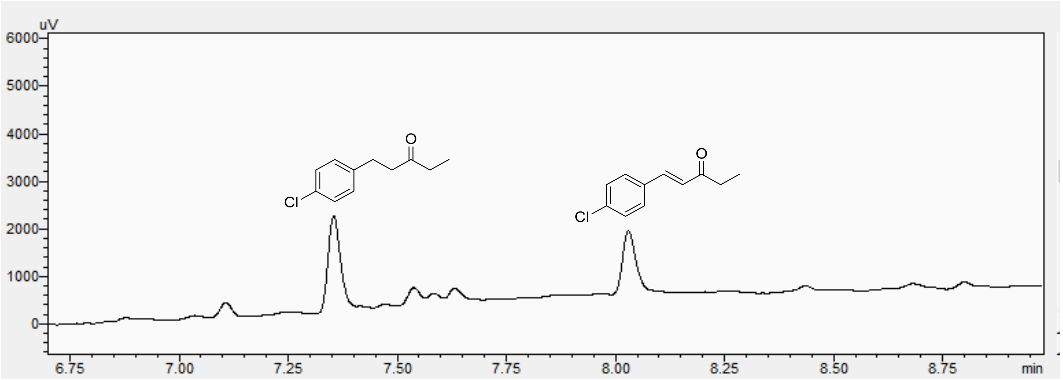

Figure S46. Representative GC chromatogram of the cascade between *rAae*UPO and OYE3 for remote C-H bond oxyfunctionalization of **13** to **13c.**

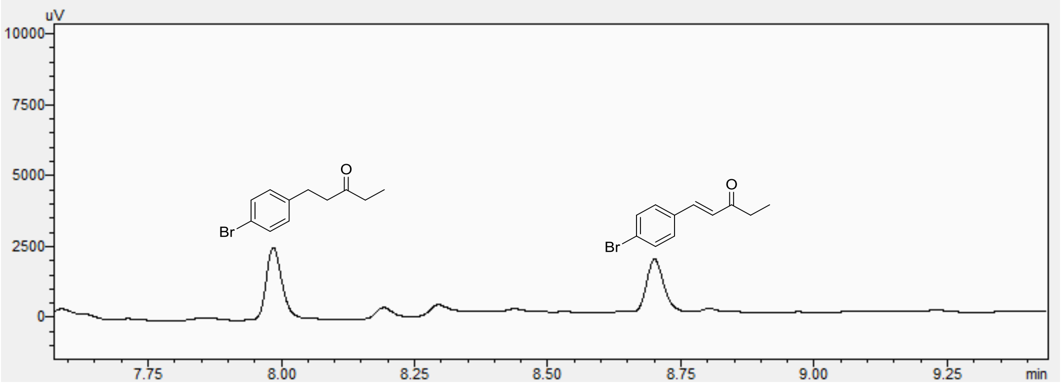

Figure S47. Representative GC chromatogram of the cascade between r*Aae*UPO and OYE3 for remote C-H bond oxyfunctionalization of **14** to **14c.**

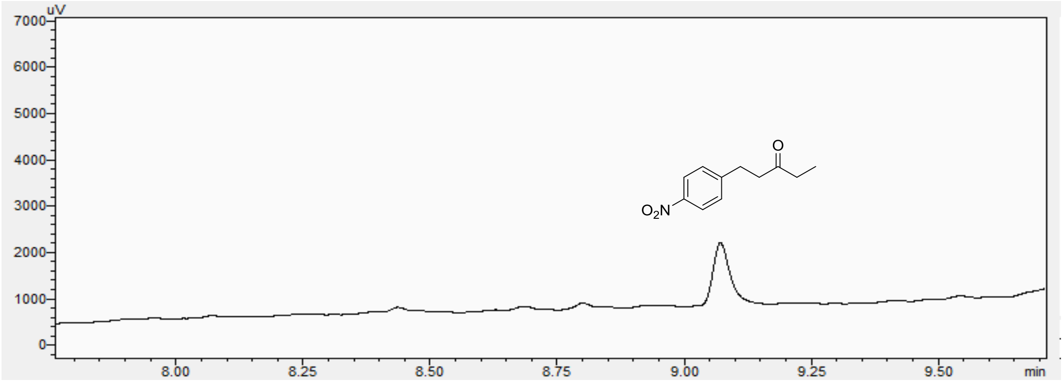

Figure S48. Representative GC chromatogram of the cascade between r*Aae*UPO and OYE3 for remote C-H bond oxyfunctionalization of **15** to **15c.**

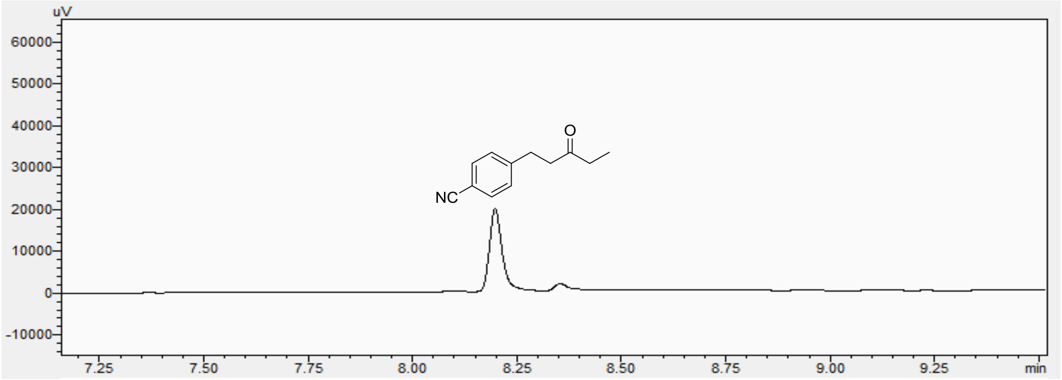

Figure S49. Representative GC chromatogram of the cascade between r*Aae*UPO and OYE3 for remote C-H bond oxyfunctionalization of **16** to **16c.**

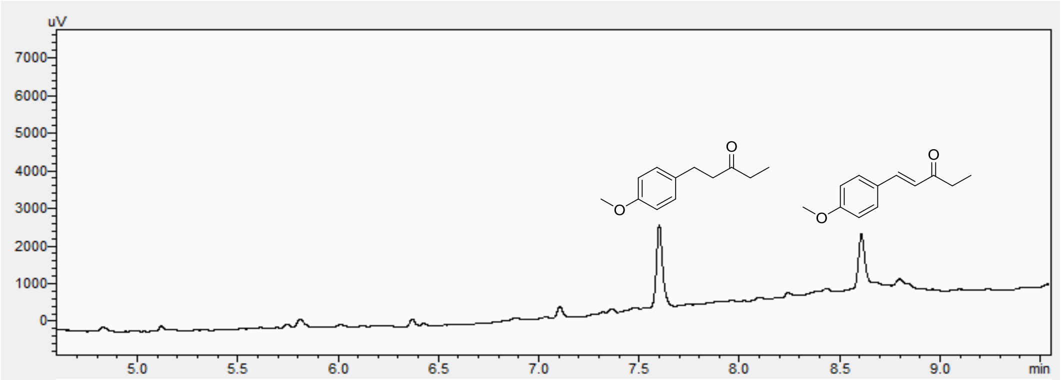

Figure S50. Representative GC chromatogram of the cascade between r*Aae*UPO and OYE3 for remote C-H bond oxyfunctionalization of **17** to **17c.**

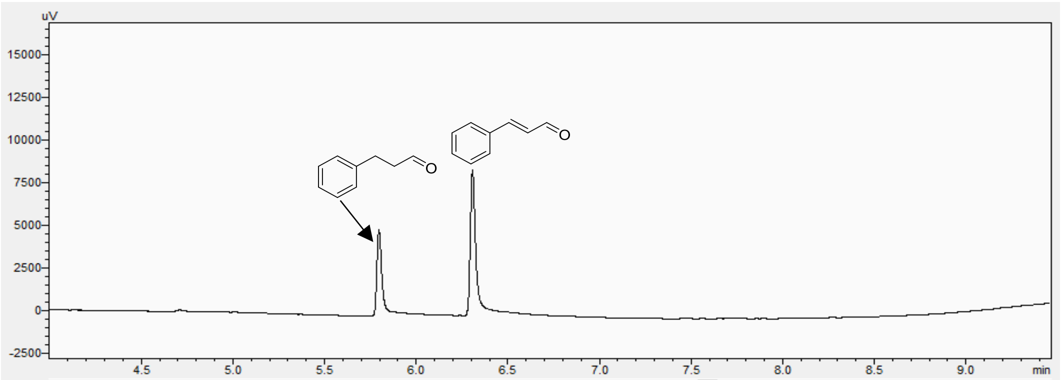

Figure S51. Representative GC chromatogram of the cascade between r*Aae*UPO and OYE3 for remote C-H bond oxyfunctionalization of **20** to **20c.**

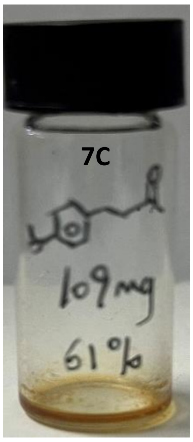

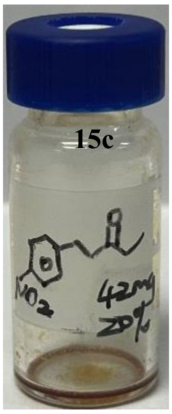

Figure S52. Image of isolated remote ketone products **7c** (left) and **15c** (right) from semipreparative reactions.

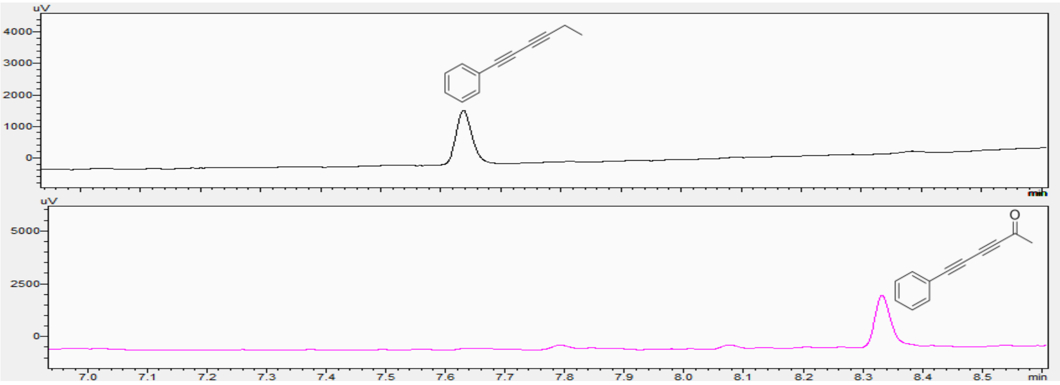

Figure S53. Representative GC chromatogram of r*Aae*UPO -catalyzed oxidation of **22** to **22b**.

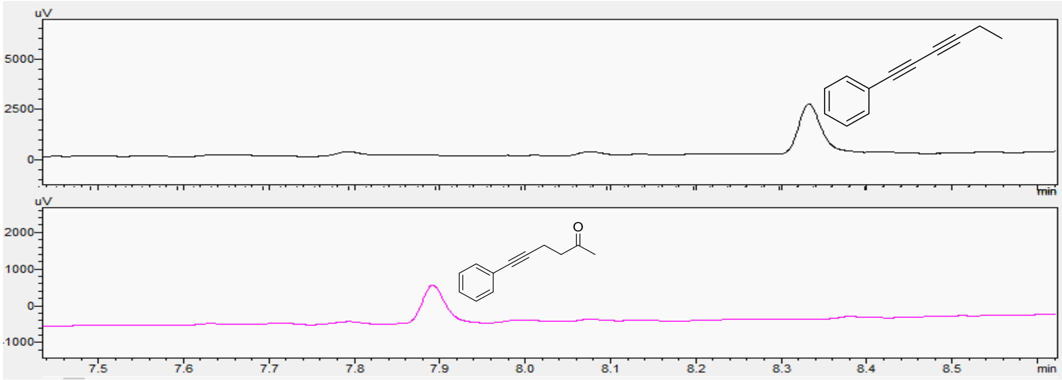

Figure S54. Representative GC chromatogram of the cascade between r*Aae*UPO and OYE3 for remote C-H bond oxyfunctionalization of **22** to **22c.**

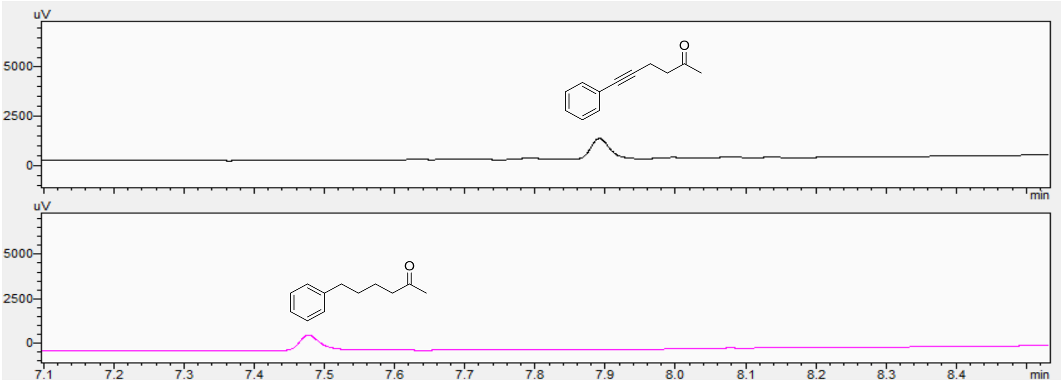

Figure S55. Representative GC chromatogram of the cascade r*Aae*UPO, OYE3 and Pd-C/H_2 for remote C-H bond oxyfunctionalization of **21** to **21d.**

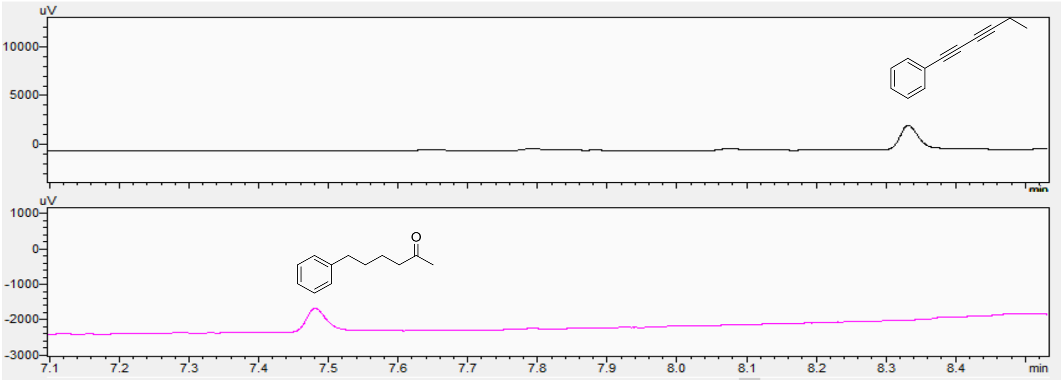

Figure S56. Representative GC chromatogram of the cascade between r*Aae*UPO and Pd-C/H_2 for remote C-H bond oxyfunctionalization of **21** to **21d.**

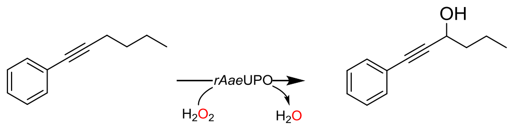

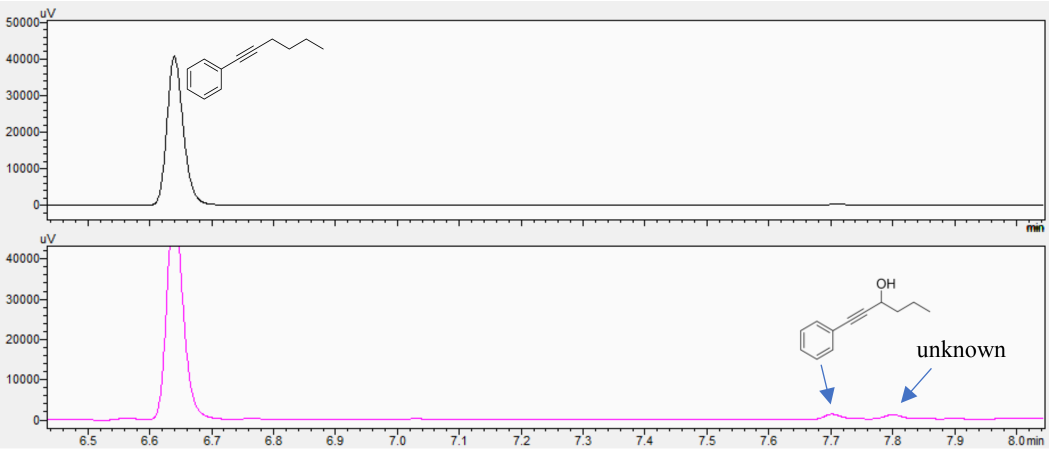

Figure S57. Representative GC chromatogram of r*Aae*UPO-catalyzed C-H bond oxyfunctionalization of hexyne. The alkynol product was confirmed by GC-MS shown in Figure S58.

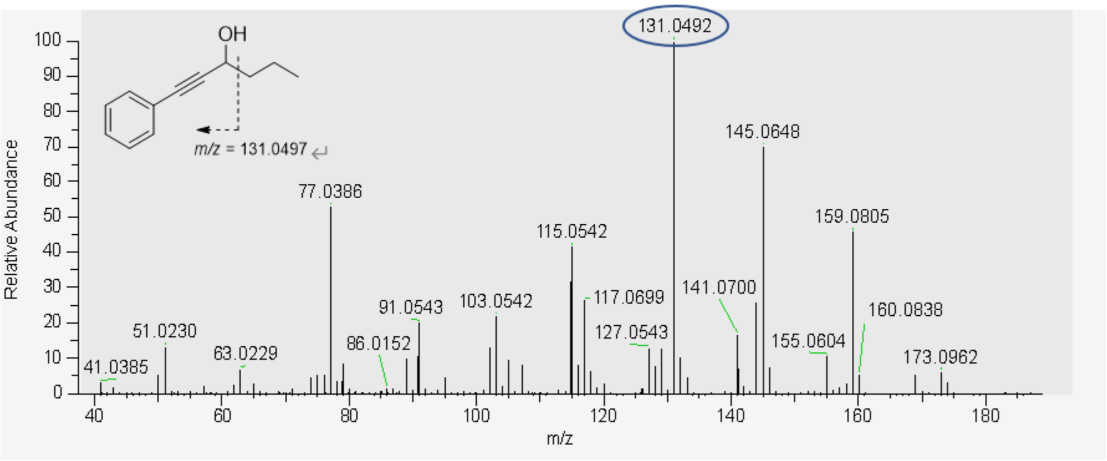

Figure S58. GC–MS chromatogram of **22a**.

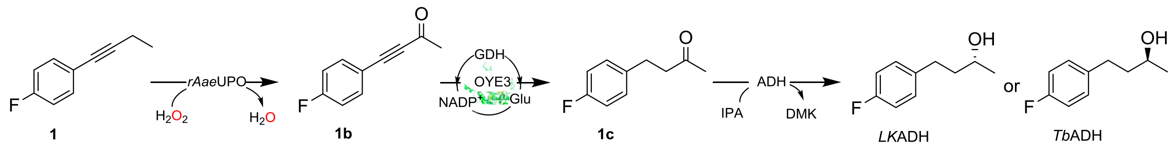

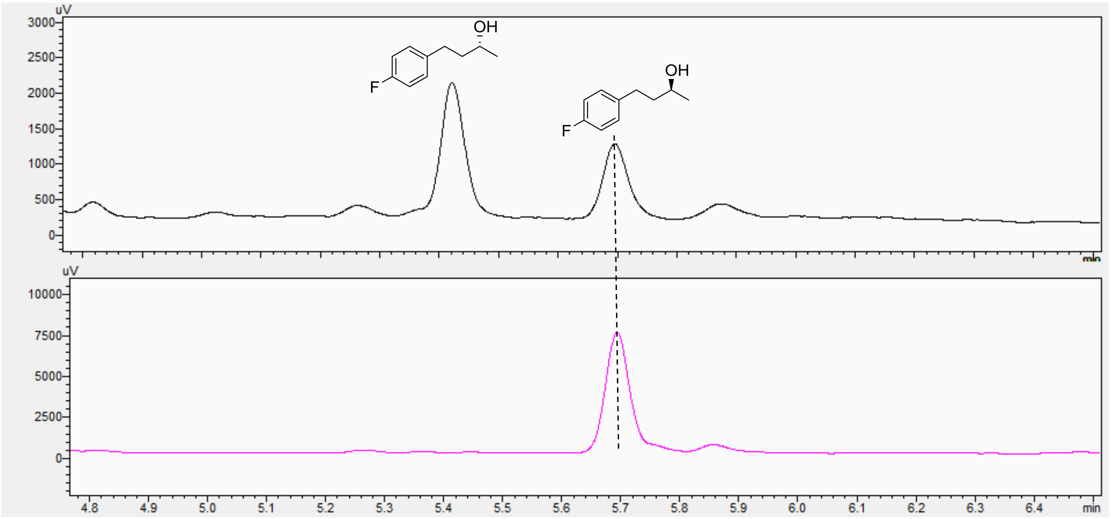

Figure S59. GC chromatogram of remote alcohol synthesis of r*Aae*UPO cascaded with OYE3 and ADH. The black line: reaction for r*Aae*UPO cascaded with OYE3 and *Lk*ADH. The pink line: reaction for r*Aae*UPO cascaded with OYE3 and *Tb*ADH. The retention times of (*S*)-4-(4-fluorophenyl)butan-2-ol and (*R*)- 4-(4-fluorophenyl)butan-2-ol were 5.422 and 5.695 min, respectively. **Sequential reaction conditions:** [**1a**] = 5 mM, [r*Aae*UPO] = 500 nM, $[H_2O_2]_{final}$ = 15 mM added at 3 mM h^{-1}, 30% (v/v) MeCN, NaPi buffer (100 mM, pH = 7), 800 rpm, 30 °C, 1 mL, 5 h. After the reaction, [OYE3] = 25 µM, [Glu] = 50 mM, [GDH] = 1 mg mL^{-1}, and $[NADP^+]$ = 2 mM were added to the above reaction mixture and reacted for 24 h. Then, [*Lk*ADH] = 50 µM, or [*Tb*ADH] = 25 µM, 50 µL isopropanol, [lysozyme] = 1 mg mL^{-1}, and [Dnase I] = 6 U mL^{-1} were added to the reaction for 5 h. **Sample preparation:** After the reaction, the reaction mixture was extracted using ethyl acetate and dried over Na_2SO_4. Then, the samples were subjected to derivatization with acetic anhydride and reacted for 2 hours. The reaction was quenched by 0.1 M HCl and dried before analysis by GC equipped with a chiral CP7503 column (Column B).

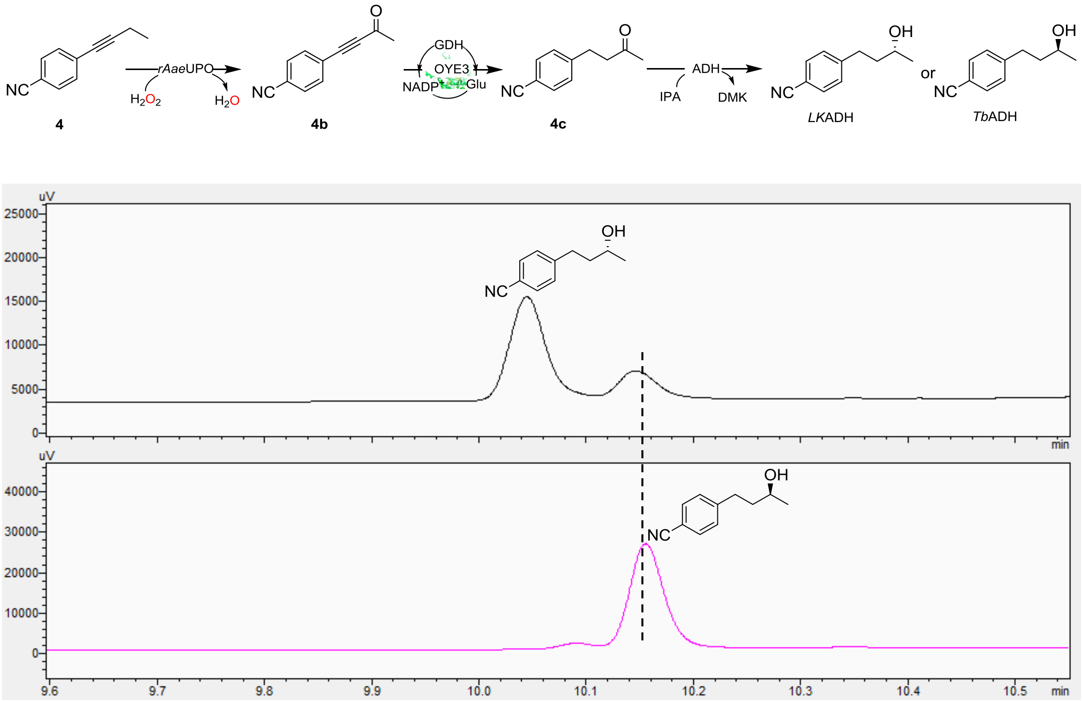

Figure S60. GC chromatogram of remote alcohol synthesis of r*Aae*UPO cascaded with OYE3 and ADH. The black line: reaction for r*Aae*UPO cascaded with OYE3 and *Lk*ADH. The pink line: reaction for r*Aae*UPO cascaded with OYE3 and *Tb*ADH. The retention times of (*S*)-4-(3-hydroxybutyl) benzonitrile and (*R*)-4-(3-hydroxybutyl)benzonitrile were 10.045 and 10.155 min, respectively. **Sequential reaction conditions:** [**4a**] = 5 mM, [r*Aae*UPO] = 500 nM, $[H_2O_2]_{final}$ = 15 mM added at 3 mM h^{-1}, 30% (v/v) MeCN, NaPi buffer (100 mM, pH = 7), 800 rpm, 30 °C, 1 mL, 5 h. After the reaction, [OYE3] = 25 µM, [Glu] = 50 mM, [GDH] = 1 mg mL^{-1}, and $[NADP^+]$ = 2 mM were added to the above reaction mixture and reacted for 24 h. Then, [*Lk*ADH] = 50 µM, or [*Tb*ADH] = 25 µM, 50 µL isopropanol, [lysozyme] = 1 mg mL^{-1}, and [Dnase I] = 6 U mL^{-1} were added to the reaction for 5 h. **Sample preparation:** After the reaction, the reaction mixture was extracted using ethyl acetate and dried over Na_2SO_4. Then, the samples were subjected to derivatization with acetic anhydride and reacted for 2 hours. The reaction was quenched by 0.1 M HCl and dried before analysis by GC equipped with a chiral CP7503 column (Column B).

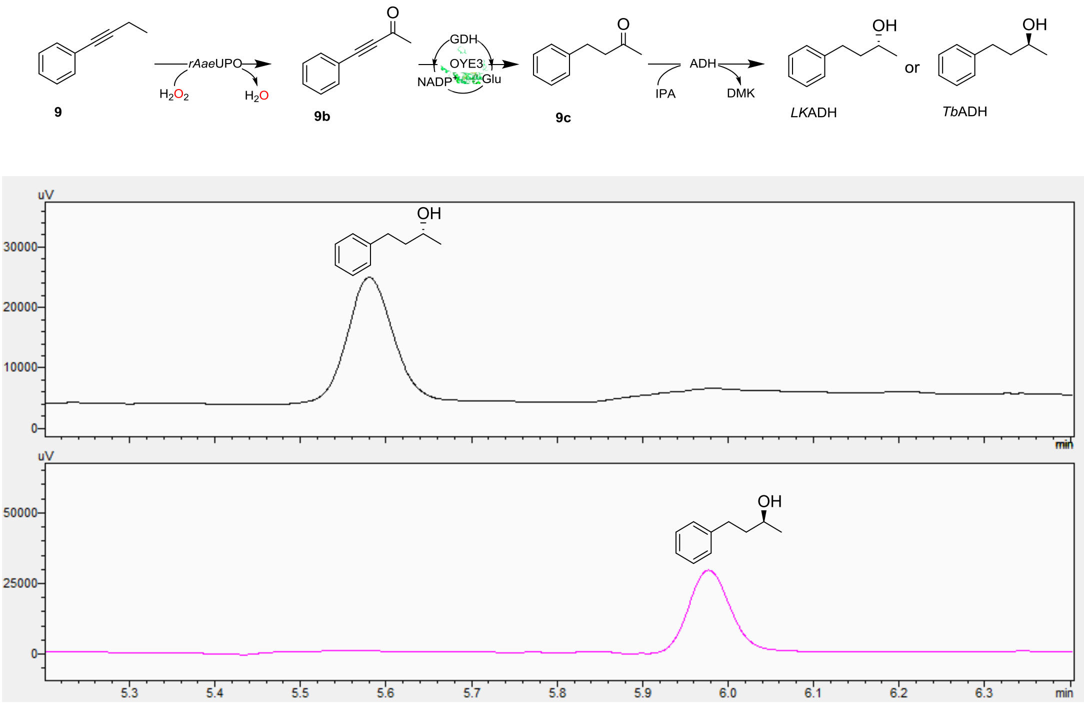

Figure S61. GC chromatogram of remote alcohol synthesis of r*Aae*UPO cascaded with OYE3 and ADH. The black line: reaction for r*Aae*UPO cascaded with OYE3 and *Lk*ADH. The pink line: reaction for r*Aae*UPO cascaded with OYE3 and *Tb*ADH. The retention times of (*S*)-4-phenylbutan-2-ol and (*R*)-4-phenylbutan-2-ol were 5.576 and 5.976 min, respectively. **Sequential reaction conditions:** [**9a**] = 5 mM, [r*Aae*UPO] = 500 nM, $[H_2O_2]_{final}$ = 15 mM added at 3 mM h^{-1}, 30% (v/v) MeCN, NaPi buffer (100 mM, pH = 7), 800 rpm, 30 °C, 1 mL, 5 h. After the reaction, [OYE3] = 25 µM, [Glu] = 50 mM, [GDH] = 1 mg mL^{-1}, and $[NADP^+]$ = 2 mM were added to the above reaction mixture and reacted for 24 h. Then, [*Lk*ADH] = 50 µM, or [*Tb*ADH] = 25 µM, 50 µL isopropanol, [lysozyme] = 1 mg mL^{-1}, and [Dnase I] = 6 U mL^{-1} were added to the reaction for 5 h. **Sample preparation:** After the reaction, the reaction mixture was extracted using ethyl acetate and dried over Na_2SO_4. Then, the samples were subjected to derivatization with acetic anhydride and reacted for 2 hours. The reaction was quenched by 0.1 M HCl and dried before analysis by GC equipped with a chiral CP7503 column (Column B).

NMR spectrum

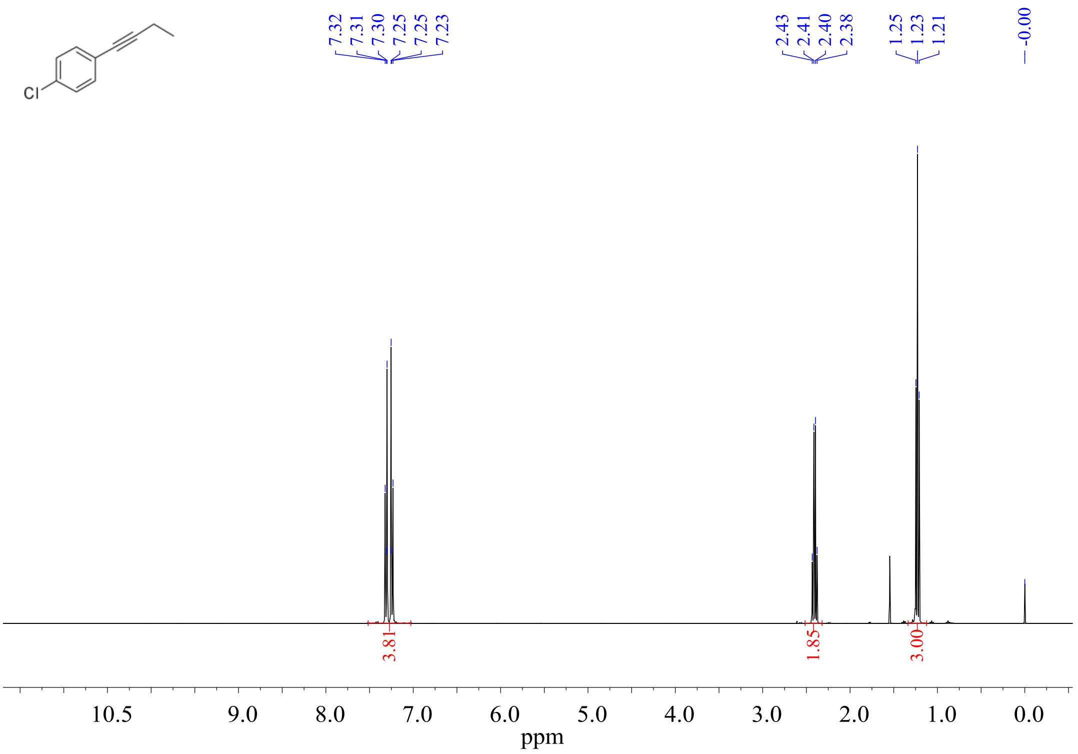

Figure S62. ^{1}H NMR of **2** synthesized in-house (in $CDCl_3$).

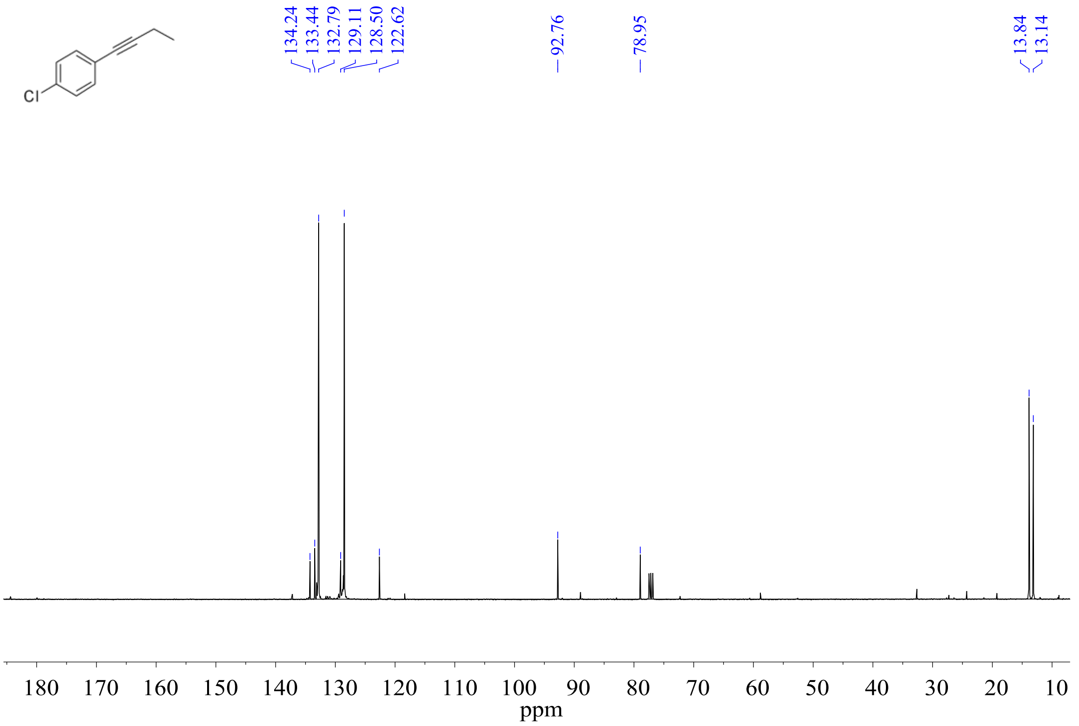

Figure S63. ^{13}C NMR of **2** synthesized in-house (in $CDCl_3$).

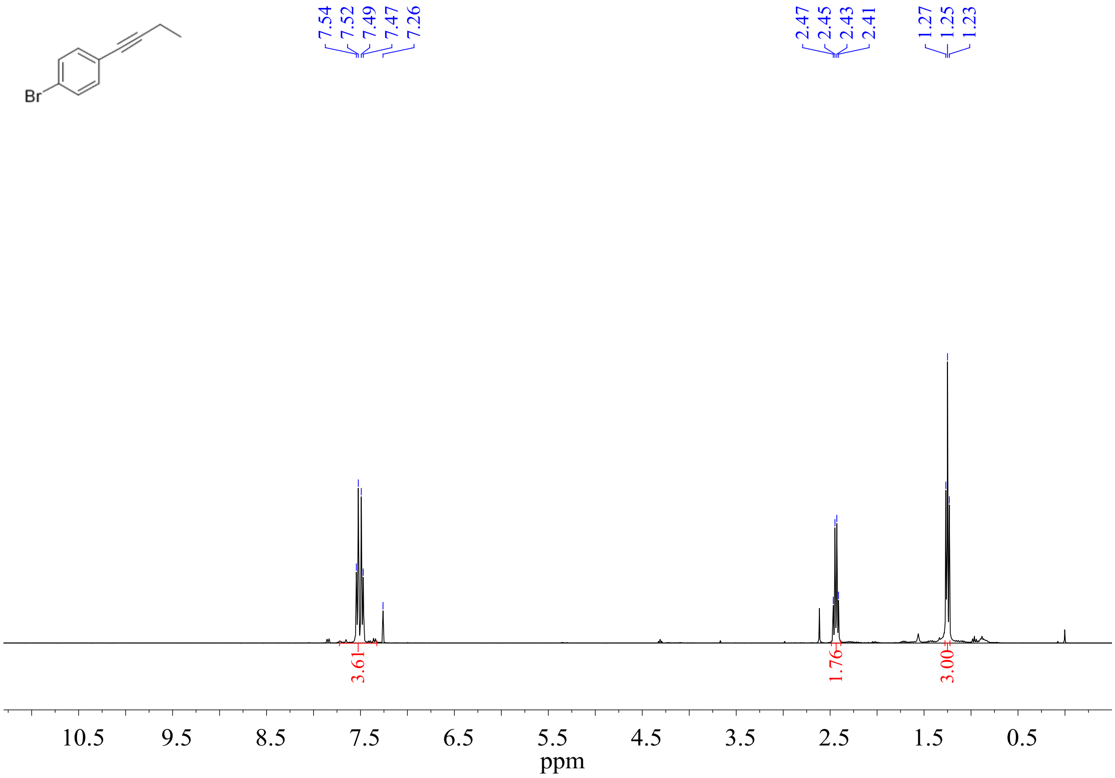

Figure S64. ^{1}H NMR of **3** synthesized in-house (in $CDCl_3$).

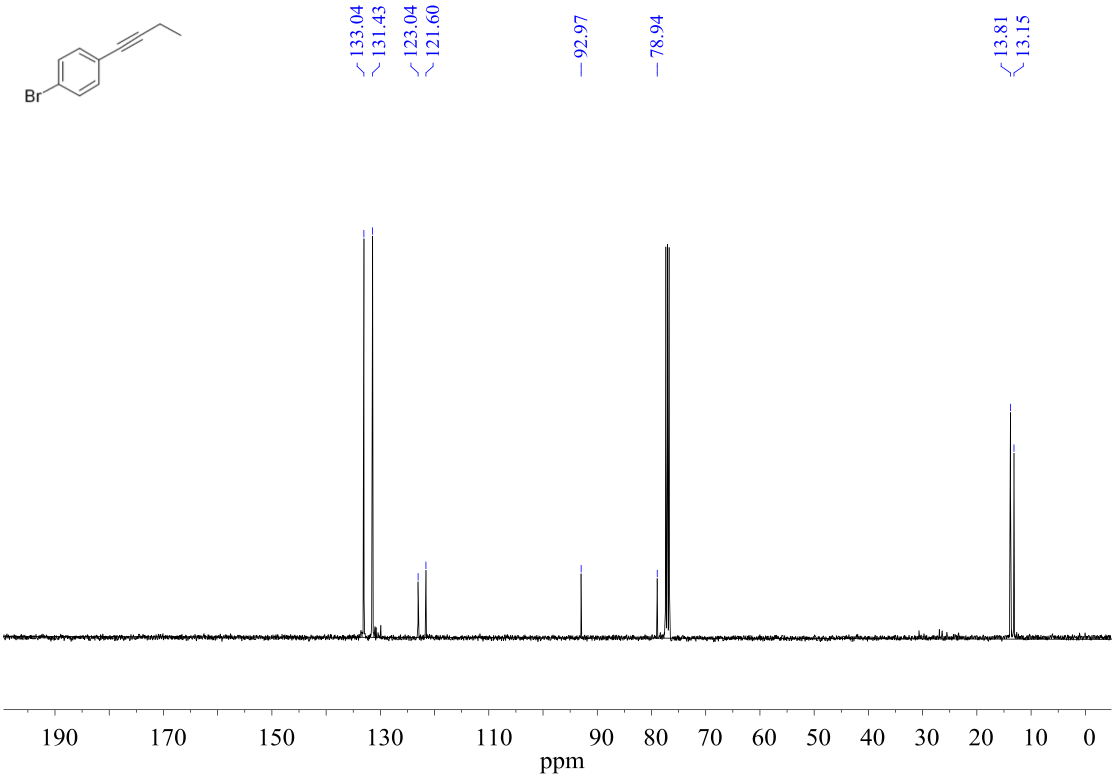

Figure S65. ^{13}C NMR of **3** synthesized in-house (in $CDCl_3$).

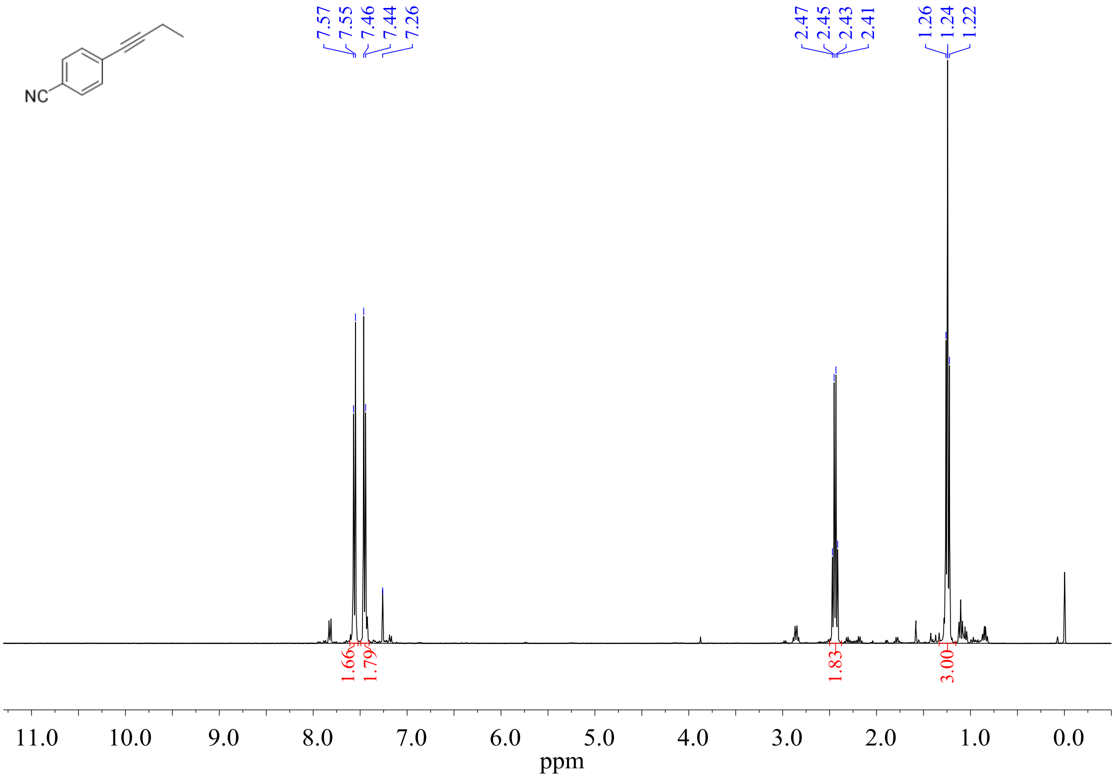

Figure S66. ^{1}H NMR of **4** synthesized in-house (in $CDCl_3$).

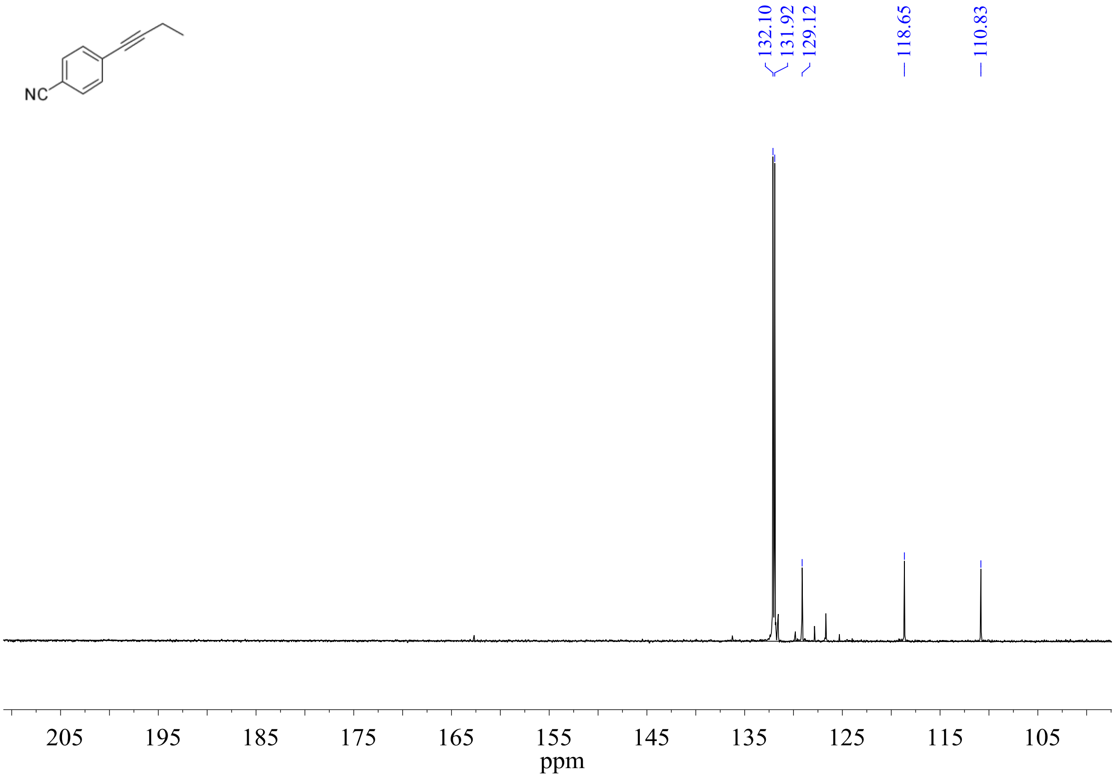

Figure S67. ^{13}C NMR of **4** synthesized in-house (in $CDCl_3$).

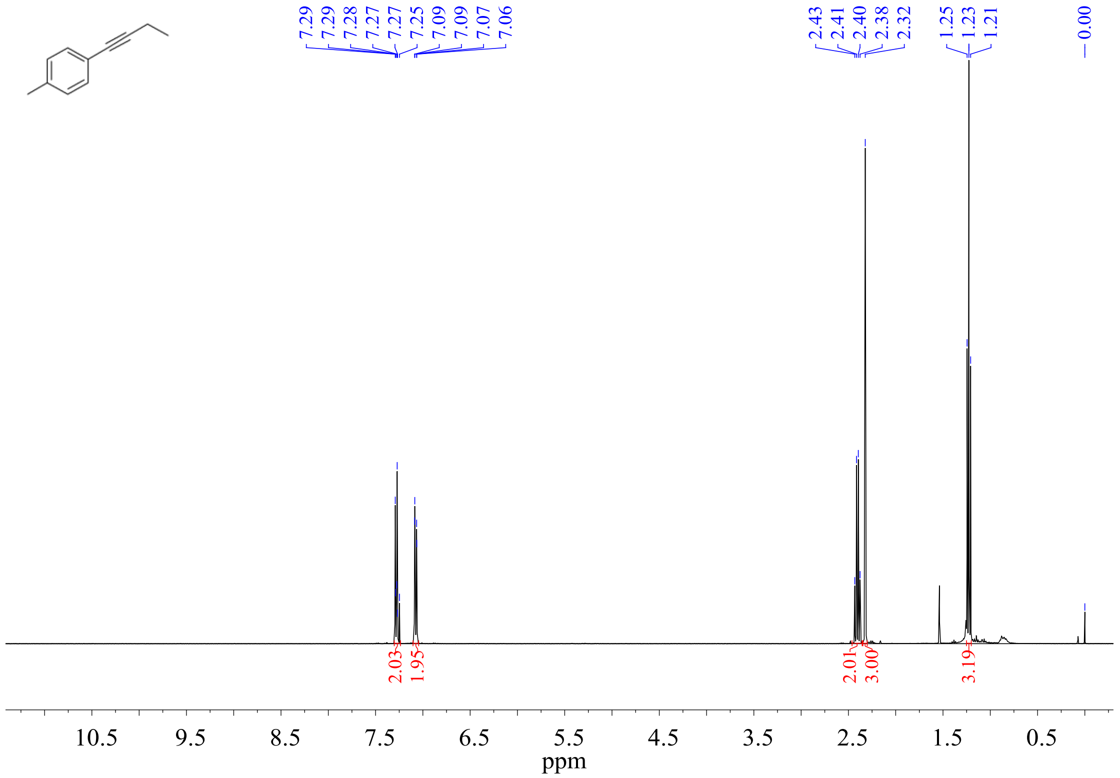

Figure S68. ^{1}H NMR of **6** synthesized in-house (in $CDCl_3$).

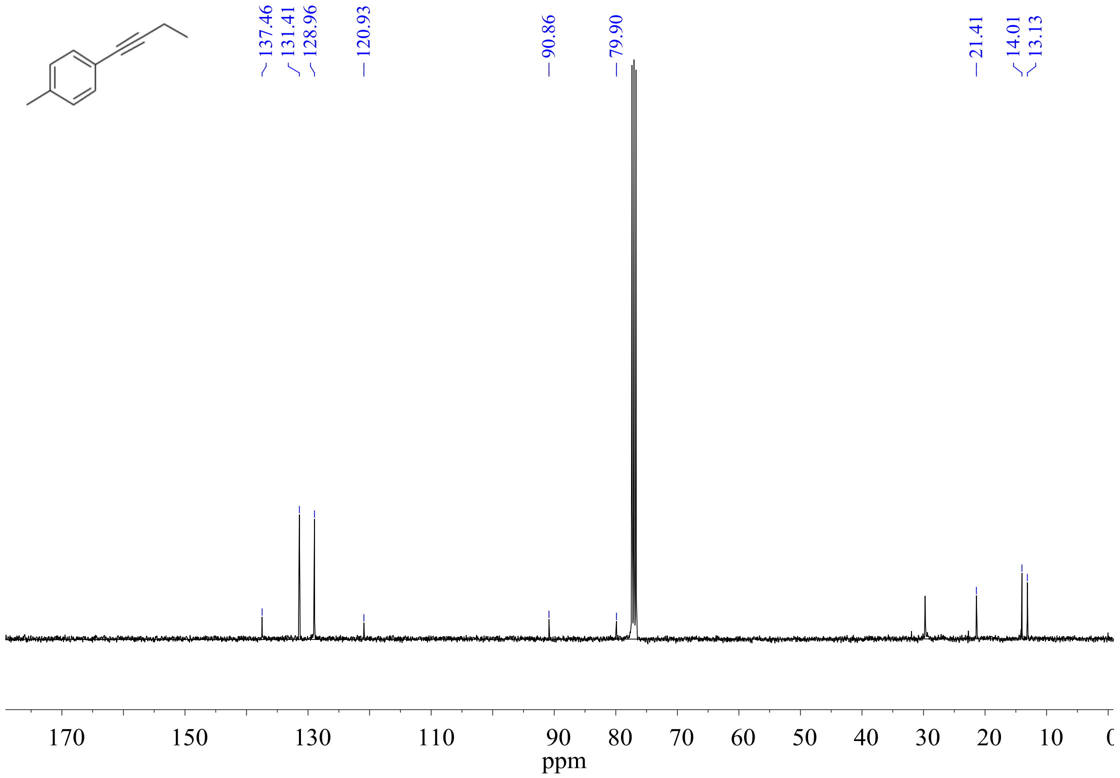

Figure S69. ^{13}C NMR of **6** synthesized in-house (in $CDCl_3$).

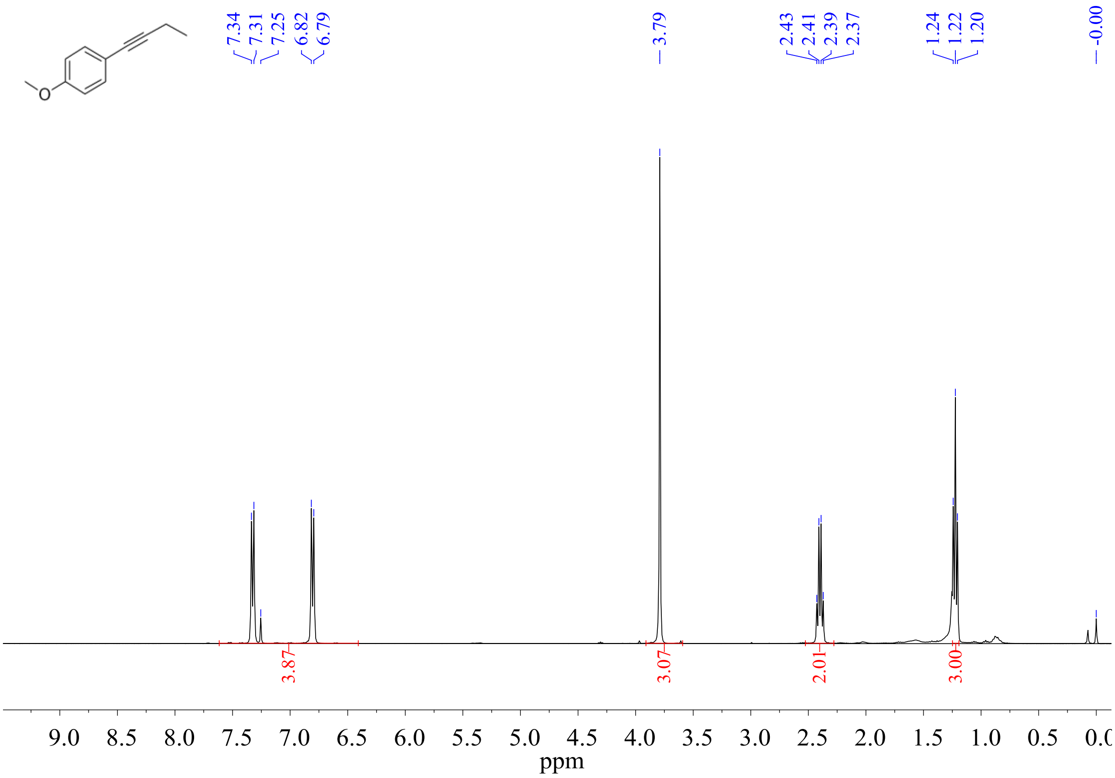

Figure S70. ^{1}H NMR of **7** synthesized in-house (in $CDCl_3$).

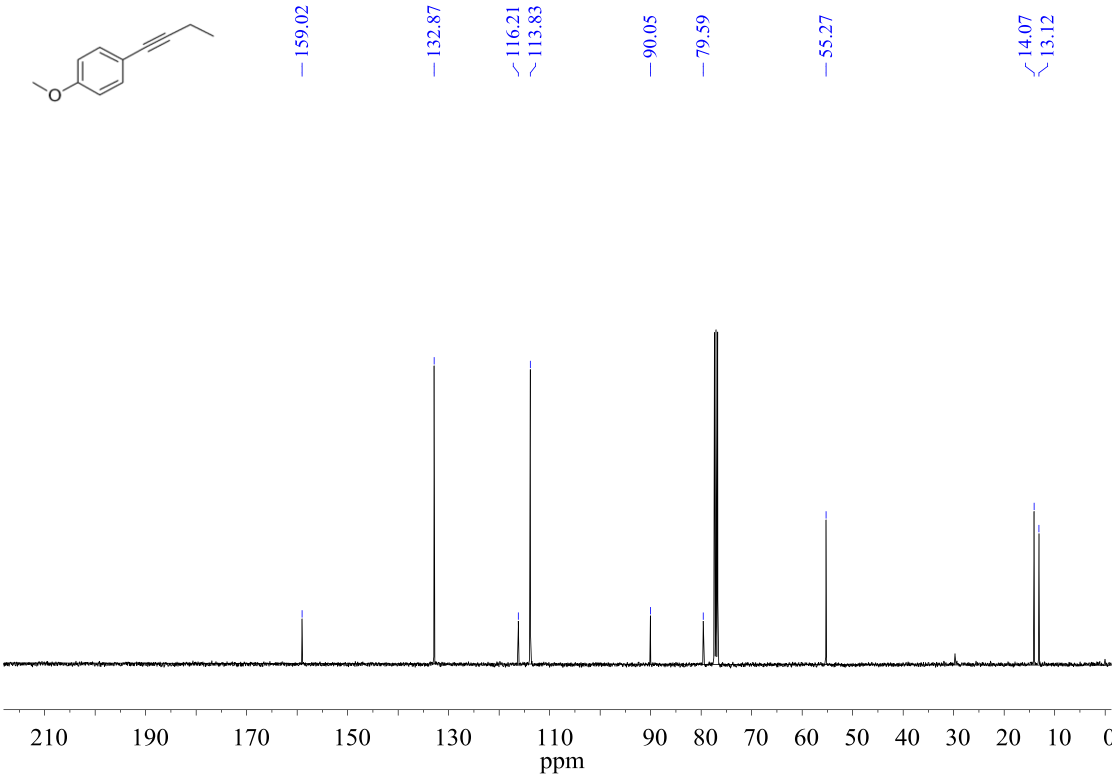

Figure S71. ^{13}C NMR of **7** synthesized in-house (in $CDCl_3$).

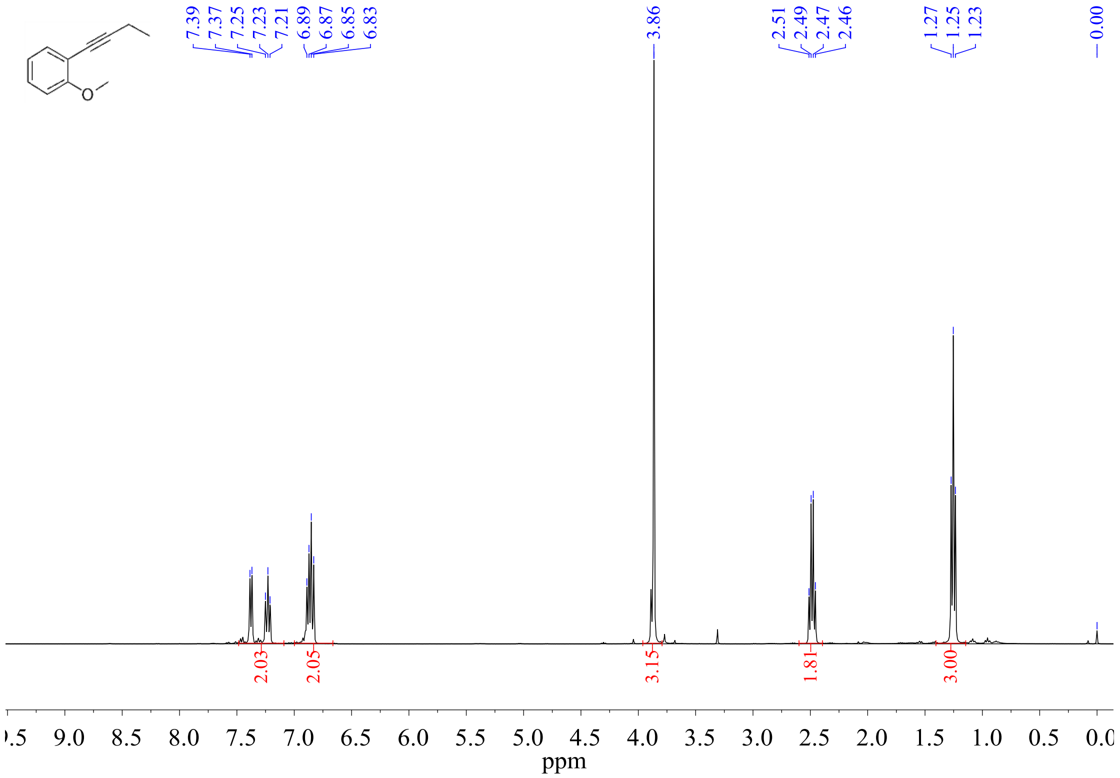

Figure S72. ^{1}H NMR of **8** synthesized in-house (in $CDCl_3$).

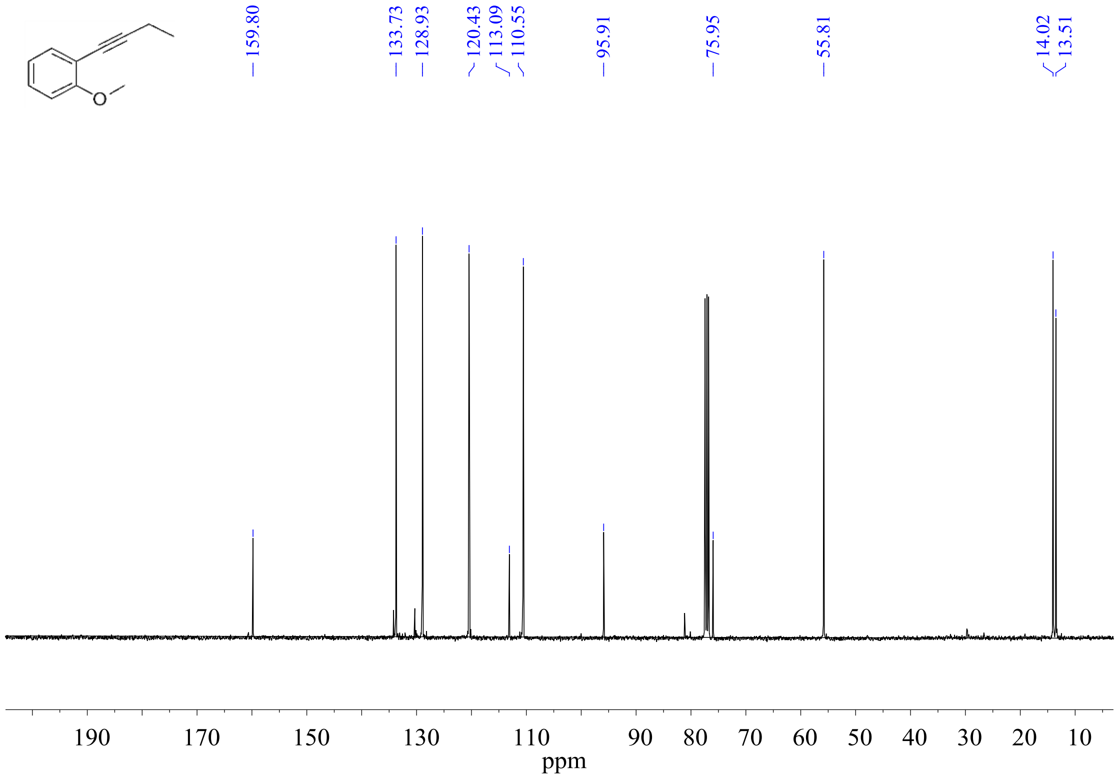

Figure S73. ^{13}C NMR of **8** synthesized in-house (in $CDCl_3$).

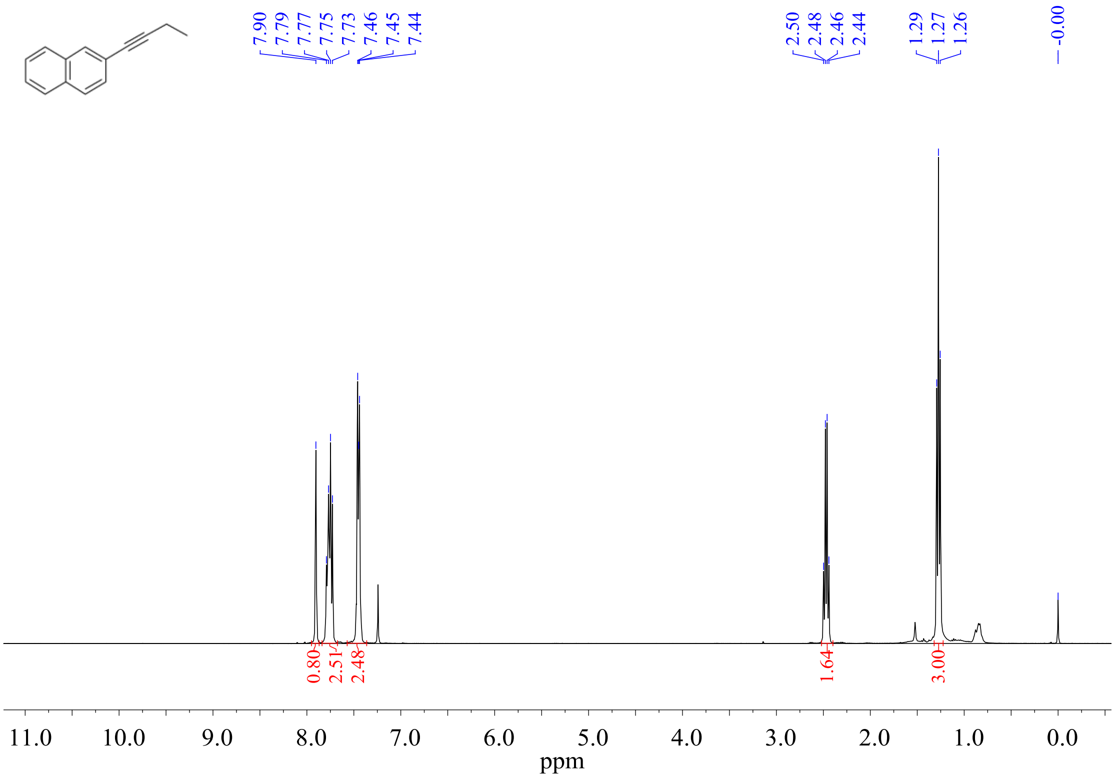

Figure S74. ^{1}H NMR of **10** synthesized in-house (in $CDCl_3$).

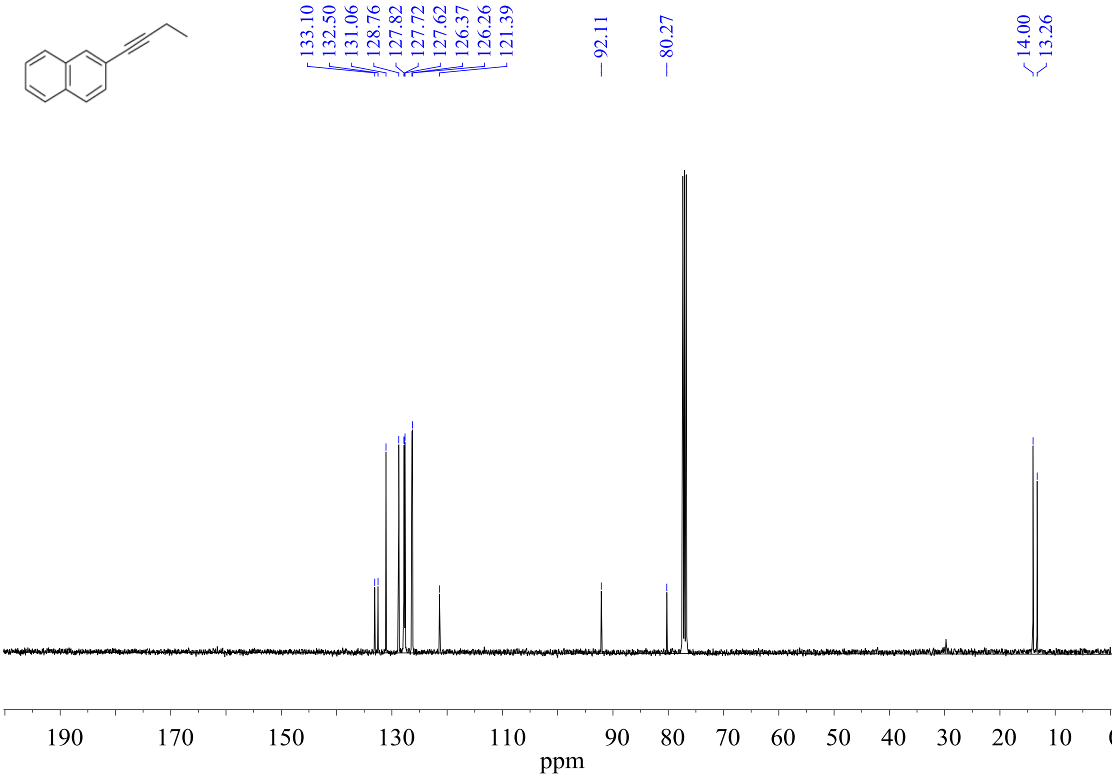

Figure S75. ^{13}C NMR of **10** synthesized in-house (in $CDCl_3$).

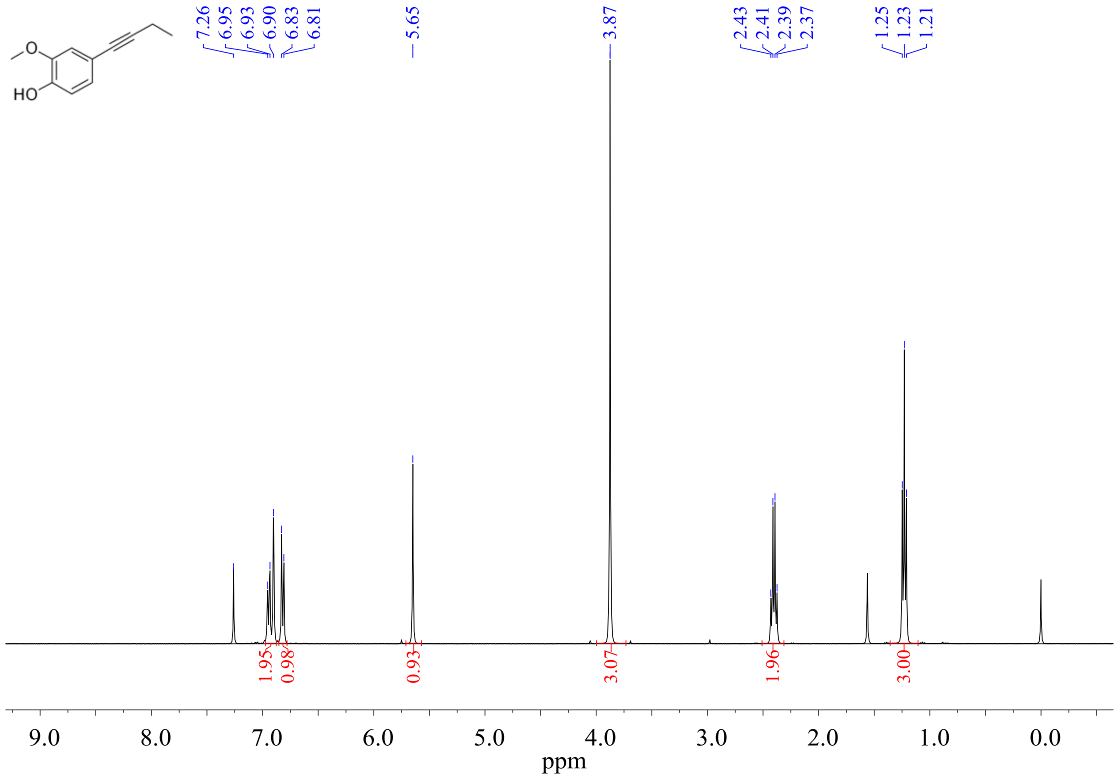

Figure S76. ^{1}H NMR of **11** synthesized in-house (in $CDCl_3$).

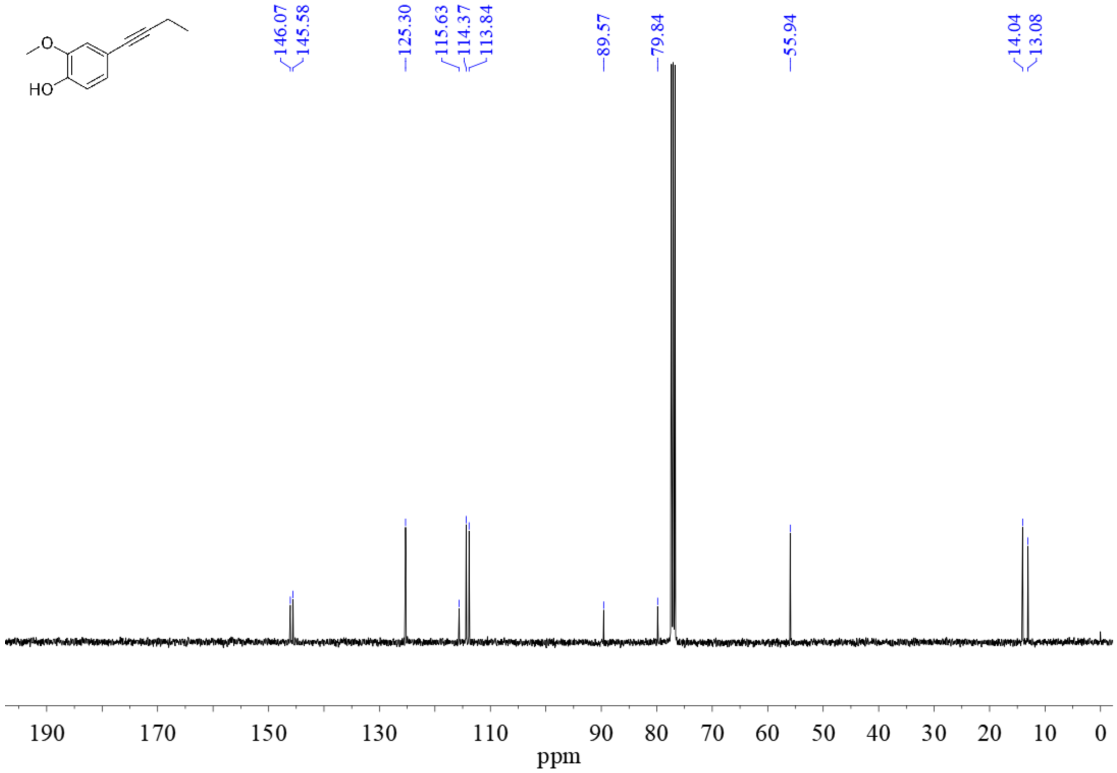

Figure S77. ^{13}C NMR of **11** synthesized in-house (in $CDCl_3$).

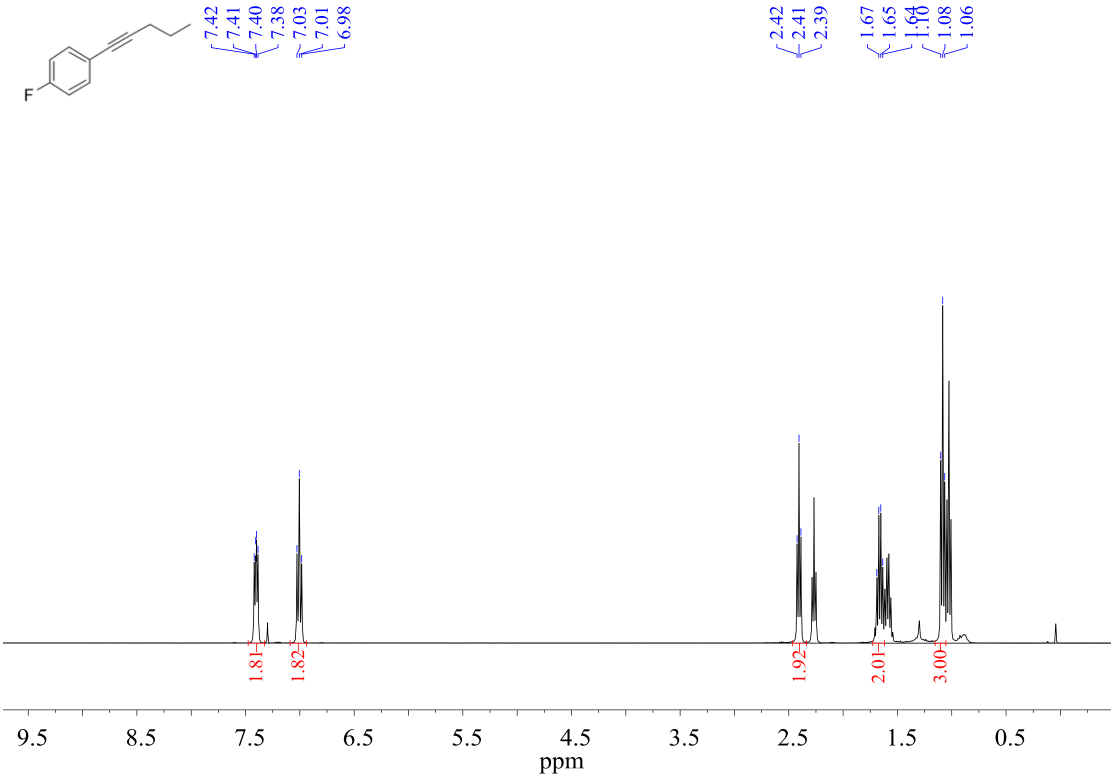

Figure S78. ^{1}H NMR of **12** synthesized in-house (in $CDCl_3$).

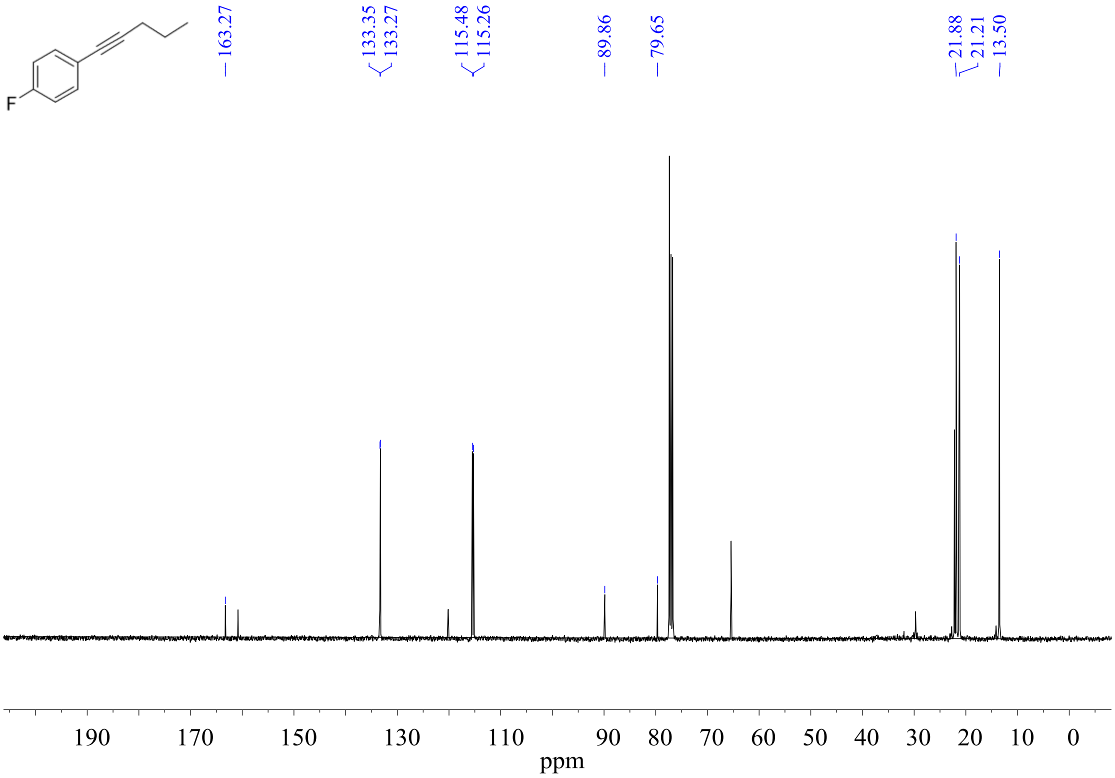

Figure S79. ^{13}C NMR of **12** synthesized in-house (in $CDCl_3$).

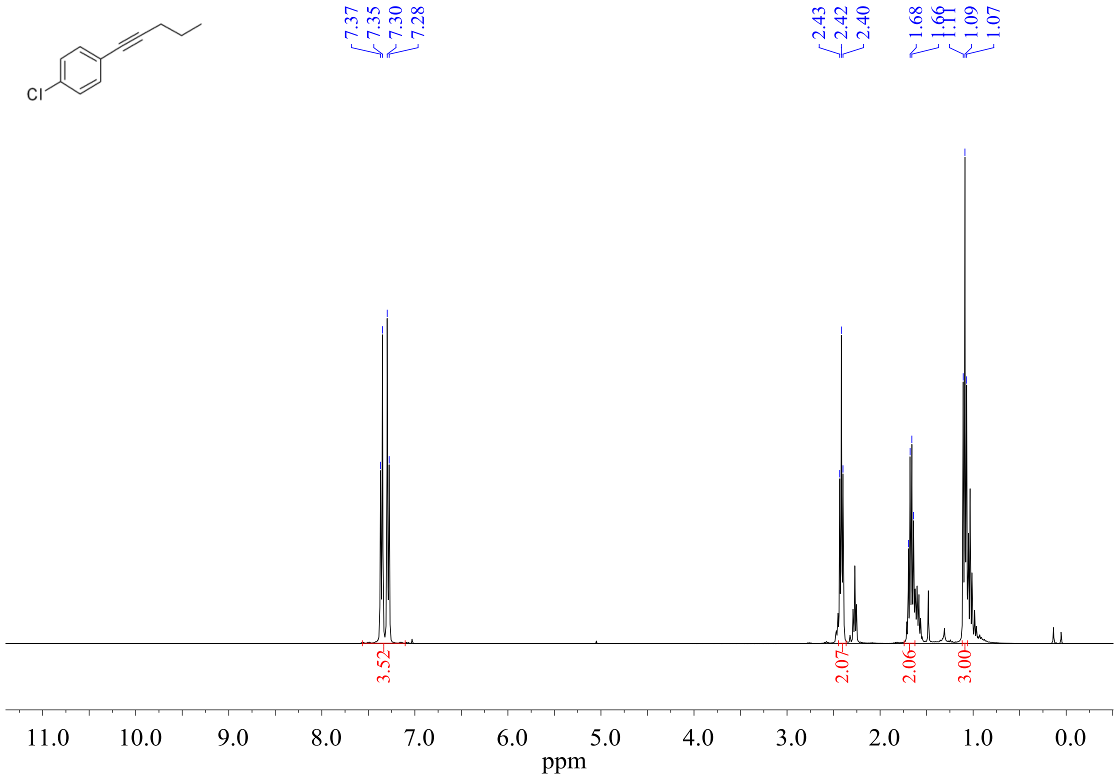

Figure S80. ^{1}H NMR of **13** synthesized in-house (in CCl$_3$).

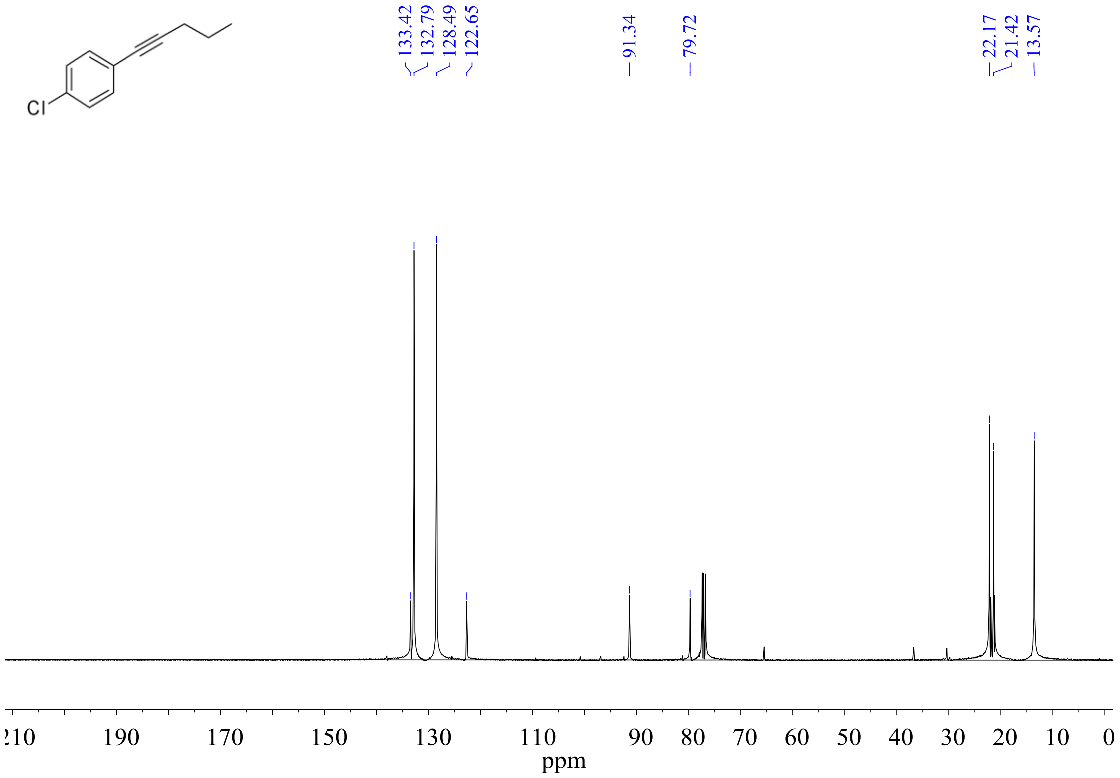

Figure S81. ^{13}C NMR of **13** synthesized in-house (in CDCl$_3$).

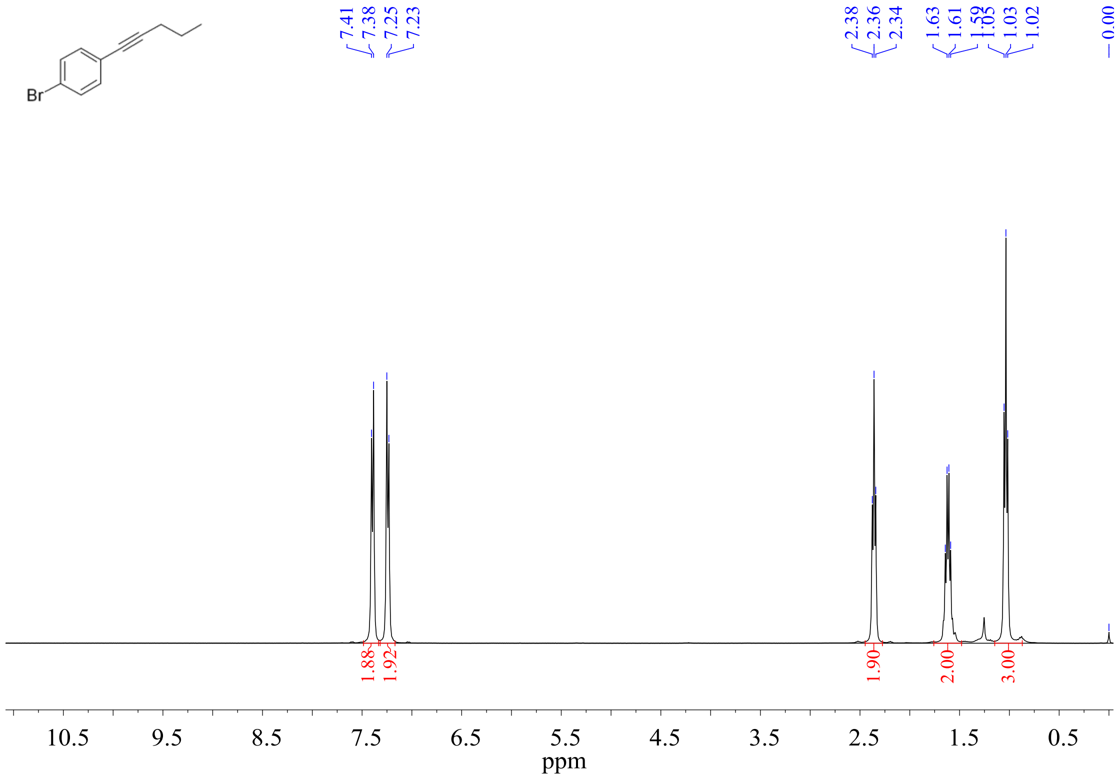

Figure S82. ^{1}H NMR of **14** synthesized in-house (in $CDCl_3$).

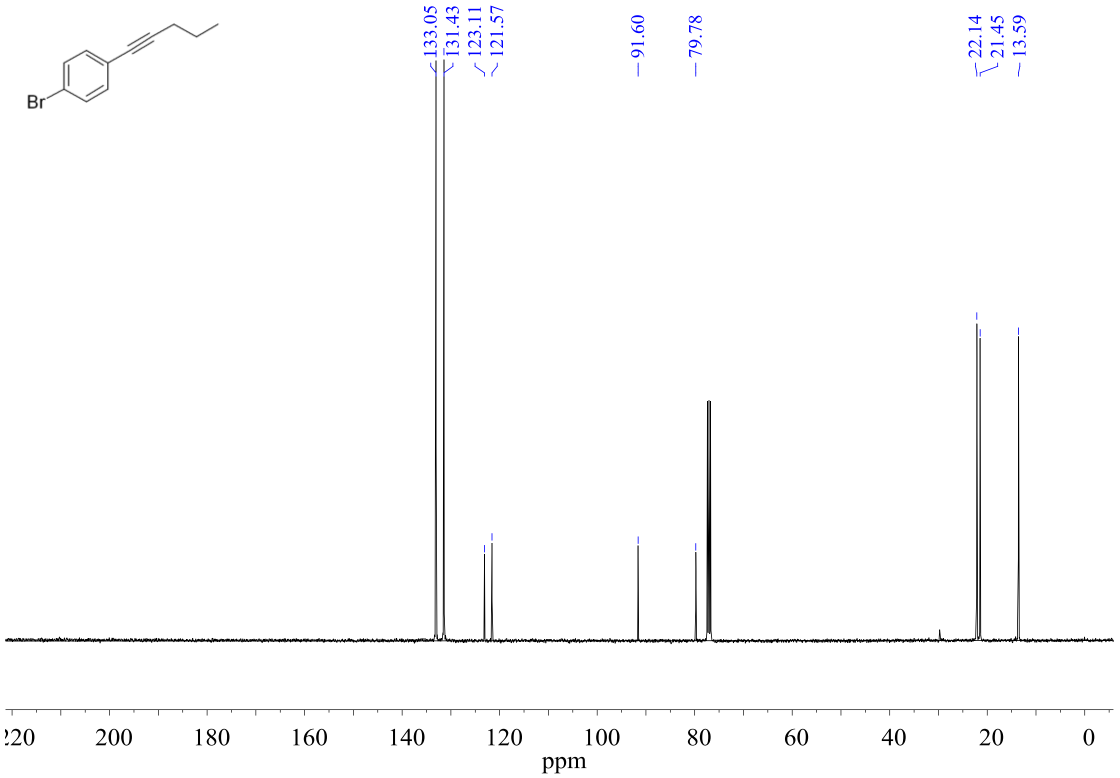

Figure S83. ^{13}C NMR of **14** synthesized in-house (in $CDCl_3$).

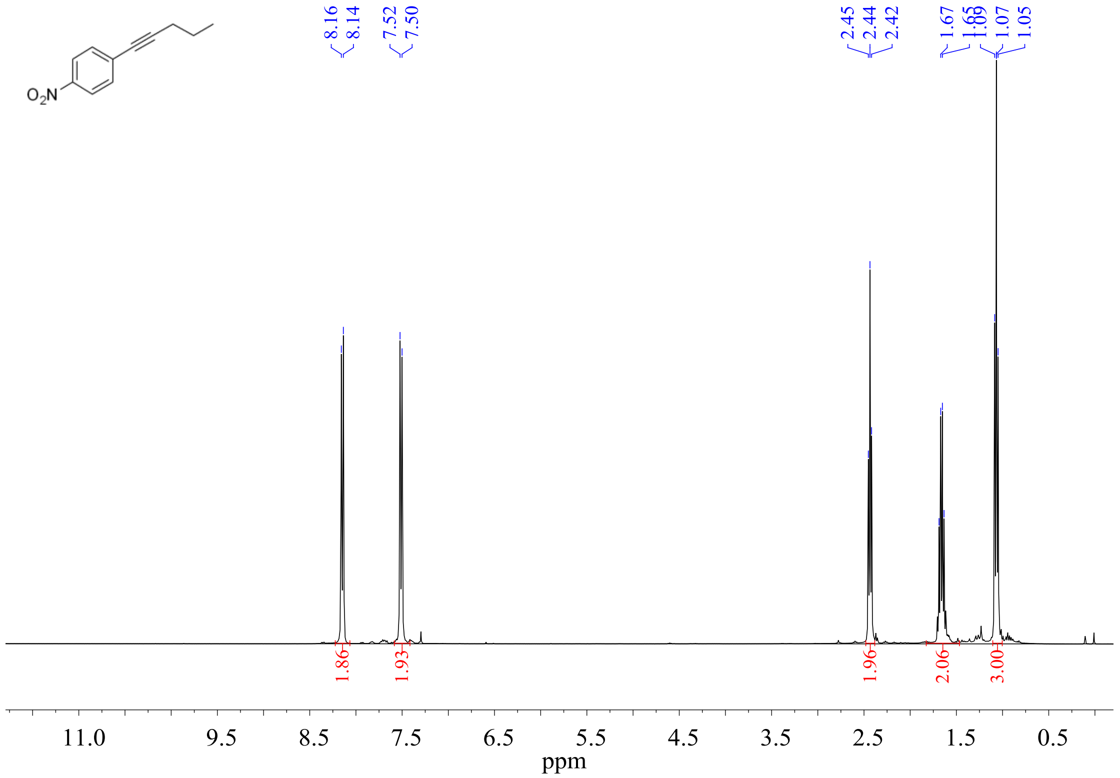

Figure S84. ^{1}H NMR of **15** synthesized in-house (in $CDCl_3$).

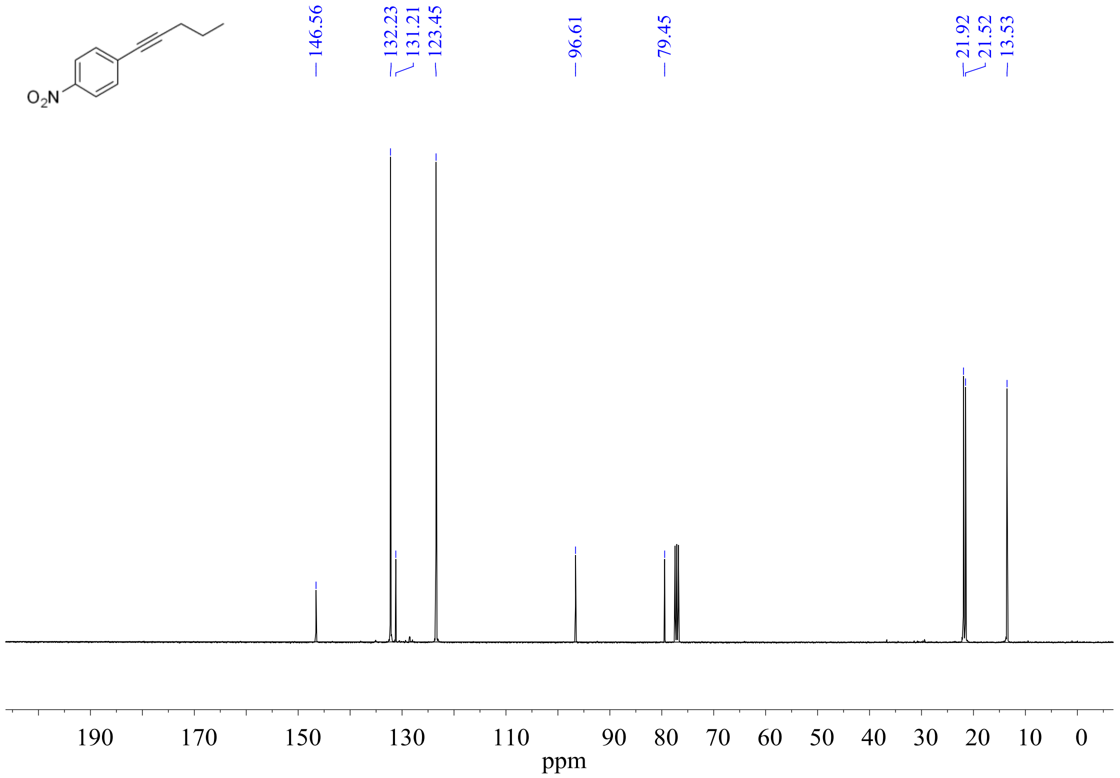

Figure S85. ^{13}C NMR of **15** synthesized in-house (in $CDCl_3$).

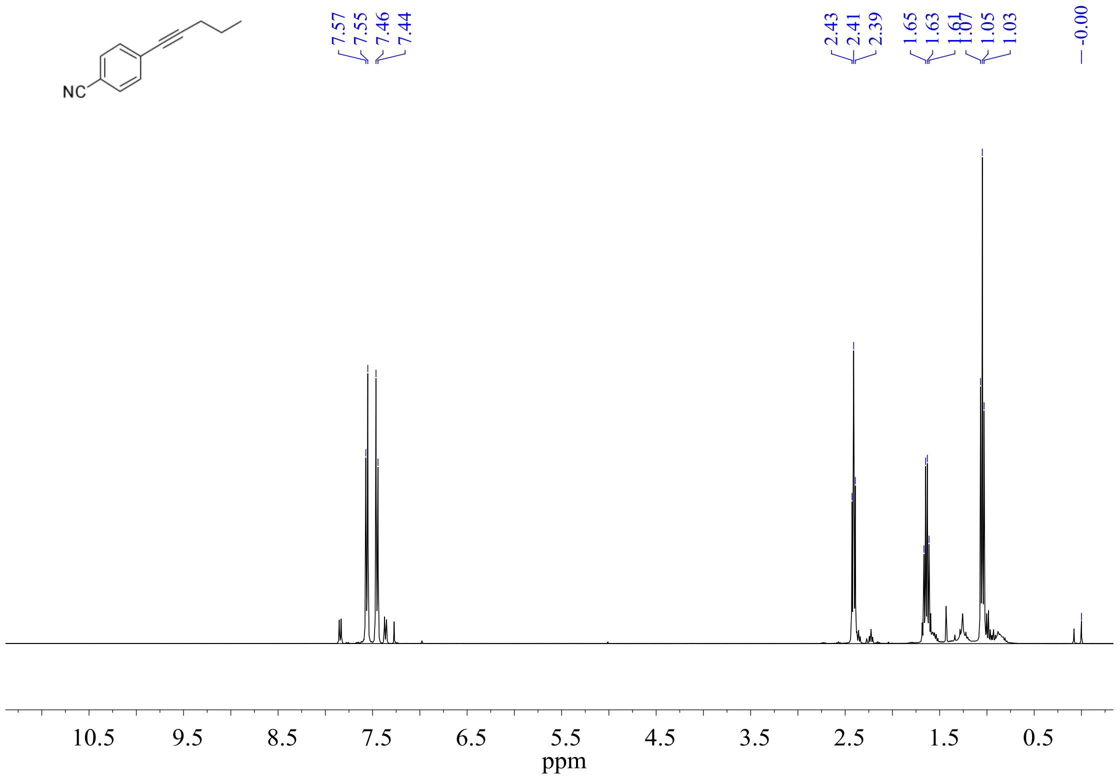

Figure S86. ^{1}H NMR of **16** synthesized in-house (in $CDCl_3$).

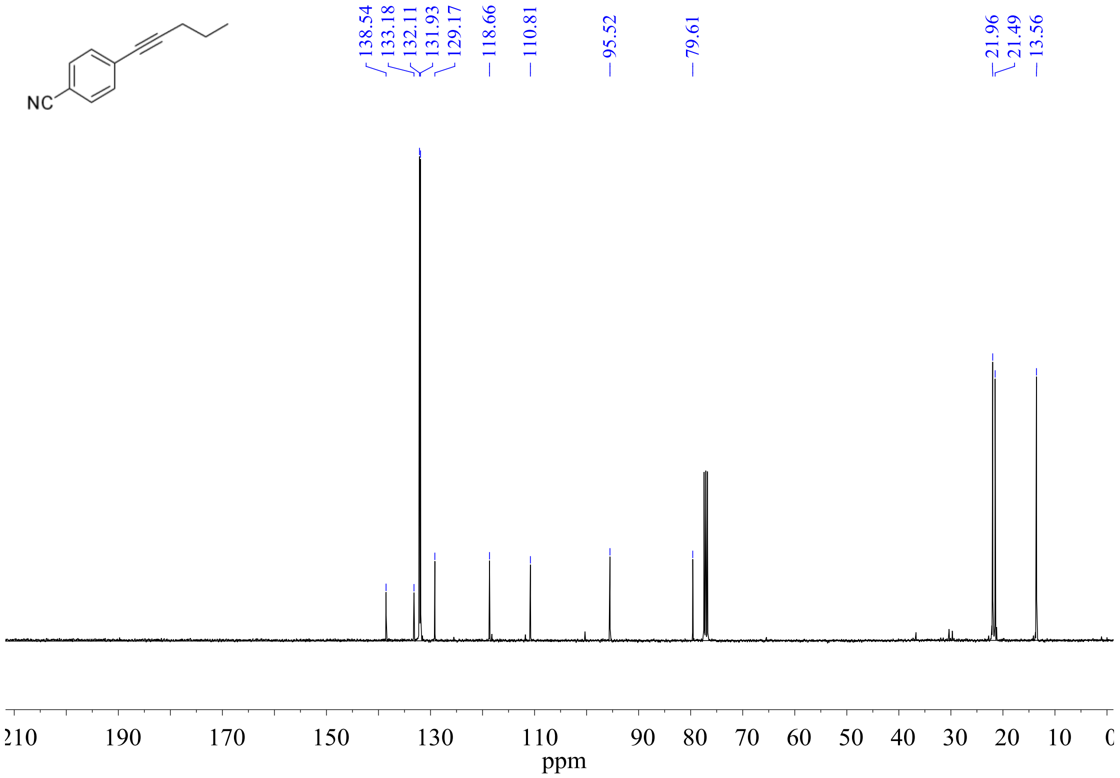

Figure S87. ^{13}C NMR of **16** synthesized in-house (in $CDCl_3$).

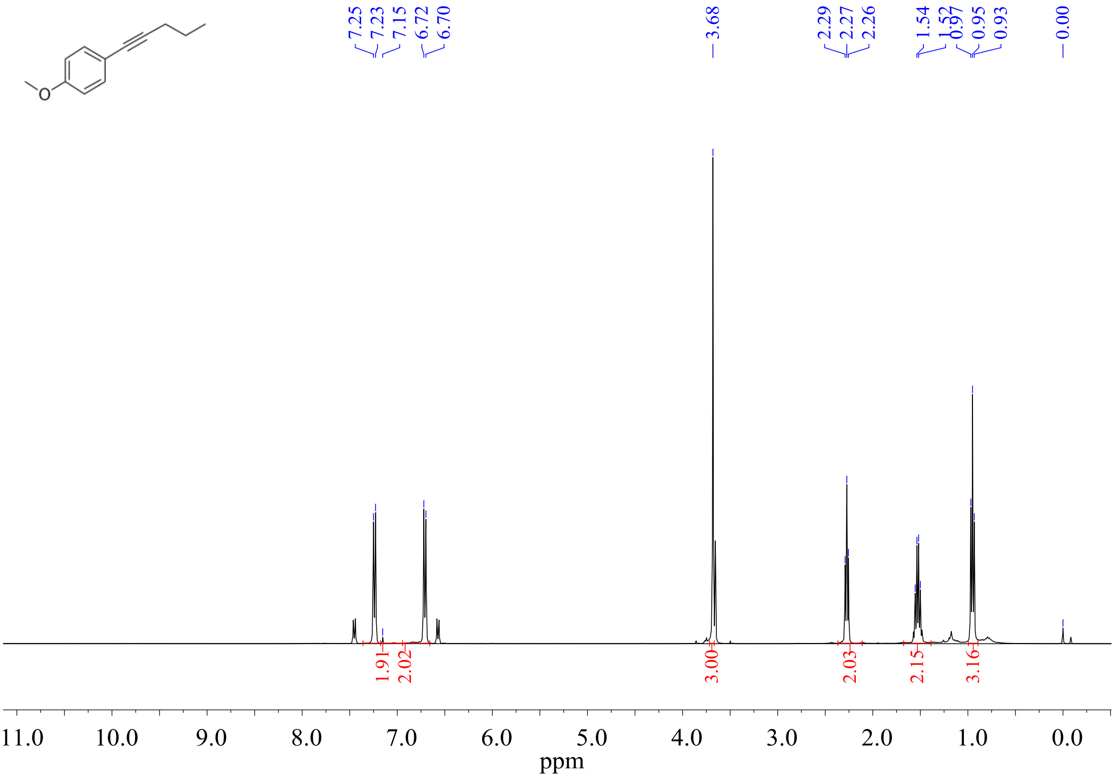

Figure S88. ^{1}H NMR of **17** synthesized in-house (in $CDCl_3$).

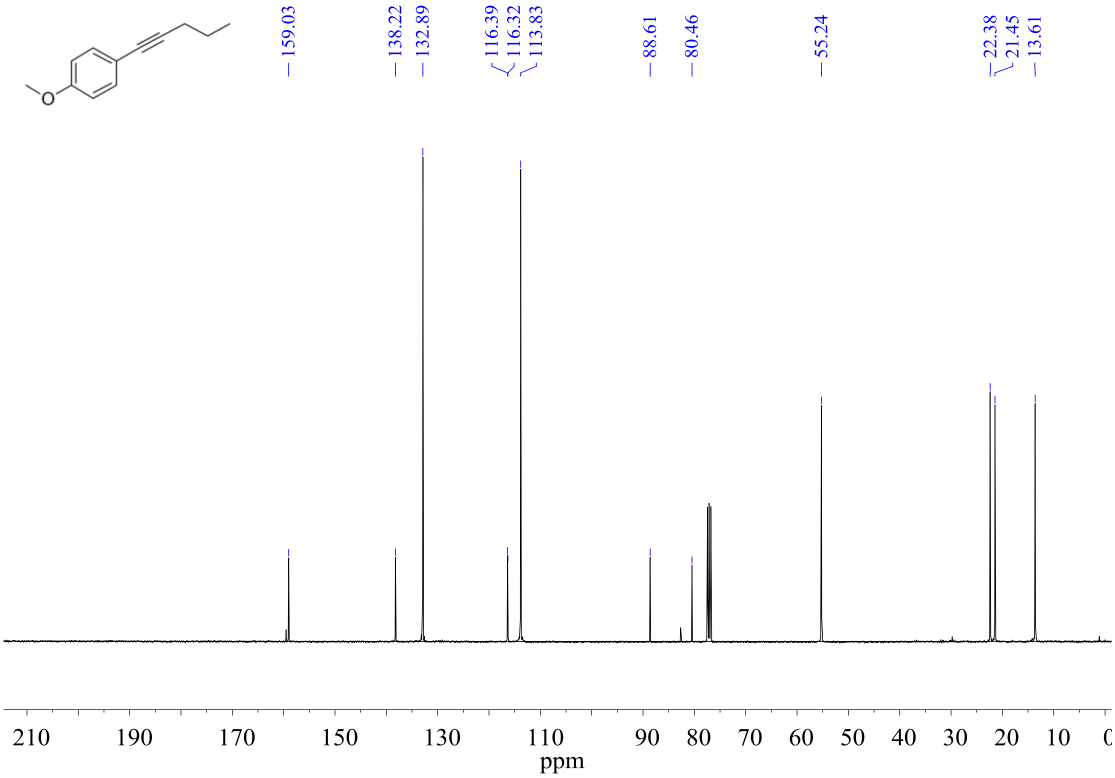

Figure S89. ^{13}C NMR of **17** synthesized in-house (in $CDCl_3$).

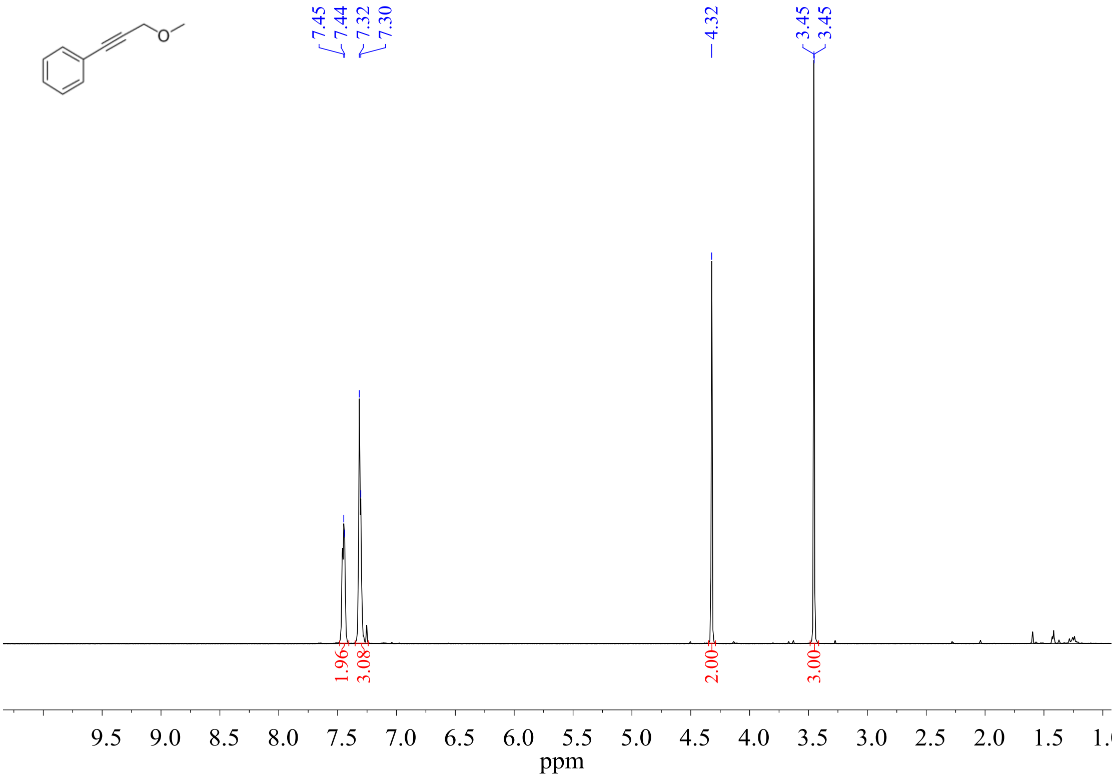

Figure S90. ^{1}H NMR of **18** synthesized in-house (in $CDCl_3$).

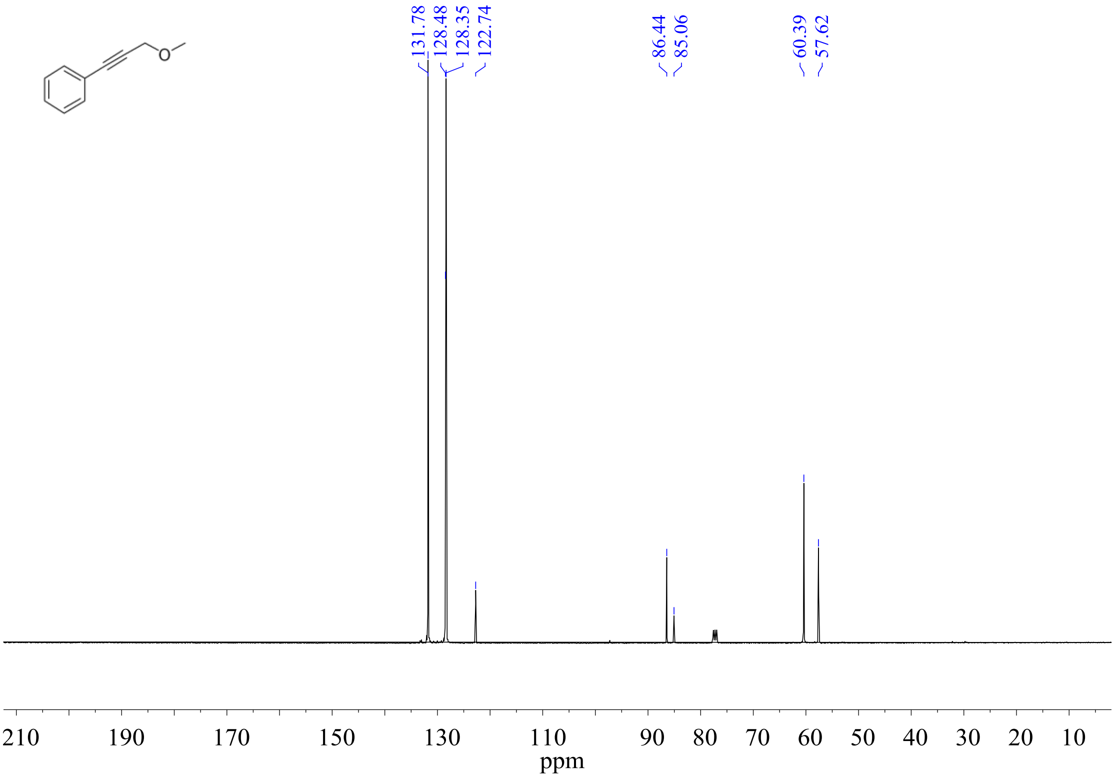

Figure S91. ^{13}C NMR of **18** synthesized in-house (in $CDCl_3$).

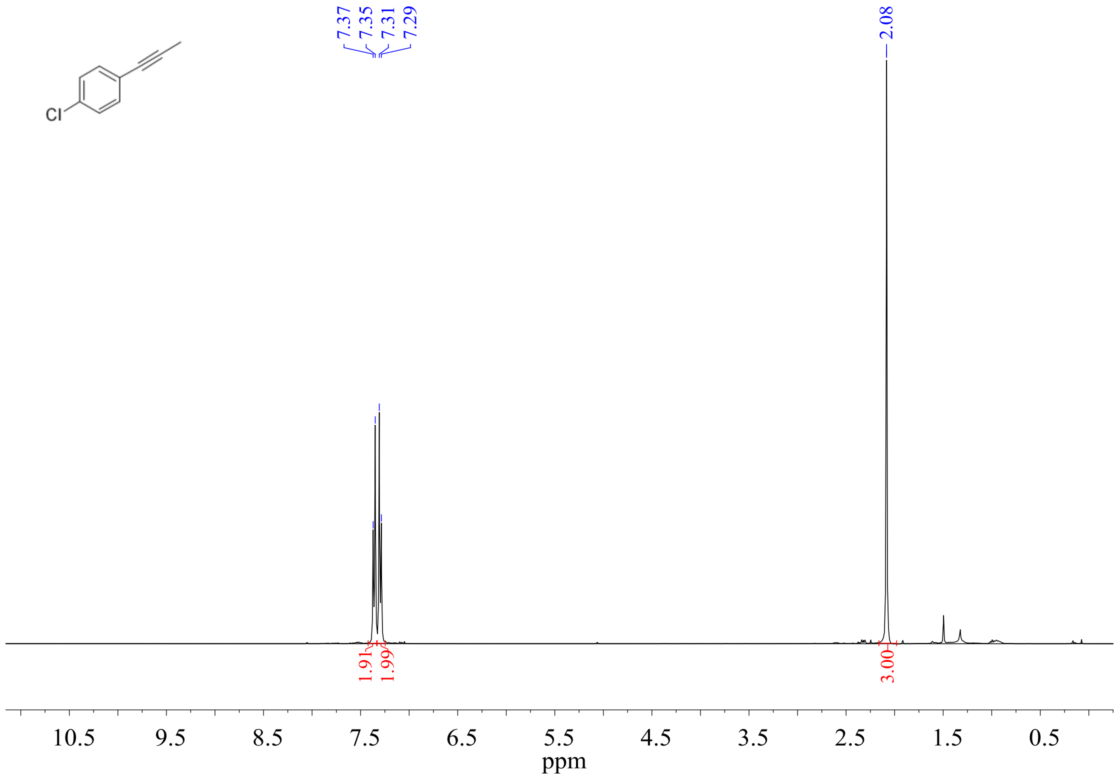

Figure S92. ^{1}H NMR of **19** synthesized in-house (in $CDCl_3$).

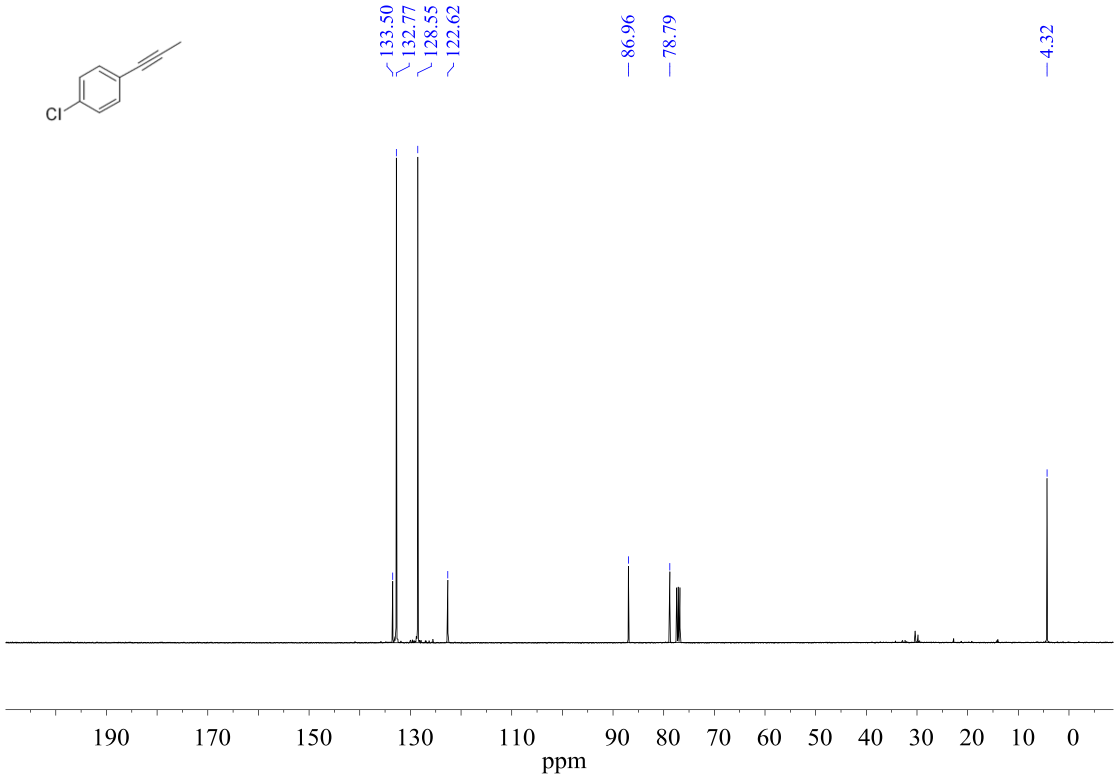

Figure S93. ^{13}C NMR of **19** synthesized in-house (in $CDCl_3$).

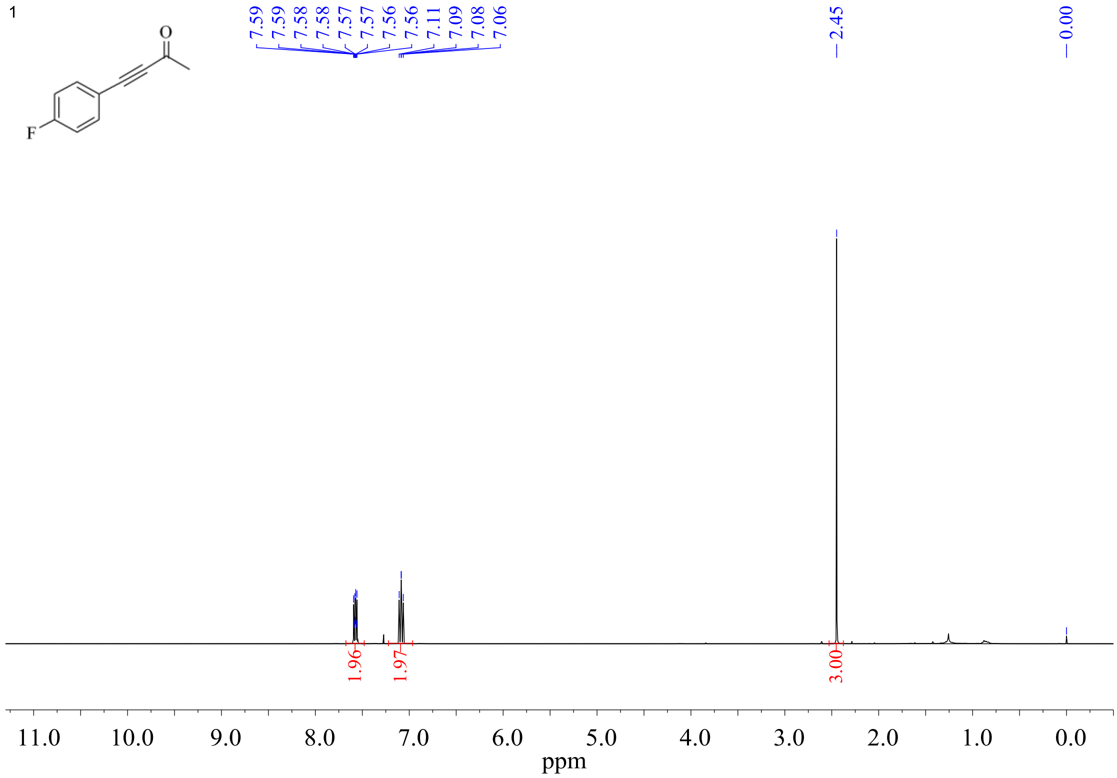

Figure S94. ^{1}H NMR of **1b** synthesized in-house (in $CDCl_3$).

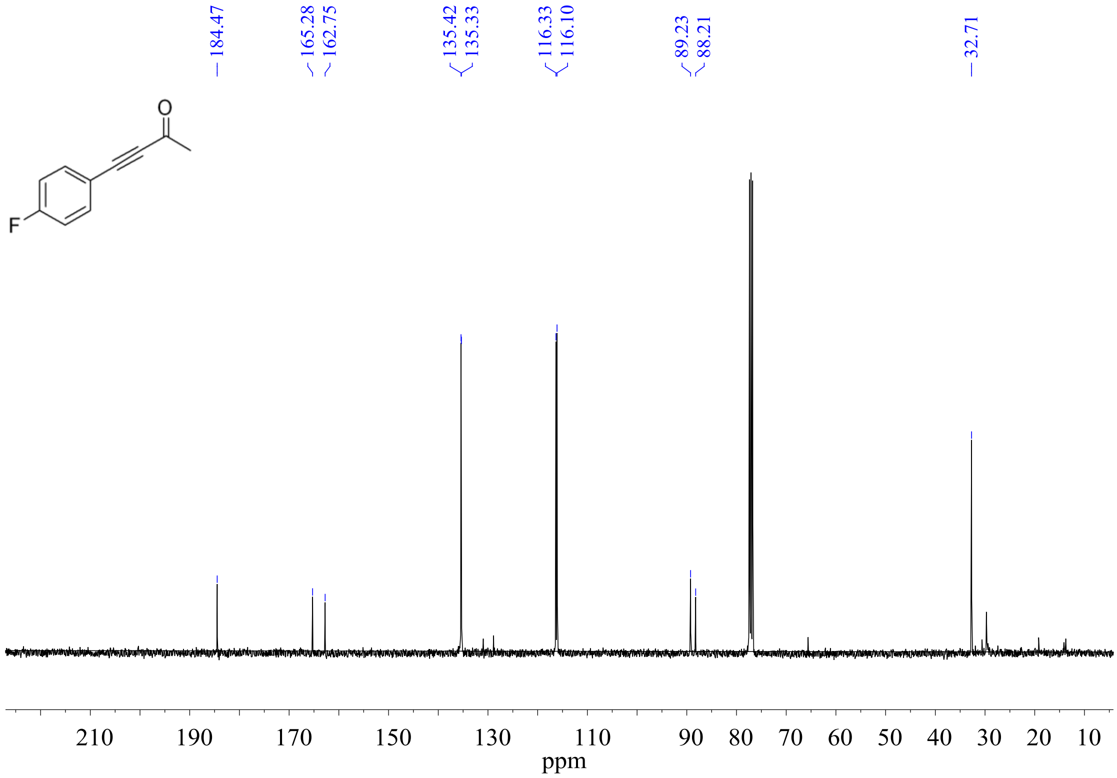

Figure S95. ^{13}C NMR of **1b** synthesized in-house (in $CDCl_3$).

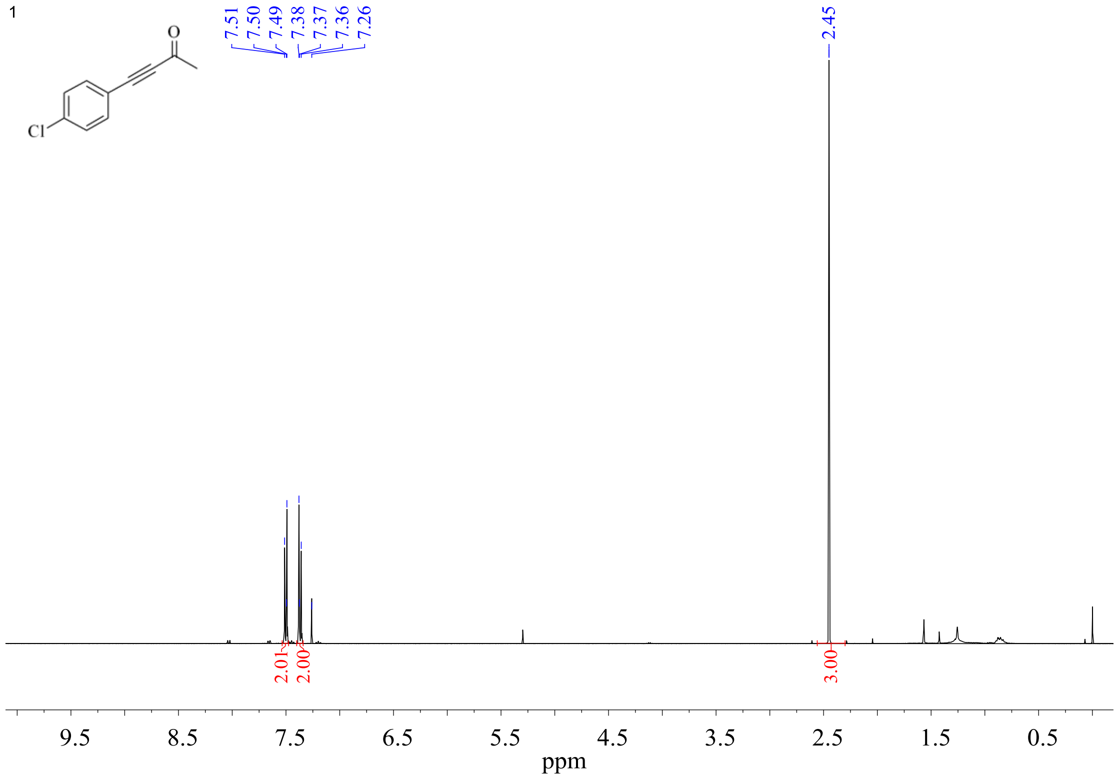

Figure S96. ^{1}H NMR spectrum of **2b** synthesized in-house (in $CDCl_3$).

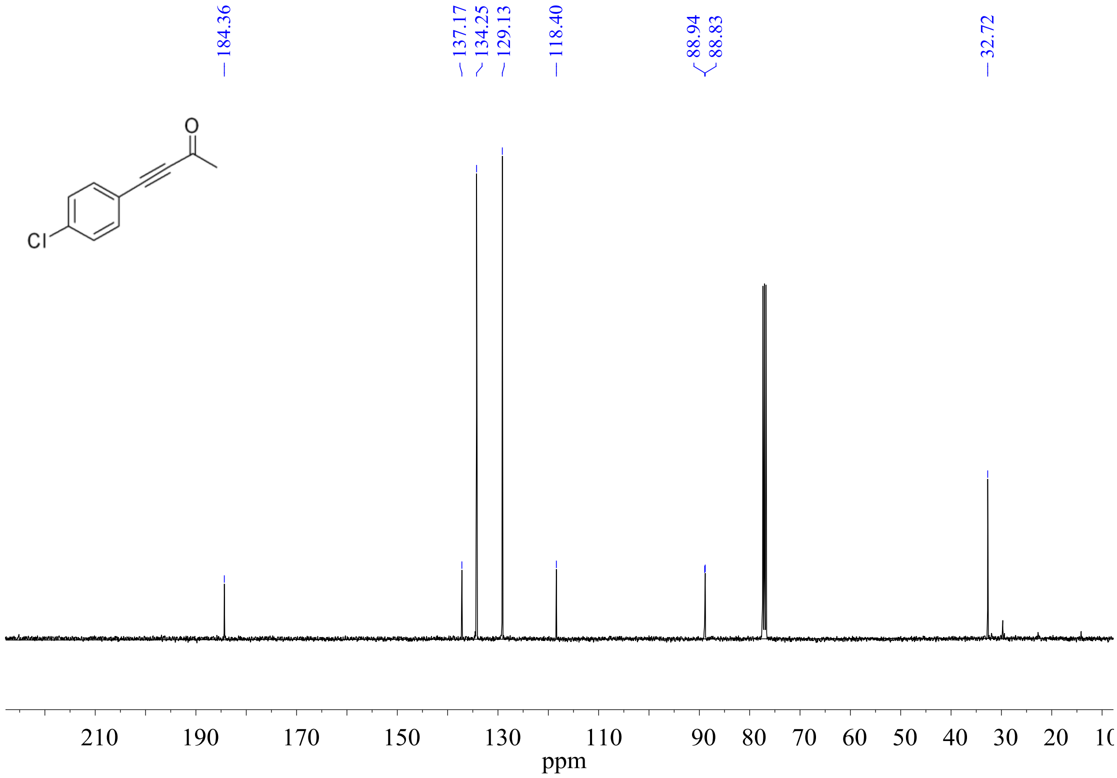

Figure S97. ^{13}C NMR of **2b** synthesized in-house (in $CDCl_3$).

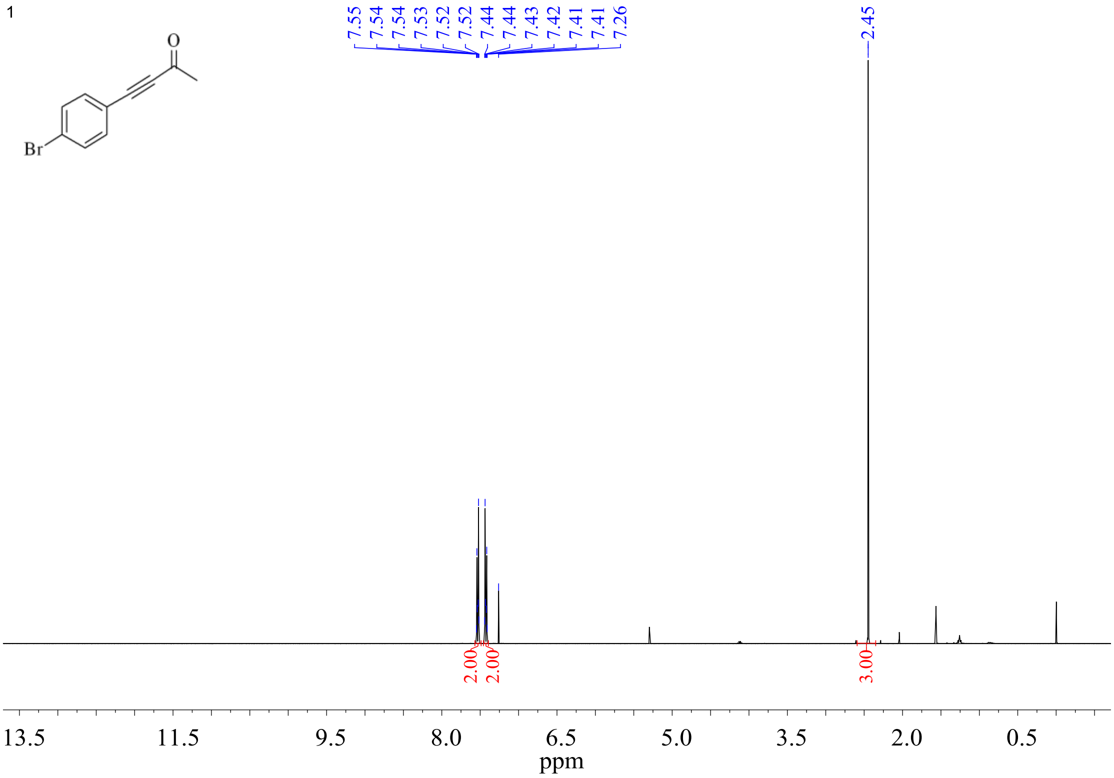

Figure S98. ^{1}H NMR spectrum of **3b** synthesized in-house (in $CDCl_3$).

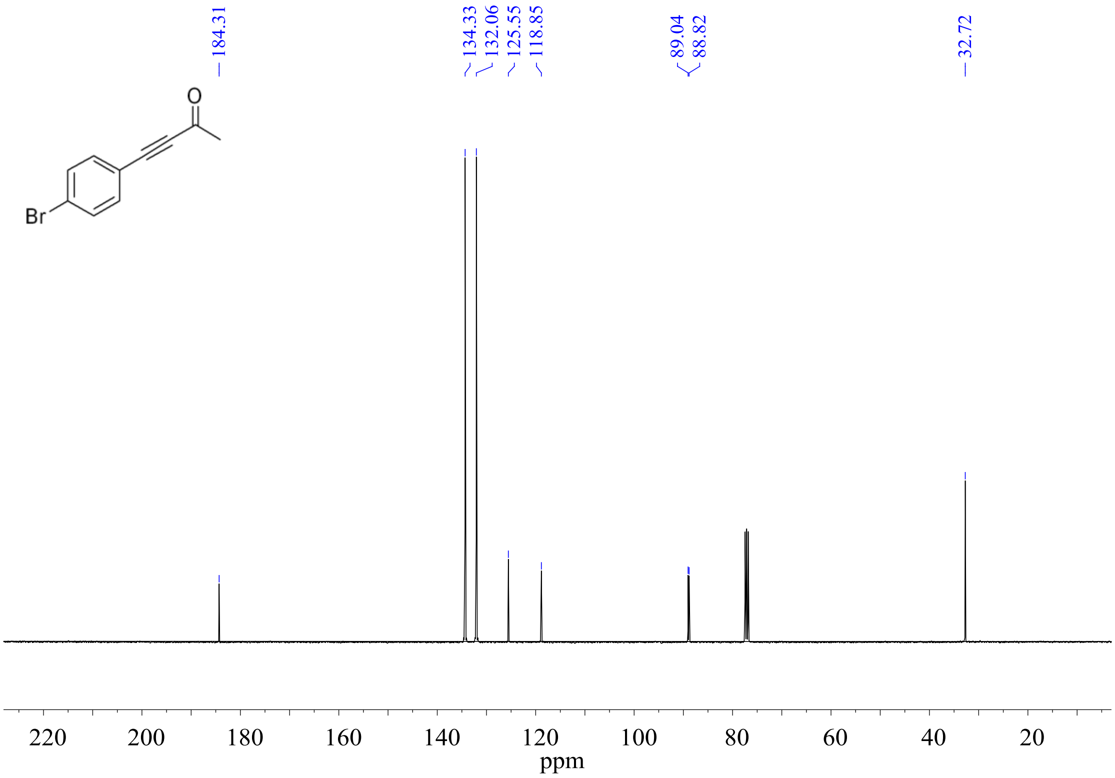

Figure S99. ^{13}C NMR of **3b** synthesized in-house (in $CDCl_3$).

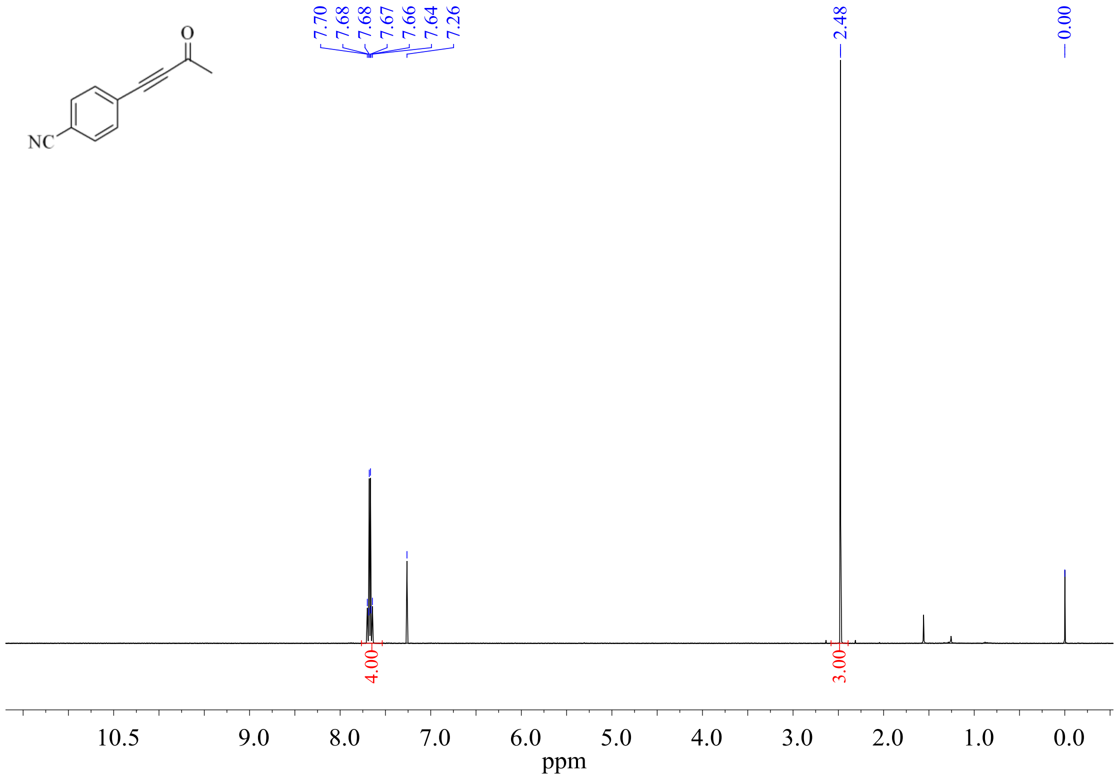

Figure S100. ^{1}H NMR of **4b** synthesized in-house (in $CDCl_3$).

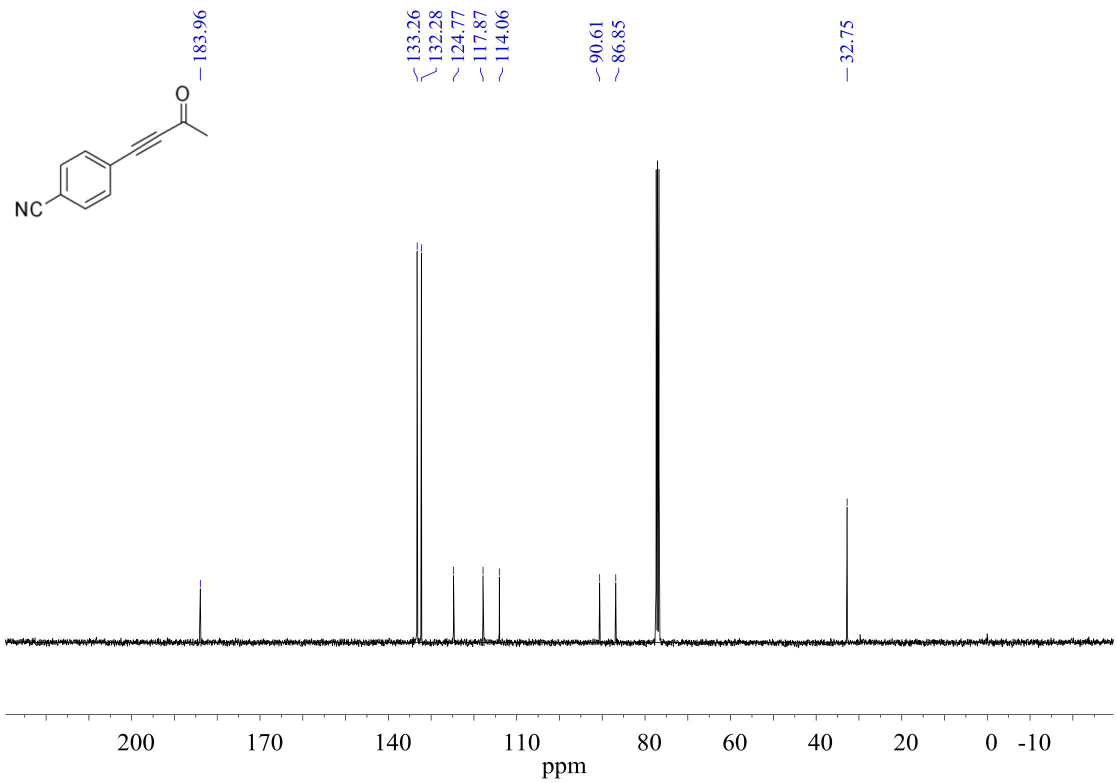

Figure S101. ^{13}C NMR of **4b** synthesized in-house (in $CDCl_3$).

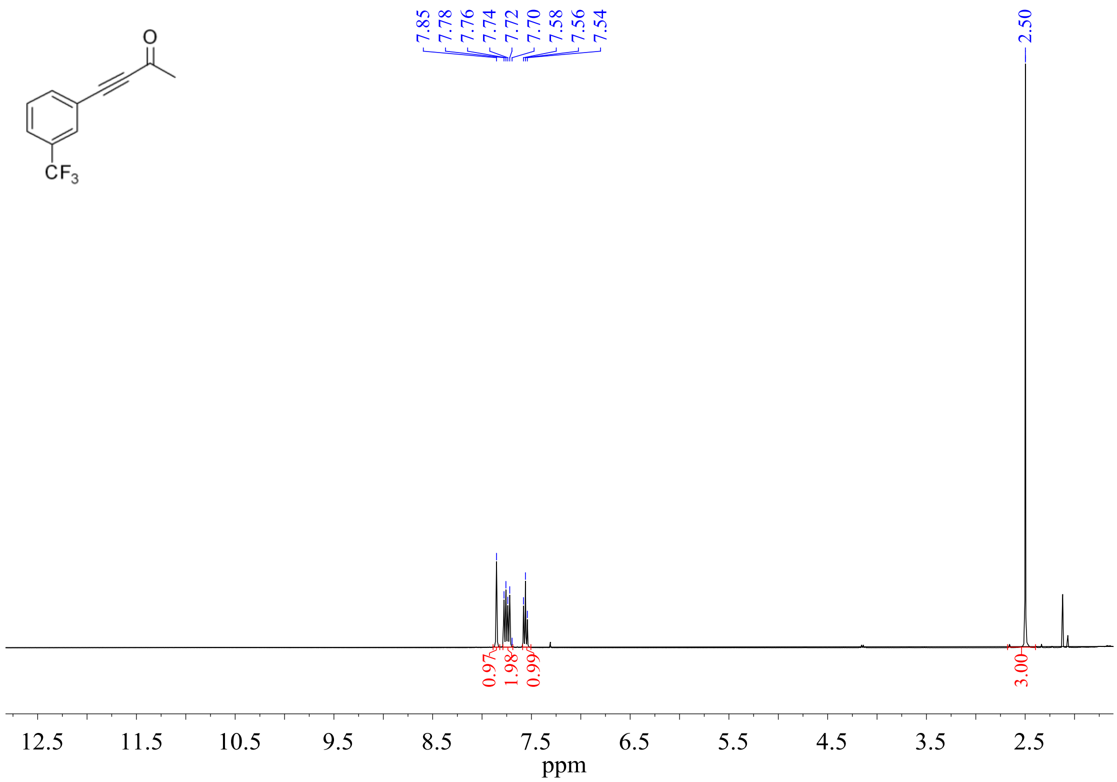

Figure S102. ^{1}H NMR spectrum of **5b** synthesized in-house (in $CDCl_3$).

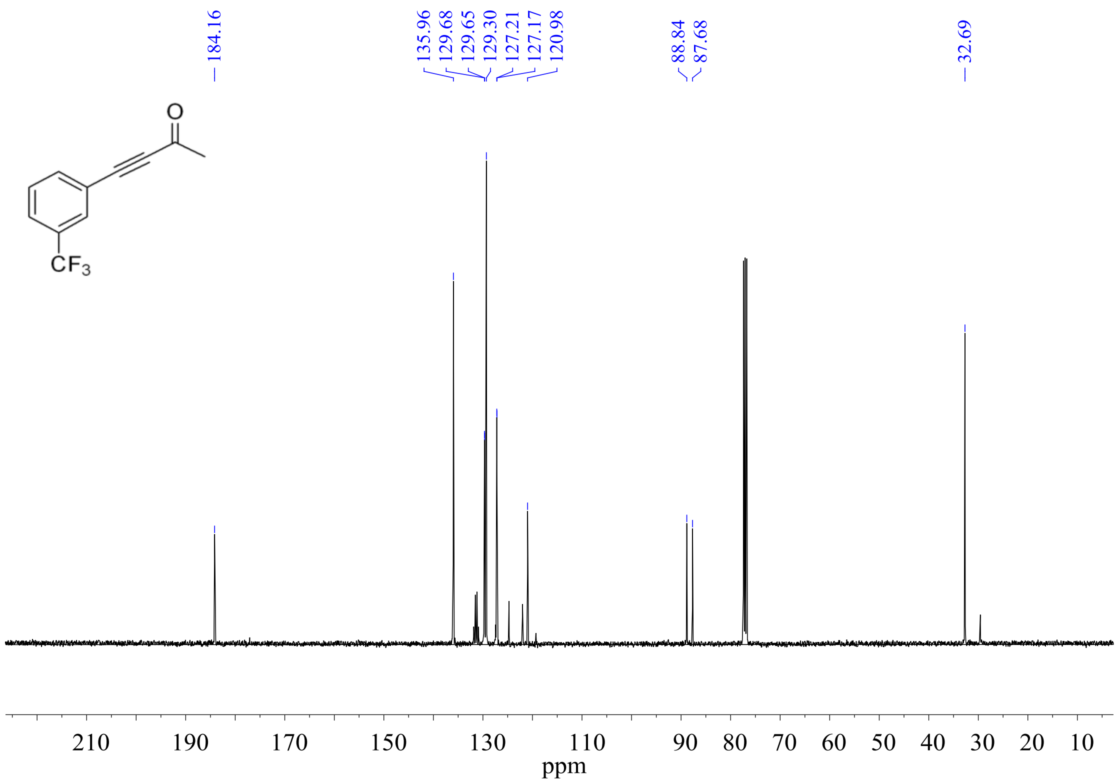

Figure S103. ^{13}C NMR of **5b** synthesized in-house (in $CDCl_3$).

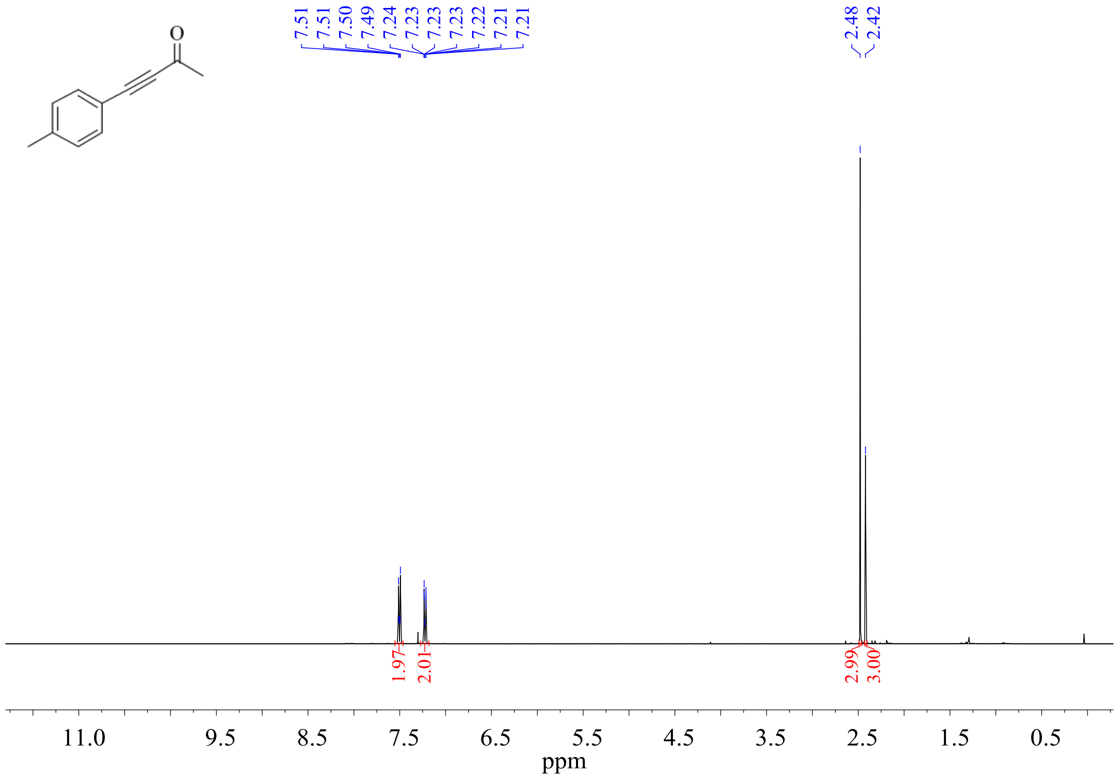

Figure S104. ^{1}H NMR spectrum of **6b** synthesized in-house (in $CDCl_3$).

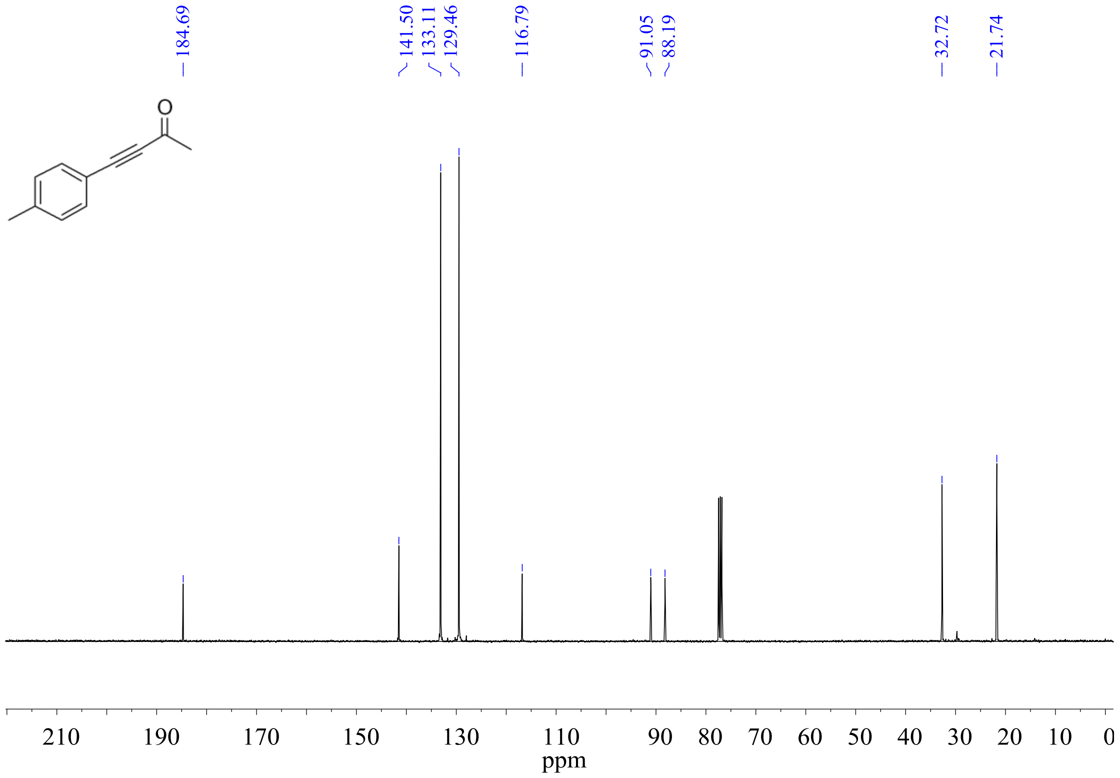

Figure S105. ^{13}C NMR of **6b** synthesized in-house (in $CDCl_3$).

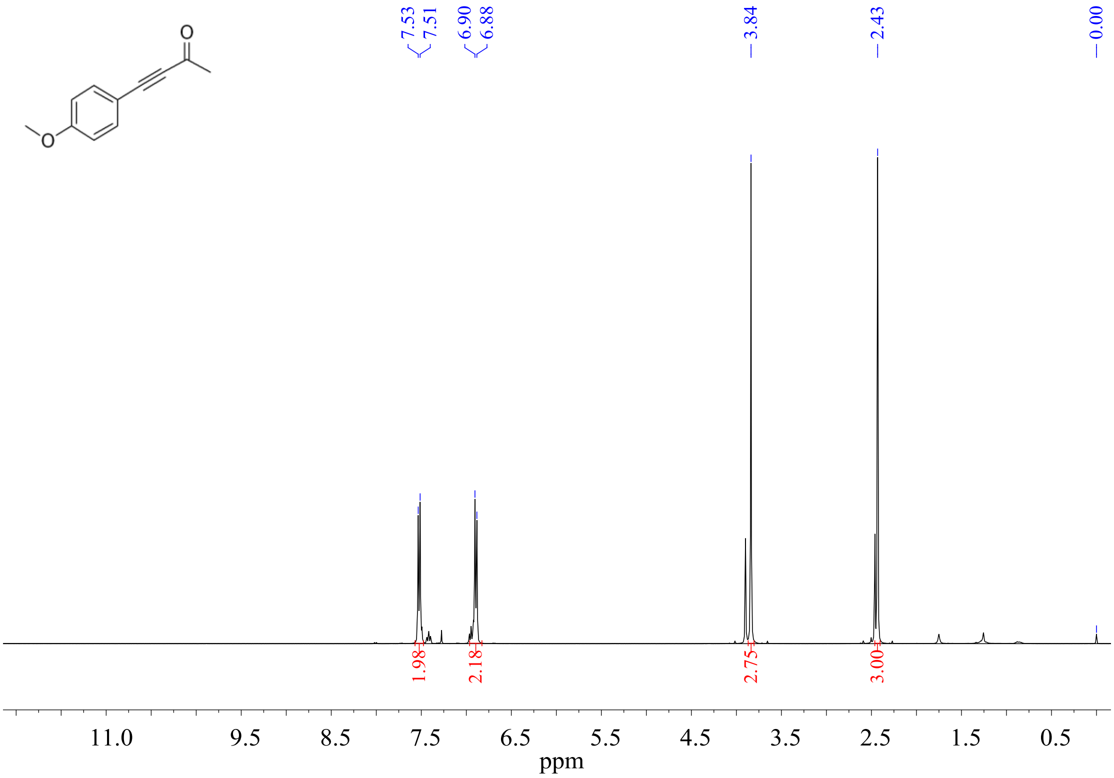

Figure S106. ^{1}H NMR spectrum of **7b** synthesized in-house (in $CDCl_3$).

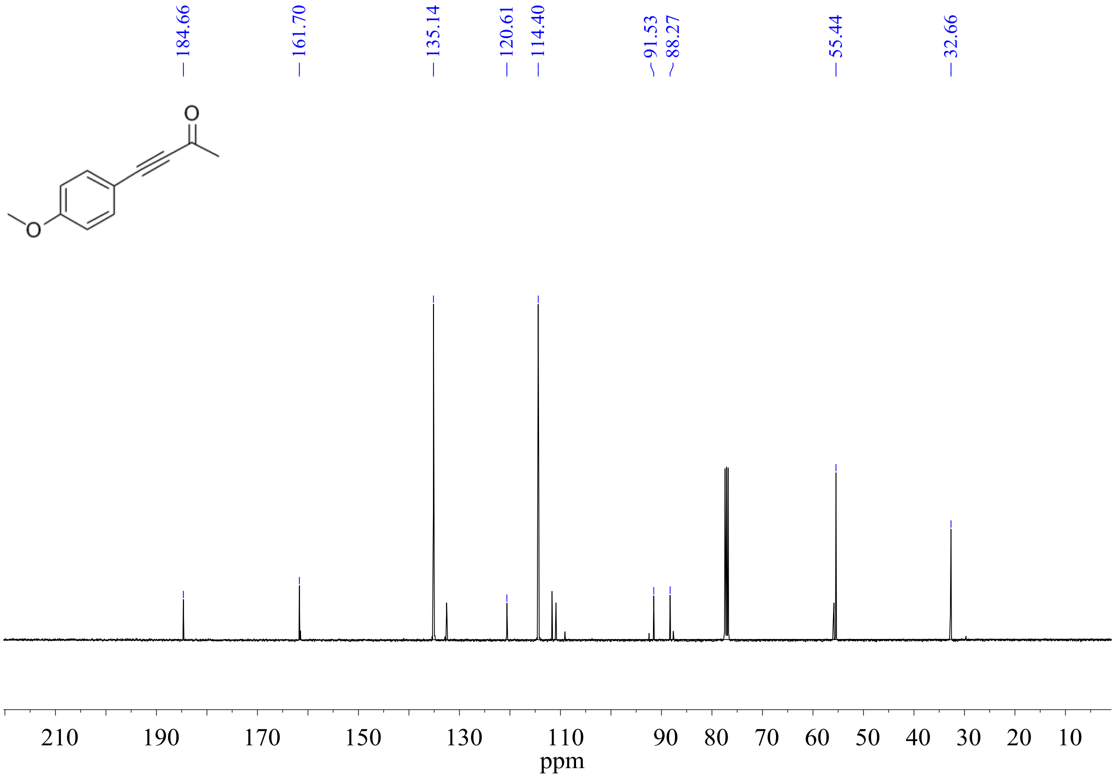

Figure S107. ^{13}C NMR of **7b** synthesized in-house (in $CDCl_3$).

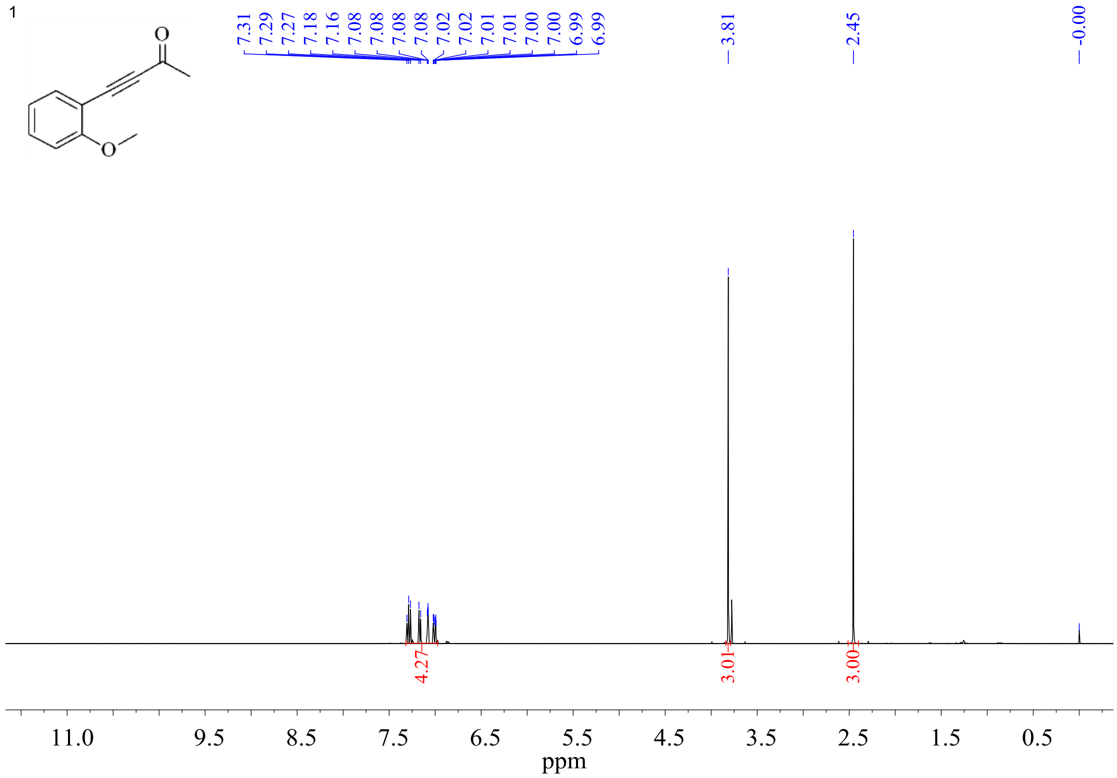

Figure S108. ^{1}H NMR of **8b** synthesized in-house (in $CDCl_3$).

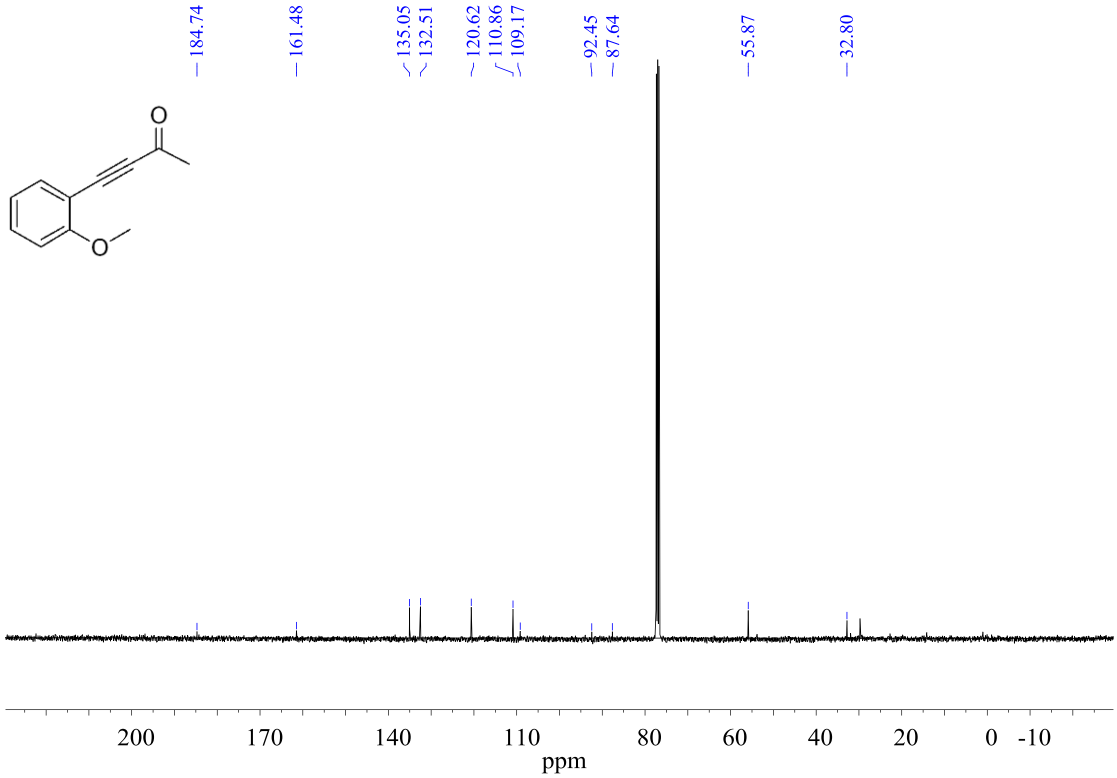

Figure S109. ^{13}C NMR of **8b** synthesized in-house (in $CDCl_3$).

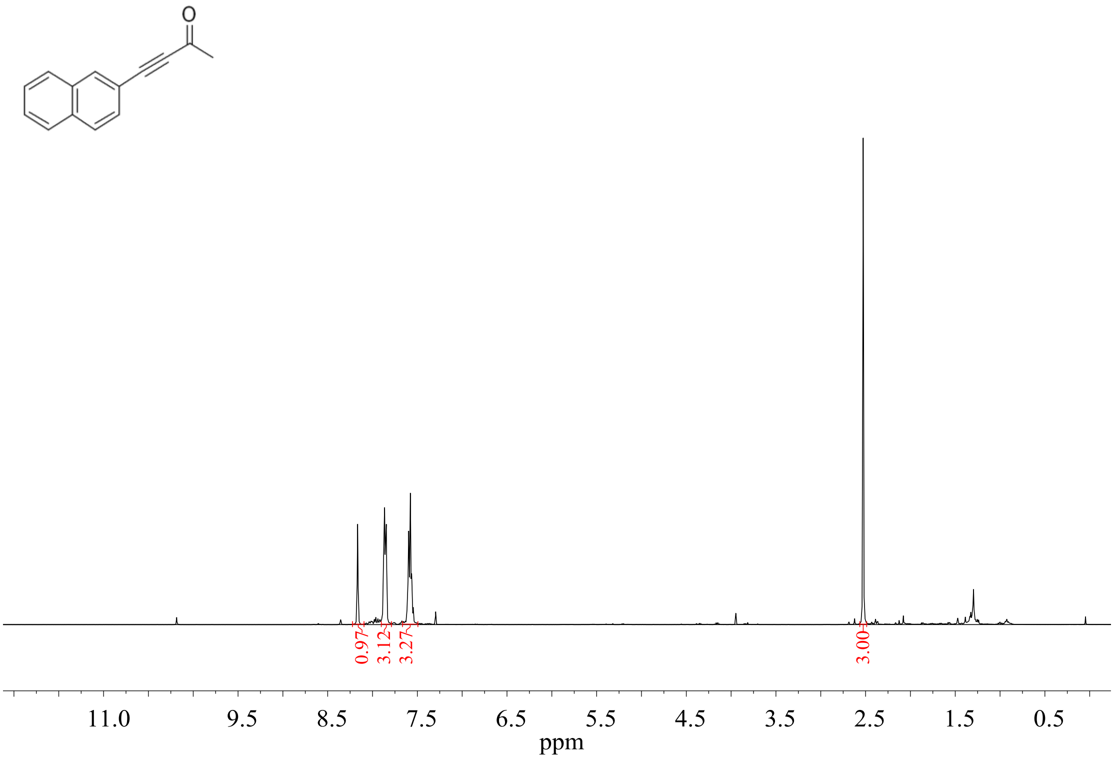

Figure S110. ^{1}H NMR of **10b** synthesized in-house (in $CDCl_3$).

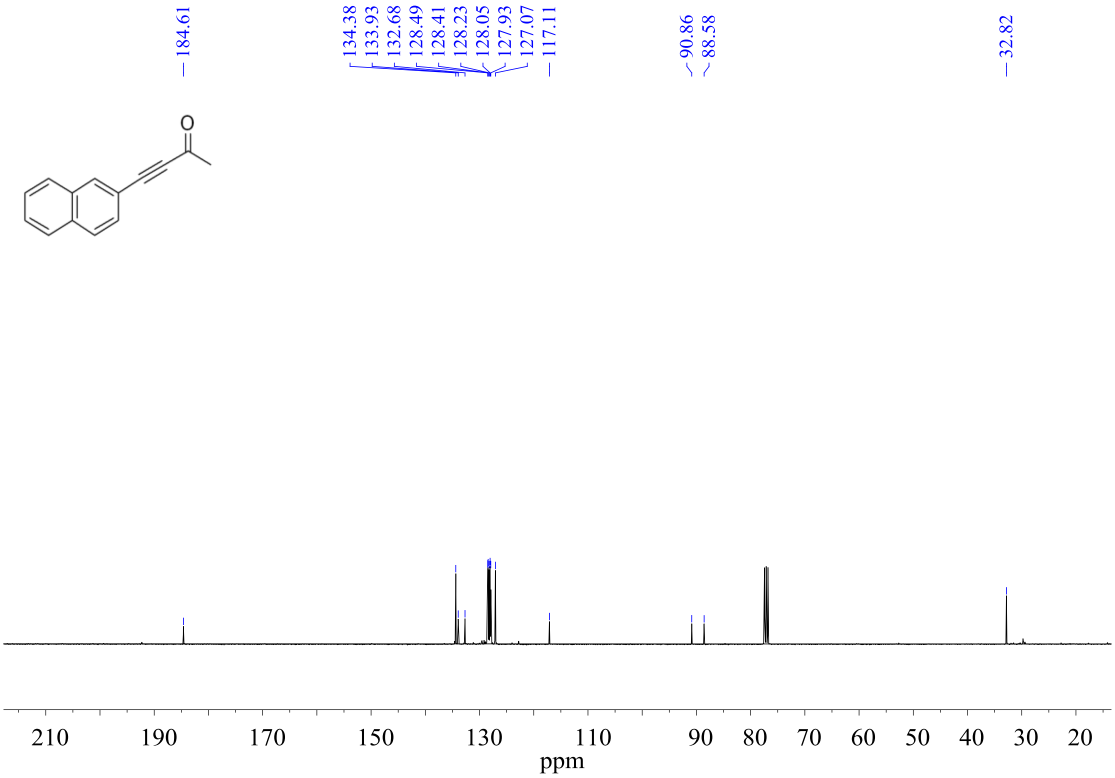

Figure S111. ^{13}C NMR of **10b** synthesized in-house (in $CDCl_3$).

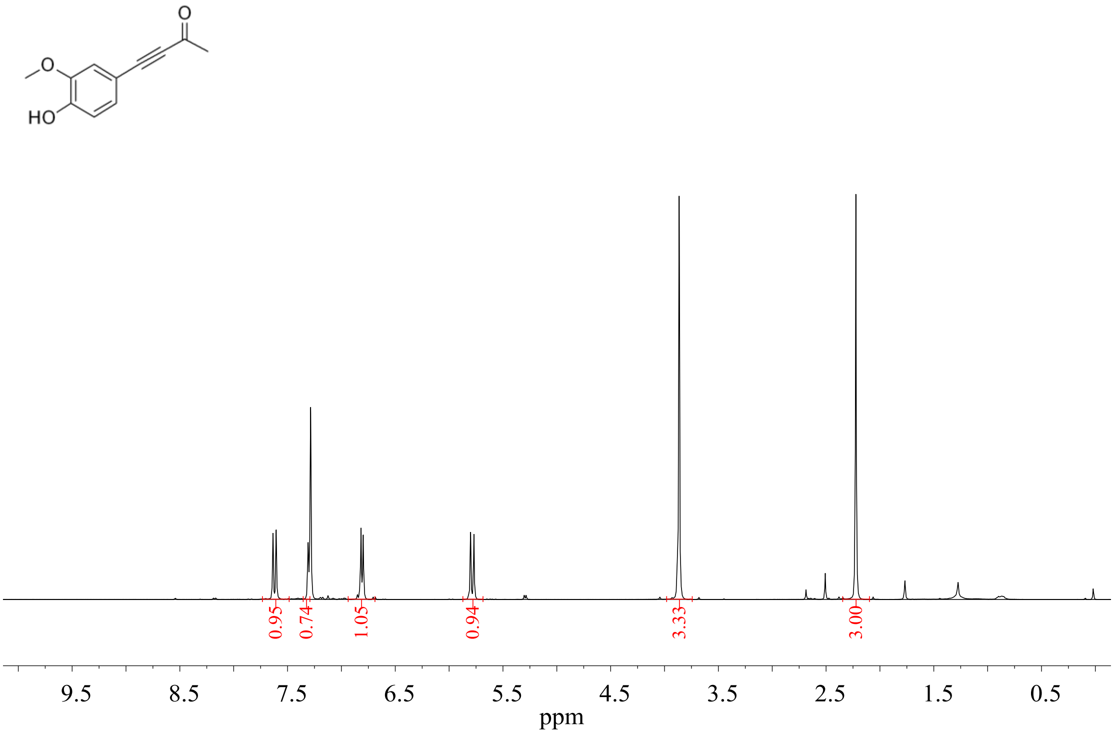

Figure S112. ^{1}H NMR spectrum of **11b** synthesized in-house (in $CDCl_3$).

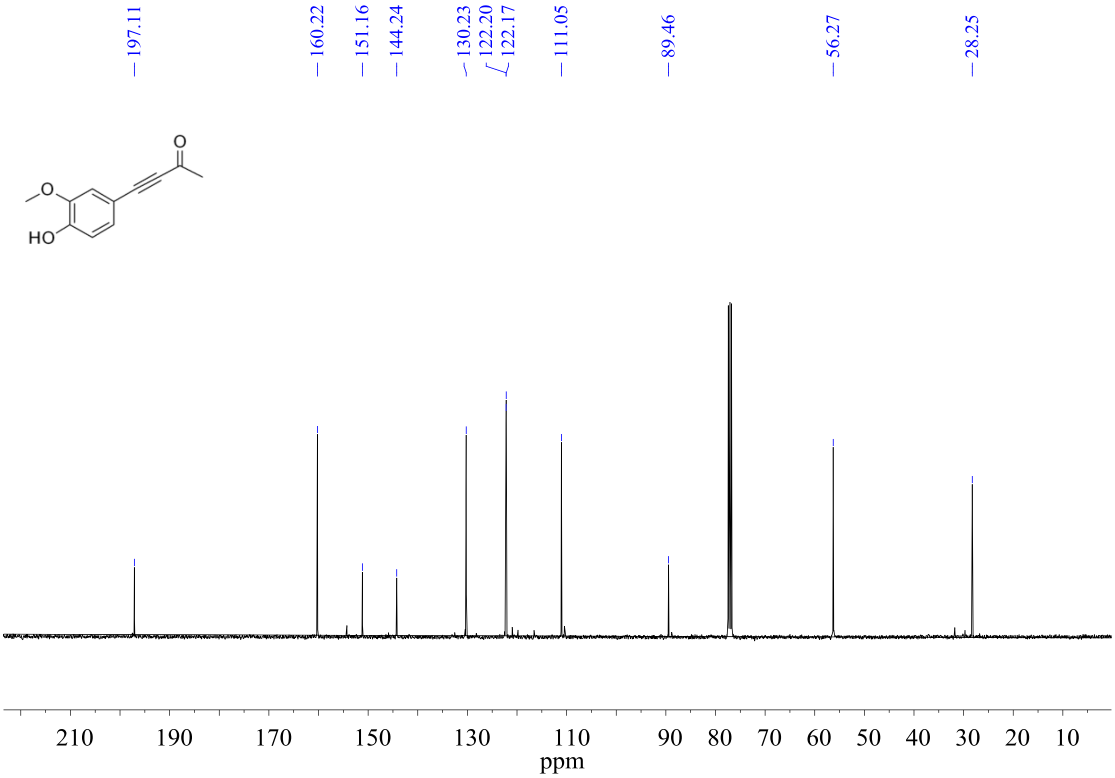

Figure S113. ^{13}C NMR of **11b** synthesized in-house (in $CDCl_3$).

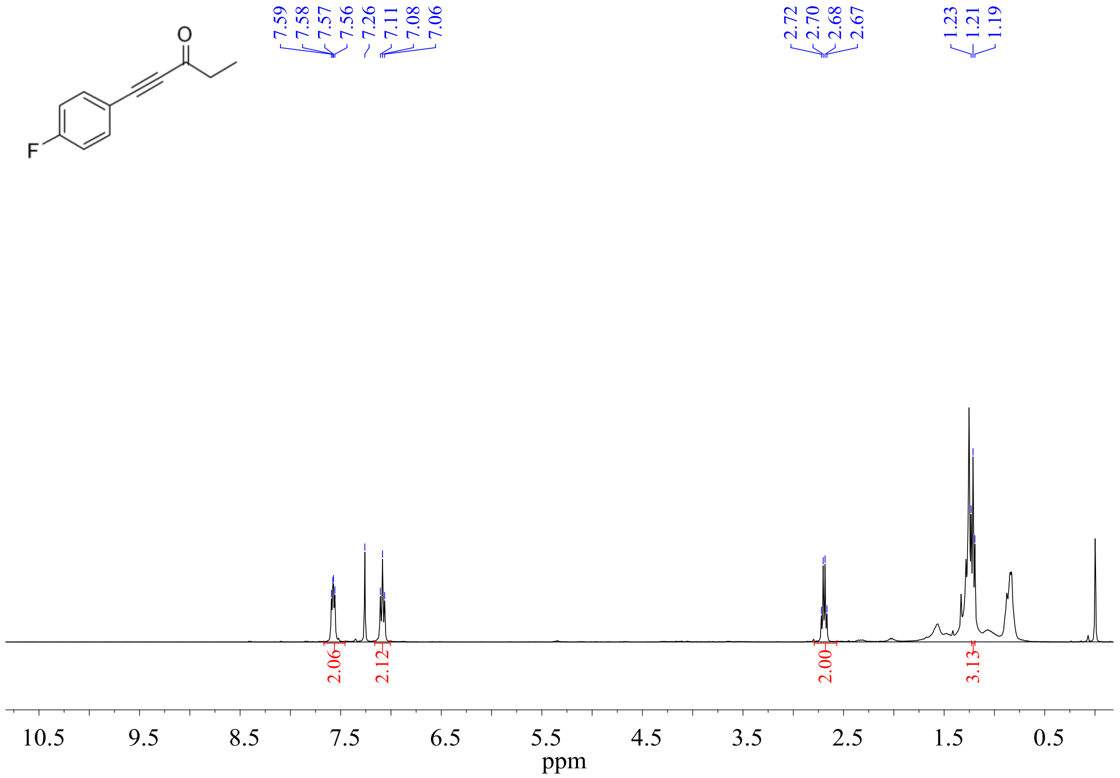

Figure S114. ^{1}H NMR of **12b** synthesized in-house (in $CDCl_3$).

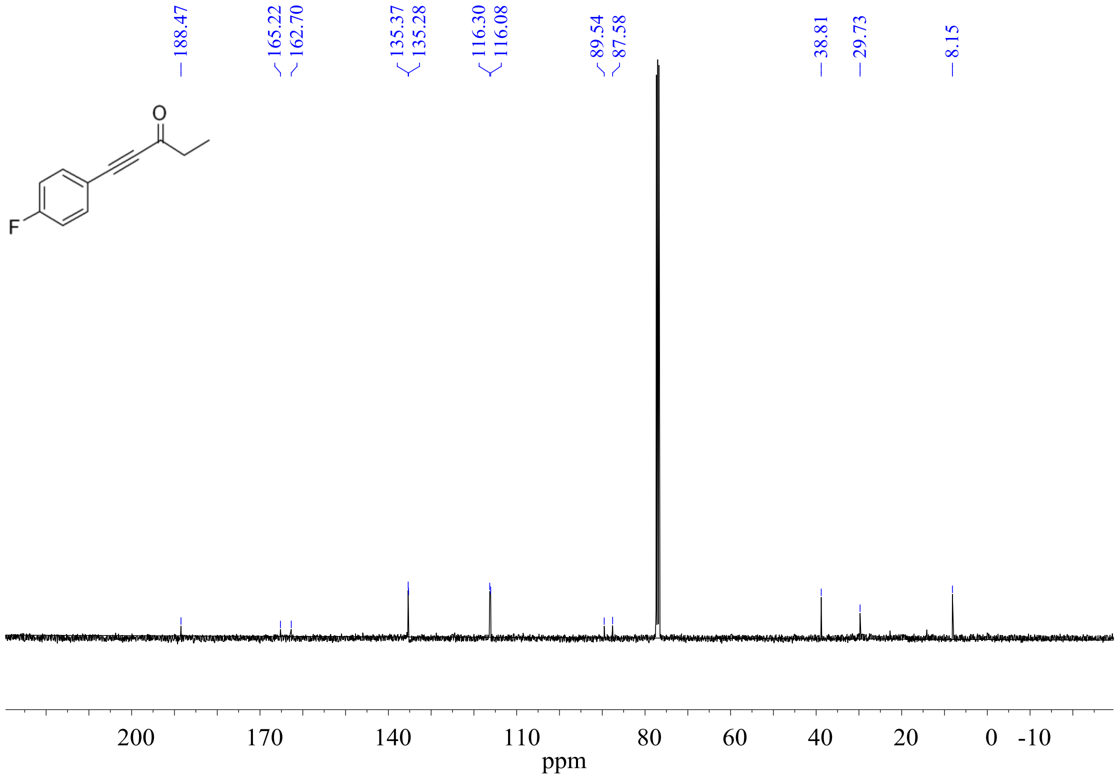

Figure S115. ^{13}C NMR of **12b** synthesized in-house (in $CDCl_3$).

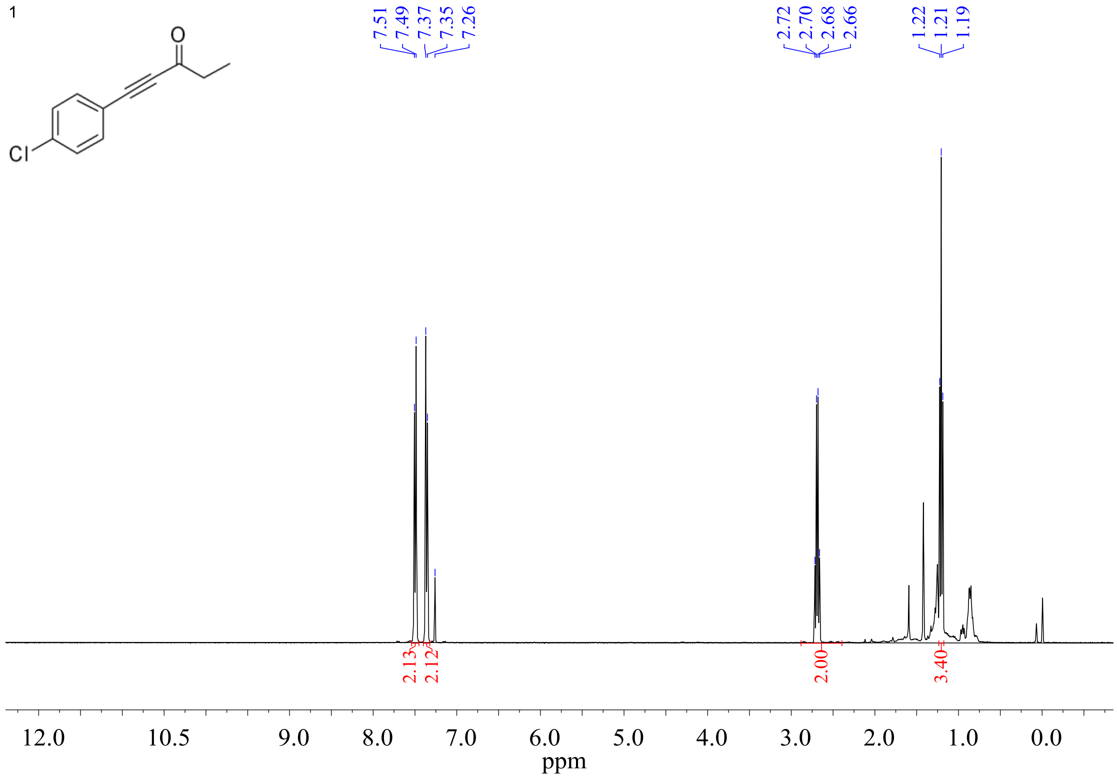

Figure S116. ^{1}H NMR of **13b** synthesized in-house (in $CDCl_3$).

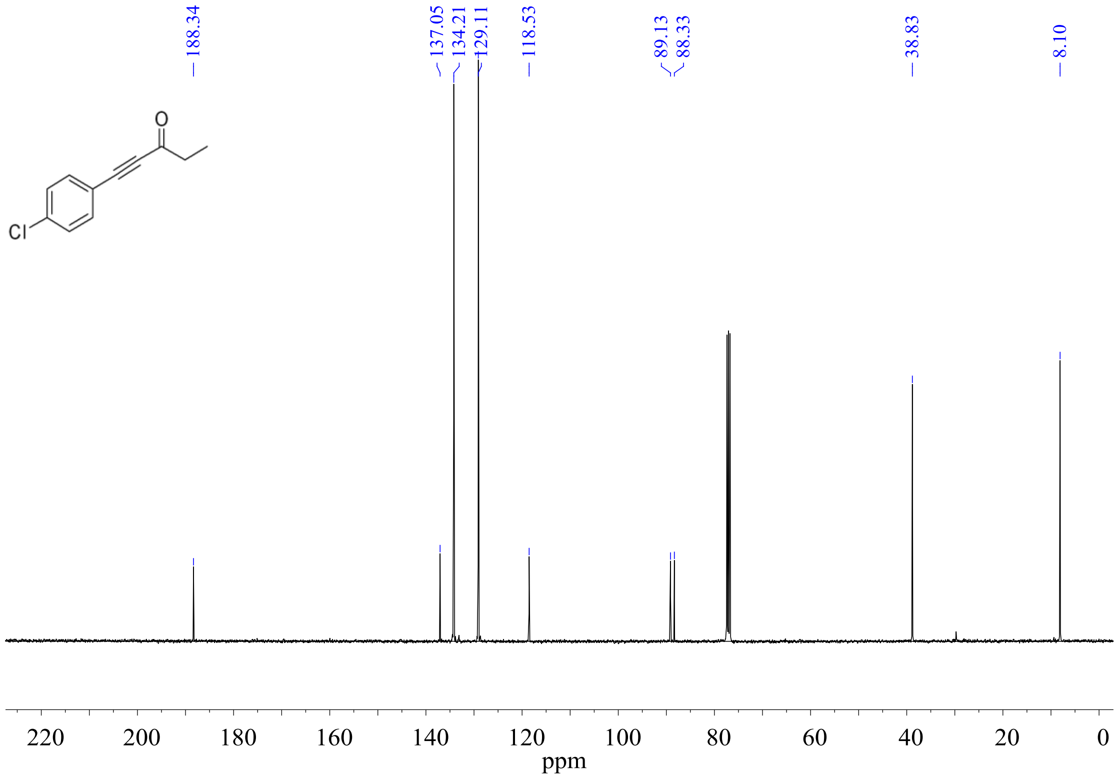

Figure S117. ^{13}C NMR of **13b** synthesized in-house (in $CDCl_3$).

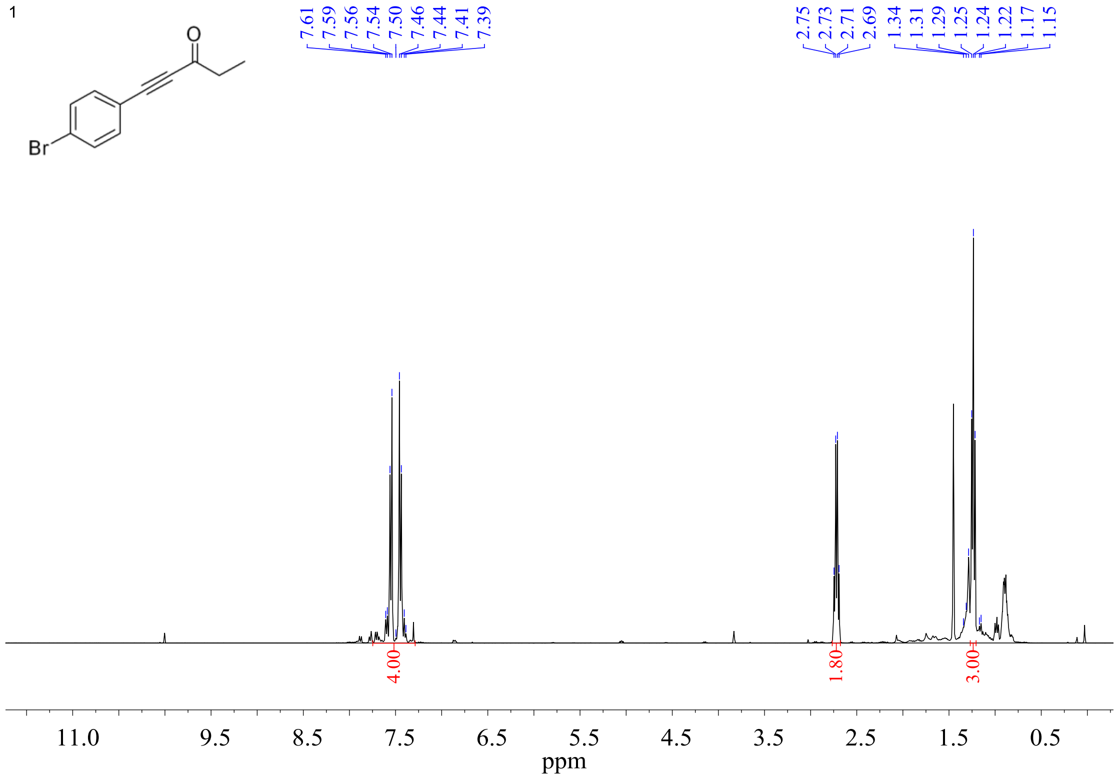

Figure S118. ^{1}H NMR spectrum of **14b** synthesized in-house (in $CDCl_3$).

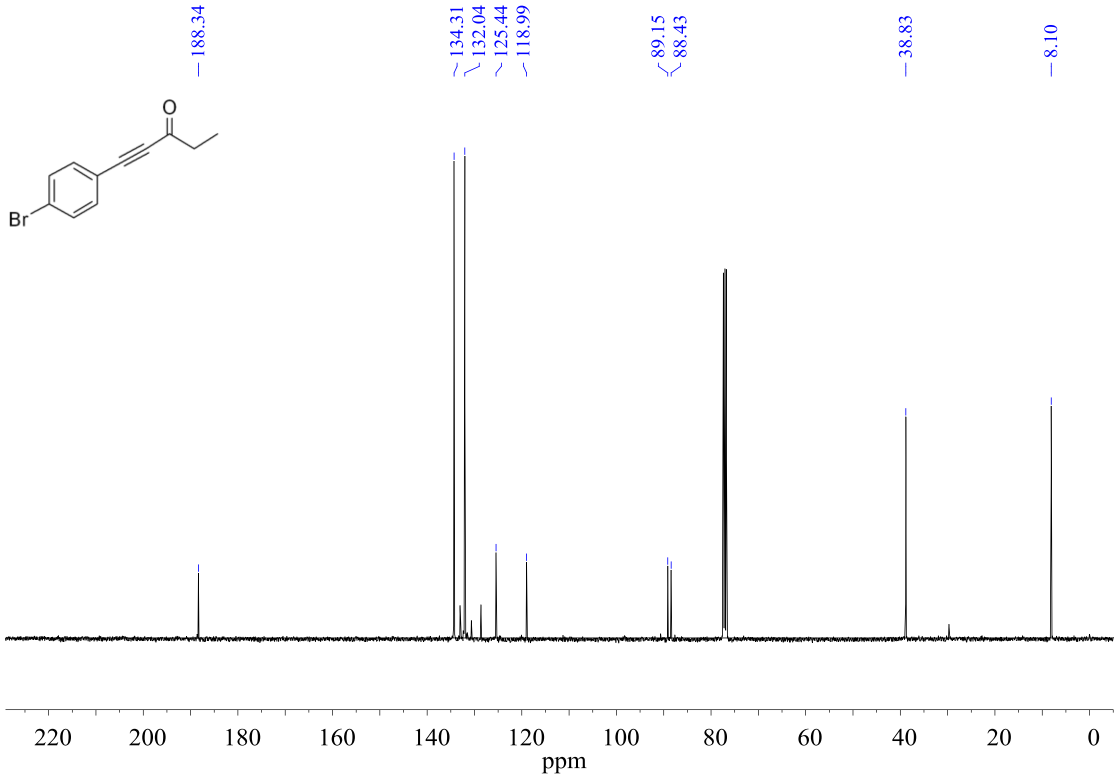

Figure S119. ^{13}C NMR spectrum of **14b** synthesized in-house (in $CDCl_3$).

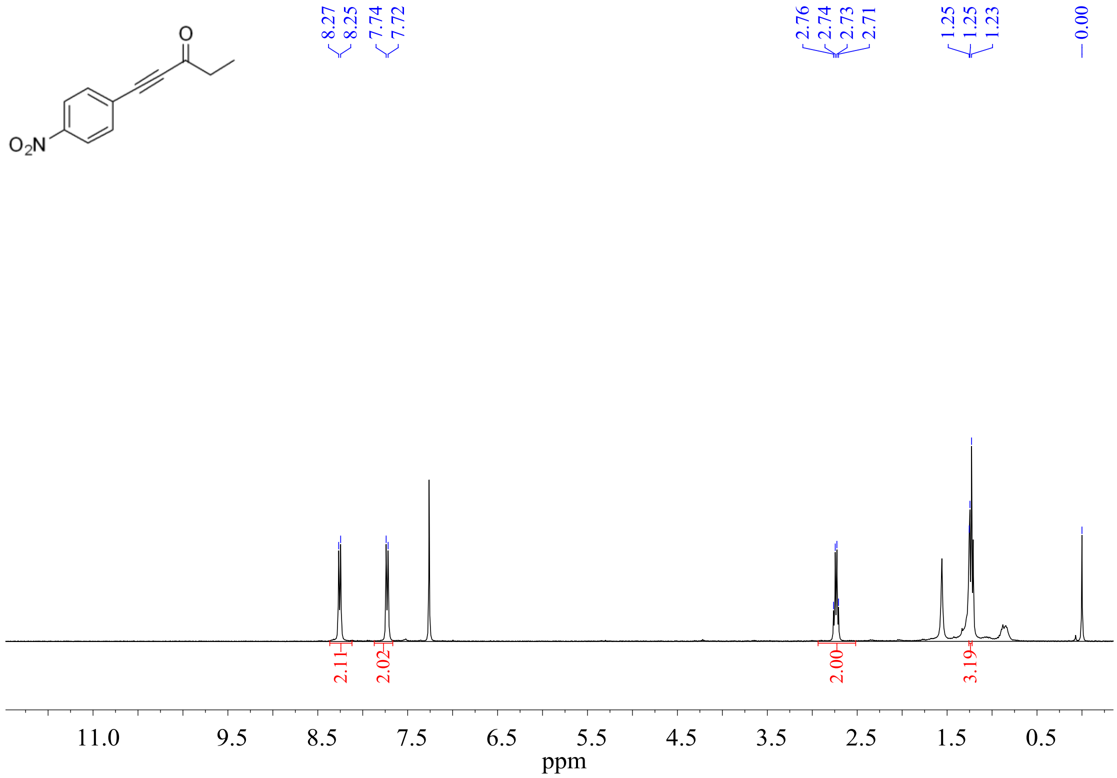

Figure S120. ^{1}H NMR spectrum of **15b** synthesized in-house (in $CDCl_3$).

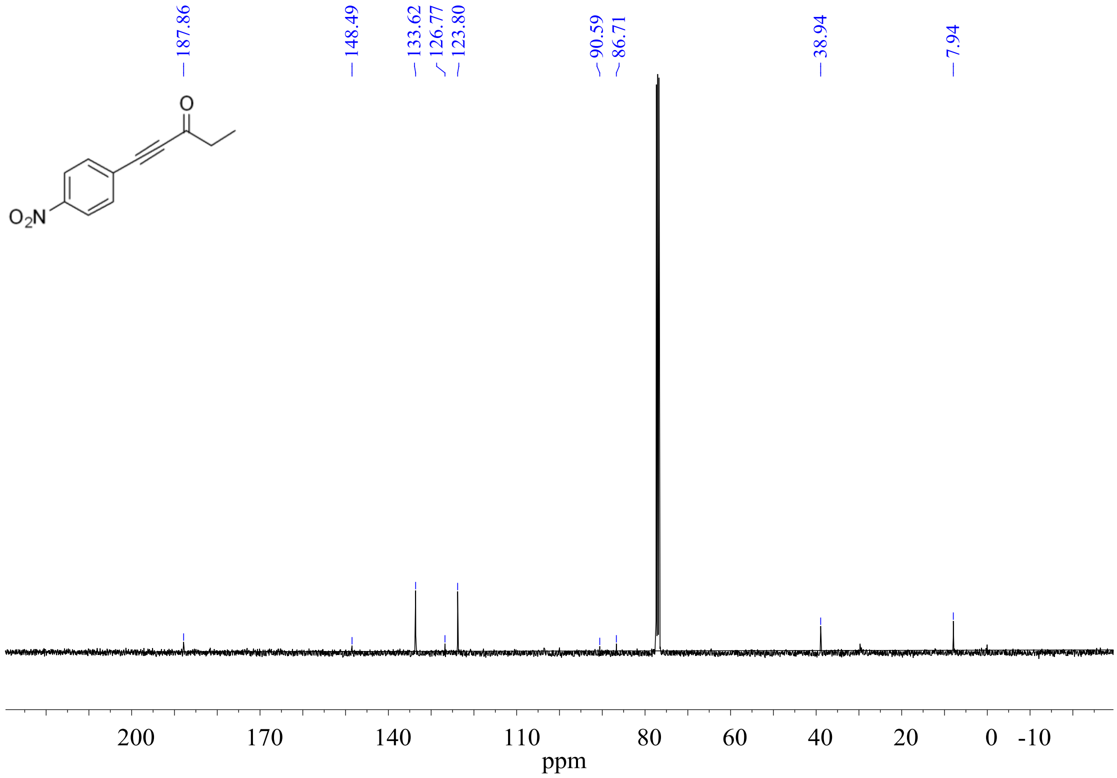

Figure S121. ^{13}C NMR of **15b** synthesized in-house (in $CDCl_3$).

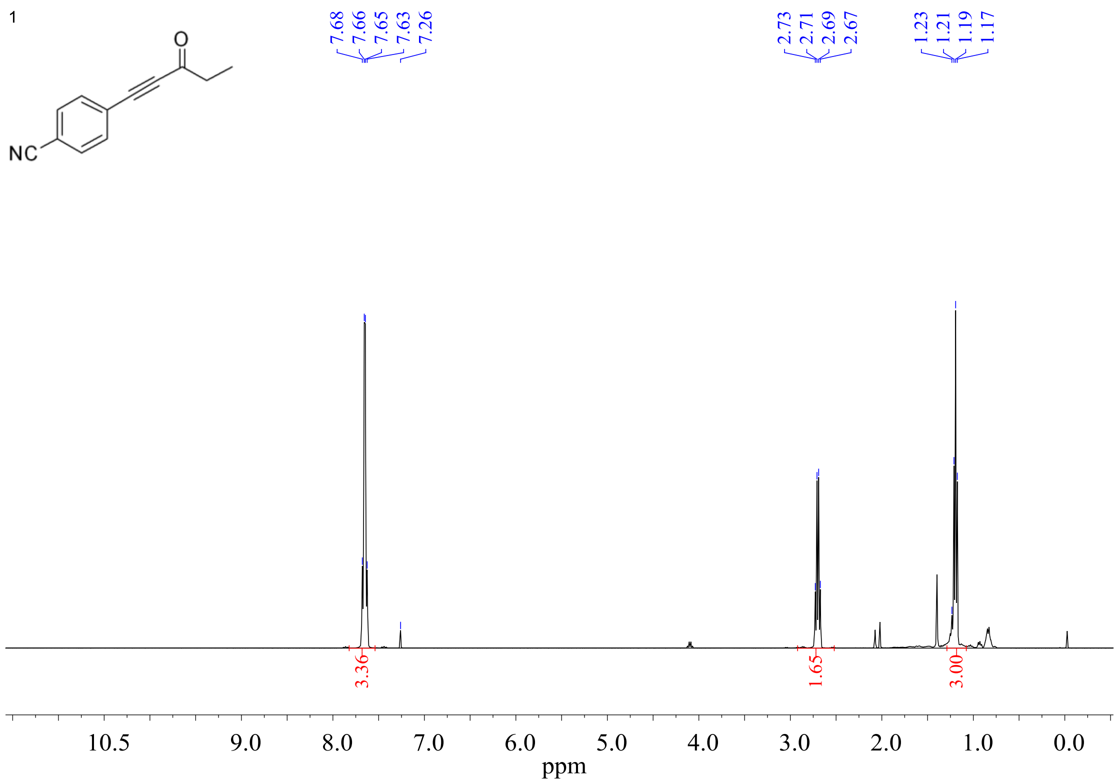

Figure S122. ^{1}H NMR spectrum of **16b** synthesized in-house (in $CDCl_3$).

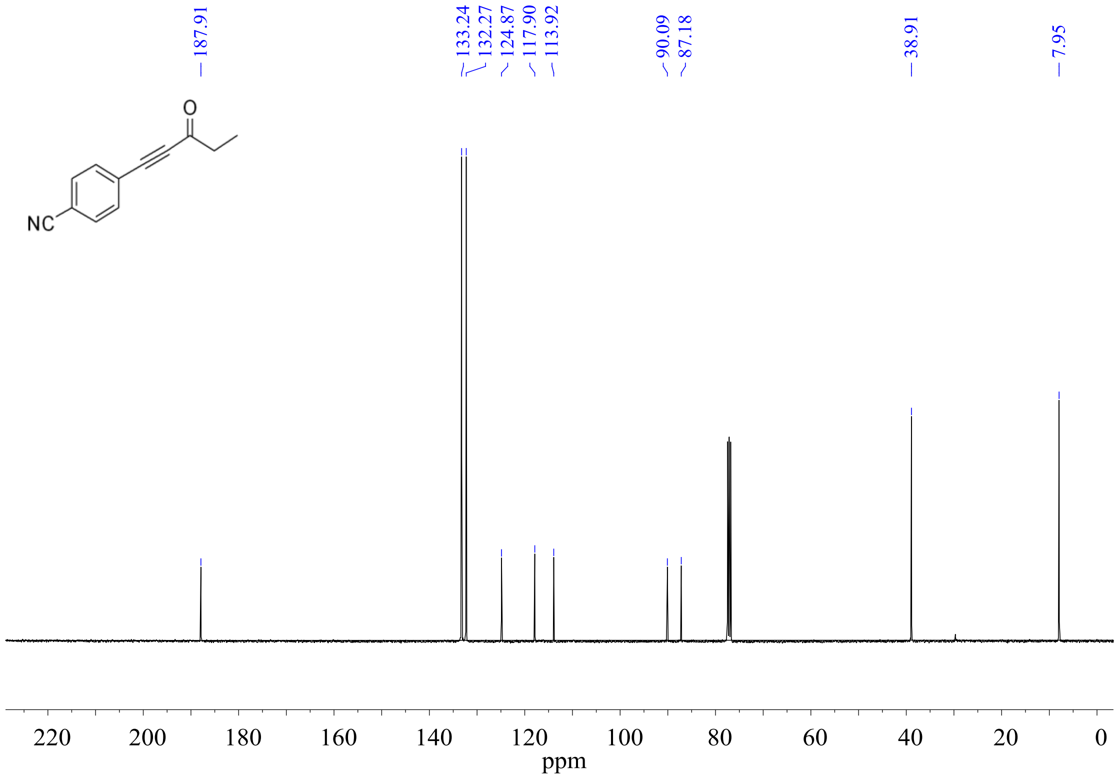

Figure S123. ^{13}C NMR of **16b** synthesized in-house (in $CDCl_3$).

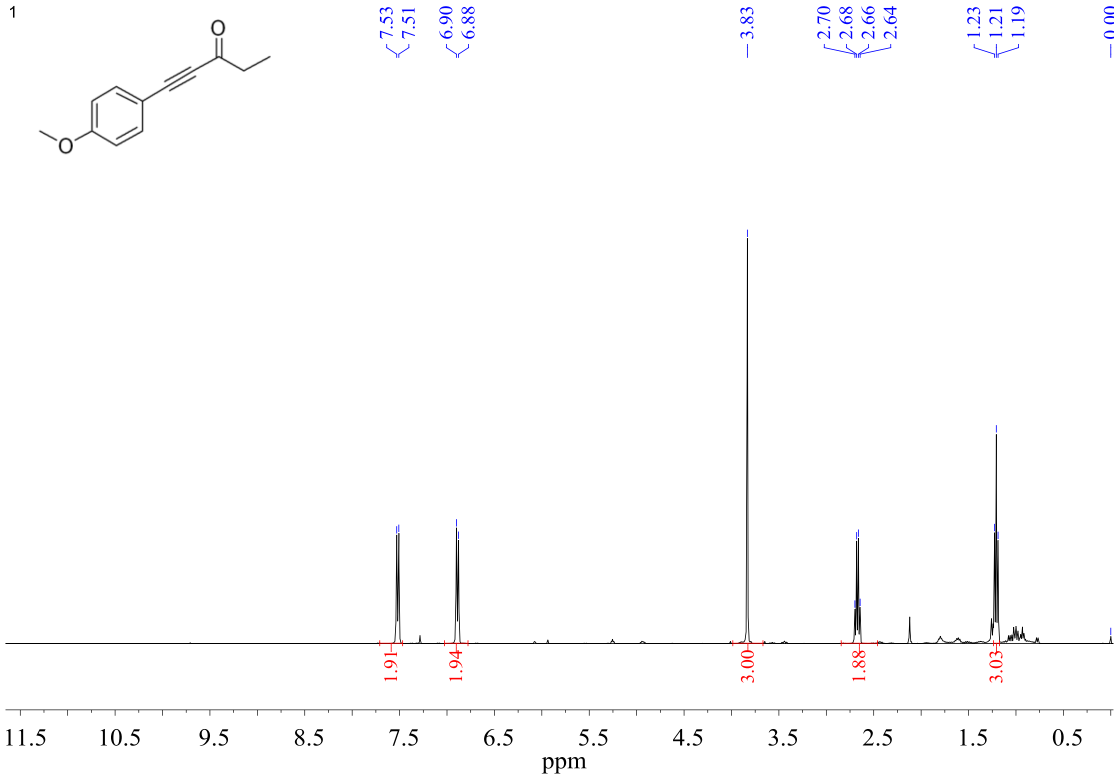

Figure S124. ^{1}H NMR spectrum of **17b** synthesized in-house (in $CDCl_3$).

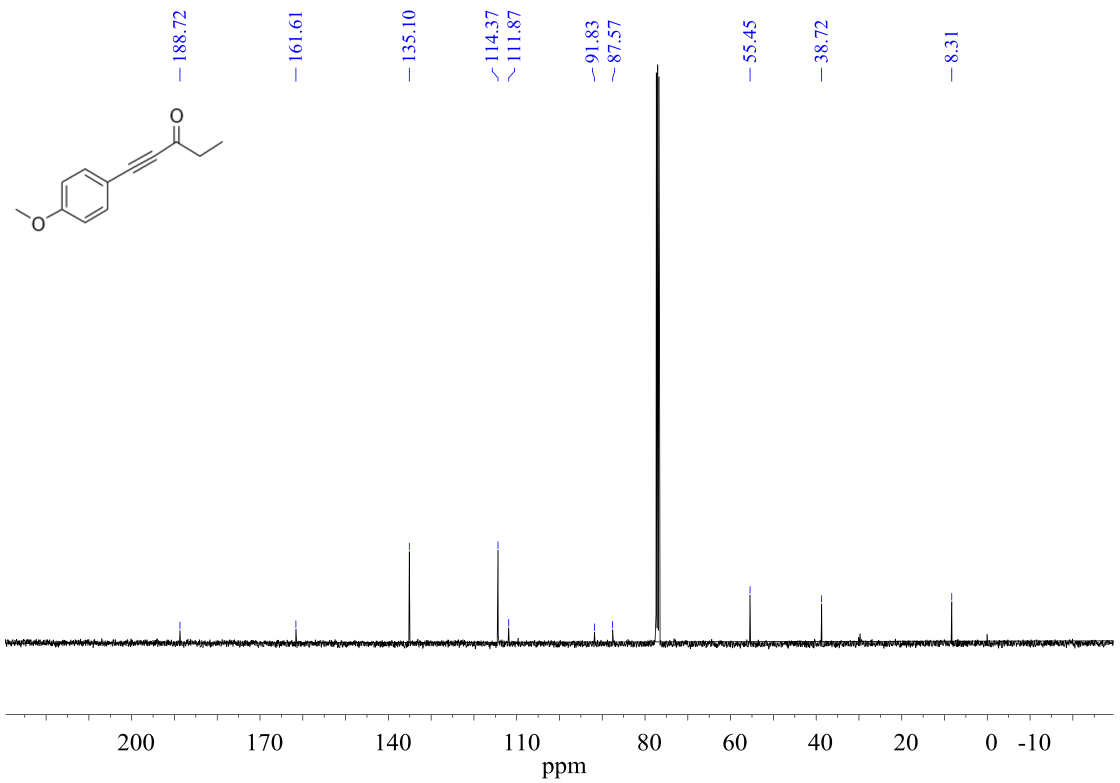

Figure S125. ^{13}C NMR of **17b** synthesized in-house (in $CDCl_3$).

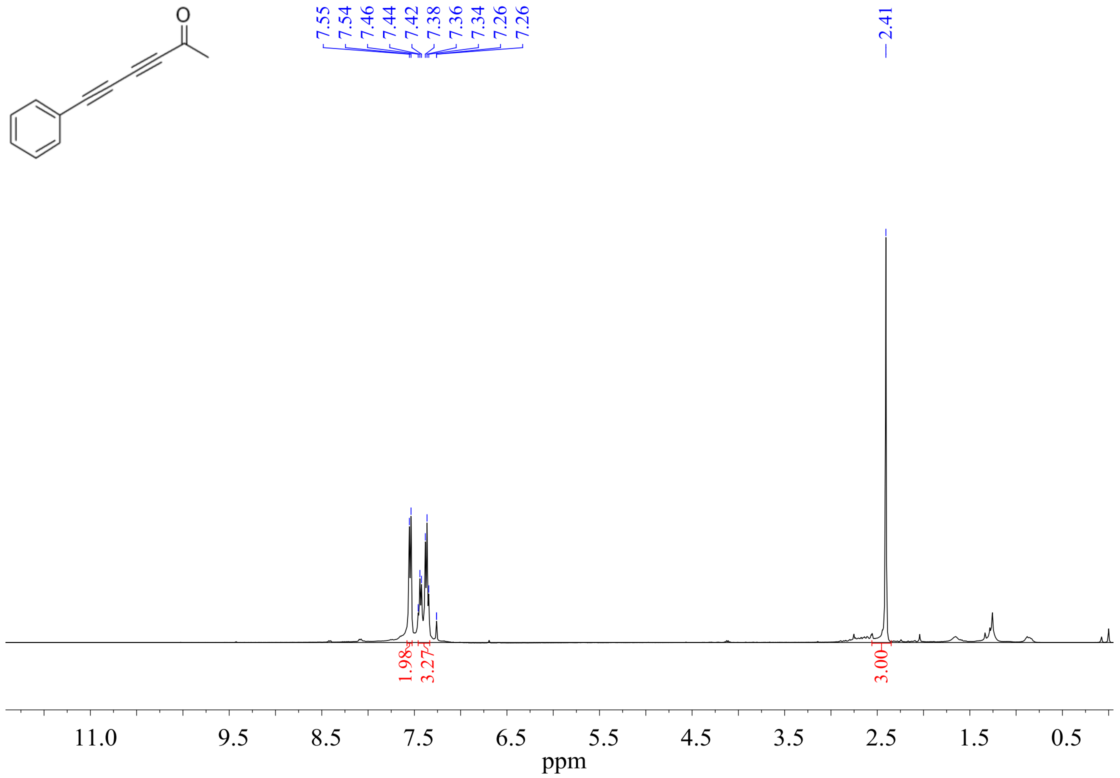

Figure S126. ^{1}H NMR of **21b** synthesized in-house (in $CDCl_3$).

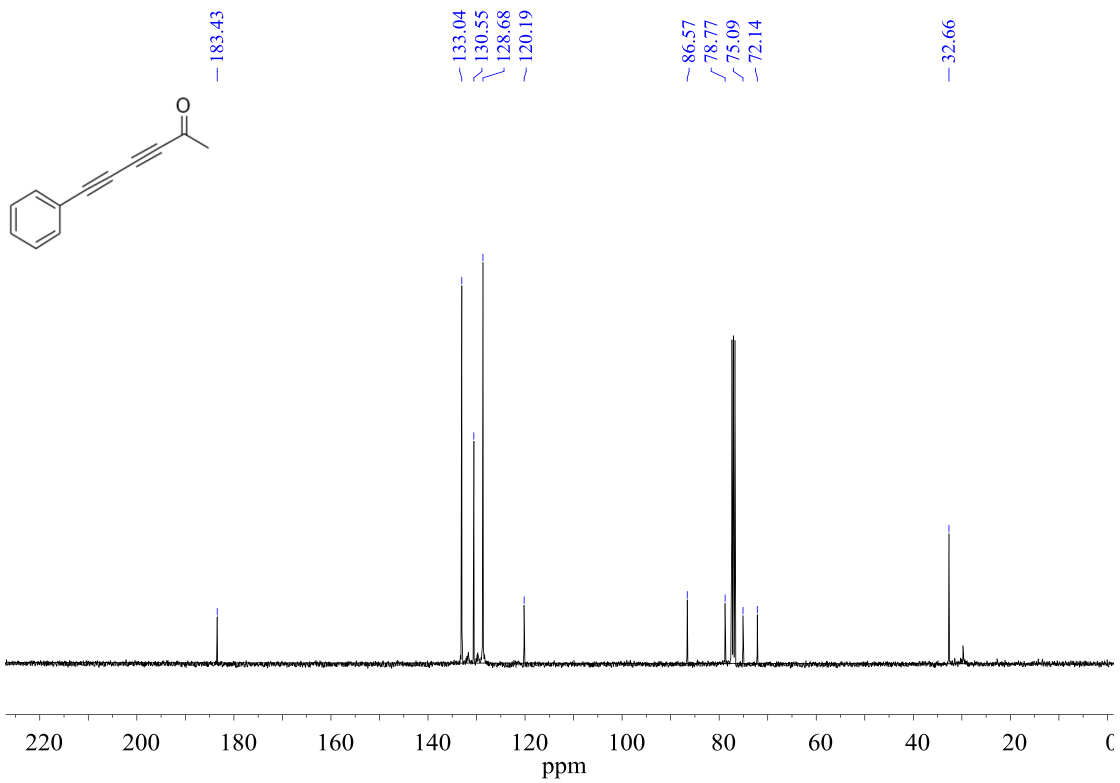

Figure S127. ^{13}C NMR of **21b** synthesized in-house (in $CDCl_3$).

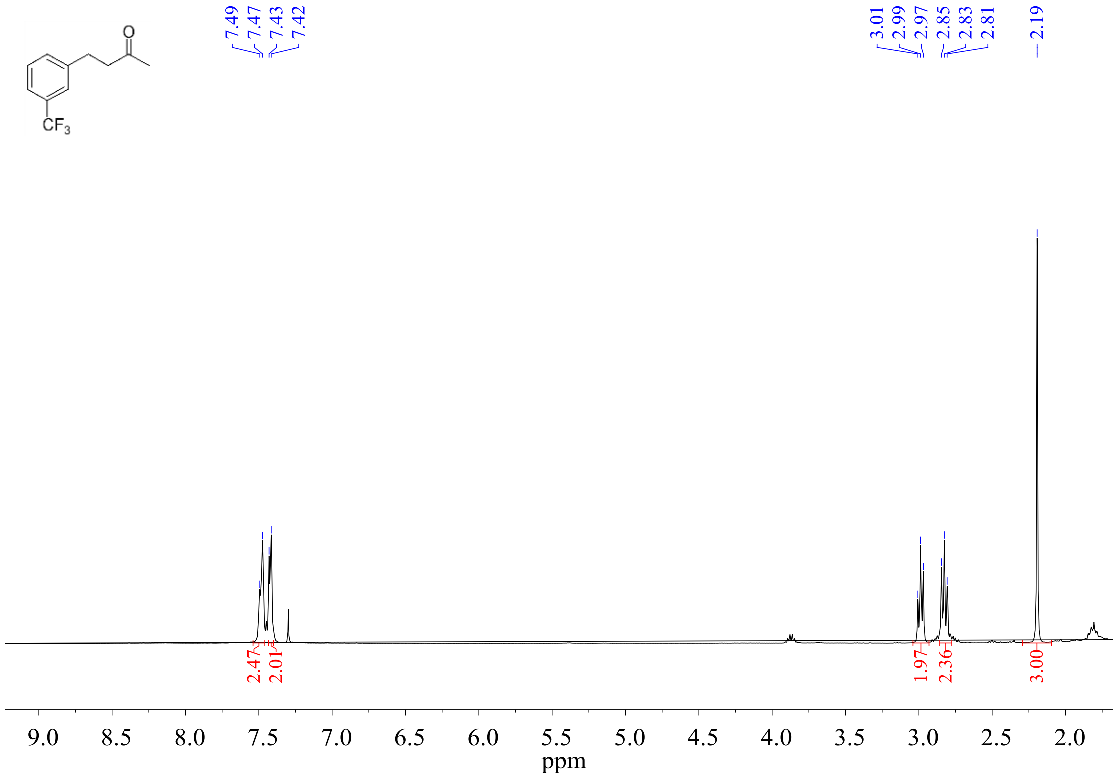

Figure S128. ^{1}H NMR spectrum of **5c** synthesized in-house (in $CDCl_3$).

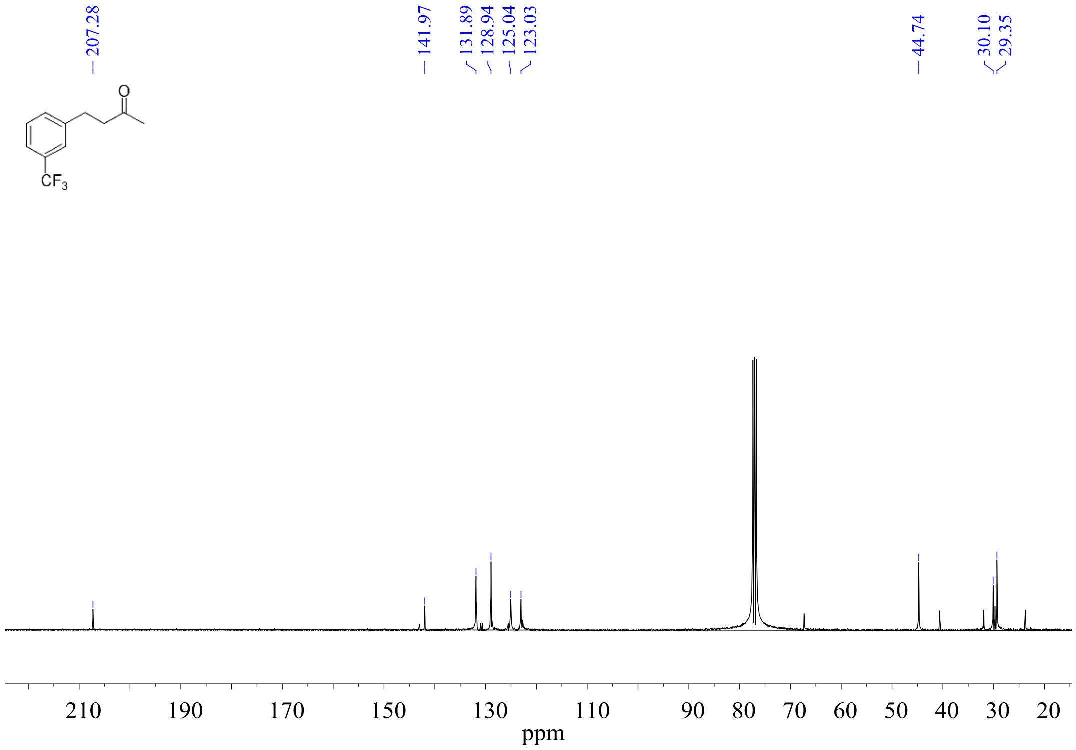

Figure S129. ^{13}C NMR of **5c** synthesized in-house (in $CDCl_3$).

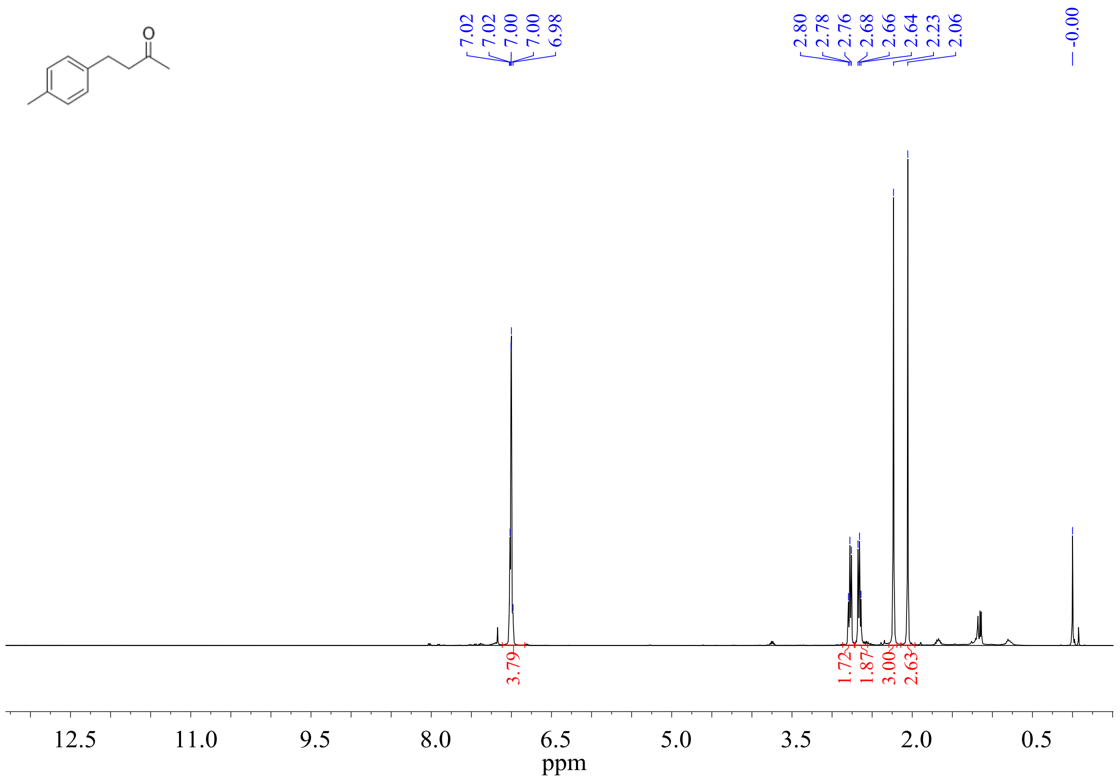

Figure S130. ^{1}H NMR spectrum of **6c** synthesized in-house (in $CDCl_3$).

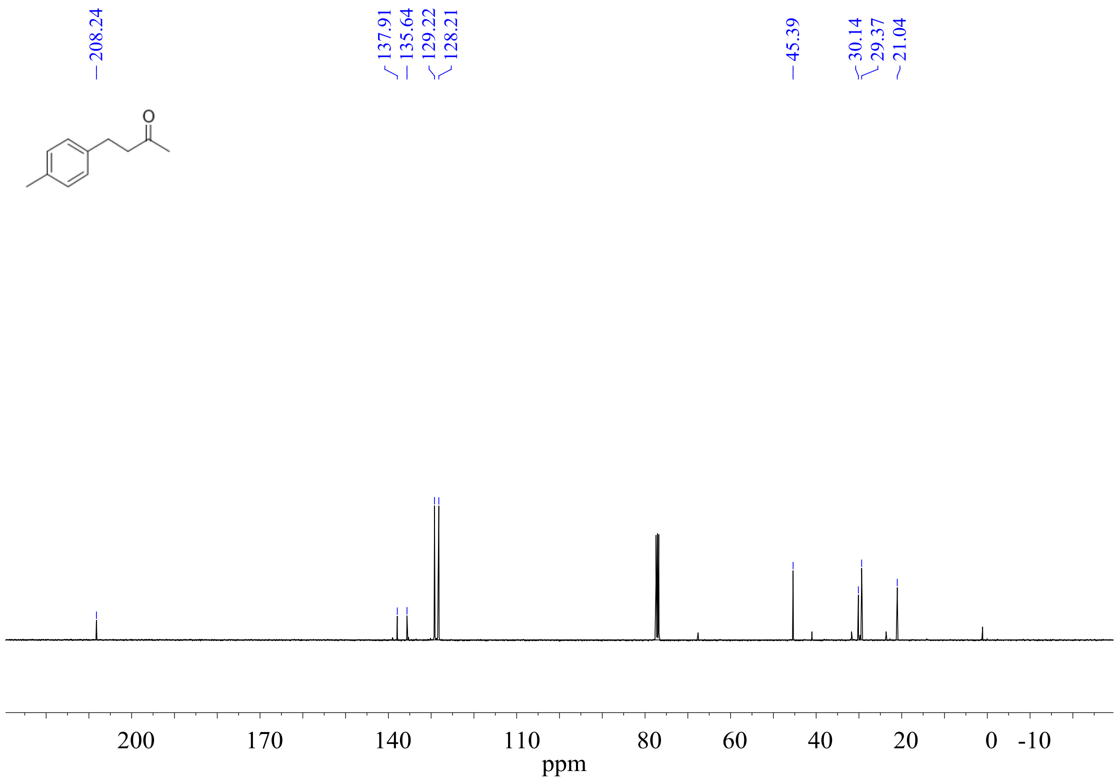

Figure S131. ^{13}C NMR of **6c** synthesized in-house (in $CDCl_3$).

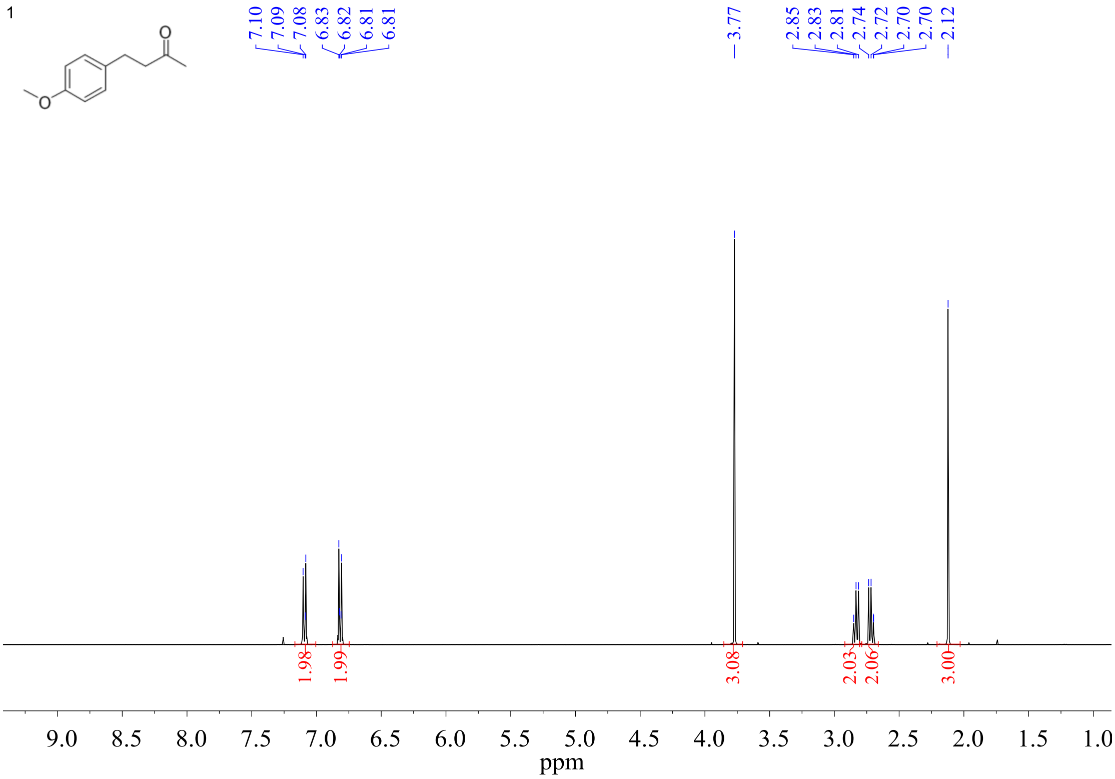

Figure S132. ^{1}H NMR spectrum of **7c** synthesized in-house (in $CDCl_3$).

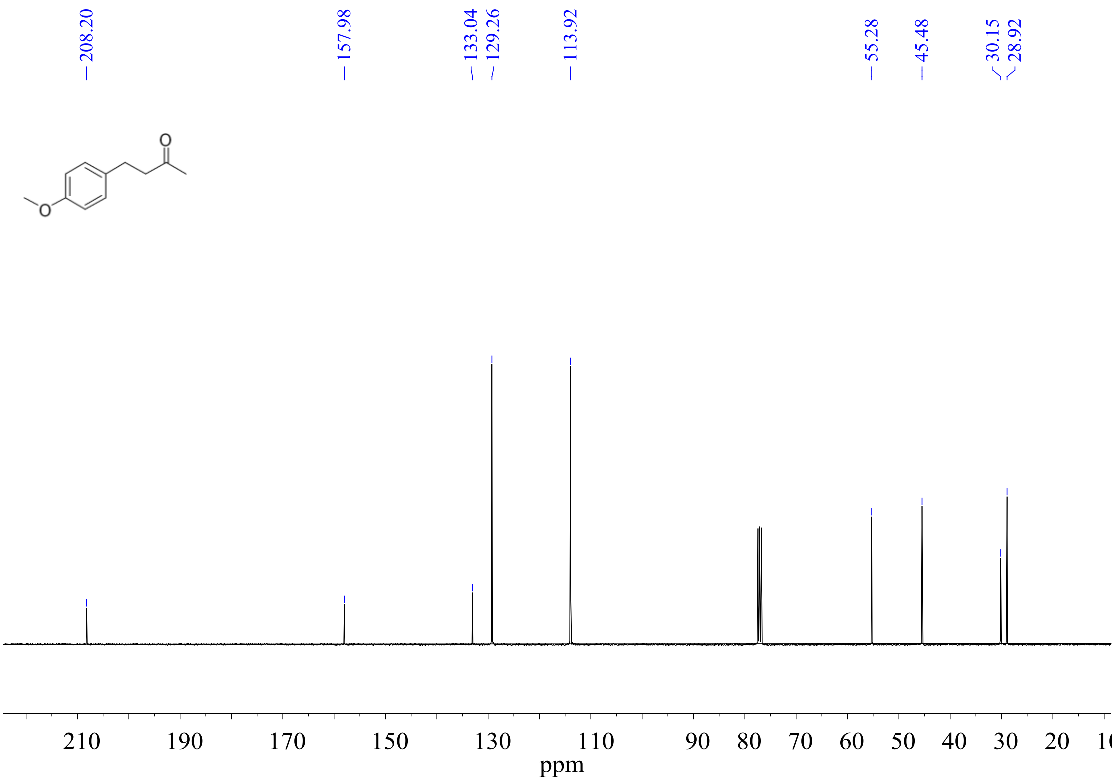

Figure S133. ^{13}C NMR of **7c** synthesized in-house (in $CDCl_3$).

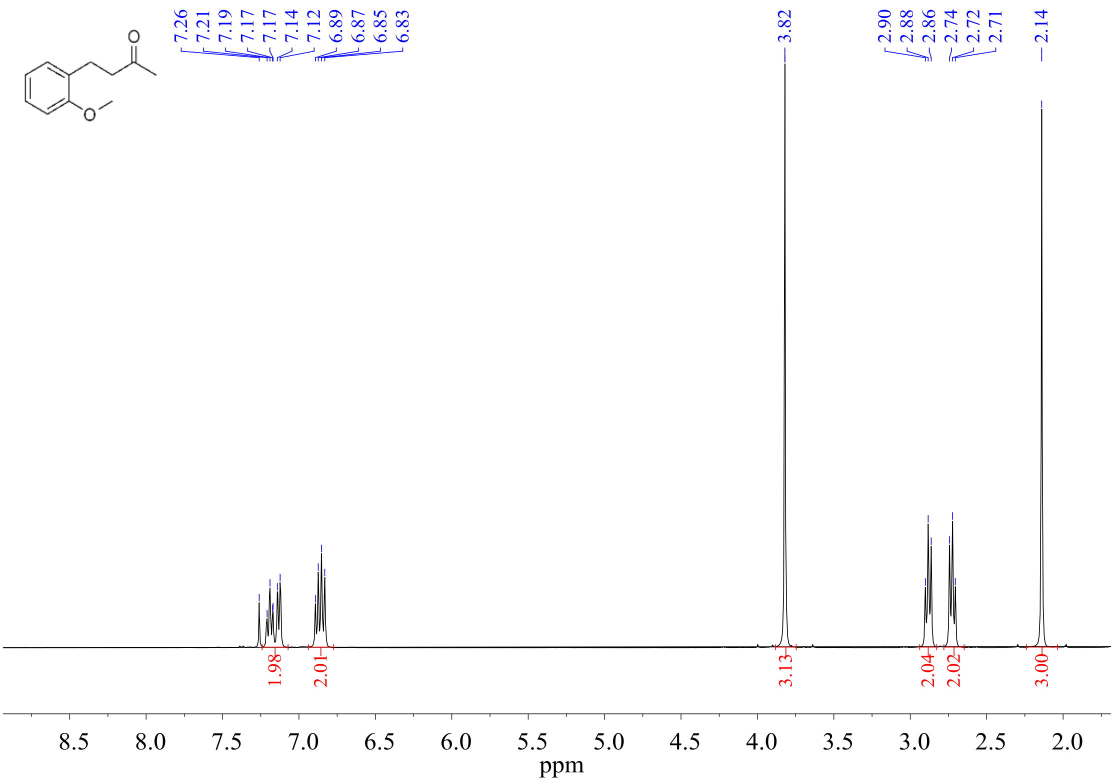

Figure S134. ^{1}H NMR of **8c** synthesized in-house (in $CDCl_3$).

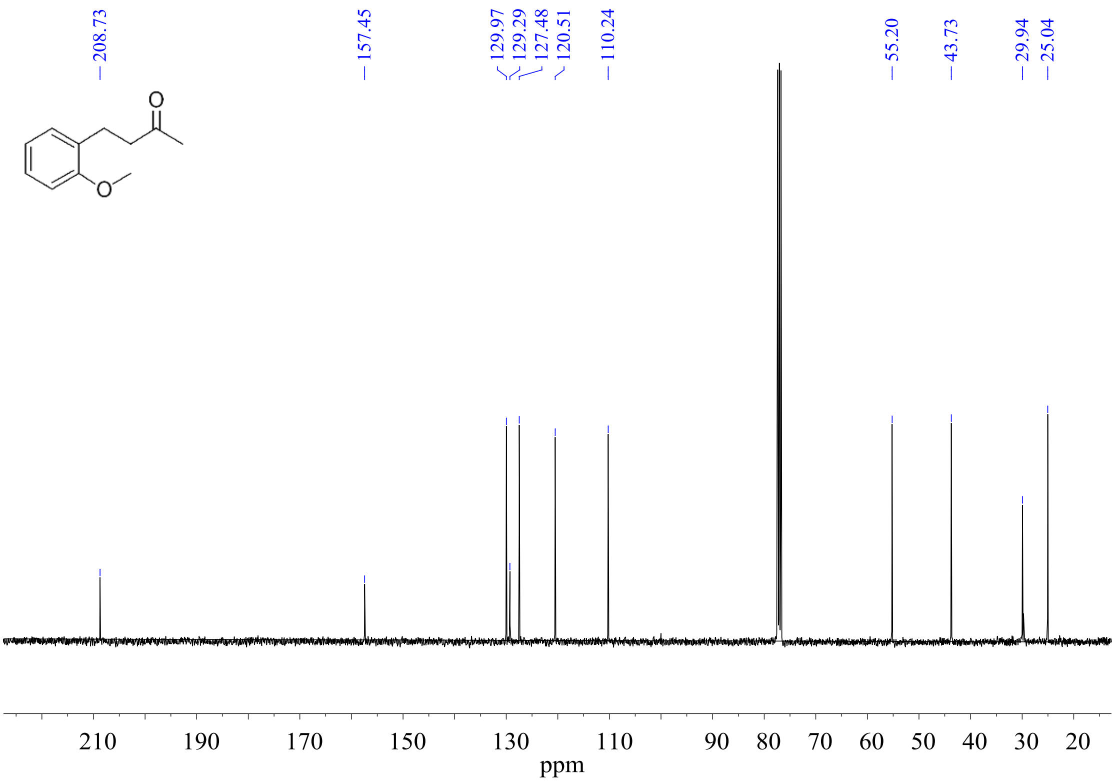

Figure S135. ^{13}C NMR of **8c** synthesized in-house (in $CDCl_3$).

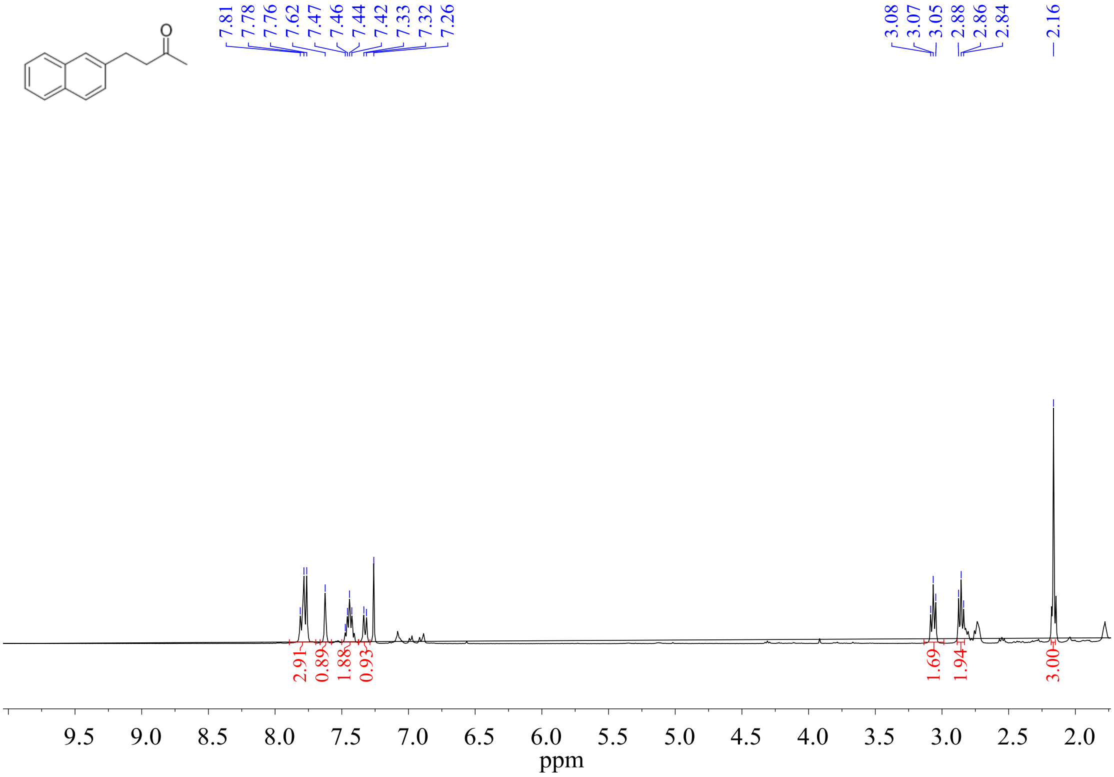

Figure S136. ^{1}H NMR of **10c** synthesized in-house (in $CDCl_3$).

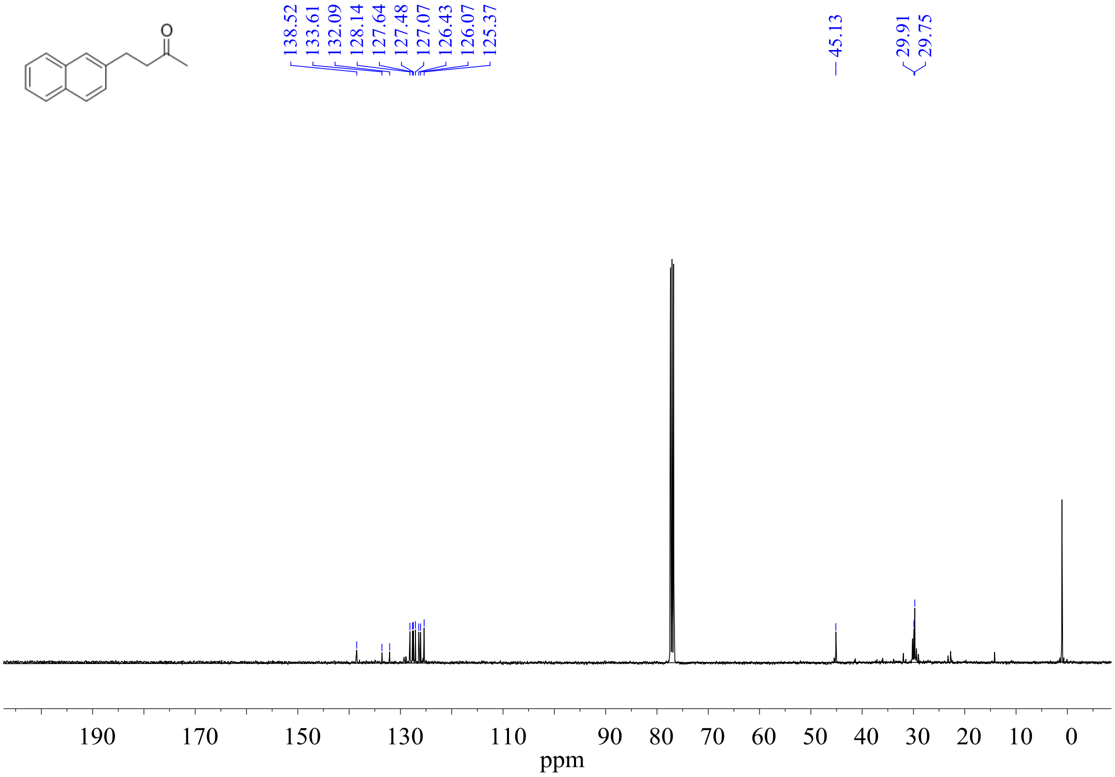

Figure S137. ^{13}C NMR of **10c** synthesized in-house (in $CDCl_3$).

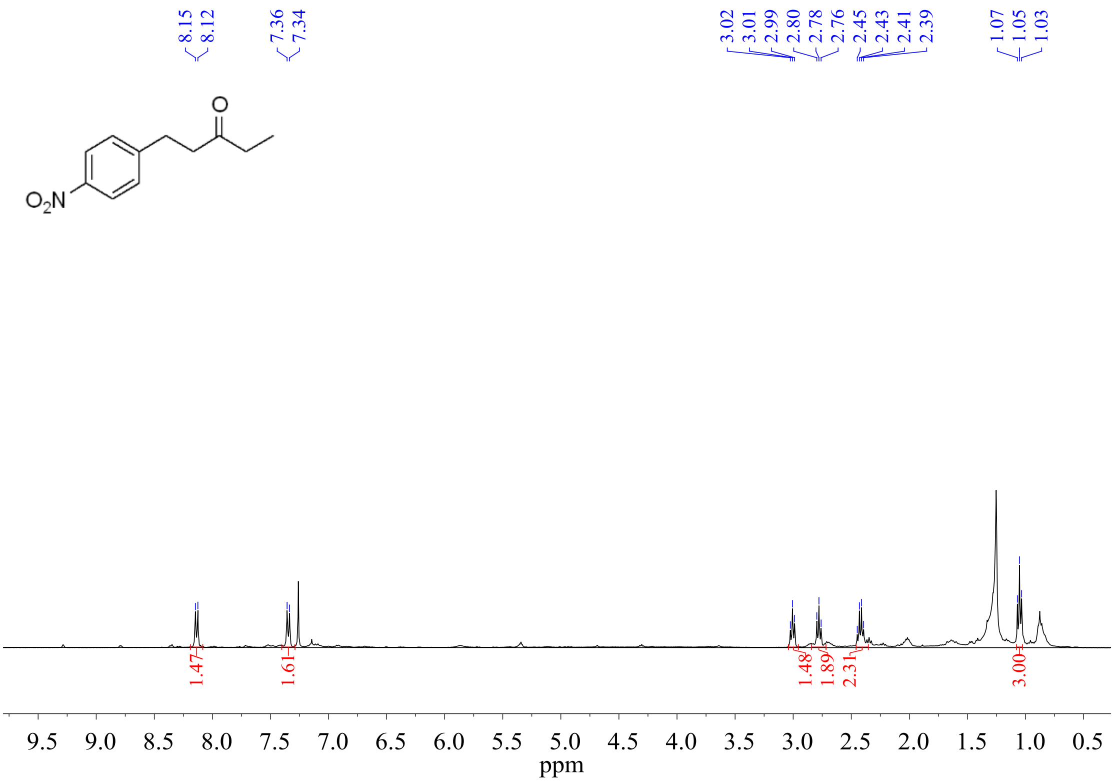

Figure S138. ^{1}H NMR of **15c** synthesized in-house (in $CDCl_3$).

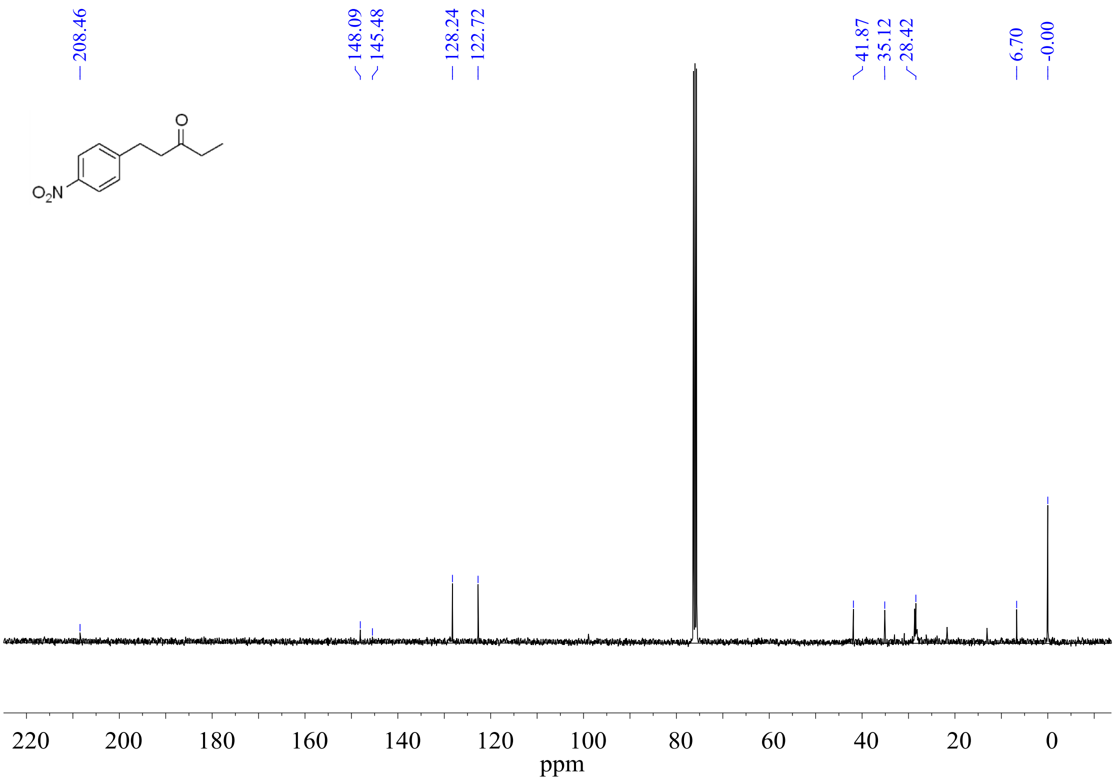

Figure S139. ^{13}C NMR of **15c** synthesized in-house (in $CDCl_3$).

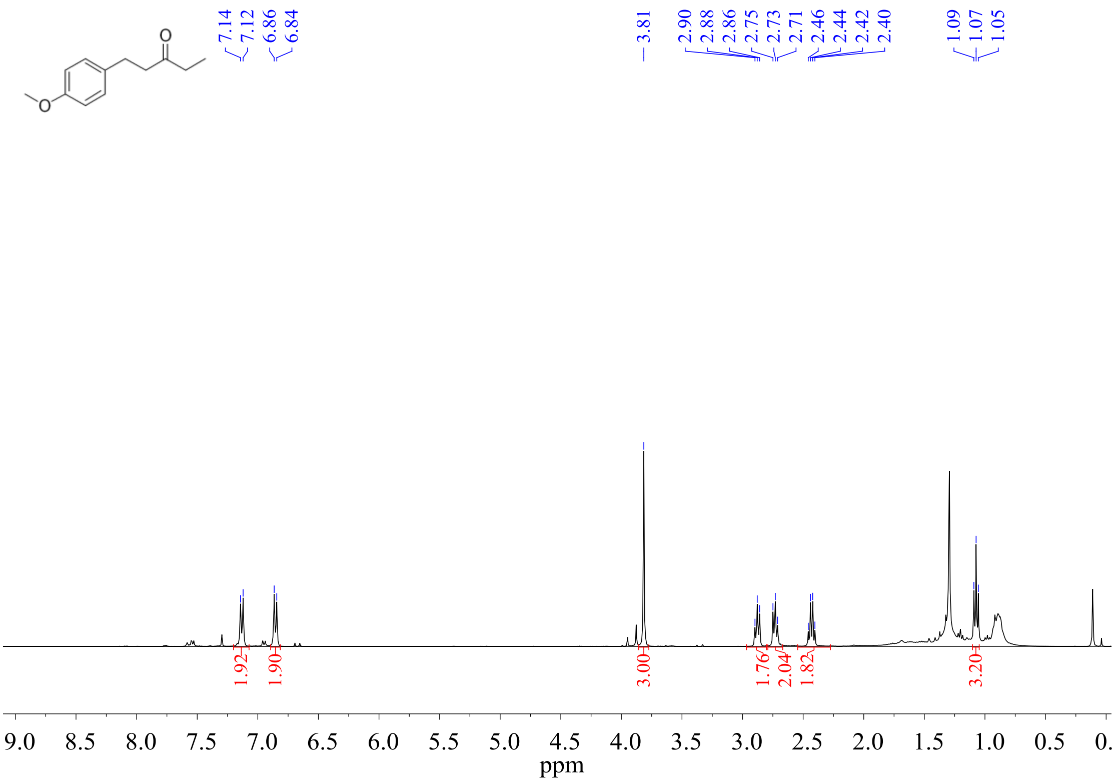

Figure S140. ^{1}H NMR of **17c** synthesized in-house (in $CDCl_3$).

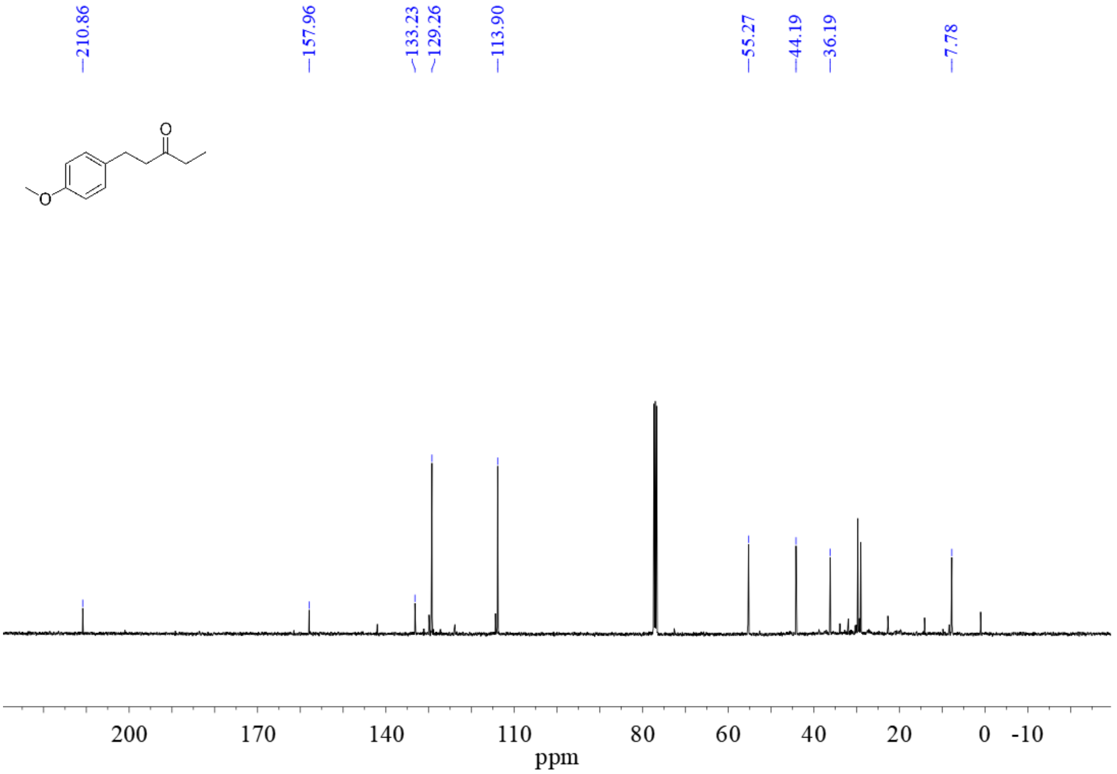

Figure S141. ^{13}C NMR of **17c** synthesized in-house (in $CDCl_3$).

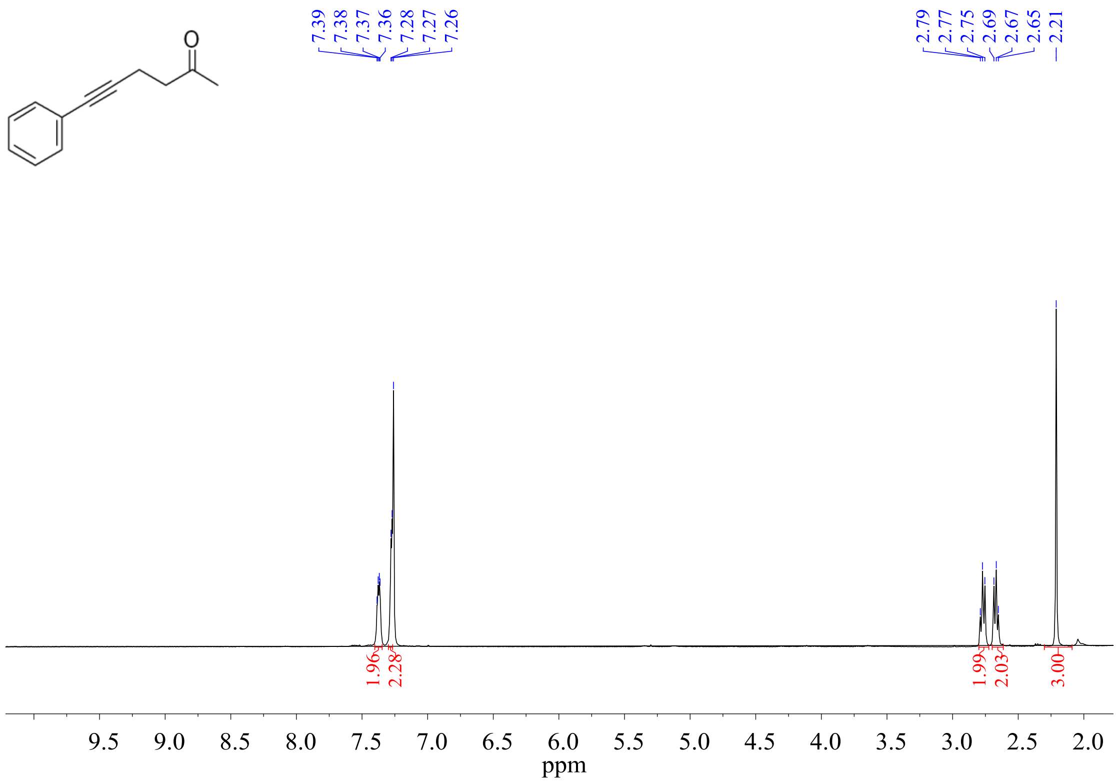

Figure S142. ^{1}H NMR of **21c** synthesized in-house (in $CDCl_3$).

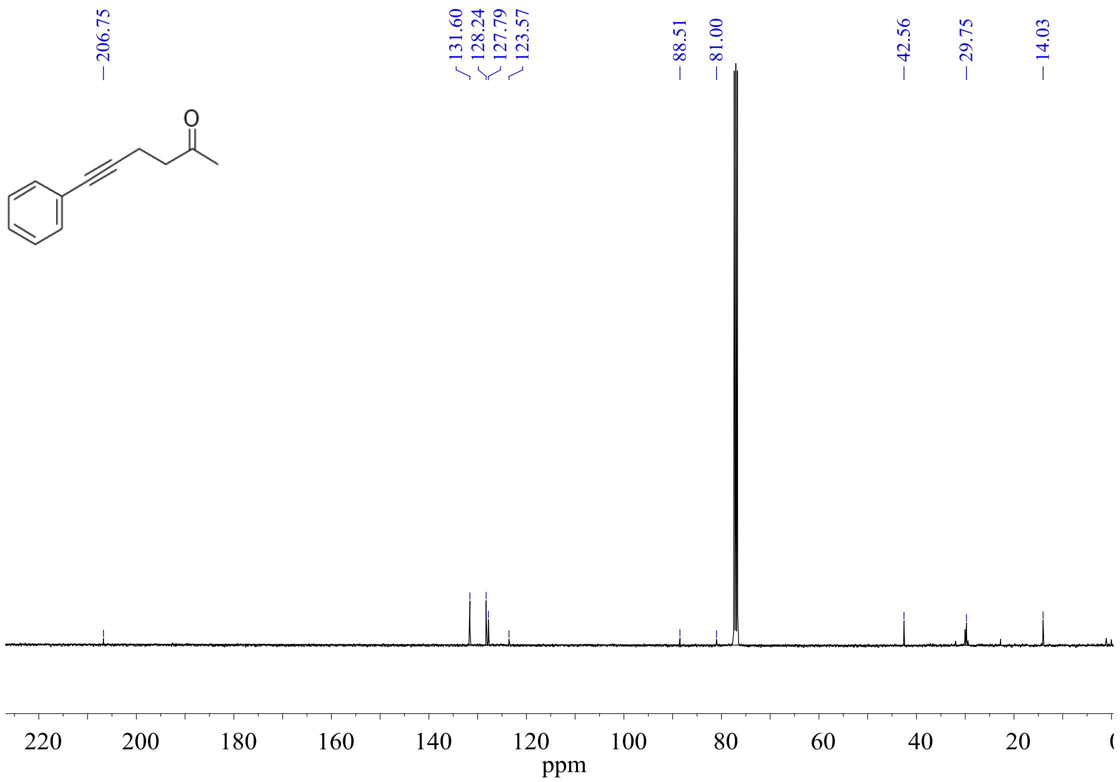

Figure S143. ^{13}C NMR of **21c** synthesized in-house (in $CDCl_3$).

Table S5. NMR data of the corresponding substrates and product standards synthesized in-house.

Compound	NMR data
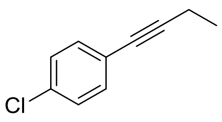	**^{1}H NMR (400 MHz, $CDCl_3$):** δ 7.28 (td, *J* = 8.6, 6.5 Hz, 4H), 2.40 (q, *J* = 7.5 Hz, 2H), 1.23 (t, *J* = 7.5 Hz, 3H). **^{13}C NMR (101 MHz, $CDCl_3$):** δ 134.24 (s), 133.44 (s), 132.79 (s), 129.11 (s), 128.50 (s), 122.62 (s), 92.76 (s), 78.95 (s), 13.84 (s), 13.14 (s).
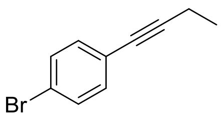	**^{1}H NMR (400 MHz, $CDCl_3$):** δ 7.51 (dd, *J* = 21.0, 8.3 Hz, 4H), 2.44 (q, *J* = 7.5 Hz, 2H), 1.25 (t, *J* = 7.5 Hz, 3H). **^{13}C NMR (101 MHz, $CDCl_3$):** δ 133.04 (s), 131.43 (s), 123.04 (s), 121.60 (s), 92.97 (s), 78.94 (s), 13.81 (s), 13.15 (s).
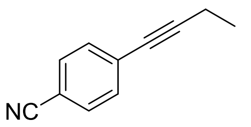	**^{1}H NMR (400 MHz, $CDCl_3$):** δ 7.56 (d, *J* = 8.2 Hz, 2H), 7.45 (d, *J* = 8.2 Hz, 2H), 2.44 (q, *J* = 7.5 Hz, 2H), 1.24 (t, *J* = 7.5 Hz, 3H). **^{13}C NMR (101 MHz, $CDCl_3$):** δ 132.01 (d, *J* = 17.7 Hz), 129.12 (s), 118.65 (s), 110.83 (s), 96.83 (s), 78.78 (s), 13.60 (s), 13.23 (s).
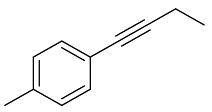	**^{1}H NMR (400 MHz, $CDCl_3$):** δ 7.30 – 7.24 (m, 2H), 7.08 (dd, *J* = 8.4, 0.6 Hz, 2H), 2.41 (dt, *J* = 12.5, 5.0 Hz, 2H), 2.32 (s, 3H), 1.25 – 1.20 (m, 3H). **^{13}C NMR (101 MHz, $CDCl_3$):** δ 137.46 (s), 131.41 (s), 128.96 (s), 120.93 (s), 90.86 (s), 79.90 (s), 21.41 (s), 14.01 (s), 13.13 (s).
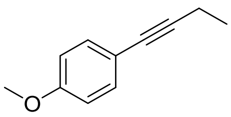	**^{1}H NMR (400 MHz, $CDCl_3$):** δ 7.62 – 6.41 (m, 4H), 3.79 (s, 3H), 2.40 (q, J = 7.5 Hz, 2H), 1.22 (t, J = 7.5 Hz, 3H). **^{13}C NMR (101 MHz, $CDCl_3$):** δ 159.02 (s), 132.87 (s), 116.21 (s), 113.83 (s), 90.05 (s), 79.59 (s), 55.27 (s), 14.07 (s), 13.12 (s).
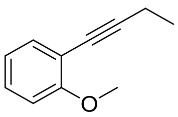	**^{1}H NMR (400 MHz, $CDCl_3$):** δ 7.48 – 7.09 (m, 2H), 6.86 (dd, *J* = 16.0, 8.0 Hz, 2H), 3.86 (s, 3H), 2.48 (q, *J* = 7.5 Hz, 2H), 1.25 (t, *J* = 7.5 Hz, 3H). **^{13}C NMR (101 MHz, $CDCl_3$):** δ 159.80 (s), 133.73 (s), 128.93 (s), 120.43 (s), 113.09 (s), 110.55 (s), 95.91 (s), 75.95 (s), 55.81 (s), 14.02 (s), 13.51 (s).
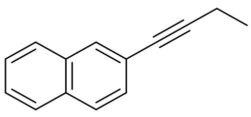	**^{1}H NMR (400 MHz, $CDCl_3$):** δ 7.90 (s, 1H), 7.76 (dd, *J* = 17.1, 8.8 Hz, 3H), 7.57 – 7.36 (m, 2H), 2.47 (q, *J* = 7.5 Hz, 2H), 1.27 (t, *J* = 7.5 Hz, 3H). **^{13}C NMR (101 MHz, $CDCl_3$):** δ 133.10 (s), 132.50 (s), 131.06 (s), 128.76 (s), 127.98 – 127.46 (m), 126.32 (d, *J* = 11.1 Hz), 121.39 (s), 92.11 (s), 80.27 (s), 14.00 (s), 13.26 (s).

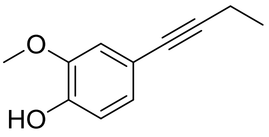	**^{1}H NMR (400 MHz, $CDCl_3$):** δ 6.97 – 6.88 (m, 2H), 6.82 (d, J = 8.1 Hz, 1H), 5.65 (s, 1H), 3.87 (s, 3H), 2.40 (q, J = 7.5 Hz, 2H), 1.23 (t, J = 7.5 Hz, 3H).<br>**^{13}C NMR (101 MHz, $CDCl_3$)**: δ 146.07 (s), 145.58 (s), 125.30 (s), 115.63 (s), 114.37 (s), 113.84 (s), 89.57 (s), 79.84 (s), 55.94 (s), 14.04 (s), 13.08 (s).
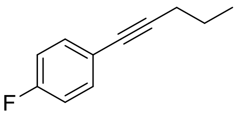	**^{1}H NMR (400 MHz, $CDCl_3$):** δ 7.40 (dd, *J* = 8.6, 5.5 Hz, 2H), 7.01 (t, *J* = 8.7 Hz, 2H), 2.41 (t, *J* = 7.0 Hz, 2H), 1.66 (dd, *J* = 14.5, 7.2 Hz, 2H), 1.08 (t, *J* = 7.4 Hz, 3H).<br>**^{13}C NMR (101 MHz, $CDCl_3$):** δ 163.27 (s), 133.31 (d, *J* = 8.2 Hz), 115.48 (s), 115.26 (s), 89.86 (s), 79.65 (s), 21.88 (s), 21.21 (s), 13.50 (s).
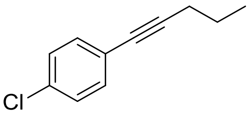	**^{1}H NMR (400 MHz, $CDCl_3$):** δ 7.32 (dd, *J* = 28.8, 8.5 Hz, 4H), 2.42 (t, *J* = 7.0 Hz, 2H), 1.67 (dd, *J* = 14.4, 7.2 Hz, 2H), 1.09 (t, *J* = 7.4 Hz, 3H).<br>**^{13}C NMR (101 MHz, $CDCl_3$):** δ 133.42 (s), 132.79 (s), 128.49 (s), 122.65 (s), 91.34 (s), 79.72 (s), 22.17 (s), 21.42 (s), 13.57 (s).
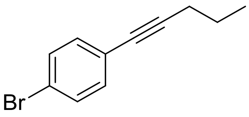	**^{1}H NMR (400 MHz, $CDCl_3$):** δ 7.39 (d, *J* = 8.3 Hz, 2H), 7.24 (d, *J* = 8.2 Hz, 2H), 2.36 (t, *J* = 7.0 Hz, 2H), 1.62 (dd, *J* = 14.4, 7.2 Hz, 2H), 1.03 (t, *J* = 7.3 Hz, 3H).<br>**^{13}C NMR (101 MHz, $CDCl_3$):** δ 133.05 (s), 131.42 (s), 123.12 (s), 121.57 (s), 91.57 (s), 79.79 (s), 65.50 (s), 22.15 (s), 21.45 (s), 13.59 (s).
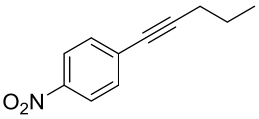	**^{1}H NMR (400 MHz, $CDCl_3$):** δ 8.15 (d, *J* = 8.8 Hz, 2H), 7.51 (d, *J* = 8.7 Hz, 2H), 2.44 (t, *J* = 7.0 Hz, 2H), 1.66 (dd, *J* = 14.5, 7.2 Hz, 2H), 1.07 (t, *J* = 7.4 Hz, 3H).<br>**^{13}C NMR (101 MHz, $CDCl_3$):** δ 146.56 (s), 132.23 (s), 131.21 (s), 123.45 (s), 96.61 (s), 79.45 (s), 21.92 (s), 21.52 (s), 13.53 (s).
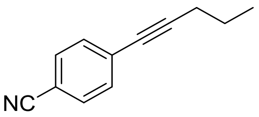	**^{1}H NMR (400 MHz, $CDCl_3$):** δ 7.51 (dd, *J* = 43.0, 8.4 Hz, 4H), 2.41 (t, *J* = 7.0 Hz, 2H), 1.64 (dd, *J* = 14.5, 7.2 Hz, 2H), 1.05 (t, *J* = 7.4 Hz, 3H).<br>**^{13}C NMR (101 MHz, $CDCl_3$):** δ 138.54 (s), 133.18 (s), 132.02 (d, *J* = 19.1 Hz), 129.17 (s), 118.66 (s), 110.81 (s), 95.52 (s), 79.61 (s), 21.96 (s), 21.49 (s), 13.56 (s).
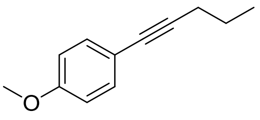	**^{1}H NMR (400 MHz, CDCl3):** δ 7.44 (d, *J* = 8.4 Hz, 2H), 7.29 (d, *J* = 8.4 Hz, 2H), 2.41 (t, *J* = 7.0 Hz, 2H), 1.66 (dd, *J* = 14.5, 7.2 Hz, 2H), 1.08 (t, *J* = 7.4 Hz, 3H).<br>**^{13}C NMR (101 MHz, $CDCl_3$):** δ 159.03 (s), 138.22 (s), 132.89 (s), 116.35 (d, *J* = 7.3 Hz), 113.83 (s), 88.61 (s), 80.46 (s), 55.24 (s), 22.38 (s), 21.45 (s), 13.61 (s).

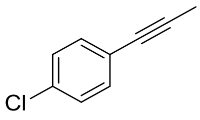	**^{1}H NMR (400 MHz, $CDCl_3$):** δ 7.36 (d, *J* = 8.6 Hz, 2H), 7.30 (d, *J* = 8.6 Hz, 2H), 2.08 (s, 3H).<br>**^{13}C NMR (101 MHz, $CDCl_3$):** δ 133.50 (s), 132.77 (s), 128.55 (s), 122.62 (s), 86.96 (s), 78.79 (s), 4.32 (s).
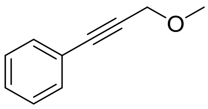	**^{1}H NMR (400 MHz, $CDCl_3$):** δ 7.44 (d, *J* = 3.3 Hz, 2H), 7.31 (d, *J* = 5.0 Hz, 3H), 4.32 (s, 2H), 3.45 (d, *J* = 0.5 Hz, 3H).<br>**^{13}C NMR (101 MHz, $CDCl_3$):** δ 131.78 (s), 128.42 (d, *J* = 13.3 Hz), 122.74 (s), 86.44 (s), 85.06 (s), 60.39 (s), 57.62 (s).
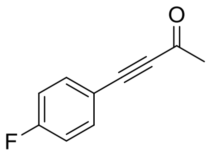	**^{1}H NMR (400 MHz, $CDCl_3$):** δ 7.67 – 7.48 (m, 2H), 7.09 (dd, *J* = 8.9, 8.5 Hz, 2H), 2.45 (s, 3H).<br>**^{13}C NMR (101 MHz, $CDCl_3$):** δ 184.47 (s), 165.28 (s), 162.75 (s), 135.38 (d, *J* = 8.9 Hz), 116.33 (s), 116.10 (s), 89.23 (s), 88.21 (s), 32.71 (s).
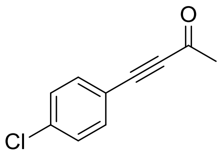	**^{1}H NMR (400 MHz, $CDCl_3$):** δ 7.54 – 7.48 (m, 2H), 7.40 – 7.34 (m, 2H), 2.45 (s, 3H).<br>**^{13}C NMR (101 MHz, $CDCl_3$):** δ 184.36 (s), 137.17 (s), 134.25 (s), 129.13 (s), 118.40 (s), 88.88 (d, *J* = 11.2 Hz), 32.72 (s).
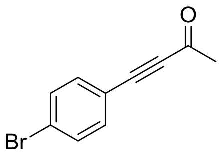	**^{1}H NMR (400 MHz, $CDCl_3$):** δ 7.57 – 7.49 (m, 2H), 7.46 – 7.39 (m, 2H), 2.45 (s, 3H).<br>**^{13}C NMR (101 MHz, $CDCl_3$):** δ 184.31 (s), 134.33 (s), 132.06 (s), 125.55 (s), 118.85 (s), 89.04 (s), 88.82 (s), 32.72 (s).
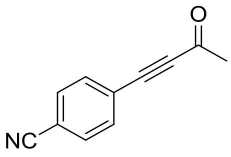	**^{1}H NMR (400 MHz, $CDCl_3$):** δ 7.76 – 7.54 (m, 4H), 2.48 (s, 3H).<br>**^{13}C NMR (101 MHz, $CDCl_3$):** δ 183.96 (s), 133.26 (s), 132.28 (s), 124.77 (s), 117.87 (s), 114.06 (s), 90.61 (s), 86.85 (s), 32.75 (s).
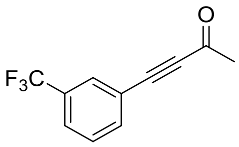	**^{1}H NMR (400 MHz, $CDCl_3$):** δ 7.85 (s, 1H), 7.74 (dt, *J* = 17.5, 8.6 Hz, 2H), 7.56 (t, *J* = 7.8 Hz, 1H), 2.50 (s, 3H).<br>**^{13}C NMR (101 MHz, $CDCl_3$):** δ 184.16 (s), 135.96 (s), 130.18 – 129.25 (m), 129.25 – 129.10 (m), 127.19 (d, *J* = 3.5 Hz), 120.98 (s), 88.84 (s), 87.68 (s), 32.69 (s).
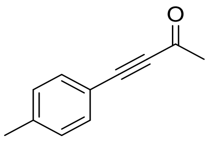	**^{1}H NMR (400 MHz, $CDCl_3$):** δ 7.55 – 7.46 (m, 2H), 7.27 – 7.18 (m, 2H), 2.48 (s, 3H), 2.42 (s, 3H).<br>**^{13}C NMR (101 MHz, $CDCl_3$):** δ 184.69 (s), 141.50 (s), 133.11 (s), 129.46 (s), 116.79 (s), 91.05 (s), 88.19 (s), 32.72 (s), 21.74 (s).

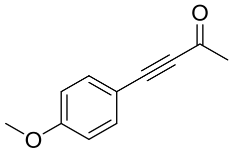	**^{1}H NMR (400 MHz, CDCl$_3$):** δ 7.51 (t, *J* = 8.1 Hz, 2H), 6.91 (dd, *J* = 16.0, 9.5 Hz, 2H), 3.84 (s, 3H), 2.45 (d, *J* = 12.0 Hz, 3H).<br>**^{13}C NMR (101 MHz, CDCl$_3$):** δ 184.66 (s), 161.70 (s), 135.14 (s), 120.61 (s), 114.40 (s), 91.53 (s), 88.27 (s), 55.44 (s), 32.66 (s).
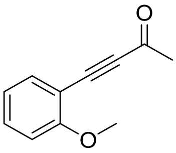	**^{1}H NMR (400 MHz, CDCl$_3$):** δ 7.32 – 6.97 (m, 4H), 3.81 (s, 3H), 2.45 (s, 3H).<br>**^{13}C NMR (101 MHz, CDCl$_3$):** δ 184.74 (s), 161.48 (s), 135.05 (s), 132.51 (s), 120.62 (s), 110.86 (s), 109.17 (s), 87.64 (s), 55.87 (s), 32.80 (s).
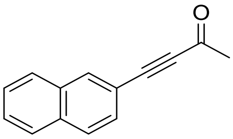	**^{1}H NMR (400 MHz, CDCl$_3$):** δ 8.17 (s, 1H), 7.86 (dd, *J* = 7.6, 3.1 Hz, 3H), 7.67 – 7.49 (m, 3H), 2.53 (s, 3H).<br>^{13}C NMR (101 MHz, CDCl$_3$) δ 184.61 (s), 134.38 (s), 133.93 (s), 132.68 (s), 128.70 – 127.77 (m), 127.07 (s), 117.11 (s), 90.86 (s), 88.58 (s), 32.82 (s).
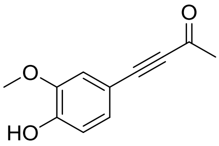	**^{1}H NMR (400 MHz, CDCl$_3$):** δ 7.62 (dd, *J* = 12.4, 0.6 Hz, 1H), 7.30 (d, *J* = 6.5 Hz, 1H), 6.94 – 6.69 (m, 1H), 5.78 (d, *J* = 12.4 Hz, 1H), 3.98 – 3.74 (m, 3H), 2.22 (s, 3H).<br>**^{13}C NMR (101 MHz, CDCl$_3$):** δ 197.11 (s), 160.22 (s), 151.16 (s), 144.24 (s), 130.23 (s), 122.18 (d, *J* = 3.6 Hz), 111.05 (s), 89.46 (s), 56.27 (s), 28.25 (s).
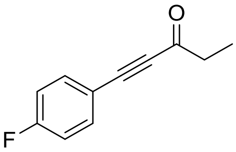	**^{1}H NMR (400 MHz, CDCl$_3$):** δ 7.58 (dd, *J* = 8.3, 5.5 Hz, 2H), 7.08 (t, *J* = 8.5 Hz, 2H), 2.69 (q, *J* = 7.3 Hz, 2H), 1.20 (d, *J* = 7.3 Hz, 3H).<br>**^{13}C NMR (101 MHz, CDCl$_3$):** δ 188.47 (s), 165.22 (s), 162.70 (s), 135.32 (d, *J* = 8.8 Hz), 116.30 (s), 116.08 (s), 89.54 (s), 87.58 (s), 38.81 (s), 29.73 (s), 8.15 (s).
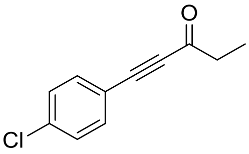	**^{1}H NMR (400 MHz, CDCl$_3$):** δ 7.50 (d, *J* = 8.4 Hz, 2H), 7.36 (d, *J* = 8.4 Hz, 2H), 2.69 (q, *J* = 7.4 Hz, 2H), 1.21 (t, *J* = 7.4 Hz, 3H).<br>**^{13}C NMR (101 MHz, CDCl$_3$):** δ 188.34 (s), 137.05 (s), 134.21 (s), 129.11 (s), 118.53 (s), 89.13 (s), 88.33 (s), 38.83 (s), 8.10 (s).
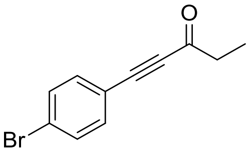	**^{1}H NMR (400 MHz, CDCl$_3$):** δ 7.75 – 7.29 (m, 4H), 2.72 (q, *J* = 7.4 Hz, 2H), 1.24 (t, *J* = 7.4 Hz, 3H).<br>**^{13}C NMR (101 MHz, CDCl$_3$):** δ 188.34 (s), 134.31 (s), 132.04 (s), 125.44 (s), 118.99 (s), 89.15 (s), 88.43 (s), 38.83 (s), 8.10 (s).

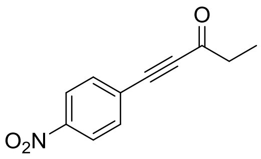	**^{1}H NMR (400 MHz, CDCl3):** δ 8.26 (d, *J* = 8.8 Hz, 2H), 7.73 (d, *J* = 8.8 Hz, 2H), 2.74 (q, *J* = 7.3 Hz, 2H), 1.26 – 1.22 (m, 3H).<br>**^{13}C NMR (101 MHz, $CDCl_3$):** δ 187.86 (s), 133.62 (s), 123.80 (s), 86.71 (s), 38.94 (s), 7.94 (s).
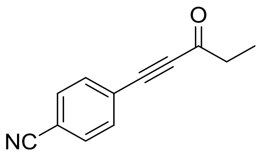	**^{1}H NMR (400 MHz, $CDCl_3$):** δ 7.65 (q, *J* = 8.2 Hz, 3H), 2.70 (q, *J* = 7.3 Hz, 2H), 1.29 – 1.08 (m, 3H).<br>**^{13}C NMR (101 MHz, $CDCl_3$):** δ 187.91 (s), 133.24 (s), 132.27 (s), 124.87 (s), 117.90 (s), 113.92 (s), 90.09 (s), 87.18 (s), 38.91 (s), 7.95 (s).
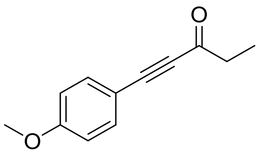	**^{1}H NMR (400 MHz, $CDCl_3$):** δ 7.52 (d, *J* = 8.9 Hz, 2H), 6.89 (d, *J* = 8.8 Hz, 2H), 3.83 (s, 3H), 2.67 (q, *J* = 7.4 Hz, 2H), 1.21 (t, *J* = 7.4 Hz, 3H).<br>**^{13}C NMR (101 MHz, $CDCl_3$):** δ 188.72 (s), 161.61 (s), 135.10 (s), 114.37 (s), 111.87 (s), 91.83 (s), 87.57 (s), 55.45 (s), 38.72 (s), 8.31 (s).
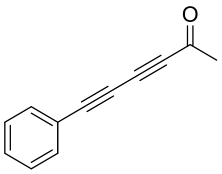	**^{1}H NMR (400 MHz, CDCl3):** δ 7.54 (d, *J* = 7.3 Hz, 2H), 7.40 (dt, *J* = 31.2, 7.3 Hz, 3H), 2.41 (s, 3H).<br>**^{13}C NMR (101 MHz, $CDCl_3$):** δ 183.43 (s), 133.04 (s), 130.55 (s), 128.68 (s), 120.19 (s), 86.57 (s), 78.77 (s), 75.09 (s), 72.14 (s), 32.66 (s).
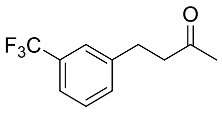	**^{1}H NMR (400 MHz, $CDCl_3$):** δ 7.48 (d, *J* = 7.6 Hz, 2H), 7.42 (d, *J* = 5.9 Hz, 2H), 2.99 (t, *J* = 7.5 Hz, 2H), 2.83 (t, *J* = 7.6 Hz, 2H), 2.19 (s, 3H).<br>**^{13}C NMR (101 MHz, $CDCl_3$)** δ 207.28 (s), 141.97 (s), 131.89 (s), 128.94 (s), 125.04 (s), 123.03 (s), 44.74 (s), 30.10 (s), 29.35 (s).
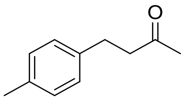	**^{1}H NMR (400 MHz, $CDCl_3$):** δ 7.11 – 6.83 (m, 4H), 2.78 (t, *J* = 7.5 Hz, 2H), 2.71 – 2.56 (m, 2H), 2.23 (s, 3H), 2.06 (s, 3H).<br>**^{13}C NMR (101 MHz, $CDCl_3$):** δ 208.24 (s), 137.91 (s), 135.64 (s), 129.22 (s), 128.21 (s), 45.39 (s), 30.14 (s), 29.37 (s), 21.04 (s).
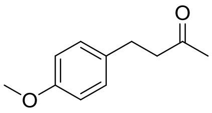	**^{1}H NMR (400 MHz, $CDCl_3$):** δ 7.17 – 7.01 (m, 2H), 6.87 – 6.75 (m, 2H), 3.77 (s, 3H), 2.92 – 2.79 (m, 2H), 2.71 (dd, *J* = 11.1, 4.2 Hz, 2H), 2.12 (s, 3H).<br>**^{13}C NMR (101 MHz, $CDCl_3$):** δ 208.20 (s), 157.98 (s), 133.04 (s), 129.26 (s), 113.92 (s), 55.28 (s), 45.48 (s), 30.15 (s), 28.92 (s).
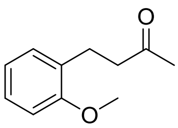	**^{1}H NMR (400 MHz, $CDCl_3$):** δ 7.24 – 7.07 (m, 2H), 6.86 (dd, *J* = 16.5, 7.9 Hz, 2H), 3.82 (s, 3H), 2.88 (t, *J* = 7.6 Hz, 2H), 2.72 (t, *J* = 7.6 Hz, 2H), 2.14 (s, 3H).<br>**^{13}C NMR (101 MHz, $CDCl_3$):** δ 208.73 (s), 157.45 (s), 129.97 (s), 129.29 (s), 127.48 (s), 120.51 (s), 110.24 (s), 55.20 (s), 43.73 (s), 29.94 (s), 25.04

	(s).
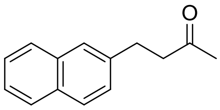	**^{1}H NMR (400 MHz, CDCl$_3$):** δ 7.89 – 7.70 (m, 3H), 7.62 (s, 1H), 7.45 (dd, *J* = 12.9, 6.7 Hz, 2H), 7.32 (d, *J* = 7.8 Hz, 1H), 3.07 (t, *J* = 7.6 Hz, 2H), 2.86 (t, *J* = 7.6 Hz, 2H), 2.16 (s, 3H).<br><br>**^{13}C NMR (101 MHz, CDCl$_3$):** δ 207.98 (s), 138.52 (s), 133.61 (s), 132.09 (s), 128.14 (s), 127.56 (d, *J* = 16.7 Hz), 127.07 (s), 126.43 (s), 126.07 (s), 125.37 (s), 45.13 (s), 29.83 (d, *J* = 16.7 Hz).
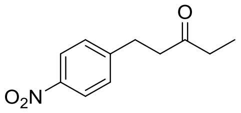	**^{1}H NMR (400 MHz, CDCl3):** 8.14 (d, J = 8.6 Hz, 1H), 7.35 (d, J = 8.5 Hz, 2H), 3.01 (t, J = 7.3 Hz, 1H), 2.78 (t, J = 7.3 Hz, 2H), 2.42 (q, J = 7.3 Hz, 2H), 1.05 (t, J = 7.3 Hz, 3H).<br><br>**^{13}C NMR (101 MHz, CDCl$_3$)**: 208.46 (s), 148.09 (s), 128.24 (s), 122.72 (s), 41.87 (s), 35.12 (s), 28.42 (s), 6.70 (s).
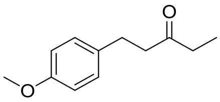	**^{1}H NMR (400 MHz, CDCl$_3$)**: δ 7.13 (d, *J* = 8.5 Hz, 2H), 6.85 (d, *J* = 8.5 Hz, 2H), 3.81 (s, 3H), 2.88 (t, *J* = 7.5 Hz, 2H), 2.73 (t, *J* = 7.5 Hz, 2H), 2.43 (q, *J* = 7.3 Hz,<br><br>1.07 (t, *J* = 7.3 Hz, 3H).<br><br>**^{13}C NMR (101 MHz, CDCl$_3$):** δ 210.86 (s), 157.96 (s), 133.23 (s), 129.26 (s), 113.90 (s), 55.27 (s), 44.19 (s), 36.19 (s), 7.78 (s).
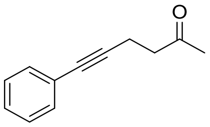	**^{1}H NMR (400 MHz, CDCl$_3$)**: 7.37 (dd, *J* = 6.3, 2.8 Hz, 2H), 7.28 (d, *J* = 3.4 Hz, 2H), 2.77 (t, *J* = 6.9 Hz, 2H), 2.67 (t, *J* = 6.8 Hz, 2H), 2.21 (s, 3H).<br><br>**^{13}C NMR (101 MHz, CDCl$_3$):** 206.75 (s), 131.60 (s), 128.24 (s), 127.79 (s), 123.57 (s), 81.00 (s), 42.56 (s), 29.75 (s), 14.03 (s).

Details of the gas chromatograph and temperature profiles

Substrates	T_R (min)	Temperature profile[a]
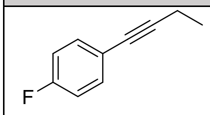	1-(but-1-yn-1-yl)-4-fluorobenzene: 5.074<br>4-(4-fluorophenyl) but-3-yn-2-one: 6.090<br>(E)-4-(4-fluorophenyl) but-3-en-2-one:6.685<br>4-(4-fluorophenyl) butan-2-one: 5.906	110 °C hold 0.5 min,<br>30 °C/min to<br>180 °C hold 0.5 min,<br>30 °C/min to<br>230 °C hold 1.3 min,<br>30 °C/min to<br>320 °C hold 1 min.
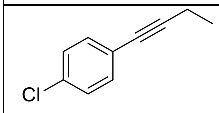	1-(but-1-yn-1-yl)-4-chlorobenzene: 6.498<br>4-(4-chlorophenyl) but-3-yn-2-one: 7.503<br>(E)-4-(4-chlorophenyl) but-3-en-2-one: 8.037<br>4-(4-chlorophenyl) butan-2-one:7.371	110 °C hold 0.5 min,<br>30 °C/min to<br>180 °C hold 0.5 min,<br>30 °C/min to<br>230 °C hold 1.3 min,<br>30 °C/min to<br>320 °C hold 2 min.
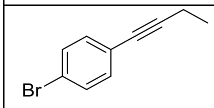	1-bromo-4-(but-1-yn-1-yl) benzene: 7.204<br>4-(4-bromophenyl) but-3-yn-2-one8.204<br>(E)-4-(4-bromophenyl) but-3-en-2-one: 8.786<br>4-(4-bromophenyl) butan-2-one:8.198	110 °C hold 0.5 min,<br>30 °C/min to<br>180 °C hold 0.5 min,<br>30 °C/min to<br>230 °C hold 1.3 min,<br>30 °C/min to<br>320 °C hold 2 min.
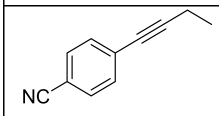	4-(but-1-yn-1-yl) benzonitrile: 7.285<br>4-(3-oxobut-1-yn-1-yl) benzonitrile: 8.217<br>(E)-4-(3-oxobut-1-en-1-yl) benzonitrile:8.828<br>4-(3-oxobutyl) benzonitrile: 8.312	110 °C hold 0.5 min,<br>30 °C/min to<br>180 °C hold 0.5 min,<br>30 °C/min to<br>230 °C hold 1.3 min,<br>30 °C/min to<br>320 °C hold 2 min.
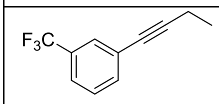	1-(but-1-yn-1-yl)-3-(trifluoromethyl)benzene: 5.050	110 °C hold 0.5 min,<br>30 °C/min to

	4-(3-(trifluoromethyl)phenyl)but-3-yn-2-one:5.907<br>(4-(3-(trifluoromethyl)phenyl)butan-2-one:5.807	180 °C hold 0.5 min, 30 °C/min to<br>230 °C hold 1.3 min, 30 °C/min to<br>320 °C hold 2 min.
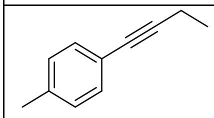	1-(but-1-yn-1-yl)-4-methylbenzene:5.933<br>4-(p-tolyl)but-3-yn-2-one: 7.158<br>(E)-4-(p-tolyl)but-3-en-2-one:7.624<br>4-(p-tolyl)butan-2-one:6.660	110 °C hold 0.5 min, 30 °C/min to<br>180 °C hold 0.5 min, 30 °C/min to<br>230 °C hold 1.3 min, 30 °C/min to<br>320 °C hold 2 min.
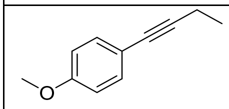	1-(but-1-yn-1-yl)-4-methoxybenzene: 7.060<br>4-(4-methoxyphenyl)but-3-yn-2-one: 8.282<br>(E)-4-(4-methoxyphenyl)but-3-en-2-one:8.718<br>4-(4-methoxyphenyl)butan-2-one: 7.674	110 °C hold 0.5 min, 30 °C/min to<br>180 °C hold 0.5 min, 30 °C/min to<br>230 °C hold 1.3 min, 30 °C/min to<br>320 °C hold 2 min.
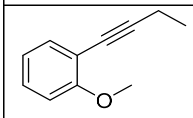	1-(but-1-yn-1-yl)-2-methoxybenzene: 6.735<br>4-(2-methoxyphenyl)but-3-yn-2-one:7.923<br>(E)-4-(2-methoxyphenyl)but-3-en-2-one:8.437<br>4-(2-methoxyphenyl)butan-2-one:7.593	110 °C hold 0.5 min, 30 °C/min to<br>180 °C hold 0.5 min, 30 °C/min to<br>230 °C hold 1.3 min, 30 °C/min to<br>320 °C hold 2 min.
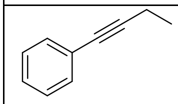	but-1-yn-1-ylbenzene:4.721<br>4-phenylbut-3-yn-2-one:6.420<br>(E)-4-phenylbut-3-en-2-one:7.014<br>4-phenylbutan-2-one:5.912	110 °C hold 0.5 min, 15 °C/min to<br>145 °C hold 2 min, 30 °C/min to<br>320 °C hold 0.5 min.
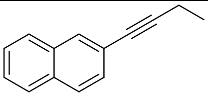	2-(but-1-yn-1-yl)naphthalene:9.177<br>4-(naphthalen-2-yl) but-3-yn-2-one:10.182	120 °C hold 0.5 min, 30 °C/min to<br>220 °C hold 1 min,

	(E)-4-(naphthalen-2-yl)but-3-en-2-one:10.707<br>4-(naphthalen-2-yl)butan-2-one:9.805	30 °C/min to<br>300 °C hold 2 min,<br>30 °C/min to<br>320 °C hold 0.5 min.
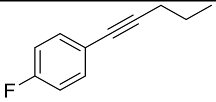	1-fluoro-4-(pent-1-yn-1-yl)benzene:5.349<br>1-(4-fluorophenyl)pent-1-yn-3-one:6.320<br>(E)-1-(4-fluorophenyl)pent-1-en-3-one: 6.754<br>1-(4-fluorophenyl)pentan-3-one:6.076	120 °C hold 0.5 min,<br>30 °C/min to<br>220 °C hold 1 min,<br>30 °C/min to<br>300 °C hold 2 min,<br>30 °C/min to<br>320 °C hold 0.5 min.
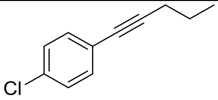	1-fluoro-4-(pent-1-yn-1-yl)benzene:6.609<br>1-(4-chlorophenyl)pent-1-yn-3-one:7.548<br>(E)-1-(4-chlorophenyl)pent-1-en-3-one:8.032<br>1-(4-chlorophenyl)pentan-3-one:7.357	120 °C hold 0.5 min,<br>30 °C/min to<br>220 °C hold 1 min,<br>30 °C/min to<br>300 °C hold 2 min,<br>30 °C/min to<br>320 °C hold 0.5 min.
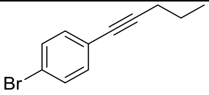	1-bromo-4-(pent-1-yn-1-yl)benzene:7.239<br>1-(4-bromophenyl)pent-1-yn-3-one:8.214<br>(E)-1-(4-bromophenyl)pent-1-en-3-one: 8.701<br>1-(4-bromophenyl)pentan-3-one:7.985	120 °C hold 0.5 min,<br>30 °C/min to<br>220 °C hold 1 min,<br>30 °C/min to<br>300 °C hold 2 min,<br>30 °C/min to<br>320 °C hold 0.5 min.
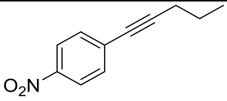	1-nitro-4-(pent-1-yn-1-yl)benzene:8.084<br>1-(4-nitrophenyl)pent-1-yn-3-one:8.856<br>(E)-1-(4- nitrophenyl)pent-1-en-3-one:9.193<br>1-(4- nitrophenyl)pentan-3-one: 8.084	120 °C hold 0.5 min,<br>30 °C/min to<br>220 °C hold 1 min,<br>30 °C/min to<br>300 °C hold 2 min,<br>30 °C/min to<br>320 °C hold 0.5 min.
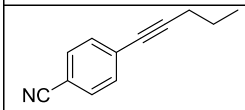	4-(pent-1-yn-1-yl)benzonitrile:7.300<br>4-(3-oxopent-1-yn-1-yl)benzonitrile:8.105	120 °C hold 0.5 min,<br>30 °C/min to<br>220 °C hold 1 min,

	(E)-4-(3-oxopent-1-en-1-yl)benzonitrile:8.724<br>4-(3-oxopentyl)benzonitrile:8.198	30 °C/min to<br>300 °C hold 2 min,<br>30 °C/min to<br>320 °C hold 0.5 min.
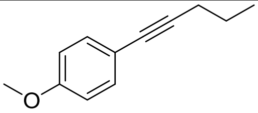	1- methoxy -4-(pent-1-yn-1-yl)benzene:7.080<br>1-(4- methoxyphenyl)pent-1-yn-3-one:8.121<br>(E)-1-(4- methoxy phenyl)pent-1-en-3-one:8.608<br>1-(4- methoxy phenyl)pentan-3-one:7.601	120 °C hold 0.5 min,<br>30 °C/min to<br>220 °C hold 1 min,<br>30 °C/min to<br>300 °C hold 2 min,<br>30 °C/min to<br>320 °C hold 0.5 min.
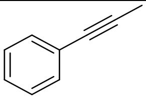	prop-1-yn-1-ylbenzene:4.670<br>3-phenylpropiolaldehyde:5.684	120 °C hold 0.5 min,<br>30 °C/min to<br>220 °C hold 1 min,<br>30 °C/min to<br>300 °C hold 2 min,<br>30 °C/min to<br>320 °C hold 0.5 min.
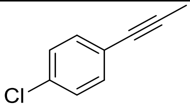	1-chloro-4-(prop-1-yn-1-yl)benzene:5.093<br>3-(4-chlorophenyl)propiolaldehyde:6.695	120 °C hold 0.5 min,<br>30 °C/min to<br>220 °C hold 1 min,<br>30 °C/min to<br>300 °C hold 2 min,<br>30 °C/min to<br>320 °C hold 0.5 min.
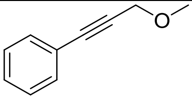	(3-methoxyprop-1-yn-1-yl)benzene:6.065<br>3-phenylpropiolaldehyde: 5.684<br>Cinnamaldehyde: 5.797<br>3-phenylpropanal: 6.308	120 °C hold 0.5 min,<br>30 °C/min to<br>220 °C hold 1 min,<br>30 °C/min to<br>300 °C hold 2 min,<br>30 °C/min to<br>320 °C hold 0.5 min.
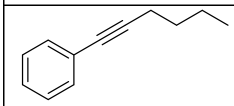	hex-1-yn-1-ylbenzene:6.640<br>1-phenylhex-1-yn-3-ol: 7.702	110 °C hold 0.5 min,<br>30 °C/min to<br>180 °C hold 0.5 min,

		30 °C/min to<br><br>230 °C hold 1.3 min,<br>30 °C/min to<br><br>320 °C hold 2 min.
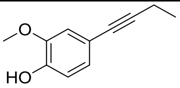	4-(but-1-yn-1-yl)-2-methoxyphenol:8.127<br><br>4-(4-hydroxy-3-methoxyphenyl)but-3-yn-2-one:10.272<br><br>4-(4-hydroxy-3-methoxyphenyl)butan-2-one:8.707	110 °C hold 0.5 min,<br>30 °C/min to<br><br>180 °C hold 0.5 min,<br>30 °C/min to<br><br>230 °C hold 1.3 min,<br>30 °C/min to<br><br>320 °C hold 2 min.
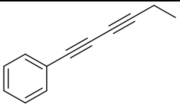	hexa-1,3-diyn-1-ylbenzene:7.629<br><br>6-phenylhexa-3,5-diyn-2-one: 8.332<br><br>6-phenylhex-5-yn-2-one: 7.897<br><br>6-phenylhexan-2-one:7.481	110 °C hold 0.5 min,<br>30 °C/min to<br><br>180 °C hold 0.5 min,<br>30 °C/min to<br><br>230 °C hold 1.3 min,<br>30 °C/min to<br><br>320 °C hold 2 min.

[a] Column A: SH-Rtx-1 column (30 m × 0.25 mm × 1 µm), FID, N_2 is the carrier gas.

References

[1] Shi, D. Liu, Z. Zhang, Z. Wei, S. Hao, C, *ChemCatChem* **2015**, 7, 1424-1426.

[2] Luridiana, A. Frongia, A. Scorciapino, M. A, *Adv. synth. cataly.* **2022**, 364, 124-131.

[3] Zhong Z. Y. Luo. Hongguang D. Jiaxi X. Pingfan L, *Chem. Sci.* **2019**, 10, 5056-5161.

[4] Stuart, D. R. Bertrand-Laperle, M. Burgess, K. M. N. Fagnou, K. *J. Am. Chem. Soc.* **2008**, 130, 16474-16475.

[5] Zhang, Y.H. Xie, Y. Yu, M. *Synthesis* **2011**, 17, 2803-2809.

[6] Ueoka, R. Bhushan, A. Probst, S. I. Bray, W. M. Lokey, R. S. Linington, R. G. Piel, J. J. A. C. *Angew. Chem. Int. Ed.* **2018**, 130, 14727-14731.

[7] Phadke, N. Findlater, M. *Molecules* **2015**, 20, 20195-205.

[8] Kouichi, W. Yusuke, M. Masataka, O. Zhou, B. Hiroaki, T. Motoi, K. *Org. Lett.* **2018**, 20, 5448-5451.

[9] Panteleev, J. Huang, R. Y. Lui, E. Lautens, M. *Org. Lett.* **2011**, 13, 5314-5317.

[10] Zhang, X. Lu, Z. Fu, C. Ma, S. Org. *Biomol. Chem.* **2009**, 7, 3258-3263.

[11] Sang, X. Tong, F. Zeng, Z. Wu, M. Yuan, B. Sun, Z. Sheng, X. Qu, G. Alcalde, M. Hollmann, F. Zhang, W. *Org. Lett.* **2022**, 24, 4252-4257.

[12] Fang, Z. Wills, M. *Org. Lett.* **2014**, 45, 374.

[13] Müller, A. Stürmer, R. Hauer, B. Rosche, B. *Angew. Chem. Int. Ed.* **2007**, 46, 3316-3318.

[14] Li, Y. Zhang, P. Sun, Z. Li, H. Ge, R. Sheng, X. Zhang, W. *Antioxidants*, **2022**, 11, 1044.

[15] Zhang, W. Fernández-Fueyo, E. Ni, Y. Schie, M. V. Gacs, J. Renirie, R. Wever, R. Mutti, F. G. Rother, D. Alcalde, M. *Nat. Catal.* **2019**, 1, 55-62.

[16] Weckbecker, A. Hummel, W. *Biocatalysis*, **2006**, 24, 380-389.

[17] Keinan, E. Hafeli, E. K. Seth, K. K. Lamed, R. *J. Am. Chem. Soc.* **1986**, 1, 162-169.

[18] O. Trott, Arthur J. Olson, J. Comput. Chem. 2010, 31, 455.
